# Palladium catalyzed carbonylative generation of potent, pyridine-based acylating electrophiles for the functionalization of arenes to ketones†

**DOI:** 10.1039/d0sc03129a

**Published:** 2020-07-28

**Authors:** Yi Liu, Angela M. Kaiser, Bruce A. Arndtsen

**Affiliations:** Department of Chemistry, McGill University 801 Sherbrooke Street West Montreal QC H3A 0B8 Canada bruce.arndtsen@mcgill.ca

## Abstract

We describe here the design of a palladium catalyzed route to generate aryl ketones *via* the carbonylative coupling of (hetero)arenes and aryl- or vinyl-triflates. In this, the use of the large bite angle Xantphos ligand on palladium provides a unique avenue to balance the activation of the relatively strong C(sp^2^)–OTf bond with the ultimate elimination of a new class of potent Friedel–Crafts acylating agent: *N*-acyl pyridinium salts. The latter can be exploited to modulate reactivity and selectivity in carbonylative arene functionalization chemistry, and allow the efficient synthesis of ketones with a diverse array of (hetero)arenes.

## Abstract 2

A palladium catalyzed approach to the overall carbonylative functionalization of arenes to form ketones with aryl- and vinyl-triflates is described.

## Graphical abstract

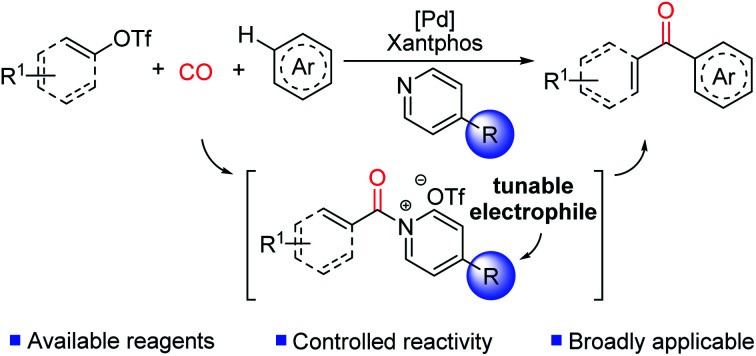

## Introduction

**Fig. 1:**
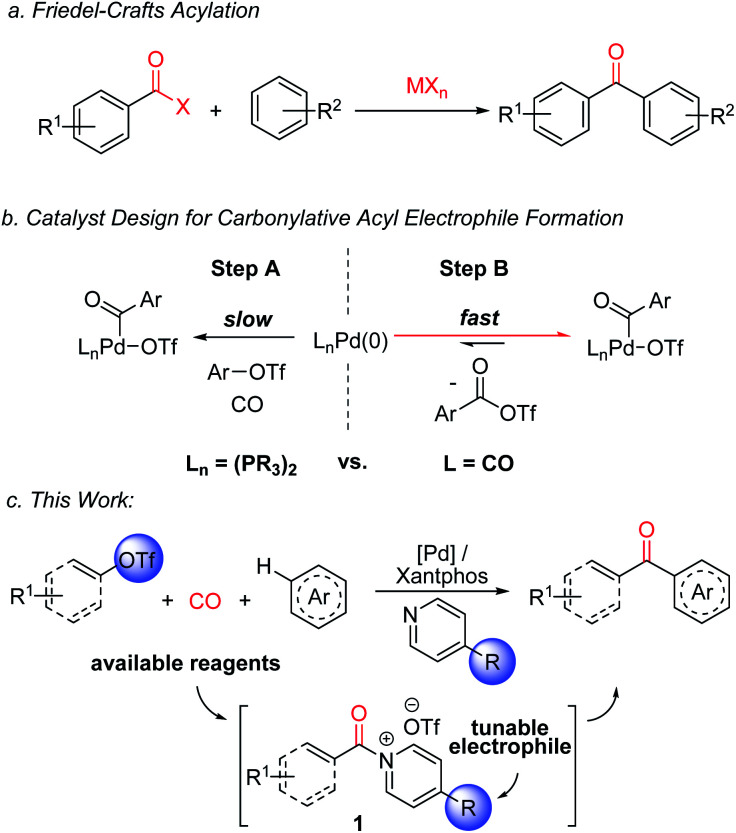
Friedel–Crafts acylations and this work: carbonylative generation of *N*-acyl pyridinium salts for C–H functionalization.

The design of catalytic methods for the efficient and selective functionalization of aromatic C–H bonds represents an important current thrust in synthetic chemistry.^1^ Despite many significant recent advances in this area, adapting these transformations to carbonylative ketone synthesis has seen less success.^2^ Aryl ketones are instead often generated from arenes with traditional Friedel–Crafts acylations using potent acyl electrophiles (Fig. 1a).^3^ Friedel–Crafts reactions have several attractive features, including their versatility, ability to proceed in an intermolecular fashion, predictable selectivity, and reactivity without directing groups. Nevertheless, a limitation of this chemistry is the need to generate the electrophiles themselves. The latter can require multiple steps and use high energy reagents (*e.g.* thionyl chloride, oxalyl chloride, or carbodiimides).^4^ Moreover, stoichiometric Lewis acids are usually employed to mediate Friedel–Crafts couplings, which leads to compatibility problems with basic functionalities or even decomposition, and generates significant metal-containing waste.^5^ These limitations are of relevance, since aryl ketones are useful motifs in a range of areas, including pharmaceuticals, photosensitizers and polymers.^6^

Recent studies by a number of labs including our own have described how carbonylations can offer an efficient alternative approach to the generation of potent acylating electrophiles.^7–10^ The reactivity of these products has allowed the application of carbonylation reactions to a range of challenging substrates, including the direct functionalization of arenes with acyl triflates.^8*e*^ However, as with classical Friedel–Crafts chemistry, the reaction here also requires the use of stoichiometric metal additives: in this case silver triflate salts, in concert with reactive aryl iodides. In addition, the acyl triflates generated are potent electrophiles, and offer little opportunity to control their reactivity without changing the substrate itself.

From an efficiency standpoint, a potential solution to these issues would be to directly carbonylate aryl triflates to aroyl electrophiles. Such a system would obviate the need for metal triflate salts, and also allow the use of broadly available aryl- or vinyl-triflate building blocks, which can be generated in an efficient fashion from the corresponding phenols or ketones, respectively.^11,12^ Unfortunately, this transformation presents a significant catalyst design challenge (Fig. 1b). The aryl triflate bond is much more robust than that in aryl iodides (*ca.* 100 *vs.* 65 kcal mol^−1^),^13^ and typically requires strong donor and chelating ligands on palladium to favor its activation in the presence of coordinating and π-acidic CO (Fig. 1b, Step A).^14^ The counter to this step, the product liberating reductive elimination of the highly electrophilic aroyl triflate from palladium, is a strongly disfavoured equilibrium, and has only been noted with the opposite catalyst, an electron deficient palladium species (*e.g.* (CO)_*n*_Pd, Step B).^8*e*^ This leads to the seemingly contradictory, and to our knowledge unprecedented, need for a catalyst that can activate a relatively strong C(sp^2^)–OTf bond, at the same time eliminate a kinetically reactive acylating agent, and do so under the conditions required for arene functionalization.

We describe herein our studies towards such a system. These illustrate how balancing ligand steric and electronic features, in concert with the correct additives, can open a palladium catalyzed route to build-up highly reactive acylating electrophiles from C(sp^2^)–triflates. Synthetically, the reactivity of these electrophiles offers an efficient route to form ketones from broadly available (hetero)arenes, aryl/vinyl triflates and carbon monoxide, and do so without the need for stoichiometric metal additives or expensive reagents. In addition, mechanistic studies suggest this reaction generates a new class of Friedel–Crafts electrophile: *N*-acyl pyridinium salts (**1**,Fig. 1c). These acylating agents possess several useful features, including the ability to functionalize arenes under non-acidic conditions, do so without Lewis acid additives, and tune reactivity or selectivity with the pyridine employed.

## Results and discussion

**Fig. 2:**
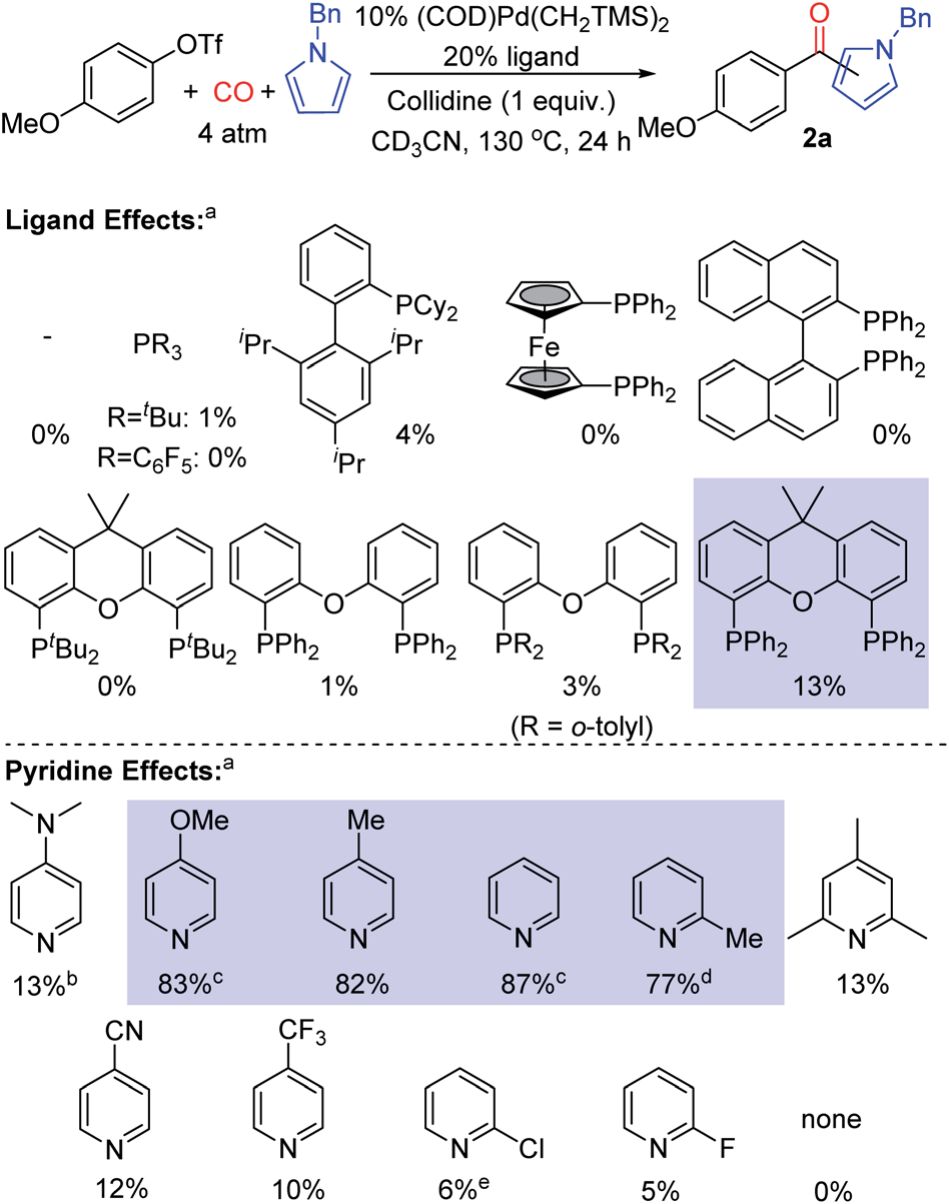
Ligand and pyridine effects on palladium catalyzed carbonylative pyrrole functionalization. ^a^NMR yields, *ca.* 2 : 1 ratio of 2-: 3-isomers of **2a**. ^b^With 5% [Pd(allyl)Cl]_2_ and 2 equiv. of DMAP; ^c^46 h; ^d^40 h; ^e^17 h.

Our initial studies examined the palladium catalyzed coupling of an aryl triflate with *N*-benzyl pyrrole and carbon monoxide (Fig. 2). Using a palladium catalyst without any added phosphine ligand, in analogy to results with aryl iodide reagents,^8*e*^ leads to no ketone product, nor does the use of various monodentate phosphine ligands. Instead, the unreacted aryl triflate is recovered. We next turned to bidentate ligands, as these have been demonstrated to allow the oxidative addition of aryl triflates in traditional carbonylations.^14^ Many of these were similarly ineffective, which may reflect the now difficult reductive elimination of the reactive aroyl triflates from palladium.^8*e*^ We considered using steric effects to balance the electronic needs of oxidative addition with the ultimate reductive elimination of a reactive acyl electrophile.^8^ After examining various sterically encumbered bidentate ligands, we were pleased to find that the large bite angle, moderate donor Xantphos allowed the intermolecular functionalization of *N*-benzyl pyrrole to afford ketone **2a** in low yield (13%).^15^

In probing approaches to enhance the yield of the reaction, one simple modification was to change the base employed. This was found to dramatically influence reactivity. For example, catalysis with electron deficient pyridines such as 4-trifluoromethyl-, 2-chloro- or 2-fluoropyridine leads to diminished yields (5–10%, Fig. 2). Electron rich 4-dimethylaminopyridine (DMAP) is also poorly effective (13%), and no ketone **2a** is observed without a pyridine additive (see Fig. S1† for full list of pyridines examined). However, using pyridines between these two extremes of nucleophilicity, such as 4-methoxypyridine and simple pyridine, can allow the overall functionalization of pyrrole and ketone generation with high efficiency (up to 87% yield).^16^

**Fig. 3:**
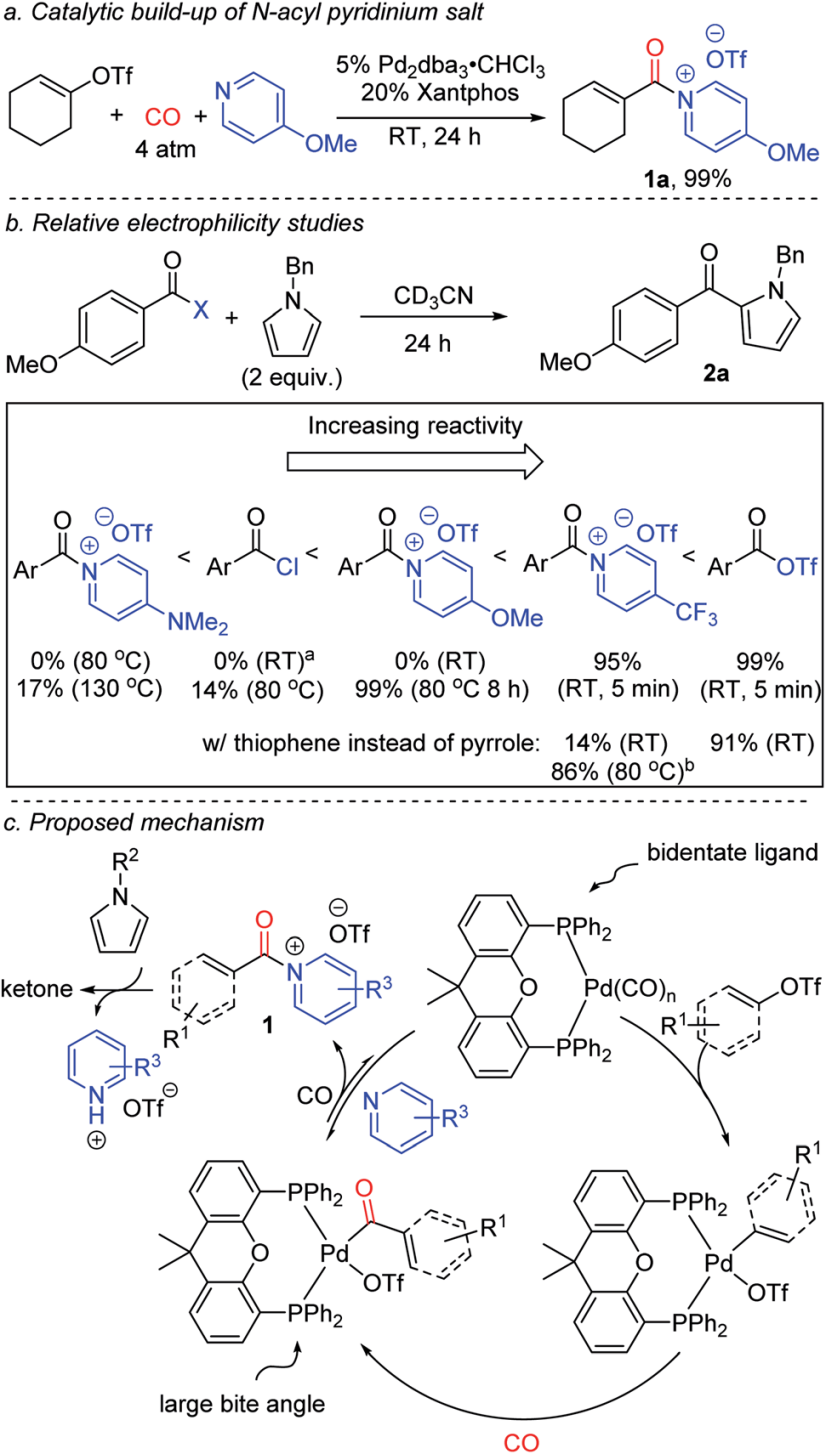
Mechanistic experiments on the carbonylative formation and reactivity of *N*-acyl pyridinium salts. ^a^Reactions with acid chlorides were performed in the presence of 1 equivalent of 2,4,6-collidine. ^b^1 h.

The dramatic influence of the pyridine derivative employed in these reactions is unusual, and suggests it may play a more direct role than simply that of a base. Acylating electrophiles are established to react with pyridine to form *N*-acyl pyridinium salts.^17^ Indeed, control experiments show this association occurs between an *in situ* generated aroyl triflate and 4-methoxypyridine within minutes at ambient temperature (Fig. S2†). More conclusive evidence for the formation of *N*-acyl pyridinium salts (**1**) as intermediates can be seen by performing the palladium catalyzed carbonylation in the absence of the pyrrole trap. While aryl triflates react under these conditions to form a mixture of products (Fig. S3†), the carbonylation of a more reactive vinyl triflate leads to the formation of the *N*-acyl pyridinium salt **1a** (Fig. 3a).^9*n*,18^ The formation of **1a** provides a rationale for the significant role of the pyridine base in the chemistry, where the more nucleophilic pyridine can presumably favor reductive elimination relative to the potential build-up of a labile acyl triflate. However, this is counterbalanced by the ability of these systems to react with arenes. The latter can be seen as well in control experiments (Fig. 3b). The 4-trifluoromethyl substituted pyridinium salt reacts within minutes at ambient temperature with *N*-benzyl pyrrole to form ketone. Replacing the pyrrole with thiophene also leads to ketone (Fig. S4†), although the latter requires more pressing conditions than acyl triflates themselves. In contrast, the more electron rich 4-methoxy substituted pyridinium salt does not react with pyrrole at ambient temperature, and requires heating to 80 °C to form product. For comparison, acid chlorides show low reactivity, 14%, under these conditions, and the electron rich 4-dimethylamino substituted pyridinium salt requires even more pressing conditions (130 °C) to form ketone.

Based upon this data, we postulate that this carbonylative functionalization reaction proceeds as shown in Fig. 3c. The reactivity observed with the Xantphos ligand is presumably related in part to its ability to associate in a bidentate fashion to palladium and better activate the relatively strong C(sp^2^)–OTf bond towards carbonylation, in concert with its large bite angle, which can favor the reductive elimination.^8,19^ The combination of these features offers an avenue to build-up kinetically reactive acylating agents from C(sp^2^)–triflates.^20^ The importance of pyridine, and the specific pyridine structure, in catalysis is tied to its ability to stabilize the reductive elimination product relative to a highly reactive acid triflate. The adducts **1** represent a new class of Friedel–Crafts acylating reagent, and incorporate several attractive features, including the ability to react with (hetero)arenes without added Lewis acids, and tune Friedel–Crafts acylation reactivity with the pyridine employed (*vide infra*).

**Fig. 4:**
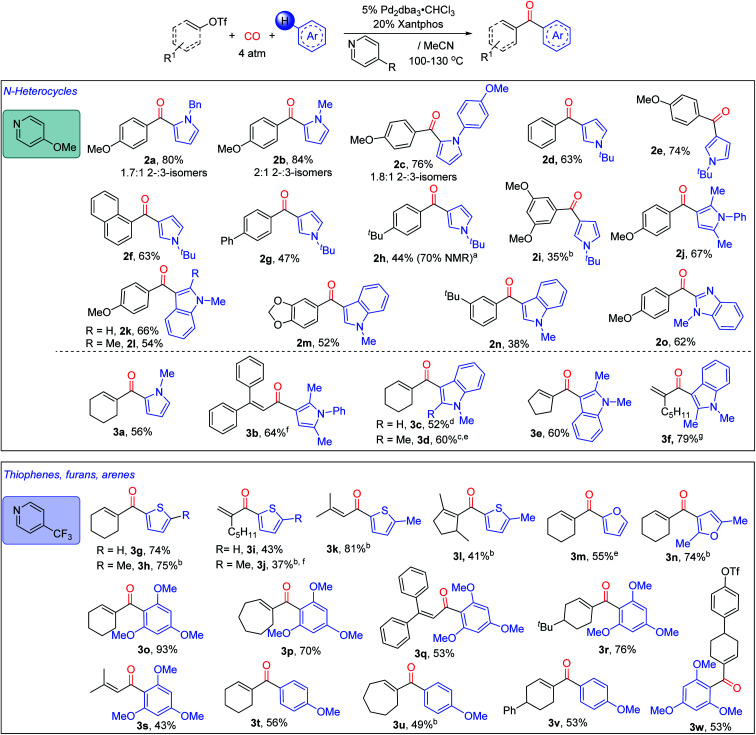
Diversity of products available from the Pd catalyzed C–H functionalization of (hetero)arenes with organotriflates. Performed with 2 equiv. aryl or vinyl triflates for pyrroles and indoles, 1 equiv. with other (hetero)arenes, 4 atm CO, 130 °C; 10% (COD)Pd(CH_2_TMS)_2_, 48 h for aryl triflates; using 5% Pd_2_dba_3_·CHCl_3_, for vinyl triflates. ^a^Difficult isolation due to the presence of phenyl ketone **2d**; ^b^10% Pd(Xantphos)_2_; ^c^1.25% Pd_2_dba_3_·CHCl_3_; ^d^150 °C; ^e^100 °C; ^f^80 °C; ^g^25 °C.

The *in situ* generation of *N*-acyl pyridinium salts offers a versatile approach to the overall carbonylative conversion of arenes to ketones. As shown in Fig. 4, various pyrroles can be employed in this reaction in concert with 4-methoxypyridine, such as those with *N*-benzyl, -methyl and -aryl substituents. These each lead to the favored formation of the 2-substituted ketone product (**2a–2c**), which is consistent with a Friedel–Crafts mechanism of reaction.^4*a*,21^ The more sterically encumbered *N-tert*-butyl pyrrole is functionalized with high selectivity at the 3-position (**2d–i**). Disubstituted pyrroles such as 2,5-dimethyl-*N*-phenyl pyrrole also undergo efficient reaction to form ketone **2j**. This chemistry can be applied to other nitrogen heterocycles such as substituted indoles, (**2k–n**), and *N*-substituted benzimidazole (**2o**). In addition to the variation of the heteroarenes, a number of aryl triflates can be employed in the reaction, including 4-methoxy, -phenyl (**2g**) and -alkyl substituted (**2h**) reagents. Meta-substituted aryl triflates are tolerated (**2i**, **2m**, and **2n**), albeit with diminished yields. Catalysis also proceeds with less electron rich substrates, such as simple phenyl (**2d**) or naphthyl triflate (**2f**). However, the use of aryl triflates with electron-withdrawing substituents such as 4-cyano and 4-trifluoromethyl substituted aryl triflates leads instead to significant amounts of the phenyl-substituted ketone (**2d**) (44% and 43%, respectively, see Fig. S5†). The latter presumably arises from the ability of the palladium-aryl intermediate to scramble substituents with the Xantphos ligand.^8*h*–*i*,22^ Similarly, less polar aryl mesylates are not reactive under these conditions (Fig. S6†).

We next explored the compatibility of this system with vinyl triflates as a route to access α,β-unsaturated ketones. The transformation here is more efficient than reactions with aryl triflates, and coupling with pyrroles and indoles can proceed at ambient temperature with some substrates (*e.g.***3f**). As with the aryl triflates, the reaction is compatible with *N*-substituted (**3a**) or 2,5-disubstituted pyrroles (**3b**), and similarly functionalized indoles using cyclic (**3c–e**) or acyclic (**3b** and **3f**) vinyl triflate reagents. These allow access to various α- or β,β-substituted acyclic enones.

In addition, the pyridine unit can be used to modulate electrophilic reactivity. Thus, while less activated (hetero)arenes (*e.g.* thiophenes or substituted arenes) do not lead to product formation using 4-methoxypyridine, the use of the weakly nucleophilic 4-trifluoromethylpyridine can allow the carbonylative C–H functionalization of various other classes of aromatic reagents. The more reactive vinyl triflates are employed here to allow the build-up of these less stable *N*-acyl pyridinium salts. Examples of hetero(arenes) that can now be functionalized include substituted thiophenes (**3g–l**), furans (**3m**, **3n**), and even substituted arenes, such as 1,3,5-trimethoxybenzene (**3o–s**, **3w**) and anisole (**3t–v**), each of which undergo C–H functionalization to afford the corresponding α,β-unsaturated ketone. This reactivity is compatible with a range of cyclic vinyl triflates (**3g**, **3l**, **3p**). Moreover, acyclic vinyl triflates bearing α- or/and β- (**3b**, **3f**), alkyl- (**3f**, **3k**) or aryl- (**3b**) substituents can also proceed to generate ketone products. Interestingly, by using 4-trifluoromethylpyridine, the vinyl triflate bond can be selectively activated in the presence of an aryl triflate (**3w**). Together, this system provides access to an array of ketones *via* the palladium catalyzed carbonylative functionalization of arenes with accessible organic triflates.

**Fig. 5:**
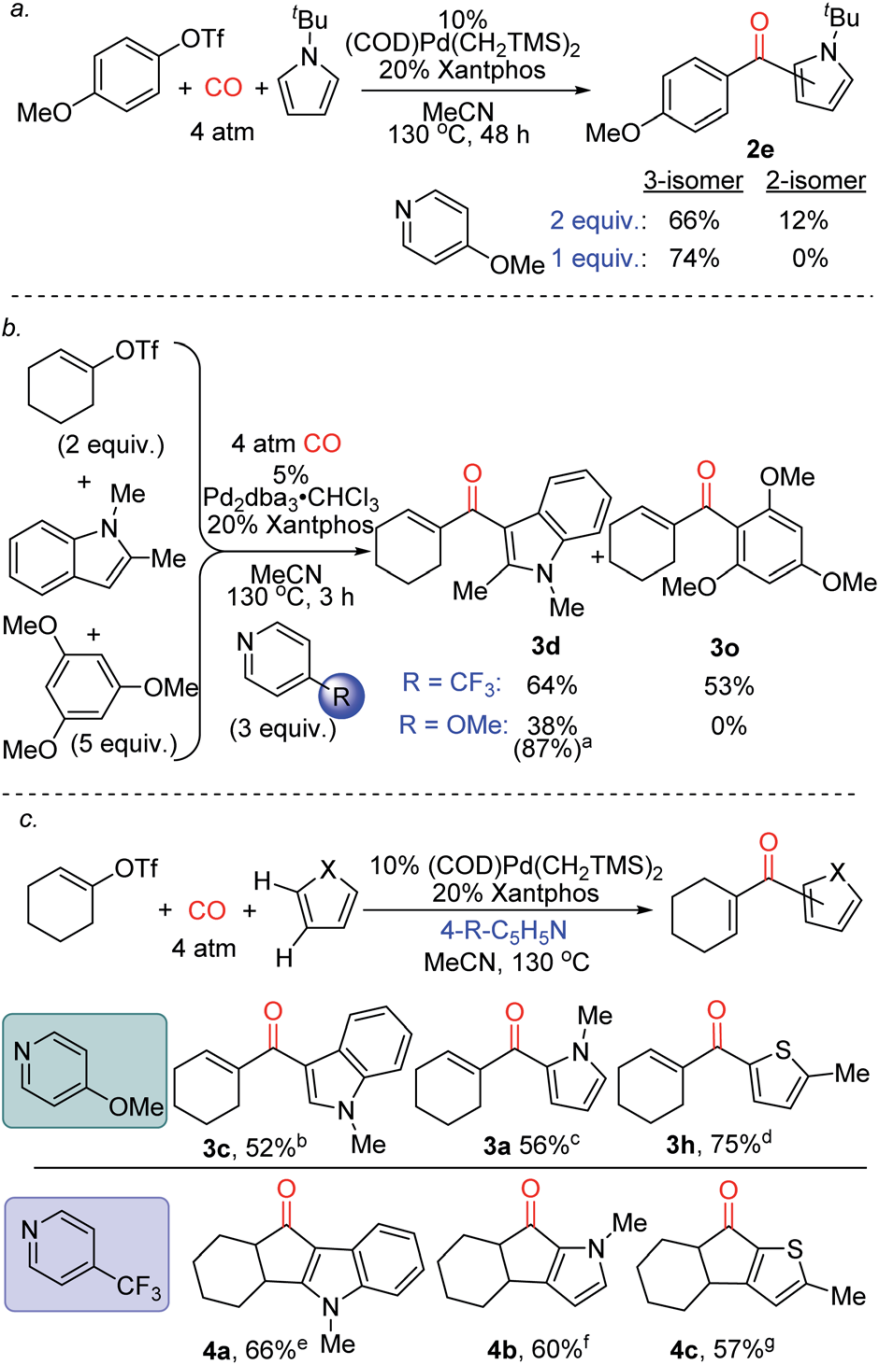
Selectivity control in carbonylative C–H functionalization with pyridine employed. ^a^1.1 equiv. 4-methoxypyridine, 100 °C, 2.5 h, 17% **3o** also formed; ^b^5% Pd_2_dba_3_·CHCl_3_,150 °C, 12 min; ^c^5% Pd_2_dba_3_·CHCl_3_, 20 min; ^d^10% Pd(Xantphos)_2_, 4-trifluoromethylpyridine, 16 h; ^e^20 h; ^f^4 h; ^g^48 h.

Finally, we have preliminarily probed the ability of the pyridine to fine-tune product selectivity. One simple effect is through concentration, where lowering the amount of pyridine employed favours the formation of 3-substituted ketone **2e** for pyrroles bearing bulky *N*-substituents (Fig. 5a). The latter may affect the rate of deprotonation of the Wheland intermediate, and thereby allow migration of the aroyl fragment to the favoured 3-position under less basic conditions.^23^ Alternatively, the pyridine can be used to influence chemoselectivity. An illustration is in the reaction of 1-cyclohexenyltriflate with both *N*-methyl indole and 1,3,5-trimethoxybenzene (Fig. 5b). While the use of 4-trifluoromethylpyridine leads to a mixture of products, 4-methoxypyridine moderates the electrophilicity of the intermediate formed, and allows functionalization to occur exclusively on the more activated indole substrate. The pyridine base can also affect the reactivity of the products (Fig. 5c). As previously noted, the catalytic formation of these products can be performed using 4-methoxypyridine. In contrast, employing the less nucleophilic 4-trifluoromethylpyridine can divert this reaction to instead generate cyclized products of indoles, pyrroles and thiophenes (**4a–c**). The generation of **4a–c** presumably arises from the ability of these systems to undergo acid catalyzed Nazarov cyclization,^24^ and offers a method to functionalize two C–H bonds at once to build up polycyclic products from heterocycles, CO and vinyl triflates.

## Conclusions

In summary, we have described a new approach to the palladium catalyzed carbonylative generation of potent electrophiles with aryl and vinyl triflates for the overall functionalization of arenes. The reaction offers a route to access ketones from combinations of available substrates (organotriflates, (hetero)arenes, CO), and without the typical need for Lewis acids or stoichiometric metal-containing reagents. Mechanistic analysis shows that the Pd/Xantphos catalyst system, in concert with pyridine additives, can provide an unusual avenue to balance the activation of relatively strong C(sp^2^)–OTf bonds with the ultimate elimination of reactive acylating agents. In this, the pyridine additive serves not only as a base, but more crucially as a building block to drive the formation of a new class of Friedel–Crafts reagent: *N*-acyl pyridinium salts. The latter can be exploited to modulate reactivity and product selectivity in carbonylative functionalization chemistry with a diverse array of (hetero)arenes.

## Conflicts of interest

There are no conflicts to declare.

## Supplementary Material

SC-011-D0SC03129A-s001
