# Supplementary material for: Palladium catalyzed carbonylative generation of potent, pyridine-based acylating electrophiles for the functionalization of arenes to ketones
Source: Chem Sci. 2020 Jul 28;11(32):8610–6. doi: 10.1039/d0sc03129a (PMC8163404; doi:10.1039/d0sc03129a)
Supplement: SC-011-D0SC03129A-s001 [file SC-011-D0SC03129A-s001.pdf]

Electronic Supplementary Material (ESI) for Chemical Science.
This journal is © The Royal Society of Chemistry 2020

Palladium Catalyzed Carbonylative Generation of Potent, Pyridine-Based Acylating Electrophiles for the Functionalization of Arenes to Ketones

Yi Liu, Angela M. Kaiser and Bruce A. Arndtsen*

Department of Chemistry, McGill University, 801 Sherbrooke Street West, Montreal, QC, Canada, H3A 0B8

I. General Considerations 1
II. Supplementary Figures 3
III. Experimental Procedures 5
1. Typical procedure for reaction development (Figure 2) 5
2. Typical procedure for the synthesis of *N*-acyl pyridinium salts (Figure S2) 6
3. Catalytic formation of *N*-acyl pyridinium salts (Figure 3a and S3) 7
4. Reactions of aryl electrophiles with heteroarenes (Figure 3b) 10
5. Typical catalytic carbonylation procedures (Figure 4) 18
6. Selectivity control in carbonylative C-H functionalization (Figure 5) 21
7. Synthesis of aryl triflates 23
8. Synthesis of vinyl triflates 23
IV. Characterization Data for Ketones 27
V. References 36
VI. NMR Spectra 37

I. General Considerations

All manipulations were carried out in an inert atmosphere glovebox or using standard Schlenk techniques, unless stated otherwise. All reagents were purchased from commercial sources, unless otherwise noted. Prior to use in catalysis, all liquid substrates were degassed, transferred to a glovebox, and dried with 4Å molecular sieves, while solid reagents were transferred to a glovebox under nitrogen, dissolved in dichloromethane, stirred over 4Å molecular sieves, then filtered and the solvent removed *in vacuo*. Solvents were dried by using a solvent purification system and stored over activated 4Å molecular sieves inside the glovebox. Deuterated acetonitrile was stirred over calcium hydride, vacuum transferred, degassed, and stored over 4Å molecular sieves. $Pd_2dba_3{\cdot}CHCl_3$[1]

and (COD)Pd(CH_2TMS)$_2$[2] were prepared according to literature procedures and stored at -35 °C in the glovebox to avoid decomposition. *N*-substituted heterocycles,[3] and vinyl[4] and aryl triflates[5] were prepared according to literature procedures, or purchased when possible. The reported syntheses of *N*-^{t}Bu and *N*-benzyl pyrroles were slightly modified, where the reaction was performed at 150 °C for 3 h. Purification of *N*-^{t}Bu pyrrole was modified from that reported by using vacuum distillation (25 °C, static vacuum at 50 mTorr.) rather than column chromatography. All other reagents were purchased from commercial suppliers and used as received after thoroughly drying to remove all traces of water. Research grade carbon monoxide (CO) (99.99%) was used as received. *Warning: CO is a poisonous gas that requires all the experiments to be conducted in well-ventilated fume hoods.* For reactions performed in a J-Young NMR tube, carbon monoxide was added by attaching the J-Young tube to a Schlenk line of a known internal gas volume (67 mL) equipped with a pressure gauge. The NMR tube solution was frozen in liquid nitrogen, the headspace evacuated, the tube closed, and the Schlenk line was filled with 800 mTorr of CO. In order to condense 4 atm CO into the 2.2 mL headspace of the NMR tube, the tube was opened until a pressure drop of 120 mTorr was recorded on the Schlenk line (corresponding to 0.44 mmol of CO; which equals 4 atm CO in the J-Young NMR tube based on the ideal gas law). For reactions in Schlenk bombs at 4 atm CO, 4 atm CO was added to the existing atmosphere of nitrogen. Nuclear magnetic resonance (NMR) characterization was performed on 400 or 500 MHz spectrometers for ^{1}H NMR, 101 or 126 MHz for ^{13}C NMR, and 377 or 471 MHz for ^{19}F NMR. ^{1}H and ^{13}C NMR chemical shifts were referenced to residual solvent. Mass spectra were recorded on a high-resolution electrospray ionization quadrupole mass spectrometer.

II. Supplementary Figures

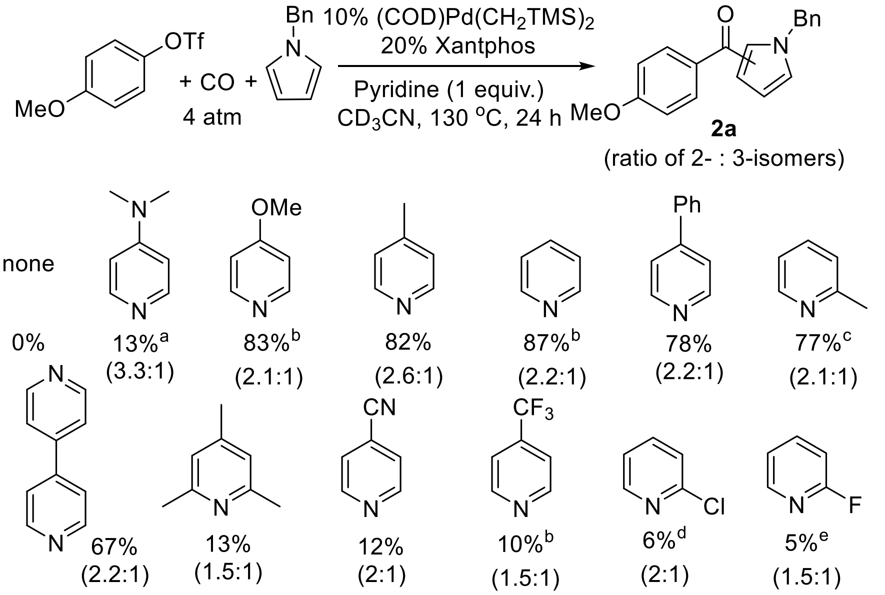

Figure S1. Pyridine influence on Pd catalyzed carbonylative C-H bond functionalization of *N*-benzyl pyrrole. [a] with $[Pd(allyl)Cl]_2$ and 2 equiv. of DMAP; [b.]46h; [c.]40h; [d.]17h.

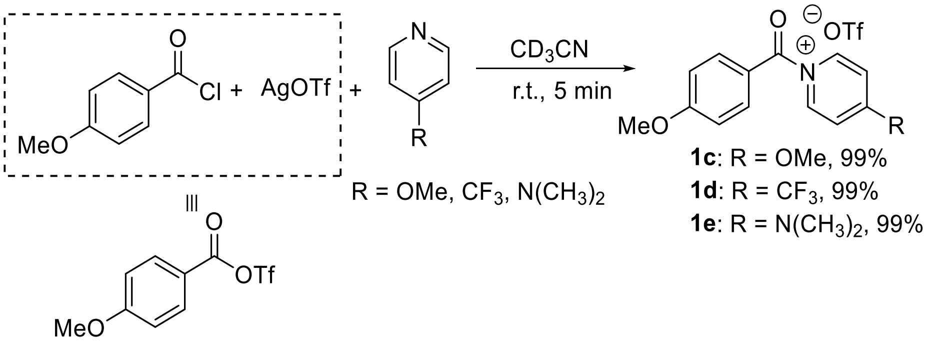

Figure S2. Reactions of pyridines with *in-situ* generated acid triflate.

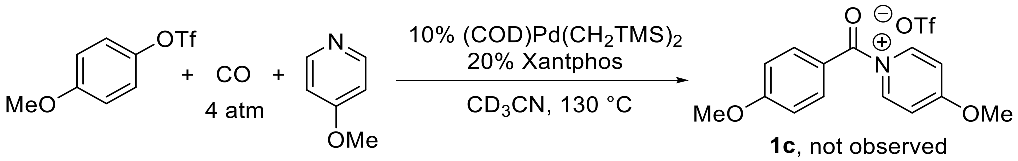

^{1}H NMR:

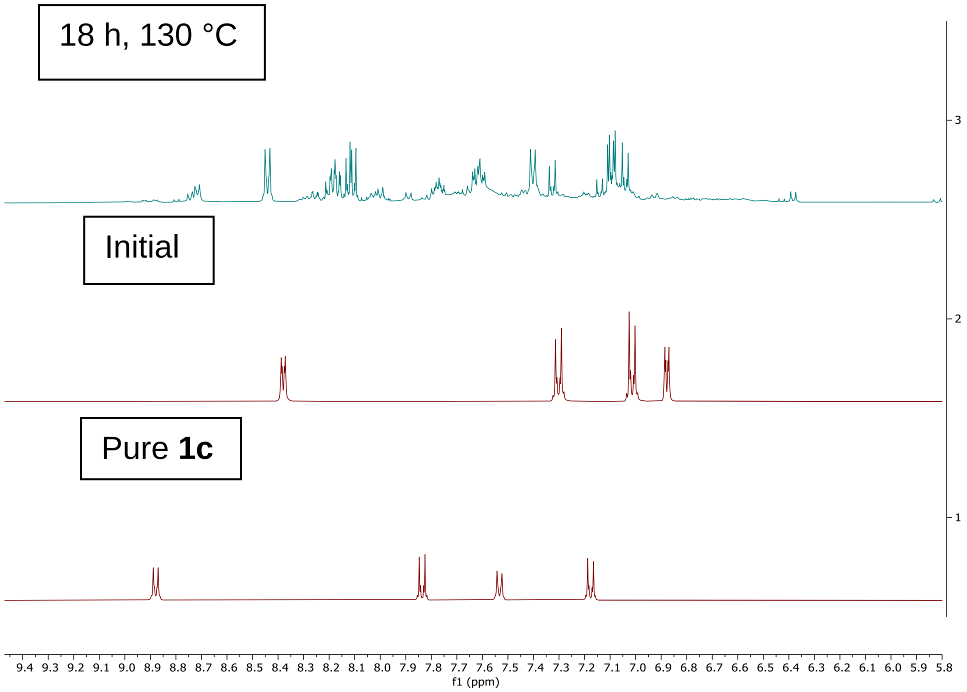

Figure S3. Attempting the catalytic build-up of aroyl pyridinium salt.

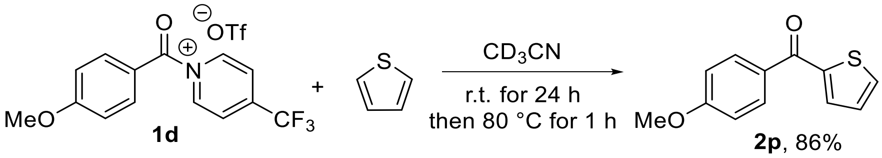

Figure S4. The reaction of *N*-acyl pyridinium salt 1d with thiophene.

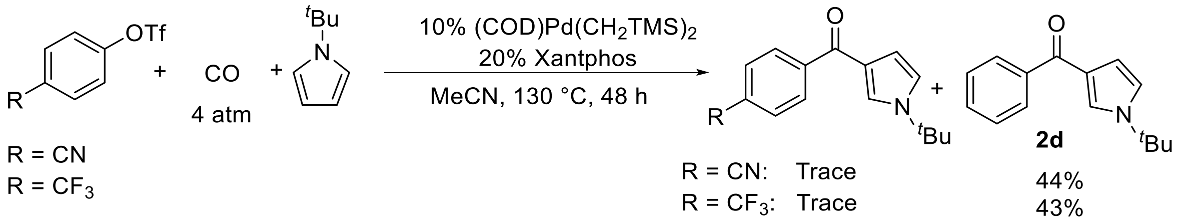

Figure S5. Reactions with electron deficient aryl triflates.

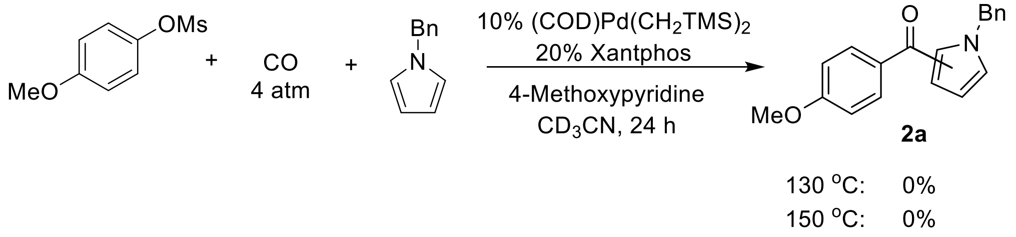

Figure S6. Reactions with 4-methoxyphenyl mesylate.

III. Experimental Procedures

1. Typical procedure for reaction development (Figure 2)

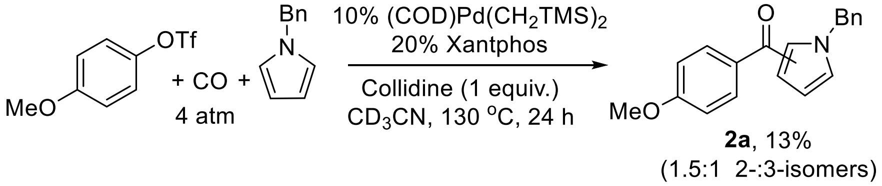

In a glovebox, 4-(methoxy)phenyl trifluoromethylsulfonate (triflate) (51 mg, 0.20 mmol), *N*-benzyl pyrrole (16 mg, 0.10 mmol), 2,4,6-collidine (12 mg, 0.10 mmol), $(COD)Pd(CH_2TMS)_2$ (3.9 mg, 0.010 mmol), Xantphos (11.6 mg, 0.020 mmol), and dimethyl sulfone standard (3 mg, 0.03 mmol) were mixed in a 5 mL vial with 0.8 mL CD_3CN. This solution was transferred into a J-Young NMR tube. The tube was closed, taken out of the glovebox, frozen under liquid nitrogen. The headspace was evacuated and then 4 atm of CO were condensed into the tube. (As noted in the general procedures, this was accomplished by condensing 120 mTorr of a CO filled vacuum line (67 mL volume) into the NMR tube (headspace 2.2 mL). The reaction was heated at 130 °C and monitored by ^1H NMR for 24 h, after which the yield of **2a**, 13% (1.5:1, 2- : 3-isomers of **2a**), was determined by ^1H NMR analysis relative to the internal standard.

2. Typical procedure for the synthesis of *N*-acyl pyridinium salts (Figure S2)

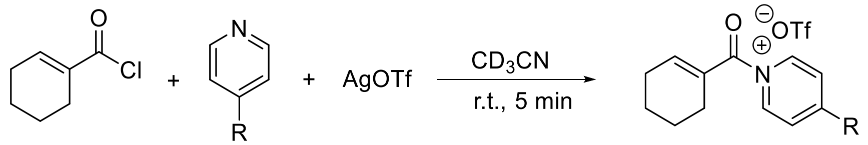

R = OMe, CF_3

1a: R = OMe, 99%
1b: R = CF_3, 99%

In a glovebox, cyclohex-1-ene-1-carbonyl chloride (obtained by the procedure described below, 7.2 mg, 0.050 mmol) and 4-(methoxy)pyridine (5.4 mg, 0.050 mmol) were added to a 5 mL vial equipped with a stir bar with 0.5 mL CD_3CN, followed by the addition of a AgOTf (14 mg, 0.053 mmol) solution in 0.3 mL CD_3CN. White precipitate formed immediately, and the mixture was stirred for additional 2 minutes. The mixture was filtered to gain the solution of **1a**, which was then characterized *in situ*. In order to determine the yield, a similar reaction was carried out in the presence of dimethyl sulfone (2.8 mg, 0.03 mmol) as the internal standard, based on which the yield was determined by ^{1}H NMR analysis.

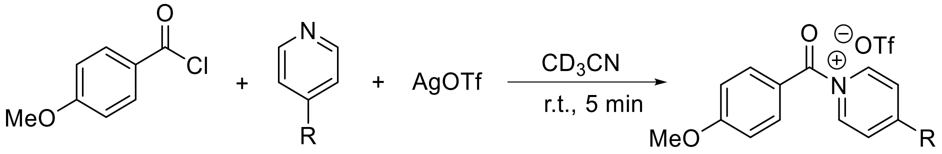

R = OMe, CF_3, $N(CH_3)_2$

1c: R = OMe, 99%
1d: R = CF_3, 99%
1e: R = $N(CH_3)_2$, 99%

Aroyl pyridinium salts **1c-e** were synthesized using similar procedures as described above.

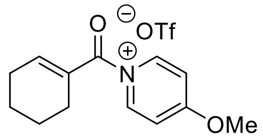

1-(cyclohex-1-ene-1-carbonyl)-4-methoxypyridin-1-ium trifluoromethanesulfonate (1a). NMR yield: 99%. ^{1}H NMR (500 MHz, Acetonitrile-*d*3) δ 8.84 (d, *J* = 7.5 Hz, 2H), 7.48 (d, *J* = 7.4 Hz, 2H), 6.72 (td, J = 4.0, 2.0 Hz, 1H), 4.20 (s, 3H), 2.48 – 2.44 (m, 2H), 2.40 – 2.35 (m, 2H), 1.80 – 1.75 (m, 2H), 1.73 – 1.68 (m, 2H). ^{13}C NMR (126 MHz, Acetonitrile-*d*3) δ 176.3, 168.0, 153.0, 144.7, 130.4, 113.7, 60.0, 27.3, 25.4, 22.1, 21.4. ^{19}F NMR (471 MHz, Acetonitrile-*d*3) δ -79.31. HRMS. Calculated for $C_{13}H_{16}NO_2^+$ (M^+): 218.1176, found: 218.1182.

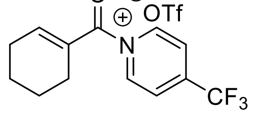

1-(cyclohex-1-ene-1-carbonyl)-4-(trifluoromethyl)pyridin-1-ium trifluoromethanesulfonate (1b) NMR yield: 99%. ^{1}H NMR (500 MHz, Acetonitrile-*d*3) δ 9.29 (d, *J* = 6.7 Hz, 2H), 8.49 (d, *J* = 6.4 Hz, 2H), 6.82 (td, *J* = 4.0, 2.0 Hz, 1H), 2.53 – 2.49 (m, 2H), 2.42 – 2.38 (m, 2H), 1.83 – 1.77 (m, 2H), 1.76 – 1.69 (m, 2H). ^{13}C NMR (126 MHz, Acetonitrile-*d*3) δ 166.3, 157.8, 145.8, 129.9, 126.1 (q, *J* = 3.4 Hz), 123.1, 120.9, 27.9, 25.2, 21.9, 21.2. ^{19}F NMR (471 MHz, Acetonitrile-*d*3) δ -66.09, -79.34. HRMS. Calculated for $C_{13}H_{13}F_3NO^+$ (M^+): 256.0944, found: 256.0941.

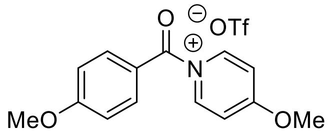

4-methoxy-1-(4-methoxybenzoyl)pyridin-1-ium trifluoromethanesulfonate (1c) ^{1}H NMR (500 MHz, CD_3CN) δ 8.88 (d, *J* = 7.8 Hz, 2H), 7.84 (d, *J* = 9.0 Hz, 2H), 7.54 (s, 2H), 7.18 (d, *J* = 9.0 Hz, 2H), 4.24 (s, 3H), 3.95 (s, 3H). ^{13}C NMR (126 MHz, CD_3CN) δ 176.6, 167.11, 167.09, 144.9, 135.7, 120.0, 116.0, 113.8, 60.1, 56.9. ^{19}F NMR (377 MHz, CD_3CN) δ -79.29. HRMS. Calculated for $C_{14}H_{14}NO_3^+$ (M^+): 244.0968, found: 244.0968.

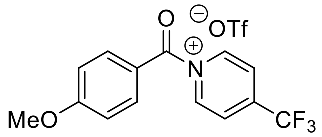

1-(4-methoxybenzoyl)-4-(trifluoromethyl)pyridin-1-ium trifluoromethanesulfonate (1d) ^{1}H NMR (500 MHz, CD_3CN) δ 9.36 (d, *J* = 6.7 Hz, 2H), 8.54 (d, *J* = 6.6 Hz, 2H), 7.89 (d, *J* = 9.1 Hz, 2H), 7.20 (d, *J* = 9.1 Hz, 2H), 3.97 (s, 3H). ^{13}C NMR (126 MHz, CD_3CN) δ 168.2, 165.6, 146.0, 136.7, 134.9, 126.2 (q, *J* = 3.6 Hz), 123.1, 120.9, 116.4, 57.2. ^{19}F NMR (471 MHz, CD_3CN) δ -66.05, -79.33. HRMS. Calculated for $C_{14}H_{11}F_3NO_2^+$ (M^+): 282.0736, found: 282.0730.

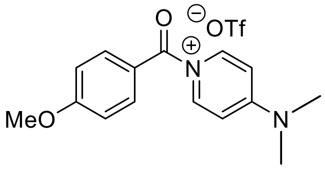

4-(dimethylamino)-1-(4-methoxybenzoyl)pyridin-1-ium (1e). ^{1}H NMR (500 MHz, CD_3CN) δ 8.42 – 8.31 (m, 2H), 7.82 – 7.72 (m, 2H), 7.18 – 7.11 (m, 2H), 6.99 – 6.90 (m, 2H), 3.93 (s, 3H), 3.33 (s, 6H). ^{13}C NMR (126 MHz, CD3CN) δ 168.15, 165.98, 159.37, 139.69, 134.61, 122.1 (q, J = 321.0 Hz), 121.44, 115.61, 107.86, 56.71, 41.58. HRMS. Calculated for $C_{15}H_{17}N_2O_2^+$ (M^+): 257.1285, found: 257.1291.

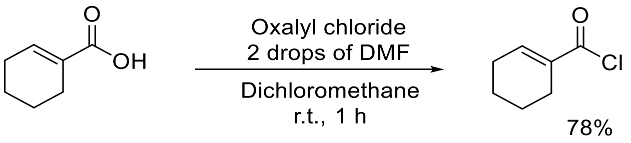

Under argon, cyclohex-1-ene-1-carboxylic acid (1.0 g, 8 mmol) and dry dichloromethane (10 mL) were added to a 50 mL round-bottom flask equipped with a magnetic stir bar. 2 drops of *N,N*-dimethyl formamide (DMF) were added to the stirred solution, followed by the addition of oxalyl chloride (1.6 mL, 18 mmol) dropwise over the course of 2 minutes via syringe. The reaction mixture was stirred at room temperature for 1 hour. The solvent and the excess of oxalyl chloride were removed *in vacuo*. The concentrated crude product was further purified by vacuum distillation (70 mTorr at 80°C) to afford cyclohex-1-ene-1-carbonyl chloride in 78% yield (0.90 g, 6.2 mmol) as a pale-yellow cloudy liquid (low melting point solid).

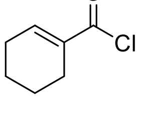

Cyclohex-1-ene-1-carbonyl chloride.[6] ^{1}H NMR (500 MHz, $CDCl_3$) δ 7.43 (td, *J* = 3.9, 1.9 Hz, 1H), 2.33 (qd, *J* = 7.4, 6.8, 3.1 Hz, 4H), 1.78 – 1.55 (m, 4H). ^{13}C NMR (126 MHz, $CDCl_3$) δ 168.4, 150.4, 135.2, 26.8, 25.3, 21.9, 21.0.

3. Catalytic formation of *N*-acyl pyridinium salts (Figure 3a and S3)

A. Attempting the catalytic build-up of aroyl pyridinium salt (Figure S3)

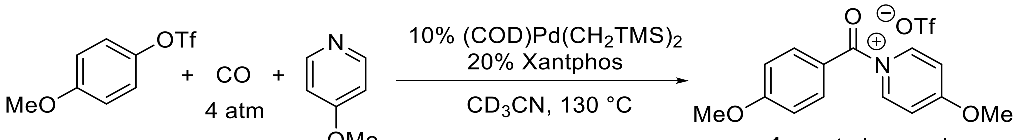

1c, not observed

In a glovebox, 4-(methoxy)phenyl triflate (26 mg, 0.1 mmol), 4-(methoxy)pyridine (11 mg, 0.1 mmol), (COD)Pd(CH_2TMS)$_2$ (3.9 mg, 0.01 mmol), Xantphos (11.6 mg, 0.02 mmol), and dimethyl sulfone standard (1.4 mg, 0.015 mmol) were mixed in a 5 mL vial with 0.8 mL CD_3CN. This solution was transferred into a J-Young NMR tube. The tube was closed, taken out of the glovebox, frozen under liquid nitrogen. The headspace was evacuated and then 4 atm CO were condensed into the tube. (As noted in the general procedures, this was accomplished by condensing 120 mTorr of a CO filled vacuum line (67 mL volume) into the NMR tube (headspace 2.2 mL). The NMR tube was heated at 130 °C and monitored by ^{1}H and ^{13}C NMR for 18 h. The formation of pyridinium salt **1c** was not observed. Instead, a mixture of unknown products is formed.

B. Catalytic build-up of vinoyl pyridinium salt (Figure 3a)

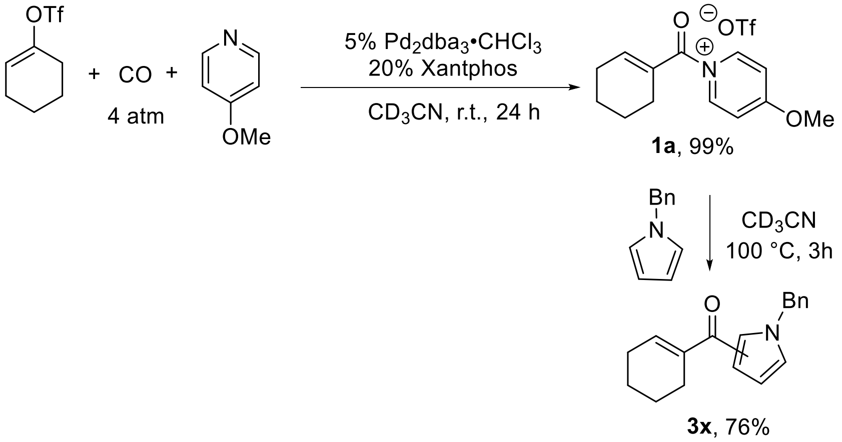

A similar procedure was conducted as above except using 1-cyclohexenyl triflate (25 mg, 0.11 mmol), 4-(methoxy)pyridine (11 mg, 0.10 mmol), Xantphos (11.6 mg, 0.020 mmol) and $Pd_2dba_3 \cdot CHCl_3$ (5.2 mg, 0.0050 mmol). The reaction was monitored by ^{1}H NMR at ambient temperature for 24 h, after which the yield of **1a,** 99%, was determined by ^{1}H NMR analysis relative to the internal standard. The tube was then frozen under liquid nitrogen and the headspace was evacuated to remove the CO. The NMR tube was brought back into the glovebox and a solution of *N*-benzyl pyrrole (31 mg, 0.2 mmol) in 0.2 mL CD_3CN was added to the NMR tube. The reaction was heated to 100 °C for 3 h and monitored by ^{1}H NMR, after which 76% of ketone **3x** (3.2:1 ratio of 2-:3-isomers) was obtained.

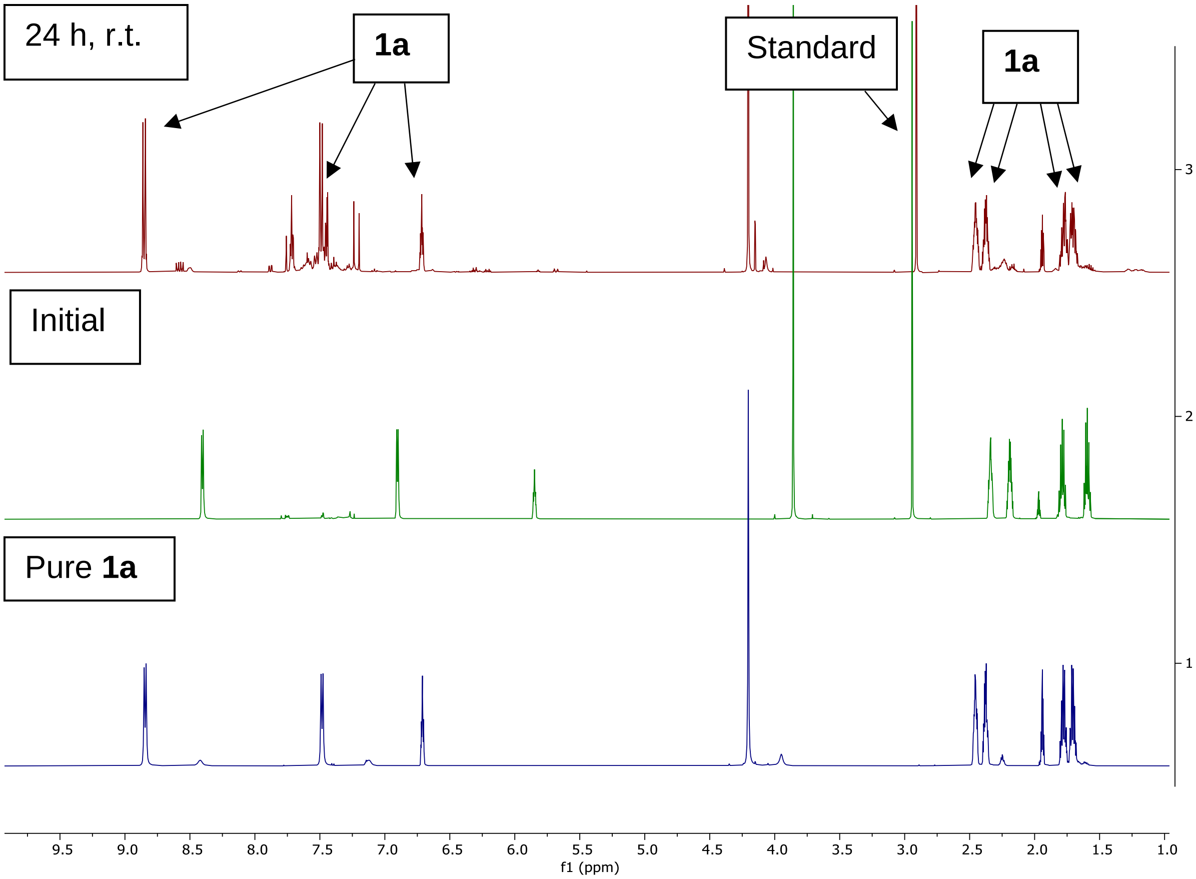

Figure S7. ^{1}H NMR of the reaction of 1-cyclohexenyl triflate, 4-(methoxy)pyridine and CO.

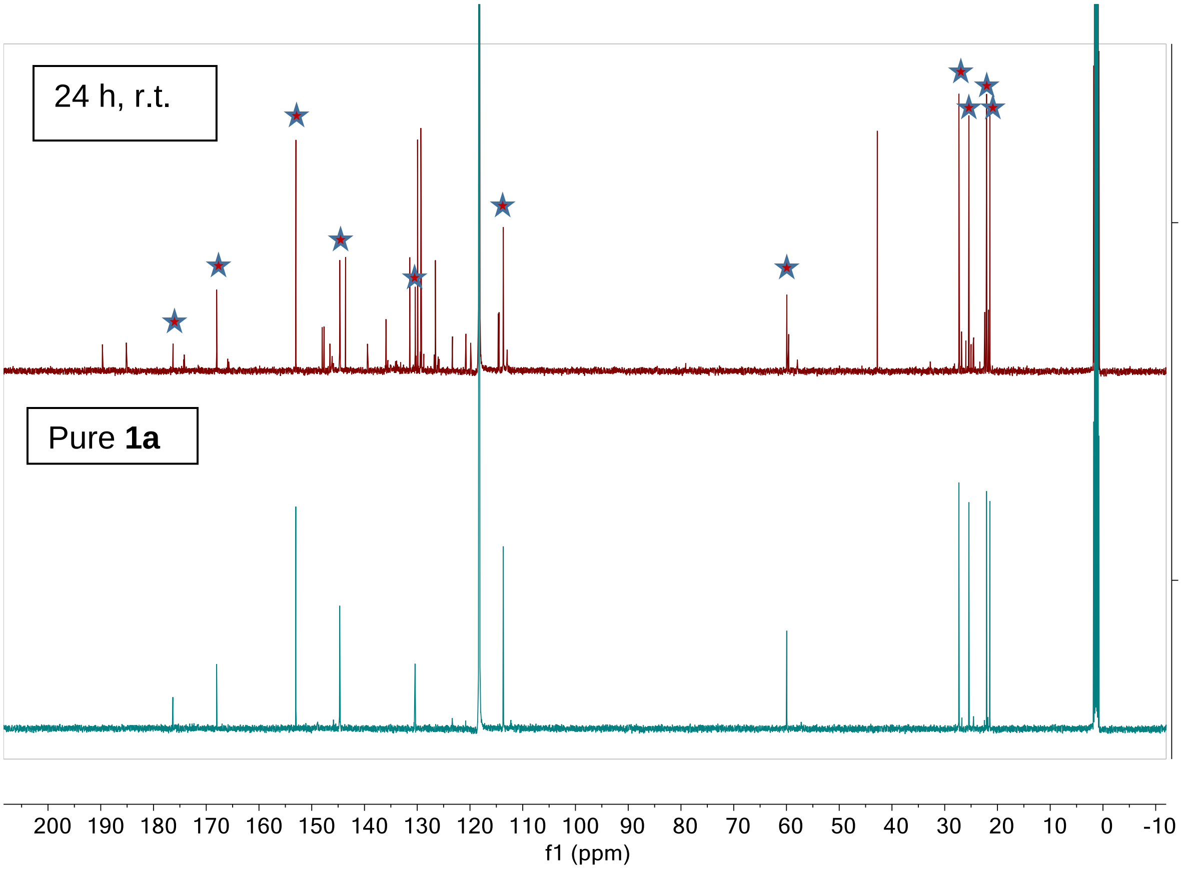

Figure S8. ^{13}C NMR of the reaction of 1-cyclohexenyl triflate, 4-(methoxy)pyridine and CO.

4. Reactions of aryl electrophiles with heteroarenes (Figure 3b)

A. The reaction of *p*-anisoyl chloride with *N*-benzylpyrrole.

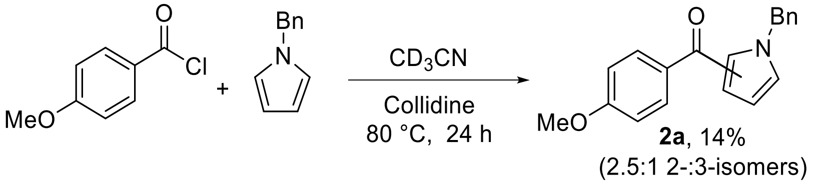

In a glovebox, *p*-anisoyl chloride (8.5 mg, 0.050 mmol), *N*-benzyl pyrrole (15.7 mg, 0.10 mmol), 2,4,6-collidine (6.1 mg, 0.050 mmol) and dimethyl sulfone standard (1.4 mg, 0.015 mmol) were mixed in a 5 mL vial with 0.8 mL CD_3CN. The solution was transferred into a J-Young NMR tube, brought out of the glovebox, and monitored at ambient temperature by ^1H NMR for 24 h, after which no formation of **2a** was observed. The reaction was then heated to 80°C for 24 h and monitored by ^1H NMR, after which ketones **2-2a** (2-isomer of **2a**, 10%) and **3-2a** (3-isomer of **2a**, 4%) were obtained in a 14% yield (2.5:1 ratio of 2-:3-isomers), as determined by ^1H NMR analysis relative to the internal standard.

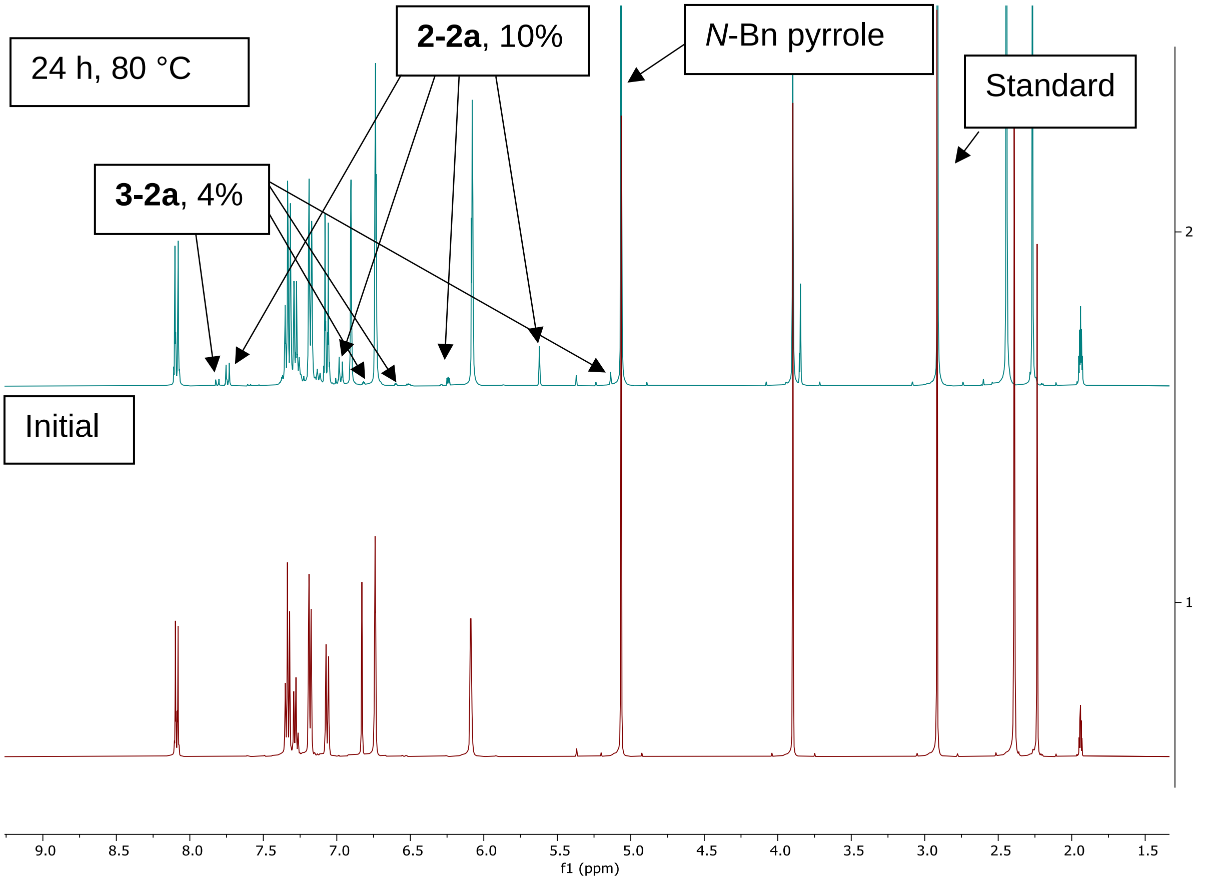

Figure S9. ^{1}H NMR of the reaction of *p*-anisoyl chloride and *N*-benzylpyrrole.

B. The reaction of *N*-acyl pyridinium salt **1c** with *N*-benzylpyrrole.

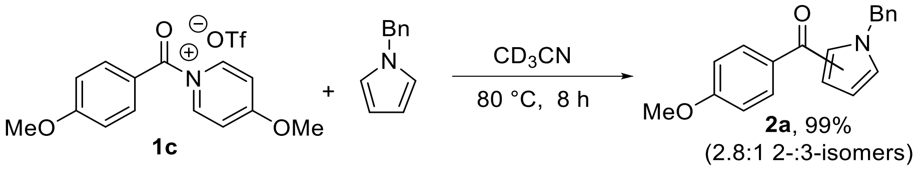

N-Acyl pyridinium salt **1c** was pre-generated as described in Section 2 using 0.6 mL CD_3CN and transferred to a J-Young NMR tube with dimethyl sulfone standard (1.4 mg, 0.015 mmol). An Initial ^1H NMR was obtained, and the NMR tube was brought back into the glove box, followed by the addition of a solution of *N*-benzyl pyrrole (16 mg, 0.10 mmol) in 0.2 mL CD_3CN. The reaction was brought out of the glovebox and monitored by ^1H NMR for 24 h at ambient temperature, after which no formation of **2a** was observed. The reaction was then heated to 80°C for 8 h, after which the yield of **2a,** 99% (2.8:1 ratio of 2-:3-isomers), was determined by ^1H NMR analysis relative to the internal standard.

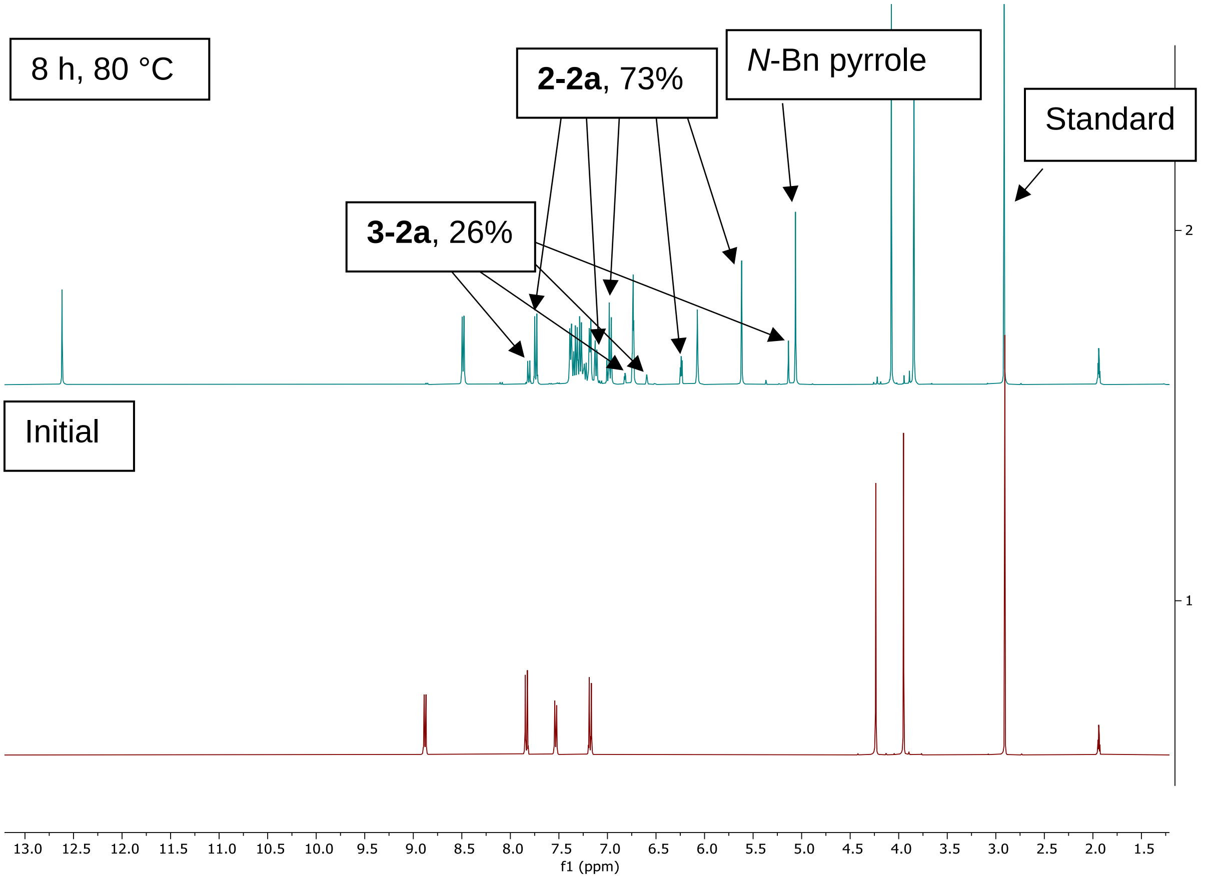

Figure S10. ^1H NMR of the reaction of 1c and *N*-benzylpyrrole.

C. The reaction of **1d** with *N*-benzylpyrrole and thiophene (Figure S4).

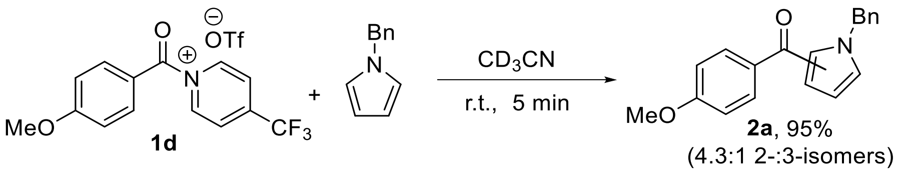

A similar procedure as described above was applied. The reaction was monitored by ^{1}H NMR at ambient temperature immediately after the addition of *N*-benzylpyrrole. The yield of **2a**, 95% (4.3:1 ratio of 2-:3-isomers), was determined by ^{1}H NMR analysis relative to the internal standard.

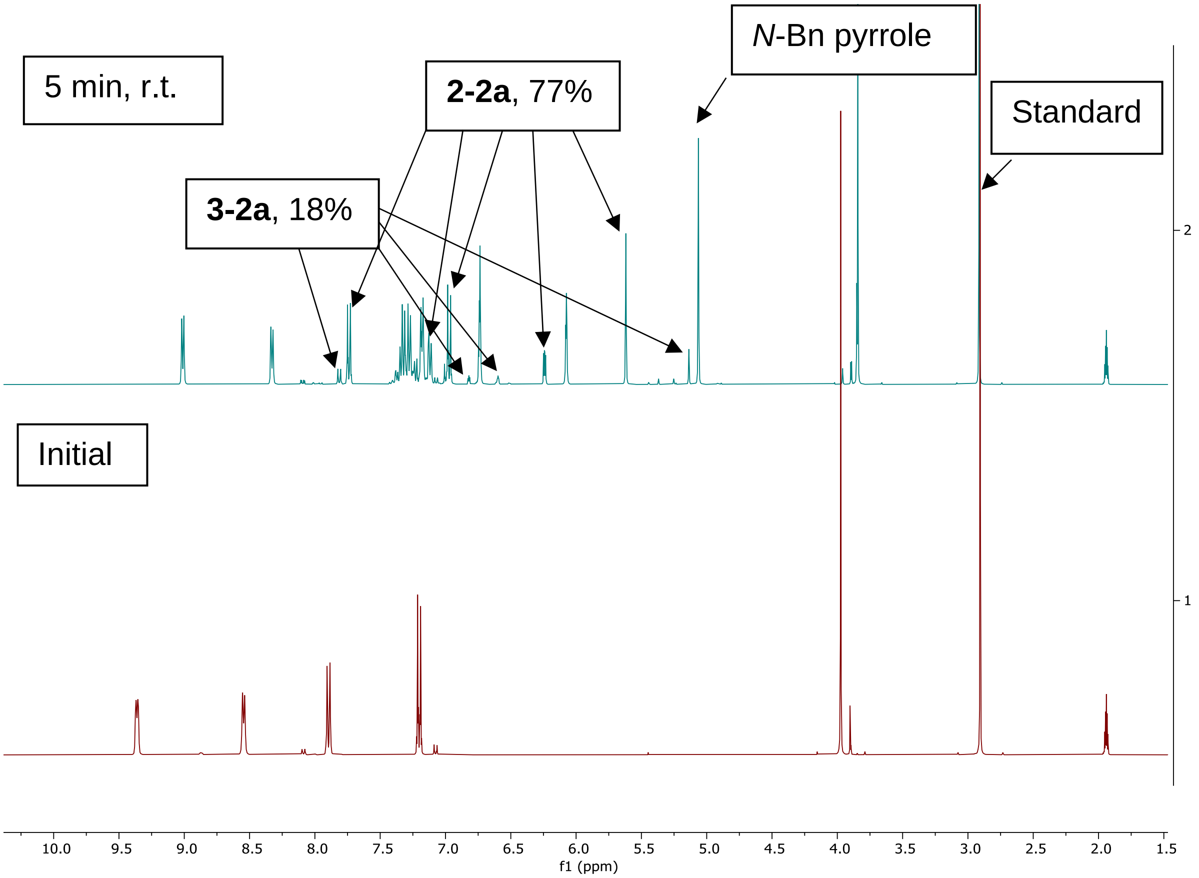

Figure S11. ^{1}H NMR of the reaction of 1d and *N*-benzylpyrrole.

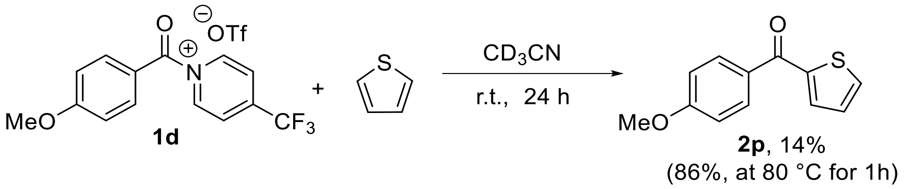

A similar procedure as described above was applied except replacing the pyrrole with thiophene (8.4 mg, 0.10 mmol). The reaction was monitored by ^{1}H NMR at ambient temperature for 24 h, after which the yield of **2p,** 14%, was determined by ^{1}H NMR analysis relative to the internal standard. The NMR tube was then heated to 80 °C for 1 hour to obtain the final yield of 86%.

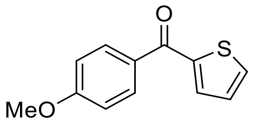

(4-methoxyphenyl)(thiophen-2-yl)methanone (2p).[7] ^{1}H NMR (500 MHz, Acetonitrile-d_3) δ 7.91 – 7.85 (m, 2H), 7.83 (dd, J = 5.0, 1.1 Hz, 1H), 7.68 (dd, J = 3.8, 1.1 Hz, 1H), 7.22 (s, 1H), 7.07 (s, 2H), 3.89 (s, 3H). ^{13}C NMR (126 MHz, Acetonitrile-d_3) δ 187.4, 175.0, 164.2, 144.6, 135.3, 132.4, 131.4, 129.2, 114.7, 56.3

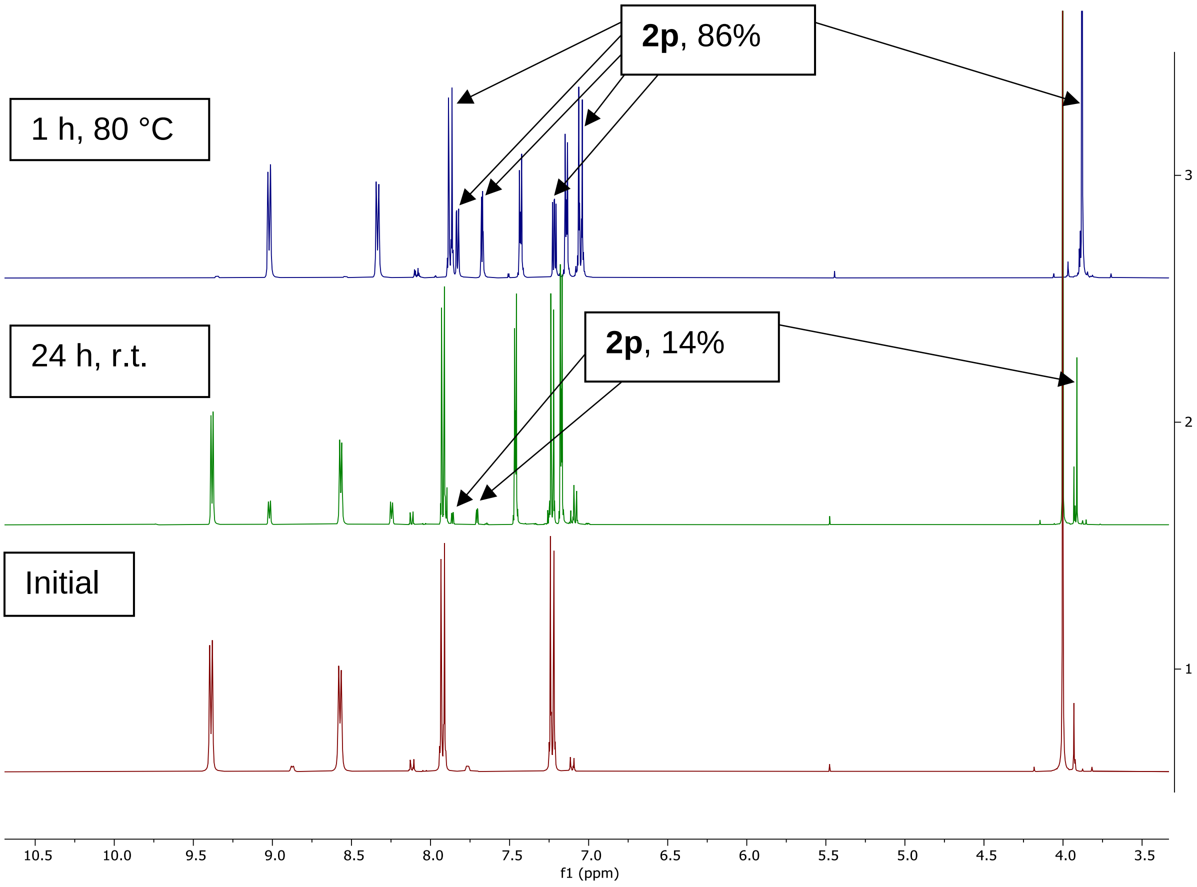

Figure S12. ^{1}H NMR of the reaction of 1d and thiophene.

D. The reaction of *N*-acyl pyridinium salt **1e** with *N*-benzylpyrrole.

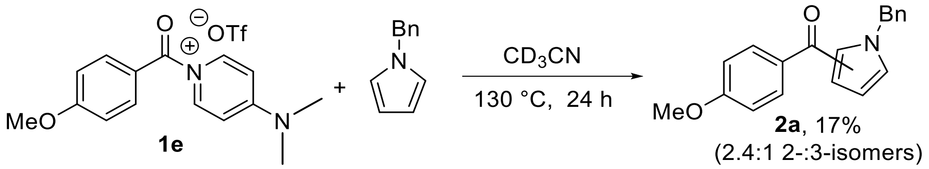

N-Acyl pyridinium salt **1e** was pre-generated as described in Section 2 using 0.6 mL CD_3CN and transferred to a J-Young NMR tube with dimethyl sulfone standard (1.4 mg, 0.015 mmol). An Initial ^1H NMR was obtained, and the NMR tube was brought back into the glove box, followed by the addition of a solution of *N*-benzyl pyrrole (16 mg, 0.10 mmol) in 0.2 mL CD_3CN. The reaction was brought out of the glovebox and monitored by ^1H NMR for 24 h at 80°C, after which no formation of **2a** was observed. The reaction was then heated to 130°C for 24 h, after which the yield of **2a,** 17% (2.4:1 ratio of 2-:3-isomers), was determined by ^1H NMR analysis relative to the internal standard.

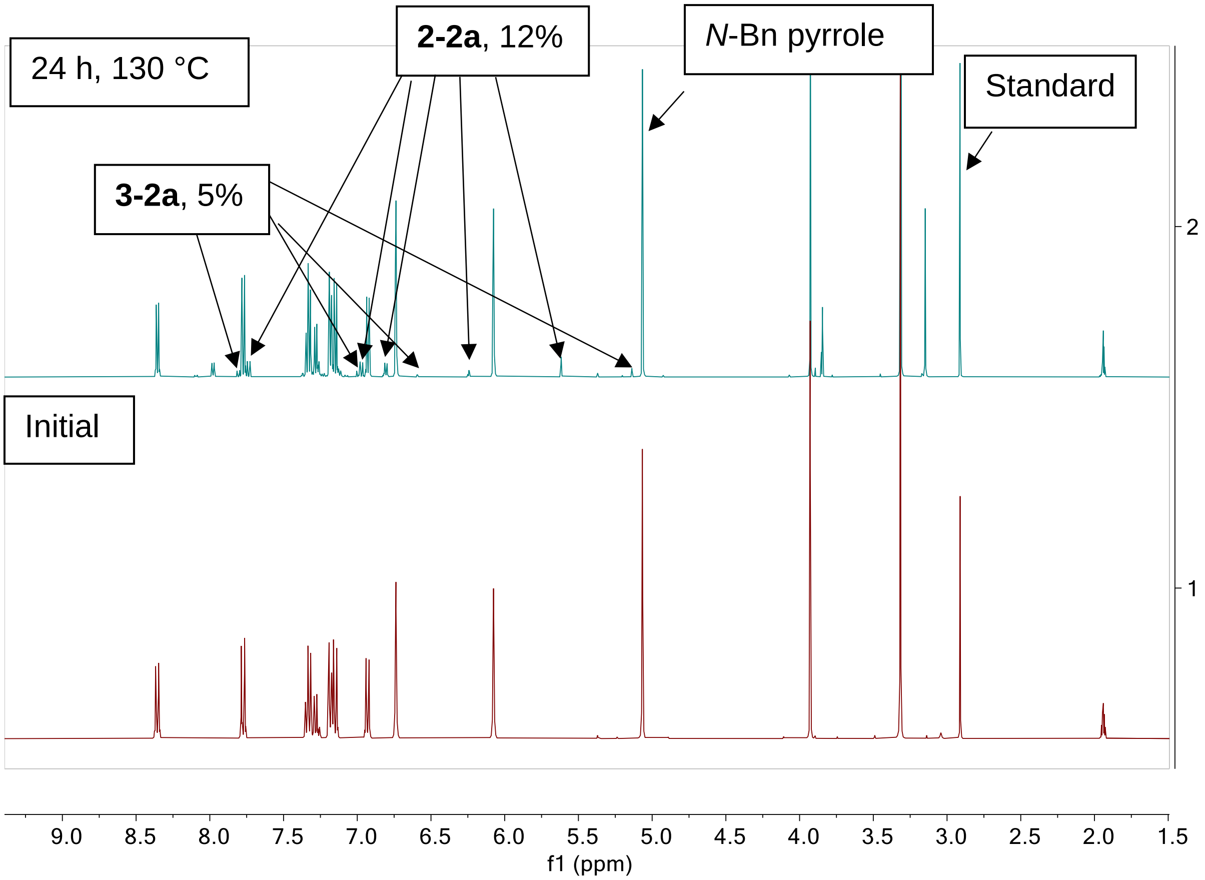

Figure S13. ^1H NMR of the reaction of 1e and *N*-benzylpyrrole.

E. The reaction of *p*-anisoyl triflate with *N*-benzylpyrrole and thiophene.

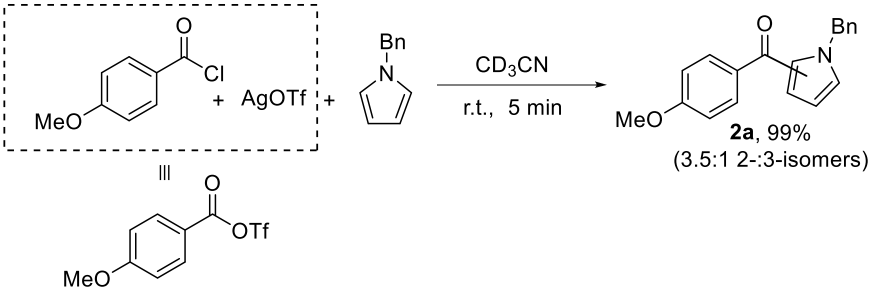

2a, 99%
(3.5:1 2-:3-isomers)

In a glovebox, of *p*-anisoyl chloride (8.5 mg, 0.050 mmol) and dimethyl sulfone standard (1.4 mg, 0.015 mmol) were dissolved in 0.5 mL CD_3CN in a 5 mL vial and transferred to a J-Young NMR tube. The NMR tube was brought out of the glovebox and an initial ^1H NMR spectrum was obtained. The NMR tube was brought back into the glovebox and an *N*-benzyl pyrrole (15.7 mg, 0.10 mmol) solution in 0.15 mL of CD_3CN was added to the NMR tube, followed by the addition of a AgOTf (13.5 mg, 0.053 mmol) solution in 0.15 mL CD_3CN. *Note: aroyl triflate must be generated in the presence of the pyrrole trap; otherwise it decomposes in acetonitrile.* The reaction was immediately monitored by ^1H NMR. Due to the generation of acid, the NMR signals were broadened. To obtain a well resolved spectra, 2,4,6-collidine (12 mg, 0.1 mmol) was added at the end of the reaction. The yield of **2a,** 99% (3.5:1 ratio of 2-:3-isomers), was determined by ^1H NMR analysis relative to the internal standard.

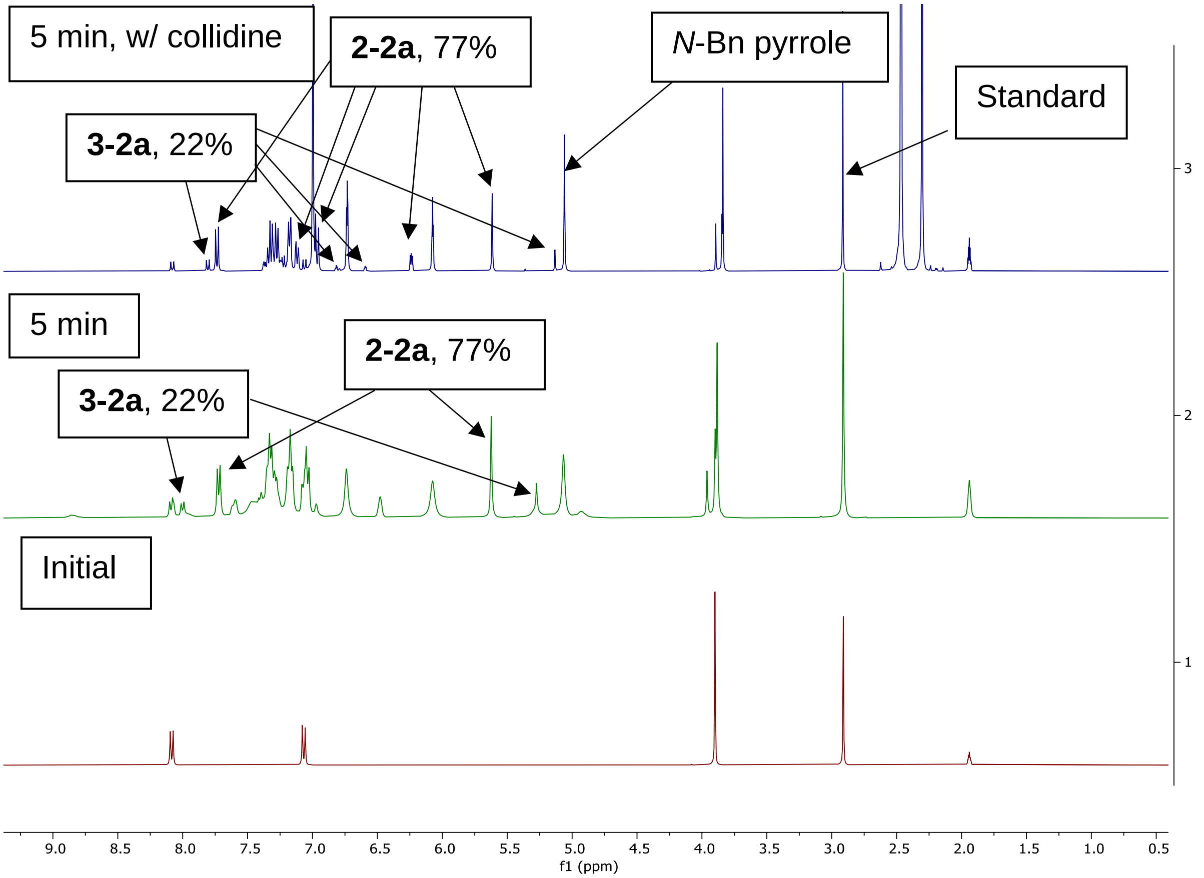

Figure S14. ^{1}H NMR of the reaction of *p*-anisoyl triflate and thiophene.

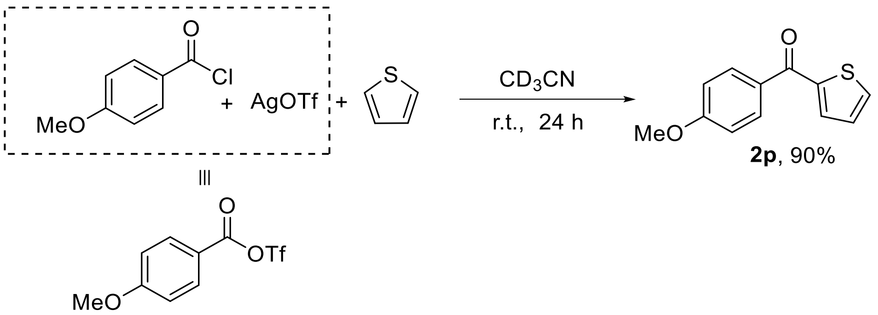

A similar procedure as described above was applied except replacing the pyrrole with thiophene (8.4 mg, 0.10 mmol). The reaction was monitored by ^{1}H NMR at ambient temperature for 24 h, after which the yield of **2p**, 90%, was determined by ^{1}H NMR analysis relative to the internal standard.

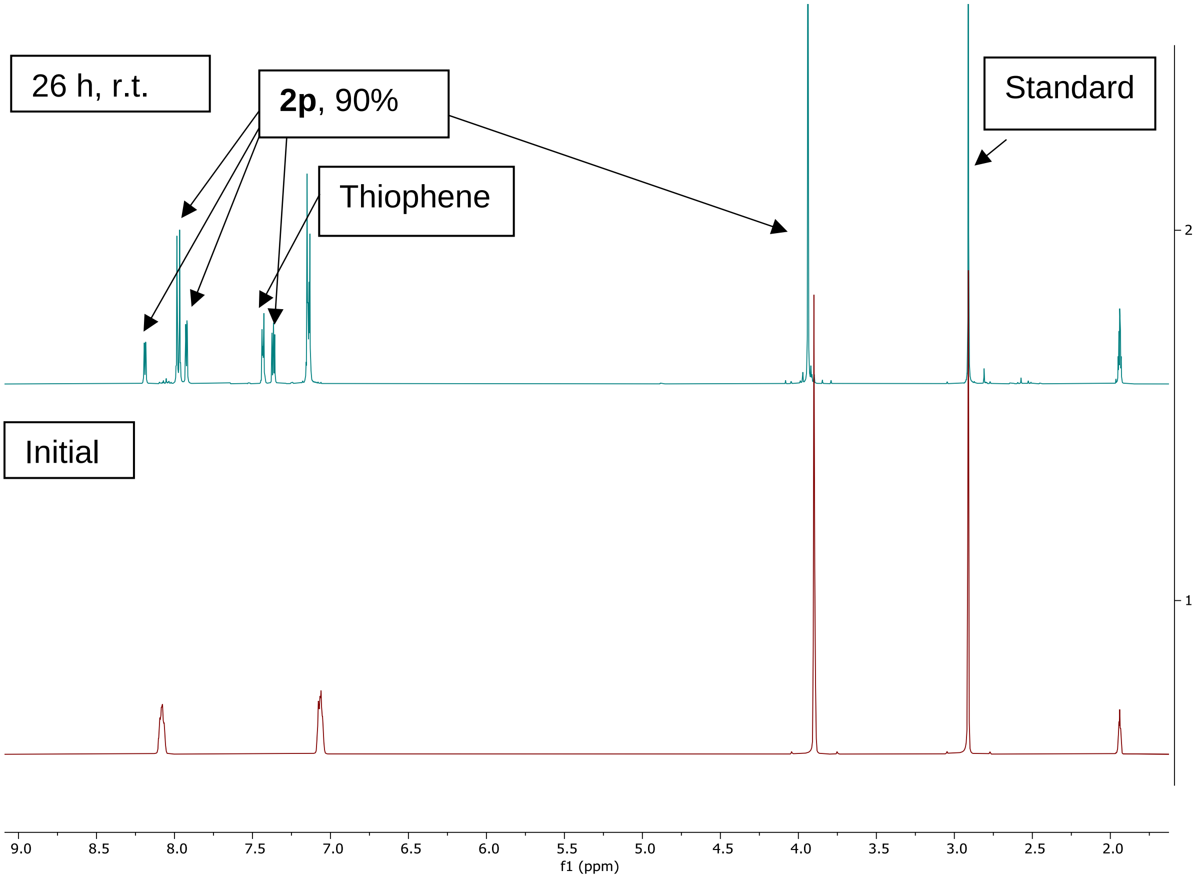

Figure S15. ^{1}H NMR of the reaction of *p*-anisoyl triflate and thiophene.

5. Typical catalytic carbonylation procedures (Figure 4)

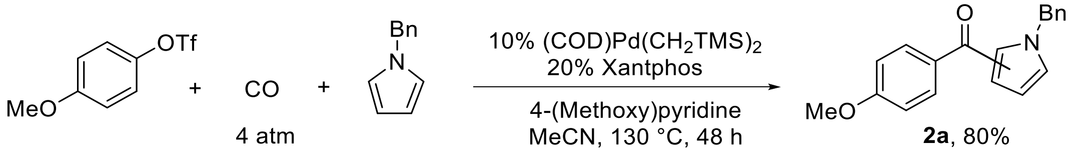

2a, 80%

(1.7: 1 2-:3-isomers)

Reactions with 4-(methoxy)pyridine: In a glovebox, 4-(methoxy)phenyl triflate (128 mg, 0.50 mmol, 2 equiv.), *N*-benzylpyrrole (39 mg, 0.25 mmol, 1 equiv.), 4-(methoxy)pyridine (27 mg, 0.25 mmol, 1 equiv.), (COD)Pd(CH_2TMS)$_2$ (9.7 mg, 0.025 mmol), Xantphos (29 mg, 0.050 mmol), and dimethyl sulfone standard (4.7 mg, 0.05 mmol) were dissolved in 1.8 mL CH_3CN. The mixture was transferred into a thick-walled 25 mL glass reaction vessel equipped with a magnetic stir bar and a Teflon cap. The vessel was closed, taken out of the glovebox and attached to a CO line. Before opening the vessel, the connecting tubing was evacuated and backfilled with carbon monoxide three times, and finally the vessel was opened and pressurized with 4 atm carbon monoxide (on top of 1 atm of nitrogen). The vessel was closed and heated at 130 °C for 48 h with stirring. After the reaction was cooled to room temperature, all volatiles were removed *in vacuo*. The crude

product was purified by column chromatography: Silica gel, gradient hexane (with 1% NEt_3)/ ethyl acetate 5% to 30%, affording pure ketones 2-**2a** (2-isomer of **2a**, 36.2 mg, 0.12 mmol) and **3-2a** (3-isomer of **2a**, 21.7 mg, 0.075 mmol) as yellow oils in 80% overall yield (1.7:1 ratio of 2- to 3-isomers).

Ketones **2b-2o** were obtained using similar procedures described above except with a modification for **2i:** $Pd(Xantphos)_2$ (31.6 mg, 0.025 mmol) was used as the catalyst without added Xantphos.

Ketones **3a-3f** were obtained by a similar procedure with the following modifications. **3a-3c** and **3e** using 5% $Pd_2dba_3{\cdot}CHCl_3$ (12.9 mg, 0.013 mmol) as the catalyst and 2 equiv. 4-(methoxy)pyridine (54.6, 0.50 mmol). **3a**: 20 min; **3b**: 80 ºC, 16 h; **3c**: 150 ºC, 12 min; **3e**: 1 h. **3d**: 0.1 mmol scale (see procedure below) 1.25% $Pd_2dba_3{\cdot}CHCl_3$ (1.29 mg, 0.00125 mmol), 2 equiv. vinyl triflate, 1.1 equiv. 4-(methoxy)pyridine, 100 °C, 5 h; **3f**: 0.1 mmol scale, 2 equiv. vinyl triflate, 2 equiv. 4-(methoxy)pyridine, 25 °C, 2 h;

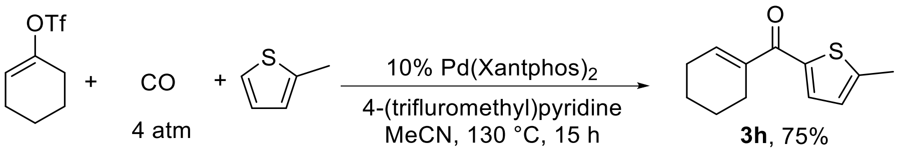

<u>Reactions with 4-(trifluoromethyl)pyridine at 0.25 mmol scale</u>: A similar procedure as described above was conducted, except using 1-cyclohexenyl triflate (57.6 mg, 0.25 mmol, 1 equiv.), 2-methylthiophene (122.7 mg, 1.25 mmol, 5 equiv.), 4-(trifluoromethyl)pyridine (73.6 mg, 0.50 mmol, 2 equiv.), and $Pd(Xantphos)_2$ (31.6 mg, 0.025 mmol). The reaction was heated at 130 °C for 15 h with stirring. After the reaction was cooled to room temperature, all volatiles were removed *in vacuo*. The crude product was purified by column chromatography: Silica gel, gradient hexane/ethyl acetate 5% to 30%, affording pure ketone **3h** as a yellow liquid in 75% yield (38.5 mg, 0.18 mmol).

Ketones **3g**, **3i**, **3k**, **3l**, **3n**, **3t**, **3u-w** were obtained using similar procedure described above with the following modifications: **3g, 3i, 3t, 3v**: 5% $Pd_2dba_3{\cdot}CHCl_3$ (12.9 mg, 0.013 mmol) and 20% Xantphos (28.9 mg, 0.050 mmol), neat arene, 24 h; **3k**: 7 equiv. of heteroarene, 48 h; **3l**: 10 equiv. heteroarene, 24 h; **3n**: 10 equiv. heteroarene, 19 h; **3u**: neat anisole, 20 h; **3w**: $Pd_2dba_3{\cdot}CHCl_3$/Xantphos as the catalyst, 0.3 mmol scale, 24 h.

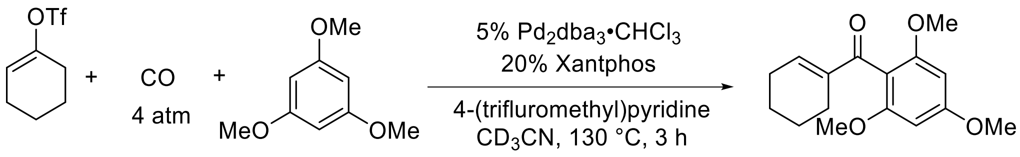

3o, 93%

<u>Reactions with 4-(trifluoromethyl)pyridine at 0.10 mmol scale</u>: In a glovebox, 1-cyclohexenyl triflate (23.0 mg, 0.10 mmol, 1 equiv.), 1,3,5-trimethoxybenzene (84.1 mg, 0.50 mmol, 5 equiv.), 4-(trifluoromethyl)pyridine (29.4 mg, 0.20 mmol, 2 equiv.), $Pd_2dba_3{\cdot}CHCl_3$ (5.2 mg, 0.0050 mmol), Xantphos (11.6 mg, 0.020 mmol), and dimethyl sulfone standard (2.8 mg, 0.03 mmol) were mixed in a 5 mL vial with 0.8 mL CD_3CN. This solution was transferred to a J-Young NMR tube. The tube was closed, taken out of the glovebox, frozen under liquid nitrogen. The headspace was evacuated and then 4 atm CO were condensed into the tube. (As noted in the general procedures, this was accomplished by condensing 120 mTorr of a CO filled vacuum line (67 mL volume) into the NMR tube (headspace 2.2 mL). The reactions were heated at 130 °C for 3 h. *Two* identical reactions were set up at the same time for the purpose of isolation. After the reactions were cooled to room temperature, they were combined, and all volatiles were removed *in vacuo*. The crude product was purified by column chromatography: Silica gel, gradient hexane/ethyl acetate 10% to 30%, affording pure ketones **3o** as a pale-yellow solid in 93% yield (25.8 mg, 0.09 mmol).

Ketones **3j**, **3m** and **3p-s** were obtained using similar procedures as described above with the following modifications: **3j**: 10% $Pd(Xantphos)_2$ (12.6 mg, 0.01 mmol), 80 °C, 10 equiv. heteroarene,18 h; **3m**: 14 equiv. of heteroarene, 100 °C, 8 h; **3p**: 2 h; **3q**: 10 equiv. of heteroarene, 9 h; **3s**, 1 x 0.1 mmol scale.

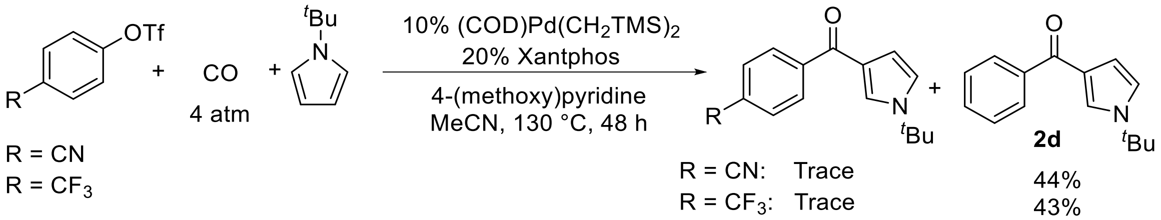

<u>Carbonylations with electron deficient aryl triflates (Figure S5):</u> reactions were conducted using similar procedures as described above. The crude mixture was purified by column chromatography: Silica gel, gradient hexane (with 1% NEt_3)/ethyl acetate 5% to 30%, affording pure ketone **2d** as a yellow oil in 44% (25.0 mg, 0.11 mmol/0.25 mmol) and 43% (24.6 mg, 0.11 mmol/0.25 mmol) yield, when 4-(cyano)phenyl triflate and 4-(trifluoromethyl)phenyl triflate were used, respectively.

6. Selectivity control in carbonylative C-H functionalization (Figure 5)

A. Regioselectivity of C-H functionalization with *N-tert*-butyl pyrrole (Figure 5a)

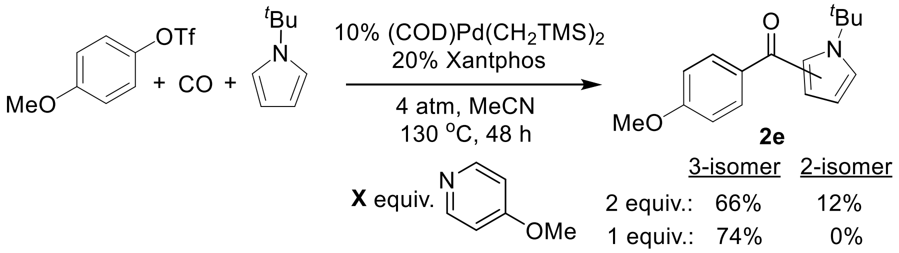

	3-isomer	2-isomer
2 equiv.:	66%	12%
1 equiv.:	74%	0%

Similar procedures as described above (section 5, procedure A) were applied.

For the reaction with 2 equiv. of 4-(methoxy)pyridine, ketone **2-2e** (2-isomer of **2e**) was isolated in 12% yield (7.7 mg, 0.030 mmol) and **3-2e** (3-isomer of **2e**) was isolated in 66% yield (42.3 mg, 0.16 mmol).

For the reaction with 1 equiv. of 4-(methoxy)pyridine, ketone **3-2e** (3-isomer of **2e**) was isolated exclusively in 74% yield (47.6 mg, 0.18 mmol).

B. Chemoselectivity of C-H functionalization with (hetero)arenes (Figure 5b)

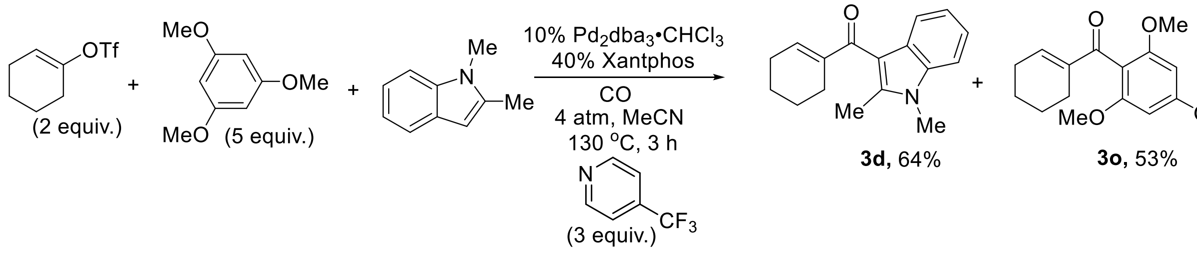

3d, 64% **3o,** 53%

In a glovebox, 1-cyclohexenyl triflate (115.1 mg, 0.50 mmol), 1,*2*-dimethyl indole (36.3 mg, 0.25 mmol), 1,3,5-trimethoxybenzene (210.2 mg, 1.25 mmol), 4-(trifluoromethyl)pyridine (110.3 mg, 0.75 mmol), $Pd_2dba_3{\cdot}CHCl_3$ (25.9 mg, 0.025 mmol), and Xantphos (57.9 mg, 0.10 mmol) were dissolved in 3.0 mL MeCN. The mixture was transferred into a thick-walled 25 mL glass reaction vessel equipped with a magnetic stir bar and a Teflon cap. The vessel was taken out of the glovebox and attached to a Schlenk line. Before opening the vessel, the connecting tubing was evacuated and backfilled with carbon monoxide three times, and finally the vessel was opened and pressurized with 4 atm carbon monoxide. The reaction was heated at 130 °C for 3 h with stirring. After the reaction was cooled to room temperature, all volatiles were removed *in vacuo*. The crude product was purified by column chromatography: Silica gel, gradient hexane/ethyl acetate 5% to 30%. Ketone **3d** was isolated in 64% yield (40.8 mg, 0.16 mmol). Ketone **3o** was isolated in 53% yield (36.8 mg, 0.13 mmol).

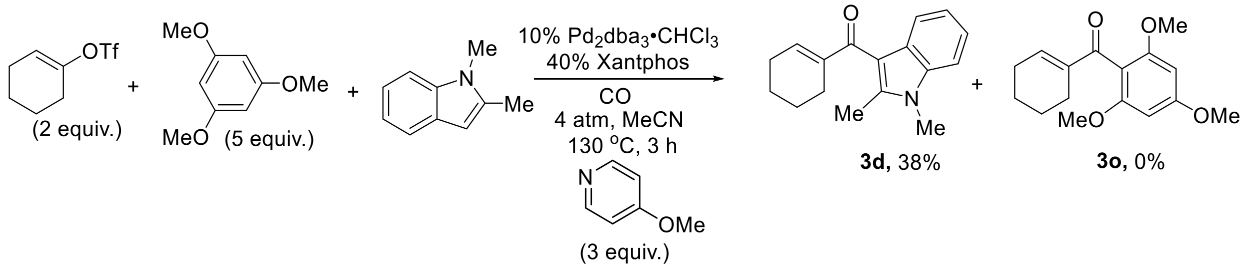

A similar procedure as described above was applied except replacing 4-(trifluoromethyl)pyridine with 4-(methoxy)pyridine (81.8 mg, 0.75 mmol). Ketone **3d** was isolated in 38% yield (24.2 mg, 0.10 mmol).

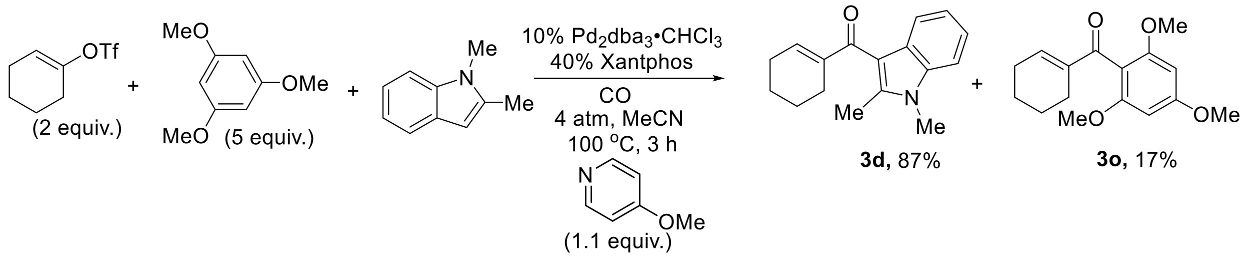

In a glovebox, 1-cyclohexenyl triflate (23.0 mg, 0.10 mmol), 1,*2*-dimethyl indole (7.3 mg, 0.05 mmol), 1,3,5-trimethoxybenzene (42.0 mg, 0.25 mmol), 4-(methoxy)pyridine (6.0 mg, 0.055 mmol), $Pd_2dba_3 \cdot CHCl_3$ (5.2 mg, 0.005 mmol) Xantphos (11.6 mg, 0.02 mmol) and dimethyl sulfone standard (2.8 mg, 0.03 mmol) were mixed in a 5 mL vial with 0.8 mL CD_3CN. This solution was transferred into a J-Young NMR tube. The tube was taken out of the glovebox, frozen under liquid nitrogen. The headspace was evacuated and then 4 atm CO were condensed into the tube. (As noted in the general procedures, this was accomplished by condensing 120 mTorr of a CO filled vacuum line (67 mL volume) into the NMR tube (headspace 2.2 mL). The reaction was heated at 100 °C for 2.5 h. The yield of ketone **3d,** 87%, and ketone **3o,** 17% were determined by ^1H NMR analysis relative to dimethyl sulfone standard.

C. Double C-H functionalization of heteroarenes (Figure 5c)

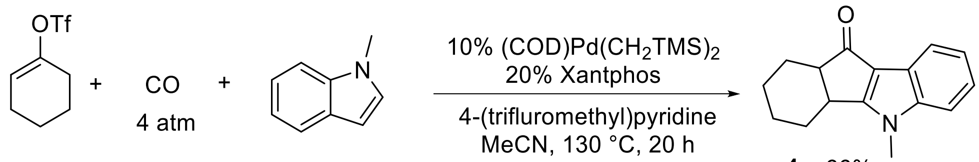

4a, 66%

In a glovebox, 1-cyclohexenyl triflate (115.1 mg, 0.50 mmol), 1-methyl indole (32.8 mg, 0.25 mmol), 4-(trifluoromethyl)pyridine (73.6 mg, 0.50 mmol), $(COD)Pd(CH_2TMS)_2$ (9.7 mg, 0.025 mmol), Xantphos (28.9 mg, 0.050 mmol), and dimethyl sulfone standard (4.7 mg, 0.05 mmol) were dissolved in 1.8 mL MeCN. The mixture was transferred into a thick-walled 25 mL glass reaction vessel equipped with a magnetic stir bar and a Teflon cap. The vessel was taken out of the glovebox and attached to a Schlenk line. Before opening the vessel, the connecting tubing was evacuated and backfilled with carbon monoxide

three times, and finally the vessel was opened and pressurized with 4 atm carbon monoxide. The reaction was heated at 130 °C for 20 h. After the reaction was cooled to room temperature, all volatiles were removed *in vacuo*. The crude product was purified by column chromatography: Silica gel, gradient hexane (with 1% NEt_3)/ ethyl acetate 5% to 30%, affording pure ketone **4a** as a pale-yellow solid in 66% yield (39.5 mg, 0.16 mmol).

Ketones **4b** and **4c** were obtained using similar procedures as described above with the following modifications: **4b**: 4 h; **4c**: 1-cyclohexenyl triflate as the limiting reagent, 5 equiv. of 2-methyl thiophene, 48 h.

7. Synthesis of aryl triflates

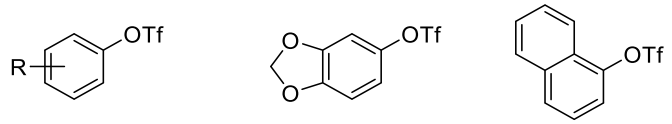

5a: R = 4-MeO **5d**: R = 4-Ph **5g** **5h**
5b: R = 4-CN **5e**: R = 4-^{t}Bu
5c: R = 4-CF_3 **5f**: R = 3-^{t}Bu

Aryl triflates were synthesized from the corresponding phenols using known literature procedures.[5] Compounds **5a**,[8] **5b**,[8] **5c**,[9] **5d**,[10] **5e**,[11] and **5h**[8] are known molecules and the characterization data of each molecule matches that found in the literature.

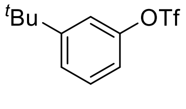

3-(*tert*-butyl)phenyl trifluoromethanesulfonate (5f). ^{1}H NMR (500 MHz, Chloroform-*d*) δ 7.45 – 7.37 (m, 2H), 7.28 – 7.26 (m, 1H), 7.11 (ddd, *J* = 7.9, 2.5, 1.2 Hz, 1H), 1.35 (s, 9H). ^{13}C NMR (126 MHz, Chloroform-*d*) δ 154.6, 149.9, 129.9, 125.5, 118.9 (q, *J* = 320.8 Hz), 118.6, 118.3, 35.2, 31.2. ^{19}F NMR (471 MHz, Chloroform-*d*) δ -72.95. HRMS. Calculated for $C_{11}H_{13}F_3O_3S^+$ (M^+): 282.0532, found: 282.0540.

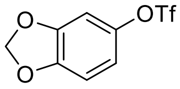

benzo[*d*][1,3]dioxol-5-yl trifluoromethanesulfonate (5g). ^{1}H NMR (500 MHz, Chloroform-*d*) δ 6.80 (d, *J* = 8.4 Hz, 1H), 6.78 – 6.73 (m, 2H), 6.05 (s, 2H). ^{13}C NMR (126 MHz, Chloroform-*d*) δ 148.7, 147.6, 143.6, 118.9 (q, *J* = 320.9 Hz), 114.6, 108.4, 103.5, 102.6. ^{19}F NMR (471 MHz, Chloroform-*d*) δ -72.72. HRMS. Calculated for $C_8H_5F_3O_5S^+$ (M^+): 269.9804, found: 269.9813.

8. Synthesis of vinyl triflates

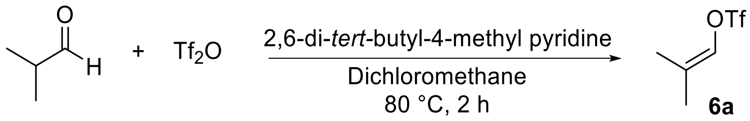

The following procedure was adapted from the literature.[4a] Under argon, isobutyraldehyde (1.08 g, 15 mmol), 2,6-di-*tert*-butyl-4-methylpyridine (3.24 g, 15.8 mmol), and 30 mL of dichloromethane were added to a 100 mL thick-walled glass reaction vessel equipped with a magnetic stir bar and a Teflon cap. Trifluoromethanesulfonic anhydride (2.6 mL, 15.8 mmol) was added via syringe over the course of 1 minute. The vessel was sealed and heated at 80 °C for 2 h. After the mixture was cooled to room temperature, and the dichloromethane was carefully removed *in vacuo*. [Care must be taken to prevent the loss of the product *in vacuo*, thus 250 mbar vacuum was applied at room temperature.] 50 mL of pentane was added to obtain a suspension. The solid was removed by vacuum filtration and washed with 30 mL × 2 pentane. The solution containing **7a** was combined, and the volitiles were carefully removed *in vacuo*. (similar care to that noted above) The crude product was purified by vacuum distillation (60 °C, 50 mTorr static vacuum). Vinyl triflate **6a** was obtained as a colorless liquid in 42% yield (1.28 g, 6.3 mmol, low isolated yield due to loss in distillation).

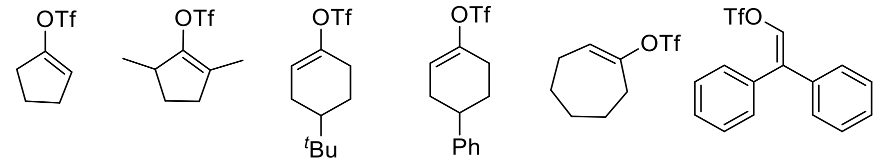

6b **6c** **6d** **6e** **6f** **6g**

Vinyl triflates **6b-h** were obtained using a similar procedure as described above with the following modifications: **6b**: reaction heated for 4 h, product obtained by distillation at 80 °C, 50 mTorr static vacuum. **6c**: reaction heated for 48 h, product obtained by distillation at 80 °C, 50 mTorr. **6d**: reaction heated for 4 h, product obtained by distillation at 80 °C, 50 mTorr. **6e**: reaction heated for 4 h, product obtained by distillation at 110 °C, 50 mTorr. **6f**: reaction heated for 4 h, product obtained by distillation at 80 °C, 50 mTorr. **6g**: obtained by distillation at 120 °C, 50 mTorr.

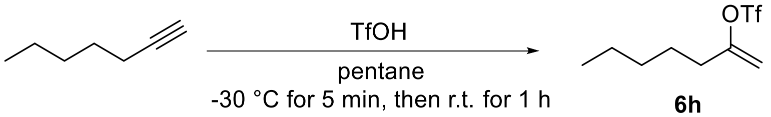

6h

The following procedure was adapted from the literature.[4b] A solution of alkyne (1.10 g, 11.4 mmol) in pentane (20 mL) was prepared in a 50 mL round-bottom flask equipped with a magnetic stir bar. The flask was purged with argon for 5 minutes and then cooled to -30 °C. Under argon, trifluoromethanesulfonic acid (0.5 mL, 5.7 mmol) was added dropwise over the course of 5 minutes via syringe to the solution. The reaction mixture was warmed to 0 °C and stirred for an additional 1 h. The reaction was quenched with 5 mL saturated $NaHCO_3$. The organic layer was separated, washed with saturated $NaHCO_3$ 2×10 mL and dried over anhydrous Na_2SO_4. The solution was concentrated, and the crude product was further purified by vacuum distillation (80 °C, 50 mTorr static vacuum). Vinyl triflate **6h** was obtained as a colorless liquid in 32% yield (0.91 g, 3.7 mmol, low isolated yield due to loss in distillation).

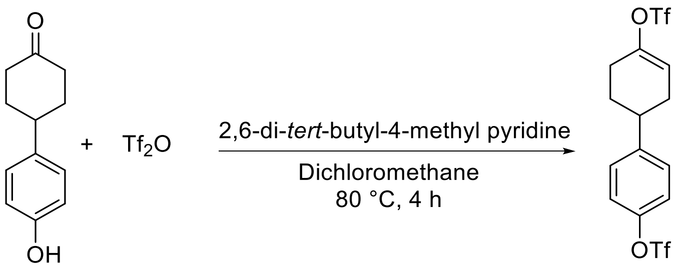

6i

Under argon, 4-(4-hydroxyphenyl)cyclohexanone (0.49 g, 2.6 mmol), 2,6-di-*tert*-butyl-4-methylpyridine (1.17 g, 5.7 mmol) and 15 mL of dichloromethane were added to a 100 mL thick-walled glass reaction vessel equipped with a magnetic stir bar and a Teflon cap. Trifluoromethanesulfonic anhydride (1.0 mL, 5.7 mmol) was added via syringe over the course of 1 minute. The vessel was sealed and heated at 80 °C for 4 h with stirring. After the mixture was cooled to room temperature, the volatiles were carefully removed *in vacuo*. 50 mL of pentane was added to obtain a suspension. The solid was removed by vacuum filtration and washed with 30 mL × 2 pentane. The solutions were combined, and the volatiles were removed *in vacuo* to afford vinyl triflate **6i** as a beige solid in 95% yield (1.20 g, 2.6 mmol).

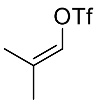

2-methylprop-1-en-1-yl trifluoromethanesulfonate (6a). Spectral data matches with literature.[12] Isolated yield 42% (1.28 g, 6.3 mmol, 99% NMR yield, low isolated yield due to loss in distillation). Colorless liquid. ^{1}H NMR (500 MHz, $CDCl_3$) δ 6.41 – 6.36 (m, 1H), 1.76 (s, 3H), 1.70 (s, 3H). ^{13}C NMR (126 MHz, $CDCl_3$) δ 130.4, 126.4, 118.6 (q, *J* = 320.7 Hz), 19.0, 15.6. ^{19}F NMR (471 MHz, $CDCl_3$) δ -74.02.

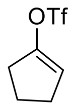

Cyclopent-1-en-1-yl trifluoromethanesulfonate (6b). Spectral data matches with literature.[13] Isolated yield 48% (1.6 g, 7.2 mmol). Colorless liquid. ^{1}H NMR (500 MHz, $CDCl_3$) δ 5.63 (p, *J* = 2.3 Hz, 1H), 2.57 (dddd, *J* = 10.5, 5.4, 4.1, 2.2 Hz, 2H), 2.41 (ddt, *J* = 10.4, 5.6, 2.8 Hz, 2H), 2.03 (tt, *J* = 8.6, 6.7 Hz, 2H). ^{13}C NMR (126 MHz, $CDCl_3$) δ 149.6, δ 118.6 (q, *J* = 320.5 Hz), 117.8, 30.8, 28.0, 20.8. ^{19}F NMR (471 MHz, $CDCl_3$) δ -73.63.

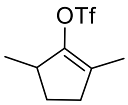

2,5-dimethylcyclopent-1-en-1-yl trifluoromethanesulfonate (6c). Spectral data matches with literature.[14] Isolated yield 64% (2.3 g, 9.6 mmol). Colorless liquid. ^{1}H NMR (500 MHz, $CDCl_3$) δ 2.96 (ddtt, *J* = 13.4, 9.0, 4.6, 2.3 Hz, 1H), 2.30 (ddt, *J* = 8.1, 6.9, 1.2 Hz, 2H), 2.26 – 2.16 (m, 1H), 1.76 – 1.69 (m, 3H), 1.56 – 1.47 (m, 1H), 1.11 (d, *J* = 6.9 Hz, 3H). ^{13}C NMR (126 MHz, $CDCl_3$) δ 146.8, 128.2, 118.5 (q, *J* = 320.0 Hz), 37.7, 31.5, 28.7, 18.4, 12.4. ^{19}F NMR (471 MHz, $CDCl_3$) δ -74.51.

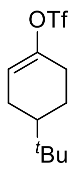

4-(tert-butyl)cyclohex-1-en-1-yl trifluoromethanesulfonate (6d). Spectral data matches with literature.[13,15] Isolated yield 64% (2.8 g, 9.6 mmol). Colorless liquid ^{1}H NMR (500 MHz, $CDCl_3$) δ 5.74 (dt, *J* = 5.0, 2.2 Hz, 1H), 2.44 – 2.29 (m, 2H), 2.24 – 2.16 (m, 1H), 2.00 – 1.90 (m, 2H), 1.45 – 1.27 (m, 2H), 0.89 (s, 9H).

^{13}C NMR (126 MHz, $CDCl_3$) δ 149.2, 118.6 (q, J = 320.1 Hz), 118.4, 42.9, 32.1, 28.6, 27.2, 25.3, 24.1. ^{19}F NMR (471 MHz, $CDCl_3$) δ -74.08.

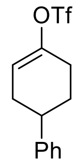

1,2,3,6-tetrahydro-[1,1'-biphenyl]-4-yl trifluoromethanesulfonate (6e). Spectral data matches with literature.[16] Isolated yield 69% (3.2 g, 10.4 mmol). Colorless oil. ^{1}H NMR (500 MHz, $CDCl_3$) δ 7.34 (t, J = 7.6 Hz, 2H), 7.27 – 7.21 (m, 2H), 5.87 (dt, J = 5.2, 2.4 Hz, 1H), 2.93 – 2.78 (m, 1H), 2.62 – 2.29 (m, 4H), 2.16 – 2.04 (m, 1H), 2.04 – 1.89 (m, 1H). ^{13}C NMR (126 MHz, $CDCl_3$) δ 149.0, 144.6, 128.6, 126.8, 126.7, 118.6 (q, J = 320.1 Hz), 118.1, 38.8, 31.6, 29.7, 27.9. ^{19}F NMR (471 MHz, $CDCl_3$) δ -73.99.

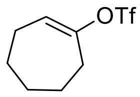

Cyclohept-1-en-1-yl trifluoromethanesulfonate (6f). Spectral data matches with literature.[13,15] Isolated yield 63% (2.3 g, 9.4 mmol). Colorless oil. ^{1}H NMR (500 MHz, $CDCl_3$) δ 5.88 (t, J = 6.5 Hz, 1H), 2.58 – 2.46 (m, 2H), 2.21 – 2.11 (m, 2H), 1.70 (tdd, J = 13.0, 5.0, 2.2 Hz, 4H), 1.66 – 1.60 (m, 2H). ^{13}C NMR (126 MHz, $CDCl_3$) δ 153.1, 123.1, 118.6 (q, J = 320.2 Hz), 33.2, 29.9, 26.3, 24.8, 24.7. ^{19}F NMR (471 MHz, $CDCl_3$) δ -73.94.

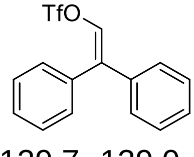

2,2-diphenylvinyl trifluoromethanesulfonate (6g). Spectral data matches with literature.[16] Isolated yield 71% (3.5 g, 10.7 mmol). Yellow oil. ^{1}H NMR (400 MHz, $CDCl_3$) δ 7.42 – 7.34 (m, 5H), 7.32 – 7.21 (m, 5H), 7.05 (s, 1H). ^{13}C NMR (101 MHz, $CDCl_3$) δ 136.5, 134.2, 134.2, 131.9, 129.7, 129.0, 128.8, 128.7, 128.4, 128.4, 118.6 (q, J = 321.0 Hz). ^{19}F NMR (377 MHz, $CDCl_3$) δ -73.74.

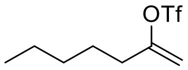

Hept-1-en-2-yl trifluoromethanesulfonate (6h). Spectral data matches with literature.[4b] Isolated yield 32% (0.91 g, 3.7 mmol). Colorless liquid. ^{1}H NMR (500 MHz, $CDCl_3$) δ 5.09 (d, J = 3.5 Hz, 1H), 4.92 (d, J = 4.6 Hz, 1H), 2.33 (t, J = 7.9 Hz, 2H), 1.59 – 1.50 (m, 2H), 1.36 – 1.30 (m, 4H), 0.94 – 0.88 (m, 3H). ^{13}C NMR (126 MHz, $CDCl_3$) δ 157.1, 118.5 (q, J = 319.9 Hz), 104.0, 33.8, 30.8, 25.6, 22.2, 13.9. ^{19}F NMR (471 MHz, $CDCl_3$) δ -74.15.

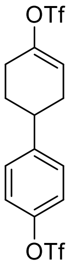

1,2,3,6-tetrahydro-[1,1'-biphenyl]-4,4'-diyl bis(trifluoromethanesulfonate (6i). Isolated yield 95% (1.2 g, 2.6 mmol). Beige solid. ^{1}H NMR (500 MHz, $CDCl_3$) δ 7.30 (d, J = 8.8 Hz, 2H), 7.23 (d, J = 8.7 Hz, 2H), 5.92 – 5.81 (m, 1H), 2.98 – 2.86 (m, 1H), 2.61 – 2.26 (m, 4H), 2.15 – 2.04 (m, 1H), 1.95 (ddd, J = 23.6, 12.3, 5.7 Hz, 1H). ^{13}C NMR (126 MHz, $CDCl_3$) δ 148.8, 148.2, 145.0, 128.6, 121.5, 118.8 (q, J = 320.6 Hz), 118.5 (q, J = 320.0 Hz), 117.7, 38.2, 31.4, 29.5, 27.6. ^{19}F NMR (471 MHz, $CDCl_3$) δ -72.86, -73.94. HRMS. Calculated for $C_{14}H_{12}F_6O_6S_2Na^+$ (M+Na^+): 476.9872, found: 476.9862.

IV. Characterization Data for Ketones

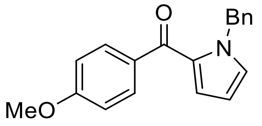

(1-benzyl-1H-pyrrol-2-yl)(4-methoxyphenyl)methanone (2-2a). Spectral data identical to reported compound.[17] Isolated yield 50% (36.2 mg, 0.12 mmol/0.25 mmol). Yellow oil. ^{1}H NMR (500 MHz, Acetonitrile-d_3) δ 7.74 (d, J = 8.9 Hz, 2H), 7.33 – 7.26 (m, 2H), 7.22 (d, J = 14.7 Hz, 1H), 7.18 (dd, J = 2.5, 1.8 Hz, 1H), 7.16 – 7.10 (m, 2H), 6.97 (d, J = 8.9 Hz, 2H), 6.74 (dd, J = 4.0, 1.7 Hz, 1H), 6.24 (dd, J = 4.0, 2.6 Hz, 1H), 5.62 (s, 2H), 3.85 (s, 3H). ^{13}C NMR (126 MHz, Acetonitrile-d_3) δ 185.6, 163.6, 140.3, 133.1, 132.2, 131.8, 130.9, 129.4, 128.2, 127.7, 123.1, 114.3, 109.2, 56.2, 52.6.

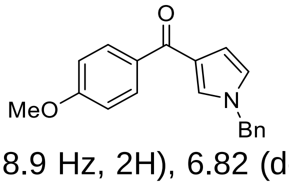

(1-benzyl-1H-pyrrol-3-yl)(4-methoxyphenyl)methanone (3-2a). Isolated yield 30% (21.5 mg, 0.074 mmol/0.25 mmol). Yellow oil. ^{1}H NMR (500 MHz, Acetonitrile-d3) δ 7.81 (d, J = 8.9 Hz, 2H), 7.39 – 7.35 (m, 3H), 7.33 – 7.30 (m, 1H), 7.27 – 7.25 (m, 2H), 7.00 (d, J = 8.9 Hz, 2H), 6.82 (dd, J = 2.8, 2.2 Hz, 1H), 6.59 (dd, J = 2.9, 1.7 Hz, 1H), 5.14 (s, 2H), 3.86 (s, 3H). ^{13}C NMR (126 MHz, Acetonitrile-d3) δ 189.4, 163.3, 138.8, 133.5, 131.8, 129.8, 128.9, 128.9, 128.4, 125.3, 123.6, 114.4, 111.4, 56.1, 54.1. HRMS. Calculated for $C_{19}H_{17}NO_2Na^+$ (M+Na$^+$): 314.1151, found: 314.1146.

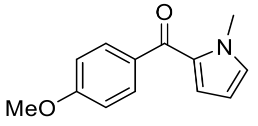

(4-methoxyphenyl)(1-methyl-1H-pyrrol-2-yl)methanone (2-2b). Spectral data identical to reported compound.[17] Isolated yield 56% (30.4 mg, 0.14 mmol/0.25 mmol). Yellow oil. ^{1}H NMR (500 MHz, Chloroform-d) δ 7.83 (d, J = 8.8 Hz, 2H), 6.95 (d, J = 8.8 Hz, 2H), 6.89 (t, J = 2.1 Hz, 1H), 6.72 (dd, J = 4.0, 1.7 Hz, 1H), 6.15 (dd, J = 4.1, 2.5 Hz, 1H), 4.01 (s, 3H), 3.87 (s, 3H). ^{13}C NMR (126 MHz, Chloroform-d) δ 185.3, 162.6, 132.6, 131.6, 131.0, 130.8, 122.0, 113.4, 108.0, 55.6, 37.3.

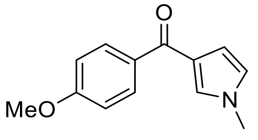

(4-methoxyphenyl)(1-methyl-1H-pyrrol-3-yl)methanone (3-2b). Isolated yield 28% (15.3 mg, 0.071 mmol/0.25 mmol). Brown oil. ^{1}H NMR (500 MHz, Chloroform-d) δ 7.85 (d, J = 8.9 Hz, 2H), 7.19 (t, J = 1.9 Hz, 1H), 6.95 (d, J = 8.9 Hz, 2H), 6.66 – 6.62 (m, 2H), 3.87 (s, 3H), 3.71 (s, 3H). ^{13}C NMR (126 MHz, Chloroform-d) δ 189.5, 162.4, 132.8, 131.3, 128.5, 124.9, 123.1, 113.5, 111.3, 55.5, 36.7. HRMS. Calculated for $C_{13}H_{13}NO_2Na^+$ (M+Na$^+$): 238.0838, found: 238.0827.

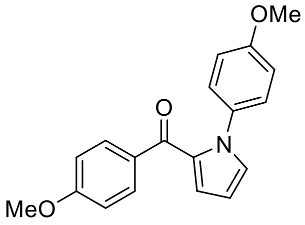

(4-methoxyphenyl)(1-(4-methoxyphenyl)-1H-pyrrol-2-yl) methanone (2-2c). Isolated yield 49% (37.6 mg, 0.12 mmol/0.25 mmol). Brown solid ^{1}H NMR (400 MHz, Chloroform-d) δ 7.90 (d, J = 8.8 Hz, 2H), 7.23 (d, J = 8.9 Hz, 2H), 7.06 – 7.05 (m, 1H), 6.95 – 6.90 (m, 4H), 6.84 (dd, J = 3.9, 1.6 Hz, 1H), 6.31 (dd, J = 4.0, 2.6 Hz, 1H), 3.88 (s, 3H), 3.83 (s, 3H). ^{13}C NMR (126 MHz, Chloroform-d) δ 184.0, 162.9, 158.8, 133.9, 131.9, 131.9, 131.4, 130.7, 126.7, 122.2, 114.2, 113.5, 109.0, 55.6, 55.6. HRMS. Calculated for $C_{19}H_{17}NO_3Na^+$ (M+Na$^+$): 330.1101, found: 330.1106.

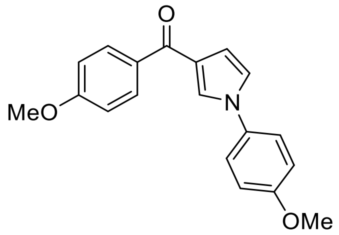

(4-methoxyphenyl)(1-(4-methoxyphenyl)-1H-pyrrol-3-yl)methanone (3-2c). Isolated yield 27% (21.0 mg, 0.068 mmol/0.25 mmol). Yellow oil. ^{1}H NMR (500 MHz, Chloroform-*d*) δ 7.91 (d, *J* = 8.9 Hz, 2H), 7.54 – 7.53 (m, 1H), 7.34 (d, *J* = 9.0 Hz, 2H), 7.01 (dd, *J* = 2.9, 2.2 Hz, 1H), 6.98 – 6.95 (m, 4H), 6.82 (dd, *J* = 3.0, 1.7 Hz, 1H), 3.87 (s, 3H), 3.84 (s, 3H). ^{13}C NMR (126 MHz, Chloroform-*d*) δ 189.6, 162.6, 158.7, 133.5, 132.6, 131.3, 126.0, 126.0, 122.8, 121.4, 114.9, 113.6, 112.2, 55.7, 55.5. HRMS. Calculated for $C_{19}H_{17}NO_3Na^+$ (M+Na$^+$): 330.1101, found: 330.1102.

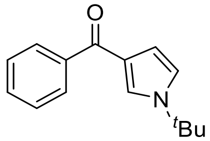

(1-(tert-butyl)-1H-pyrrol-3-yl)(phenyl)methanone (2d). Spectral data identical to reported compound.[18] Isolated yield 63% (35.8 mg, 0.16 mmol/0.25 mmol). Brown oil. ^{1}H NMR (500 MHz, Chloroform-*d*) δ 7.83 (d, *J* = 6.9 Hz, 2H), 7.54 – 7.50 (m, 1H), 7.47 – 7.44 (m, 3H), 6.87 – 6.86 (m, 1H), 6.66 (dd, *J* = 3.0, 1.8 Hz, 1H), 1.56 (s, 9H). ^{13}C NMR (126 MHz, Chloroform-*d*) δ 191.0, 140.4, 131.3, 129.0, 128.2, 125.2, 123.9, 119.4, 111.0, 56.0, 30.7.

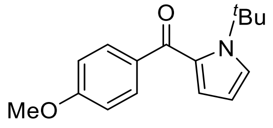

(1-(*tert*-butyl)-1*H*-pyrrol-2-yl)(4-methoxyphenyl)methanone (2-2e). Isolated yield 12% (7.7 mg, 0.030 mmol/0.25 mmol). Yellow oil. ^{1}H NMR (500 MHz, Chloroform-*d*) δ 7.82 (d, *J* = 8.8 Hz, 2H), 7.22 (dd, *J* = 2.8, 1.8 Hz, 1H), 6.93 (d, *J* = 8.8 Hz, 2H), 6.61 (dd, *J* = 3.9, 1.8 Hz, 1H), 6.08 (dd, *J* = 3.9, 2.8 Hz, 1H), 3.87 (s, 3H), 1.74 (s, 9H). ^{13}C NMR (126 MHz, Chloroform-*d*) δ 186.4, 162.6, 133.8, 132.0, 132.1, 127.0, 125.1, 113.3, 106.4, 58.5, 55.6, 31.0. HRMS. Calculated for $C_{16}H_{19}NO_2Na^+$ (M+Na$^+$): 280.1308, found: 280.1307.

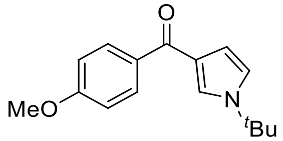

(1-(tert-butyl)-1H-pyrrol-3-yl)(4-methoxyphenyl)methanone (3-2e). Spectral data identical to reported compound.[17] Isolated yield 74% (47.6 mg, 0.18 mmol/0.25 mmol). Yellow oil. ^{1}H NMR (500 MHz, Chloroform-*d*) δ 7.88 (d, J = 8.8 Hz, 2H), 7.44 (t, *J* = 2.0 Hz, 1H), 6.95 (d, *J* = 8.8 Hz, 2H), 6.86 – 6.85 (m, 1H), 6.64 (dd, *J* = 3.0, 1.8 Hz, 1H), 3.87 (s, 3H), 1.56 (s, 9H).^{13}C NMR (126 MHz, Chloroform-*d*) δ 189.8, 162.3, 132.9, 131.3, 124.7, 124.0, 119.1, 113.5, 110.9, 55.9, 55.5, 30.7.

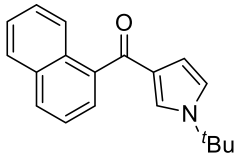

(1-(tert-butyl)-1H-pyrrol-3-yl)(naphthalen-1-yl)methanone (2f). Isolated yield 63% (43.9 mg, 0.16 mmol/0.25 mmol). Yellow oil. ^{1}H NMR (500 MHz, Chloroform-*d*) δ 8.22 – 8.19 (m, 1H), 7.94 (d, *J* = 8.3 Hz, 1H), 7.90 – 7.87 (m, 1H), 7.67 (dd, *J* = 7.0, 1.1 Hz, 1H), 7.52 – 7.47 (m, 3H), 7.33 (t, *J* = 2.0 Hz, 1H), 6.86 – 6.85 (m, 1H), 6.64 (dd, *J* = 3.0, 1.7 Hz, 1H), 1.52 (s, 9H). ^{13}C NMR (126 MHz, Chloroform-*d*) δ 192.4, 138.6, 133.9, 130.8, 130.2, 128.2, 126.8, 126.3, 126.2, 126.1, 125.9, 125.8, 124.5, 119.7, 110.8, 56.1, 30.7. HRMS. Calculated for $C_{19}H_{19}NONa^+$ (M+Na$^+$): 300.1359, found: 300.1362.

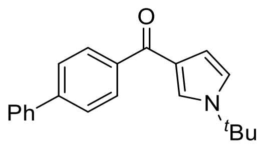

[1,1'-biphenyl]-4-yl(1-(tert-butyl)-1H-pyrrol-3-yl)methanone (2g). Isolated yield 47% (35.8 mg, 0.12 mmol/0.25 mmol). Brown solid. ^{1}H NMR (500 MHz, Chloroform-*d*) δ 7.93 (d, J = 8.4 Hz, 2H), 7.69 (d, *J* = 8.4 Hz, 2H), 7.65 (dd, *J* = 8.2, 1.1 Hz, 2H), 7.49 – 7.46 (m, 3H),

7.41 – 7.37 (m, 1H), 6.89 – 6.88 (m, 1H), 6.70 (dd, *J* = 3.0, 1.8 Hz, 1H), 1.58 (s, 9H). ^{13}C NMR (126 MHz, Chloroform-*d*) δ 190.6, 144.1, 140.5, 139.1, 129.7, 129.0, 128.0, 127.4, 127.0, 125.1, 124.0, 119.4, 111.0, 56.1, 30.8. HRMS. Calculated for $C_{21}H_{21}NONa^+$ (M+Na$^+$): 326.1515, found: 326.1516.

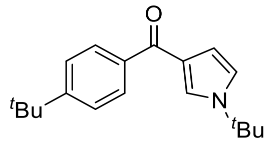

(1-(tert-butyl)-1H-pyrrol-3-yl)(4-(tert-butyl)phenyl)methanone (2h). Isolated yield 44% (NMR yield 70%) (31.1 mg, 0.11 mmol/0.25 mmol). Yellow oil. ^{1}H NMR (500 MHz, Chloroform-*d*) δ 7.79 (d, *J* = 8.5 Hz, 2H), 7.49 – 7.45 (m, 3H), 6.86 (dd, *J* = 3.0, 2.4 Hz, 1H), 6.68 (dd, *J* = 3.0, 1.8 Hz, 1H), 1.57 (s, 9H), 1.36 (s, 9H).^{13}C NMR (126 MHz, Chloroform-*d*) δ 190.7, 154.8, 137.5, 129.0, 125.2, 125.0, 124.0, 119.3, 110.9, 56.0, 35.1, 31.4, 30.7. HRMS. Calculated for $C_{19}H_{25}NONa^+$ (M+Na$^+$): 306.1828, found: 306.1830.

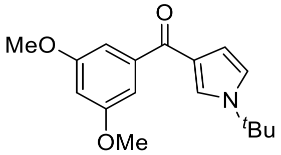

(1-(tert-butyl)-1H-pyrrol-3-yl)(3,5-dimethoxyphenyl) methanone (2i). Isolated yield 35% (25.1mg, 0.087 mmol/0.25 mmol). Yellow oil. ^{1}H NMR (500 MHz, Chloroform-*d*) δ 7.46 – 7.45 (m, 1H), 6.97 (d, *J* = 2.3 Hz, 2H), 6.86 (dd, *J* = 3.0, 2.4 Hz, 1H), 6.67 (dd, *J* = 3.0, 1.8 Hz, 1H), 6.62 (t, *J* = 2.3 Hz, 1H), 3.83 (s, 6H), 1.56 (s, 9H).^{13}C NMR (126 MHz, Chloroform-*d*) δ 190.5, 160.6, 142.3, 125.3, 123.8, 119.5, 110.9, 106.8, 103.8, 56.1, 55.7, 30.7. HRMS. Calculated for $C_{17}H_{21}NO_3Na^+$ (M+Na$^+$): 310.1414, found: 310.1419.

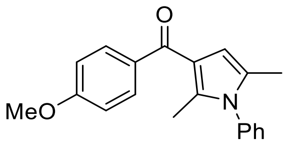

(2,5-dimethyl-1-phenyl-1H-pyrrol-3-yl)(4-methoxyphenyl) methanone (2j). Isolated yield 67% (50.9 mg, 0.17 mmol/0.25 mmol). Yellow oil. ^{1}H NMR (500 MHz, Chloroform-*d*) δ 7.89 (d, *J* = 8.8 Hz, 2H), 7.53 – 7.45 (m, 3H), 7.23 (dd, *J* = 7.0, 1.5 Hz, 2H), 6.95 (d, *J* = 8.8 Hz, 2H), 6.23 – 6.22 (m, 1H), 3.88 (s, 3H), 2.31 (s, 3H), 2.00 (s, 3H).^{13}C NMR (126 MHz, Chloroform-*d*) δ 191.4, 162.2, 137.8, 136.9, 133.6, 131.5, 129.5, 128.7, 128.4, 128.2, 119.9, 113.3, 109.8, 55.5, 13.1, 12.8. HRMS. Calculated for $C_{20}H_{19}NO_2Na^+$ (M+Na$^+$): 328.1308, found: 328.1298.

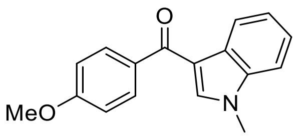

(4-methoxyphenyl)(1-methyl-1H-indol-3-yl)methanone (2k). Isolated yield 66% (43.5 mg, 0.16 mmol/0.25 mmol). Brown solid. ^{1}H NMR (500 MHz, Chloroform-*d*) δ 8.39-8.37 (m 1H), 7.83 (d, *J* = 8.8 Hz, 2H), 7.52 (s, 1H), 7.36 – 7.30 (m, 3H), 6.97 (d, *J* = 8.8 Hz, 2H), 3.88 (s, 3H), 3.82 (s, 3H).^{13}C NMR (126 MHz, Chloroform-*d*) δ 189.8, 162.3, 137.5, 137.2, 133.5, 131.0, 127.4, 123.5, 122.7, 122.5, 115.7, 113.6, 109.7, 55.5, 33.6. HRMS. Calculated for $C_{17}H_{15}NO_2Na^+$ (M+Na$^+$): 288.0995, found: 288.0987.

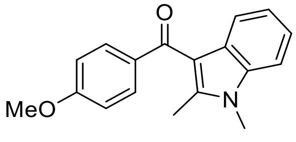

(1,2-dimethyl-1H-indol-3-yl)(4-methoxyphenyl)methanone (2l). Isolated yield 54% (37.6 mg, 0.13 mmol/0.25 mmol). Brown solid. ^{1}H NMR (500 MHz, Chloroform-*d*) δ 7.79 (d, *J* = 8.8 Hz, 2H), 7.36 (d, *J* = 8.0 Hz, 1H), 7.32 (d, *J* = 8.2 Hz, 1H), 7.22-7.19 (m, 1H), 7.09-7.06 (m, 1H), 6.94 (d, *J* = 8.8 Hz, 2H), 3.89 (s, 3H), 3.74 (s, 3H), 2.60 (s, 3H).^{13}C NMR (126 MHz, Chloroform-*d*) δ 191.8, 162.7, 144.0, 136.6, 133.9, 131.7, 127.2, 122.0,

121.3, 121.0, 113.9, 113.5, 109.2, 55.5, 29.8, 12.5. HRMS: Calculated for $C_{18}H_{17}NO_2Na^+$ ($M+Na^+$): 302.1151, found: 302.1157.

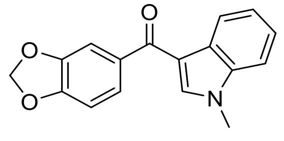

Benzo[d][1,3]dioxol-5-yl(1-methyl-1H-indol-3-yl)methanone (2m). Isolated yield 52% (36.5 mg, 0.13 mmol/0.25 mmol). Red oil. 1H NMR (500 MHz, Chloroform-d) δ 8.39 – 8.36 (m, 1H), 7.56 (s, 1H), 7.42 (dd, J = 8.0, 1.7 Hz, 1H), 7.39 – 7.31 (m, 4H), 6.89 (d, J = 8.0 Hz, 1H), 6.06 (s, 2H), 3.86 (s, 3H). ^{13}C NMR (126 MHz, Chloroform-d) δ 189.3, 150.4, 147.9, 137.6, 137.3, 135.3, 127.4, 124.4, 123.7, 122.8, 122.7, 115.6, 109.7, 109.3, 107.9, 101.7, 33.7. HRMS: Calculated for $C_{17}H_{13}NO_3Na^+$ ($M+Na^+$): 302.0788, found: 302.0784.

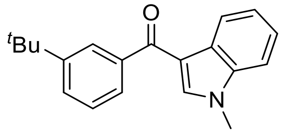

(3-(tert-butyl)phenyl)(1-methyl-1H-indol-3-yl)methanone (2n). Isolated yield 38% (27.5 mg, 0.094 mmol/0.25 mmol). Yellow oil. ^1H NMR (500 MHz, Chloroform-d) δ 8.44 – 8.41 (m, 1H), 7.87 (t, J = 1.7 Hz, 1H), 7.63 – 7.57 (m, 2H), 7.54 (s, 1H), 7.42 – 7.33 (m, 4H), 3.85 (s, 3H), 1.38 (s, 9H). ^{13}C NMR (126 MHz, Chloroform-d) δ 191.5, 151.5, 140.8, 137.9, 137.7, 128.3, 128.0, 127.4, 126.1, 125.9, 123.7, 122.9, 122.8, 115.9, 109.7, 35.0, 33.7, 31.5. HRMS: Calculated for $C_{20}H_{21}NONa^+$ ($M+Na^+$): 314.1515, found: 314.1507.

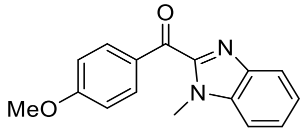

(4-methoxyphenyl)(1-methyl-1H-benzo[d]imidazol-2-yl)methanone (2o). Spectral data identical to reported compound.[19] Isolated yield 62% (41.1 mg, 0.15 mmol/0.25 mmol). Yellow solid. ^1H NMR (500 MHz, Chloroform-*d*) δ 8.40 (d, *J* = 9.0 Hz, 2H), 7.92 (d, *J* = 8.1 Hz, 1H), 7.50 – 7.44 (m, 2H), 7.39-7.36 (m, 1H), 7.01 (d, *J* = 9.0 Hz, 2H), 4.13 (s, 3H), 3.91 (s, 3H). ^{13}C NMR (126 MHz, Chloroform-*d*) δ 184.9, 164.2, 147.3, 142.0, 136.6, 133.9, 129.9, 125.5, 123.6, 122.0, 113.9, 110.5, 55.7, 32.3.

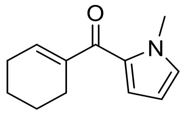

Cyclohex-1-en-1-yl(1-methyl-1H-pyrrol-2-yl)methanone (3a). Isolated yield 56% (37.4 mg, 0.20 mmol) ^1H NMR (500 MHz, Chloroform-d) δ 6.82 (t, J = 2.0 Hz, 1H), 6.72 (dd, J = 4.0, 1.7 Hz, 1H), 6.59 (tt, J = 3.8, 1.7 Hz, 1H), 6.09 (dd, J = 4.0, 2.5 Hz, 1H), 3.90 (s, 3H), 2.39 – 2.35 (m, 2H), 2.25 – 2.21 (m, 2H), 1.74 – 1.63 (m, 4H). ^{13}C NMR (126 MHz, Chloroform-d) δ 188.5, 139.6, 138.1, 130.7, 130.7, 120.9, 107.5, 37.2, 25.8, 24.6, 22.3, 22.0. HRMS. Calculated for $C_{12}H_{15}NONa^+$ ($M+Na^+$): 212.1046, found: 212.1041.

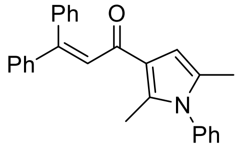

1-(2,5-dimethyl-1-phenyl-1*H*-pyrrol-3-yl)-3,3-diphenylprop-2-en-1-one (3b). Isolated yield 64% (60.7 mg, 0.16 mmol/0.25 mmol). Yellow oil. ^1H NMR (400 MHz, Chloroform-*d*) δ 7.51 – 7.42 (m, 3H), 7.38 – 7.34 (m, 5H), 7.33 – 7.30 (m, 3H), 7.28 – 7.26 (m, 2H), 7.17 – 7.15 (m, 2H), 7.05 (s, 1H), 6.37 (d, *J* = 0.9 Hz, 1H), 2.26 (s, 3H), 1.96 (s, 3H). ^{13}C NMR (126 MHz, Chloroform-*d*) δ 188.3, 151.6, 142.5, 139.9, 137.6, 136.6, 129.9, 129.5, 128.8, 128.8, 128.7, 128.6, 128.4, 128.2, 128.0, 128.0, 127.1, 122.1, 107.9, 13.2, 12.8. HRMS. Calculated for $C_{27}H_{23}NONa^+$ ($M+Na^+$): 400.1672, found: 400.1674.

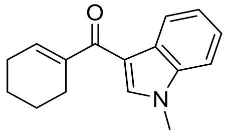

Cyclohex-1-en-1-yl(1-methyl-1*H*-indol-3-yl)methanone (3c). Isolated yield 52% (31.0 mg, 0.13 mmol/0.25mmol). Pale yellow solid. ^{1}H NMR (500 MHz, Chloroform-*d*) δ 8.35 – 8.33 (m, 1H), 7.56 (s, 1H), 7.35 – 7.27 (m, 3H), 6.54 (tt, *J* = 3.7, 1.7 Hz, 1H), 3.84 (s, 3H), 2.48 – 2.45 (m, 2H), 2.27 – 2.24 (m, 2H), 1.79 – 1.68 (m, 4H). ^{13}C NMR (126 MHz, Chloroform-*d*) δ 192.9, 140.8, 137.7, 136.4, 136.1, 127.4, 123.4, 122.8, 122.4, 115.4, 109.6, 33.5, 25.8, 24.8, 22.4, 22.1.

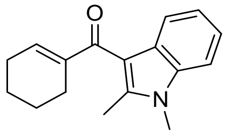

Cyclohex-1-en-1-yl(1,2-dimethyl-1H-indol-3-yl)methanone (3d). Isolated yield 60% (30.4 mg, 0.12 mmol/0.20 mmol). Pale yellow solid. ^{1}H NMR (500 MHz, $CDCl_3$) δ 7.76 (d, *J* = 7.7 Hz, 1H), 7.29 (d, *J* = 8.0 Hz, 1H), 7.22 – 7.19 (m, 1H), 7.17 – 7.14 (m, 1H), 6.51 (tt, *J* = 3.7, 1.6 Hz, 1H), 3.70 (s, 3H), 2.61 (s, 3H), 2.49 – 2.46 (m, 2H), 2.24 – 2.19 (m, 2H), 1.79 (dtd, *J* = 8.1, 6.1, 2.6 Hz, 2H), 1.72 (dtd, *J* = 9.2, 5.9, 2.6 Hz, 2H). ^{13}C NMR (126 MHz, $CDCl_3$) δ 194.8, 143.0, 141.2, 139.0, 136.6, 127.4, 121.8, 121.2, 120.9, 113.7, 109.2, 29.7, 26.0, 24.3, 22.5, 22.1, 12.5. HRMS: Calculated for $C_{17}H_{19}NONa^{+}$ ($M+Na^{+}$): 276.1359, found: 276.1346.

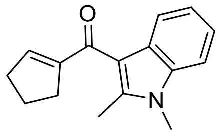

Cyclopent-1-en-1-yl(1,2-dimethyl-1H-indol-3-yl)methanone (3e). Isolated yield 60% (36.0 mg, 0.15 mmol/0.25 mmol). Pale yellow solid. ^{1}H NMR (500 MHz, $CDCl_3$) δ 7.79 (d, *J* = 7.9 Hz, 1H), 7.29 (d, *J* = 8.1 Hz, 1H), 7.22 – 7.19 (m, 1H), 7.17 – 7.13 (m, 1H), 6.43 (p, *J* = 2.3 Hz, 1H), 3.70 (s, 3H), 2.82 – 2.78 (m, 2H), 2.62 (s, 3H), 2.61 – 2.56 (m, 2H), 2.05 (p, *J* = 7.6 Hz, 2H). ^{13}C NMR (126 MHz, $CDCl_3$) δ 190.8, 147.1, 142.7, 142.7, 136.6, 127.0, 121.9, 121.2, 120.9, 114.6, 109.2, 34.1, 32.1, 29.7, 23.3, 12.5. HRMS: Calculated for $C_{16}H_{17}NONa^{+}$ ($M+Na^{+}$): 262.1199, found: 262.1202.

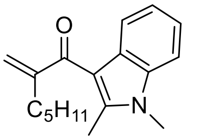

1-(1,2-dimethyl-1H-indol-3-yl)-2-methyleneheptan-1-one (3f). Isolated yield 79% (21.2 mg, 0.079 mmol/0.10 mmol). Yellow oil. ^{1}H NMR (500 MHz, Chloroform-d) δ 7.91 (d, J = 7.8 Hz, 1H), 7.30 (d, J = 8.1 Hz, 1H), 7.24 – 7.21 (m, 1H), 7.20 – 7.13 (m, 1H), 5.61 (q, J = 1.4 Hz, 1H), 5.57 (m, 1H), 3.72 (s, 3H), 2.67 (s, 3H), 2.54 – 2.50 (m, 2H), 1.61 – 1.57 (m, 2H), 1.41-1.31 (m, 4H), 0.89 (t, J = 7.1 Hz, 3H). ^{13}C NMR (126 MHz, Chloroform-d) δ 194.8, 152.0, 144.3, 136.7, 127.2, 122.1, 121.5, 121.2, 121.1, 113.4, 109.2, 32.7, 31.9, 29.8, 28.1, 22.7, 14.2, 12.7. HRMS. Calculated for $C_{18}H_{23}NONa^{+}$ ($M+Na^{+}$): 292.1672, found: 292.1667.

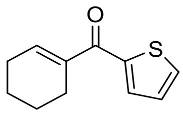

Cyclohex-1-en-1-yl(thiophen-2-yl)methanone (3g). Spectral data identical to reported compound.[20] Isolated yield 74% (35.5 mg, 0.18 mmol/0.25 mmol). Yellow liquid. ^{1}H NMR (500 MHz, $CDCl_3$) δ 7.59 (ddd, *J* = 5.1, 4.4, 1.1 Hz, 2H), 7.09 (dd, *J* = 4.9, 3.8 Hz, 1H), 6.78 (tt, *J* = 3.8, 1.7 Hz, 1H), 2.42 – 2.39 (m, 2H), 2.29 (m, 2H), 1.78 – 1.65 (m, 4H). ^{13}C NMR (126 MHz, $CDCl_3$) δ 189.5, 143.7, 140.4, 139.0, 133.0, 132.9, 127.6, 26.0, 24.5, 22.1, 21.8.

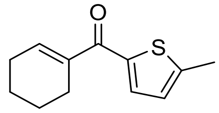

Cyclohex-1-en-1-yl(5-methylthiophen-2-yl)methanone (3h). Isolated yield 75% (38.5 mg, 0.19 mmol/0.25 mmol). Yellow oil. ^{1}H NMR (500 MHz, Chloroform-*d*) δ 7.40 (d, *J* = 3.7 Hz, 1H), 6.76 – 6.75 (m, 1H), 6.70 (tt, *J* = 3.8, 1.7 Hz, 1H), 2.52 (s, 3H), 2.40 – 2.36 (m, 2H), 2.28 – 2.24 (m, 2H), 1.74 – 1.64 (m, 4H).^{13}C NMR (126 MHz, Chloroform-*d*) δ 189.4, 148.9, 141.6, 139.2, 138.8, 133.7, 126.3, 25.9, 24.6, 22.2, 21.8, 16.0. HRMS. Calculated for $C_{12}H_{14}OSNa^+$ (M+Na$^+$): 229.0658, found: 229.0655.

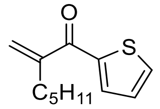

2-methylene-1-(thiophen-2-yl)heptan-1-one (3i). Isolated yield 43% (22.2 mg, 0.11 mmol/0.25 mmol). Yellow liquid. ^{1}H NMR (500 MHz, $CDCl_3$) δ 7.66 (s, 1H), 7.65 (q, *J* = 1.2 Hz, 1H), 7.12 (dd, J = 4.8 – 3.9, 1H), 5.73 (d, *J* = 0.9 Hz, 1H), 5.69 (q, *J* = 1.4 Hz, 1H), 2.46 – 2.43 (m, 2H), 1.48 (p, *J* = 7.3 Hz, 2H), 1.34 – 1.30 (m, 4H), 0.89 – 0.86 (m, 3H). ^{13}C NMR (126 MHz, $CDCl_3$) δ 190.2, 149.0, 144.0, 134.1, 134.0, 127.9, 122.3, 32.9, 31.6, 27.9, 22.6, 14.1. HRMS: Calculated for $C_{12}H_{16}OSNa^+$ (M+Na$^+$): 231.0805, found: 231.0814.

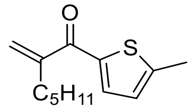

2-methylene-1-(5-methylthiophen-2-yl)heptan-1-one (3j). Isolated yield 37% (16.4 mg, 0.074 mmol/0.20 mmol). Yellow oil. ^{1}H NMR (500 MHz, Chloroform-*d*) δ 7.47 (d, *J* = 3.7 Hz, 1H), 6.79 – 6.78 (m, 1H), 5.66 (s, 1H), 5.62 (d, J = 1.1 Hz, 1H), 2.54 (s, 3H), 2.44 – 2.40 (m, 2H), 1.49 – 1.43 (m, 2H), 1.33 – 1.29 (m, 4H), 0.89 – 0.86 (m, 3H).^{13}C NMR (126 MHz, Chloroform-*d*) δ 190.0, 150.3, 148.9, 141.8, 134.8, 126.7, 121.4, 33.0, 31.6, 27.9, 22.6, 16.2, 14.2. HRMS. Calculated for $C_{13}H_{19}SO^+$ (MH$^+$): 223.1151, found: 223.1150.

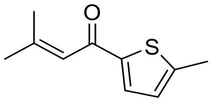

3-methyl-1-(5-methylthiophen-2-yl)but-2-en-1-one (3k). Isolated yield 81% (36.5 mg, 0.20 mmol/0.25 mmol). Yellow solid. ^{1}H NMR (500 MHz, $CDCl_3$) δ 7.50 (d, *J* = 3.7 Hz, 1H), 6.78 – 6.77 (m, 1H), 6.61 – 6.60 (m, 1H), 2.52 (s, 3H), 2.23 (s, 3H), 1.98 (s, 3H). ^{13}C NMR (126 MHz, $CDCl_3$) δ 183.2, 156.5, 148.9, 144.8, 131.4, 126.6, 120.3, 28.0, 21.1, 16.0. HRMS. Calculated for $C_{10}H_{12}OSNa^+$ (M+Na$^+$): 203.0499, found: 203.0501.

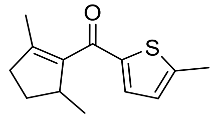

(2,5-dimethylcyclopent-1-en-1-yl)(5-methylthiophen-2-yl)methanone (3l). Isolated yield 41% (22.8 mg, 0.10 mmol/0.25 mmol). Yellow liquid. ^{1}H NMR (500 MHz, $CDCl_3$) δ 7.44 (d, *J* = 3.7 Hz, 1H), 6.80 (dd, *J* = 3.7, 1.1 Hz, 1H), 3.33 – 3.25 (m, 1H), 2.56 (d, J = 1.1 Hz, 3H), 2.53 – 2.38 (m, 2H), 2.21 (dtd, *J* = 12.5, 8.4, 4.2 Hz, 1H), 1.76 – 1.75 (m, 3H), 1.47 (dtd, *J* = 12.5, 9.0, 7.8 Hz, 1H), 1.07 (d, *J* = 6.9 Hz, 3H). ^{13}C NMR (126 MHz, $CDCl_3$) δ 189.7, 150.1, 144.8, 143.5, 141.4, 134.3, 126.8, 43.5, 38.4, 31.9, 19.9, 16.6, 16.2. HRMS: Calculated for $C_{13}H_{16}OSNa^+$ (M+Na$^+$): 243.0815, found: 243.0814.

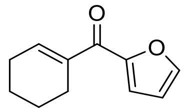

Cyclohex-1-en-1-yl(furan-2-yl)methanone (3m). Spectral data identical to reported compound.[20] Isolated yield 55% (19.3 mg, 0.11 mmol/0.20 mmol). Yellow oil. ^{1}H NMR (500 MHz, Chloroform-*d*) δ 7.59 (dd, *J* = 1.7, 0.7 Hz, 1H), 7.07 (dd, *J* = 3.5, 0.7 Hz, 1H), 6.96 (tt, *J* = 3.8, 1.7 Hz, 1H),

6.50 (dd, *J* = 3.5, 1.7 Hz, 1H), 2.41 – 2.37 (m, 2H), 2.31 – 2.27 (m, 2H), 1.75 – 1.62 (m, 4H).^{13}C NMR (126 MHz, Chloroform-*d*) δ 184.1, 152.4, 146.3, 141.0, 138.4, 118.8, 111.7, 26.2, 24.1, 22.1, 21.8. HRMS: Calculated for $C_{11}H_{12}O_2Na^+$ (M+Na$^+$): 199.0730, found: 199.0722.

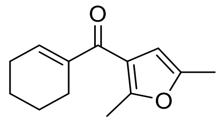

Cyclohex-1-en-1-yl(2,5-dimethylfuran-3-yl)methanone (3n). Isolated yield 74% (37.7 mg, 0.18 mmol/0.25 mmol). Yellow oil. ^{1}H NMR (500 MHz, Chloroform-*d*) δ 6.64 (tt, *J* = 3.8, 1.6 Hz, 1H), 6.09 – 6.08 (m, 1H), 2.42 (s, 3H), 2.36 – 2.32 (m, 2H), 2.26 – 2.22 (m, 5H), 1.75 – 1.55 (m, 4H).^{13}C NMR (126 MHz, Chloroform-*d*) δ 193.0, 156.0, 149.4, 140.5, 140.0, 121.2, 107.6, 26.0, 23.9, 22.2, 21.9, 14.0, 13.3. HRMS: Calculated for $C_{13}H_{16}O_2Na^+$ (M+Na$^+$): 227.1043, found: 227.1038.

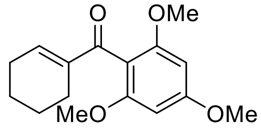

Cyclohex-1-en-1-yl(2,4,6-trimethoxyphenyl)methanone (3o). Isolated yield 93% (25.8 mg, 0.093 mmol/0.10 mmol). Yellow liquid. ^{1}H NMR (400 MHz, $CDCl_3$) δ 6.55 (m, 1H), 6.11 (s, 2H), 3.82 (s, 3H), 3.72 (s, 6H), 2.39 – 2.34 (m, 2H), 2.21 – 2.15 (m, 2H), 1.71 – 1.59 (m, 4H). ^{13}C NMR (101 MHz, $CDCl_3$) δ 196.8, 161.9, 158.4, 144.0, 140.8, 111.8, 90.8, 56.0, 55.5, 26.3, 22.8, 22.1, 21.8. HRMS: Calculated for $C_{16}H_{20}O_4Na^+$ (M+Na$^+$): 299.1254, found: 299.1244.

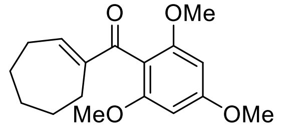

Cyclohept-1-en-1-yl(2,4,6-trimethoxyphenyl)methanone (3p). Isolated yield 70% (40.4 mg, 0.14 mmol/0.20 mmol). Yellow liquid. ^{1}H NMR (500 MHz, $CDCl_3$) δ 6.72 (t, *J* = 6.7 Hz, 1H), 6.11 (s, 2H), 3.82 (s, 3H), 3.72 (s, 6H), 2.64 – 2.62 (m , 2H), 2.30 – 2.26 (m, 2H), 1.82 – 1.77 (m, 2H), 1.56 – 1.52 (m, 4H). ^{13}C NMR (126 MHz, $CDCl_3$) δ 196.8, 161.9, 158.4, 148.4, 147.5, 112.0, 90.8, 56.0, 55.6, 32.5, 29.4, 26.4, 26.1, 25.6. HRMS: Calculated for $C_{17}H_{22}O_4Na^+$ (M+Na$^+$): 313.1403, found: 313.1410.

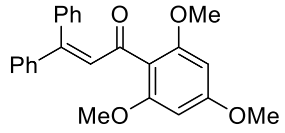

3,3-diphenyl-1-(2,4,6-trimethoxyphenyl)prop-2-en-1-one (3q). Isolated yield 53% (39.7 mg, 0.11 mmol/0.20 mmol). Yellow oil. ^{1}H NMR (500 MHz, Chloroform-*d*) δ 7.33 – 7.29 (m, 5H), 7.17 – 7.11 (m, 3H), 7.09 – 7.06 (m, 2H), 6.81 (s, 1H), 5.82 (s, 2H), 3.72 (s, 3H), 3.71 (s, 6H). ^{13}C NMR (126 MHz, Chloroform-*d*) δ 194.0, 162.4, 158.7, 152.8, 141.8, 138.7, 129.6, 129.4, 129.2, 128.6, 128.4, 127.8, 127.4, 113.6, 90.5, 55.8, 55.5. HRMS. Calculated for $C_{24}H_{22}O_4Na^+$ (M+Na$^+$): 397.1410, found: 397.1415.

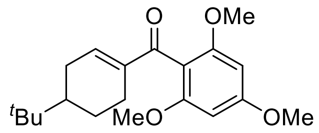

(4-(tert-butyl)cyclohex-1-en-1-yl)(2,4,6-trimethoxyphenyl) methanone (3r). Isolated yield 76% (50.8 mg, 0.15 mmol/0.20 mmol). Yellow solid ^{1}H NMR (500 MHz, $CDCl_3$) δ 6.57 – 6.55 (m, 1H), 6.11 (s, 2H), 3.82 (s, 3H), 3.72 (s, 6H), 2.72 – 2.67 (m, 1H), 2.25 – 2.12 (m, 2H), 1.99 – 1.92 (m, 2H), 1.37 – 1.30 (m, 1H), 1.16 (qd, *J* = 12.4, 5.0 Hz, 1H), 0.88 (s, 9H). ^{13}C NMR (126 MHz, $CDCl_3$) δ 196.5, 161.8, 158.4, 144.5, 140.7, 112.0,

90.8, 56.1, 55.6, 43.6, 32.3, 28.1, 27.3, 24.2, 23.6. HRMS: Calculated for $C_{20}H_{28}O_4Na^+$ (M+Na$^+$): 355.1880, found: 355.1878.

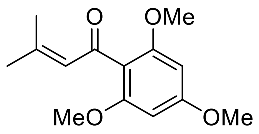

3-methyl-1-(2,4,6-trimethoxyphenyl)but-2-en-1-one (3s). Isolated yield 43% (10.8 mg, 0.043 mmol/0.10 mmol). Yellow liquid. ^{1}H NMR (500 MHz, $CDCl_3$) δ 6.25 – 6.24 (m, 1H), 6.10 (s, 2H), 3.82 (s, 3H), 3.77 (s, 6H), 2.17 (d, J = 0.9 Hz, 3H), 1.90 (d, J = 1.0 Hz, 3H). ^{13}C NMR (126 MHz, $CDCl_3$) δ 193.0, 162.1, 158.5, 154.3, 127.2, 115.2, 90.9, 56.1, 55.4, 28.1, 20.9. HRMS: Calculated for $C_{14}H_{18}O_4Na^+$ (M+Na$^+$): 273.1105, found: 273.1097.

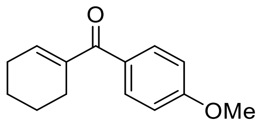

Cyclohex-1-en-1-yl(4-methoxyphenyl)methanone (3t). Spectral data identical to reported compound.[21] Isolated yield 56% (30.1 mg, 0.14 mmol/0.25 mmol). Yellow liquid. ^{1}H NMR (500 MHz, $CDCl_3$) δ 7.70 – 7.67 (m, 2H), 6.92 – 6.90 (m, 2H), 6.50 (tt, J = 3.9, 1.7 Hz, 1H), 3.86 (s, 3H), 2.42 – 2.39 (m, 2H), 2.28 – 2.24 (m, 2H), 1.76 – 1.71 (m, 2H), 1.70 – 1.65 (m, 2H). ^{13}C NMR (126 MHz, $CDCl_3$) δ 197.2, 162.5, 141.6, 138.7, 131.6, 131.0, 113.3, 55.4, 25.9, 24.4, 22.1, 21.7.

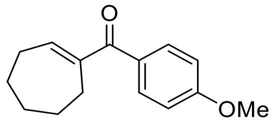

Cyclohept-1-en-1-yl(4-methoxyphenyl)methanone (3u). Spectral data identical to reported compound.[21] Isolated yield 49% (28.4 mg, 0.12 mmol/0.25 mmol). Yellow liquid. ^{1}H NMR (500 MHz, $CDCl_3$) δ 7.70 – 7.67 (m, 2H), 6.91 – 6.89 (m, 2H), 6.59 (t, J = 6.6 Hz, 1H), 3.85 (s, 3H), 2.60 – 2.58 (m, 2H), 2.36 – 2.33 (m, 2H), 1.86 – 1.81 (m, 2H), 1.63 – 1.57 (m, 4H). ^{13}C NMR (126 MHz, $CDCl_3$) δ 198.0, 162.6, 145.8, 145.3, 131.9, 131.1, 113.4, 55.5, 32.5, 29.4, 28.7, 26.8, 26.3.

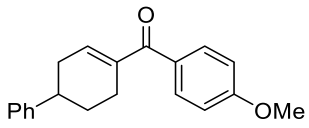

(4-methoxyphenyl)(1,2,3,6-tetrahydro-[1,1'-biphenyl]-4-yl)methanone (3v). Isolated yield 53% (38.7 mg, 0.13 mmol/0.25 mmol). Yellow solid. ^{1}H NMR (500 MHz, Chloroform-*d*) δ 7.75 – 7.73 (m, 2H), 7.35 – 7.32 (m, 2H), 7.25 – 7.22 (m, 3H), 6.95 – 6.93 (m, 2H), 6.59 – 6.58 (m, 1H), 3.87 (s, 3H), 2.92 – 2.86 (m, 1H), 2.73 – 2.68 (m, 1H), 2.61 – 2.36 (m, 3H), 2.15 – 2.10 (m, 1H), 1.89 – 1.80 (m, 1H).^{13}C NMR (126 MHz, Chloroform-*d*) δ 196.9, 162.7, 146.2, 140.7, 138.6, 131.7, 131.0, 128.7, 127.0, 126.5, 113. 5, 55.6, 39.6, 34.1, 29.5, 25.3. HRMS. Calculated for $C_{20}H_{20}NO_2Na^+$ (M+Na$^+$): 315.1361, found: 315.1356.

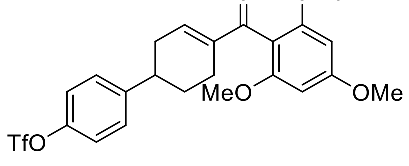

4'-(2,4,6-trimethoxybenzoyl)-1',2',3',6'-tetrahydro-[1,1'-biphenyl]-4-yl trifluoromethanesulfonate (3w). Isolated yield 53% (80.2 mg, 0.16 mmol/0.30 mmol). White solid. ^{1}H NMR (400 MHz, $CDCl_3$) δ 7.30 (d, J = 8.7 Hz, 2H), 7.20 (d, J = 8.8 Hz, 2H), 6.64 – 6.60 (m, 1H), 6.13 (s, 2H), 3.83 (s, 3H), 3.75 (s, 6H), 2.94 – 2.88 (m, 1H), 2.73 – 2.65 (m,

1H), 2.56 – 2.25 (m, 3H), 2.01 – 2.04 (m, 1H), 1.82 –1.73 (m, 1H). ^{13}C NMR (101 MHz, $CDCl_3$) δ 196.1, 161.9, 158.3, 147.9, 146.8, 141.9, 140.5, 128.6, 121.3, 118.8 (q, *J* = 320.7 Hz), 111.4, 90.7, 55.9, 55.4, 38.9, 33.9, 29.2, 23.1. ^{19}F NMR (377 MHz, $CDCl_3$) δ -72.88. HRMS. Calculated for $C_{23}H_{23}F_3O_7SNa^+$ (M+Na$^+$): 523.1009, found: 523.0987.

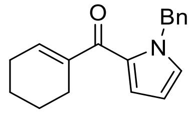

(1-benzyl-1*H*-pyrrol-2-yl)(cyclohex-1-en-1-yl)methanone (2-3x). ^{1}H NMR (500 MHz, Acetonitrile-*d*$_3$) δ 7.30 – 7.27 (m, 2H), 7.25 – 7.20 (m, 1H), 7.10 – 7.08 (m, 1H), 7.05 (d, *J* = 7.4 Hz, 2H), 6.78 (dd, *J* = 3.9, 1.7 Hz, 1H), 6.50 (tt, *J* = 3.7, 1.6 Hz, 1H), 6.17 (dd, *J* = 3.9, 2.6 Hz, 1H), 5.53 (s, 2H), 2.27 – 2.23 (m, 2H), 2.22 – 2.18 (m, 2H), 1.68 – 1.59 (m, 4H). ^{1}H NMR (500 MHz, $CDCl_3$) δ 7.31 – 7.27 (m, 2H), 7.25 – 7.21 (m, 1H), 7.13 – 7.09 (m, 2H), 6.91 (dd, *J* = 2.5, 1.7 Hz, 1H), 6.77 (dd, *J* = 4.0, 1.8 Hz, 1H), 6.57 (tt, *J* = 3.8, 1.7 Hz, 1H), 6.15 (dd, *J* = 4.0, 2.6 Hz, 1H), 5.55 (s, 2H), 2.35 (ddt, *J* = 6.1, 3.9, 2.1 Hz, 2H), 2.22 (dp, *J* = 6.2, 3.5, 3.1 Hz, 2H), 1.73 – 1.61 (m, 4H). ^{13}C NMR (126 MHz, Chloroform-*d*) δ 188.4, 139.7, 138.6, 138.3, 130.4, 130.1, 128.7, 127.4, 127.2, 121.5, 108.1, 52.1, 25.8, 24.5, 22.3, 21.9. HRMS. Calculated for $C_{18}H_{19}NONa^+$ (M+Na$^+$): 288.1359, found: 288.1371.

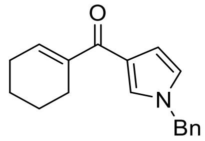

(1-benzyl-1*H*-pyrrol-3-yl)(cyclohex-1-en-1-yl)methanone (3-3x). ^{1}H NMR (500 MHz, Acetonitrile-*d*$_3$) δ 7.38 – 7.34 (m, 2H), 7.32 – 7.29 (m, 2H), 7.23 (d, *J* = 7.3 Hz, 2H), 6.75 (dd, *J* = 2.9, 2.1 Hz, 1H), 6.61 (tt, *J* = 3.8, 1.8 Hz, 1H), 6.48 (dd, *J* = 2.9, 1.7 Hz, 1H), 5.10 (s, 2H), 2.31 – 2.26 (m, 2H), 2.25 – 2.21 (m, 2H), 1.70 – 1.62 (m, 4H). ^{1}H NMR (500 MHz, $CDCl_3$) δ 7.37 – 7.30 (m, 3H), 7.20 (t, *J* = 1.9 Hz, 1H), 7.14 (d, *J* = 6.6 Hz, 2H), 6.64 (ddd, *J* = 9.0, 4.4, 2.1 Hz, 2H), 6.60 (dd, *J* = 3.0, 1.6 Hz, 1H), 5.07 (s, 2H), 2.41 – 2.35 (m, 2H), 2.23 (tt, *J* = 6.1, 3.2 Hz, 2H), 1.72 – 1.62 (m, 4H). ^{13}C NMR (126 MHz, Chloroform-*d*) δ 192.4, 140.1, 137.6, 136.9, 129.1, 128.2, 127.3, 127.1, 124.8, 122.3, 111.1, 53.9, 25.9, 24.6, 22.3, 22.0. HRMS. Calculated for $C_{18}H_{19}NONa^+$ (M+Na$^+$): 288.1359, found: 288.1353.

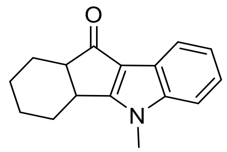

5-methyl-1,3,4,4a,4b,5,9b,10a-octahydroindeno[1,2-b]indol-10(2H)-one (4a). Isolated yield 66% (39.5 mg, 0.16 mmol/0.25 mmol). Brown solid. ^{1}H NMR (500 MHz, Chloroform-*d*) δ 7.94 (dt, *J* = 7.4, 0.9 Hz, 1H), 7.33 – 7.22 (m, 3H), 3.76 (s, 3H), 3.44 – 3.40 (m, 1H), 3.08 (q, *J* = 6.5 Hz, 1H), 2.20 – 2.10 (m, 2H), 1.83 – 1.76 (m, 1H), 1.62 – 1.55 (m, 2H), 1.49 – 1.33 (m, 3H). ^{13}C NMR (126 MHz, Chloroform-*d*) δ 197.3, 170.4, 143.0, 123.3, 122.4, 121.7, 121.3, 117.9, 110.0, 52.1, 33.9, 30.9, 27.4, 22.7, 21.1, 20.6. HRMS. Calculated for $C_{16}H_{17}NONa^+$ (M+Na$^+$): 262.1202, found: 262.1213.

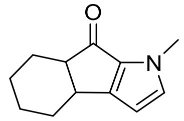

1-methyl-3b,4,5,6,7,7a-hexahydroindeno[2,1-b]pyrrol-8(1H)-one (4b). Isolated yield 60% (28.5 mg, 0.15 mmol/ 0.25 mmol). Yellow solid. ^{1}H NMR (500 MHz, Chloroform-d) δ 6.91 (d, J = 2.4 Hz, 1H), 6.01 (d, J = 2.4 Hz, 1H), 3.79 (s, 3H), 3.17 – 3.13 (m, 1H), 2.92 (q, J = 6.5 Hz, 1H), 2.03 – 1.94 (m, 1H), 1.94 – 1.74 (m, 2H), 1.56 – 1.46 (m, 2H), 1.42 – 1.30 (m, 3H). ^{13}C NMR (126 MHz, Chloroform-d) δ 194.2, 155.9, 134.7, 133.4, 104.2, 52.8, 34.2, 33.6, 29.1, 23.5, 21.1, 21.0. HRMS. Calculated for $C_{12}H_{15}NONa^+$ (M+Na$^+$): 212.1046, found: 212.1038.

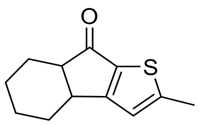

2-methyl-3b,4,5,6,7,7a-hexahydro-8H-indeno[2,1-b]thiophen-8-one (4c). Isolated yield 57% (29.5 mg, 0.14 mmol/ 0.25 mmol). Pale yellow solid. ^{1}H NMR (400 MHz, $CDCl_3$) δ 6.73 (s, 1H), 3.33 – 3.20 (m, 1H), 2.96 (q, *J* = 6.4 Hz, 1H), 2.57 (s, 3H), 2.00 (ddt, *J* = 18.0, 11.7, 4.3 Hz, 2H), 1.85 – 1.74 (m, 1H), 1.57 – 1.26 (m, 6H). ^{13}C NMR (101 MHz, $CDCl_3$) δ 199.0, 172.9, 156.7, 137.3, 121.7, 51.6, 37.0, 28.9, 23.4, 21.2, 21.1, 17.0. HRMS: Calculated for $C_{12}H_{15}OS^+$ (M+H$^+$): 207.08381, found: 207.08313.

V. References

1 S. S. Zalesskiy, V. P. Ananikov, *Organometallics* 2012, **31**, 2302-2309.

2 H. G. Lee, P. J. Milner, S. L. Buchwald, *Org. Lett.* 2013, **15**, 5602-5605.

3 a) S. Nomiyama, T. Tsuchimoto, *Advanced Synthesis & Catalysis* 2014, **356**, 3881-3891; b) A. H. Pang, A. Garzan, M. J. Larsen, T. J. McQuade, S. Garneau-Tsodikova, O. V. Tsodikov, *ACS Chem. Biol.* 2016, **11**, 3084-3092; c) K. C. Miles, S. M. Mays, B. K. Southerland, T. J. Auvil, D. M. Ketcha, *ARKIVOC* 2009, **2009**, 181-190; d) S. Islam, I. Larrosa, *Chem. Eur. J.* 2013, **19**, 15093-15096; e) S. Islam, I. Larrosa, *Chemistry* 2013, **19**, 15093-15096.

4 a) P. J. Stang, W. Treptow, *Synthesis* 1980, **4**, 283-284; b) Z. X. Tian, J. B. Qiao, G. L. Xu, X. Pang, L. Qi, W. Y. Ma, Z. Z. Zhao, J. Duan, Y. F. Du, P. Su, X. Y. Liu, X. Z. Shu, *J. Am. Chem. Soc.* 2019, **141**, 7637-7643.

5 Z. Huang, Z. Liu, J. S. Zhou, *J. Am. Chem. Soc.* 2011, **133**, 15882-15885.

6 C. L. Hugelshofer, V. Palani, R. Sarpong, *Org. Lett.* 2018, **20**, 2649-2653.

7 P. J. Tambade, Y. P. Patil, A. G. Panda, B. M. Bhanage, *Eur. J. Org. Chem.* 2009, **2009**, 3022-3025.

8 T. Maegawa, Y. Kitamura, S. Sako, T. Udzu, A. Sakurai, A. Tanaka, Y. Kobayashi, K. Endo, U. Bora, T. Kurita, A. Kozaki, Y. Monguchi, H. Sajiki, *Chem. Eur. J.* 2007, **13**, 5937-5943.

9 D. Gill, A. J. Hester, G. C. Lloyd-Jones, *Org. Biomol. Chem.* 2004, **2**, 2547-2548.

10 H. Sajiki, A. Mori, T. Mizusaki, T. Ikawa, T. Maegawa, K. Hirota, *Org. Lett.* 2006, **8**, 987-990.

11 R. L. Hartman, J. R. Naber, S. L. Buchwald, K. F. Jensen, *Angew Chem Int Ed* 2010, **49**, 899-903.

12 P. J. Stang, M. G. Mangum, D. P. Fox, P. Haak, *J. Am. Chem. Soc.* 1974, **96**, 4562-4569.

13 X. Su, H. Huang, Y. Yuan, Y. Li, *Angew. Chem. Int. Ed.* 2017, **56**, 1338-1341.

14 A. G. Martínez, A. H. Fernández, R. M. Alvarez, J. O. Barcina, *Rapid Commun. Mass Spectrom.* 1994, **8**, 427-431.

15 S. Zhang, H. Neumann, M. Beller, *Org. Lett.* 2019, **21**, 3528-3532.

16 E. J. Cho, S. L. Buchwald, *Org. Lett.* 2011, **13**, 6552-6555.

17 J. Tjutrins, B. A. Arndtsen, *J. Am. Chem. Soc.* 2015, **137**, 12050-12054.

18 S. P. Chou, T. Yuan, *Synthesis* 1991, **1991**, 171-172.

19 X. F. Wu, P. Anbarasan, H. Neumann, M. Beller, *Angew. Chem. Int. Ed.* 2010, **49**, 7316-7319.

20 A. Nakhai, J. Bergman, *Tetrahedron* 2009, **65**, 2298-2306.

21 Q. Wang, C. Chen, *Tetrahedron Lett.* 2008, **49**, 2916-2921.

VI. NMR Spectra

^{1}H, ^{19}F and ^{13}C NMR data for 1a.

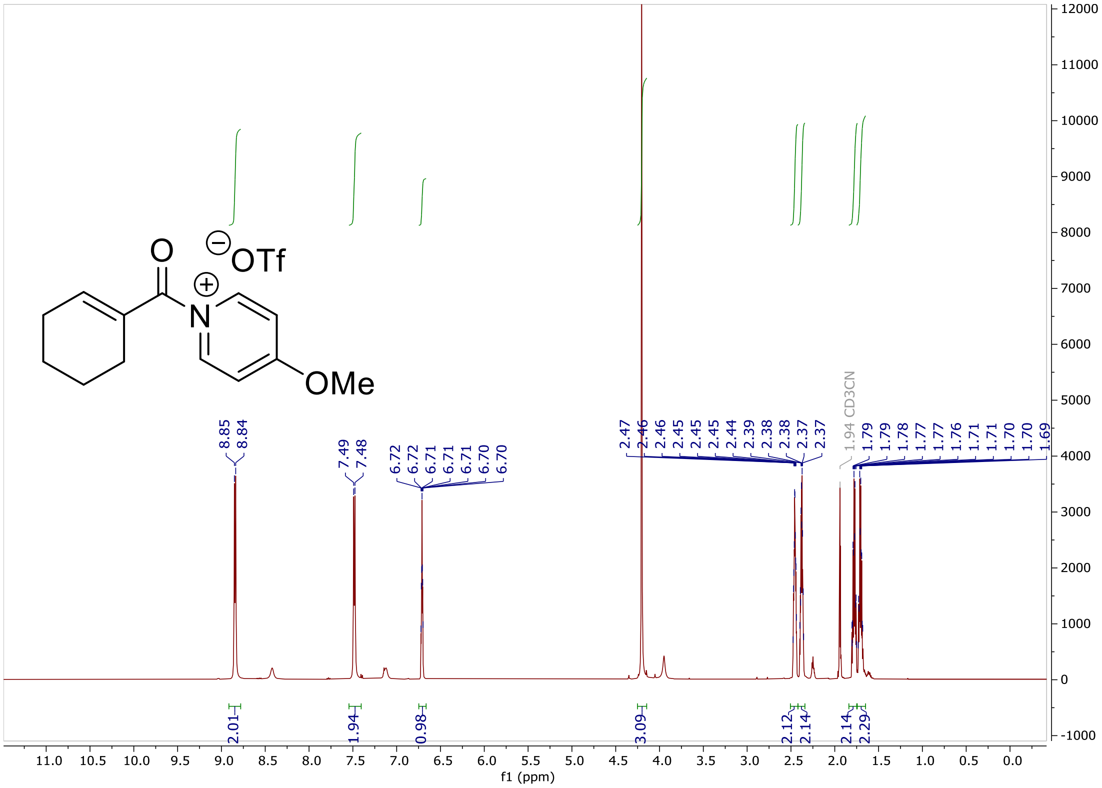

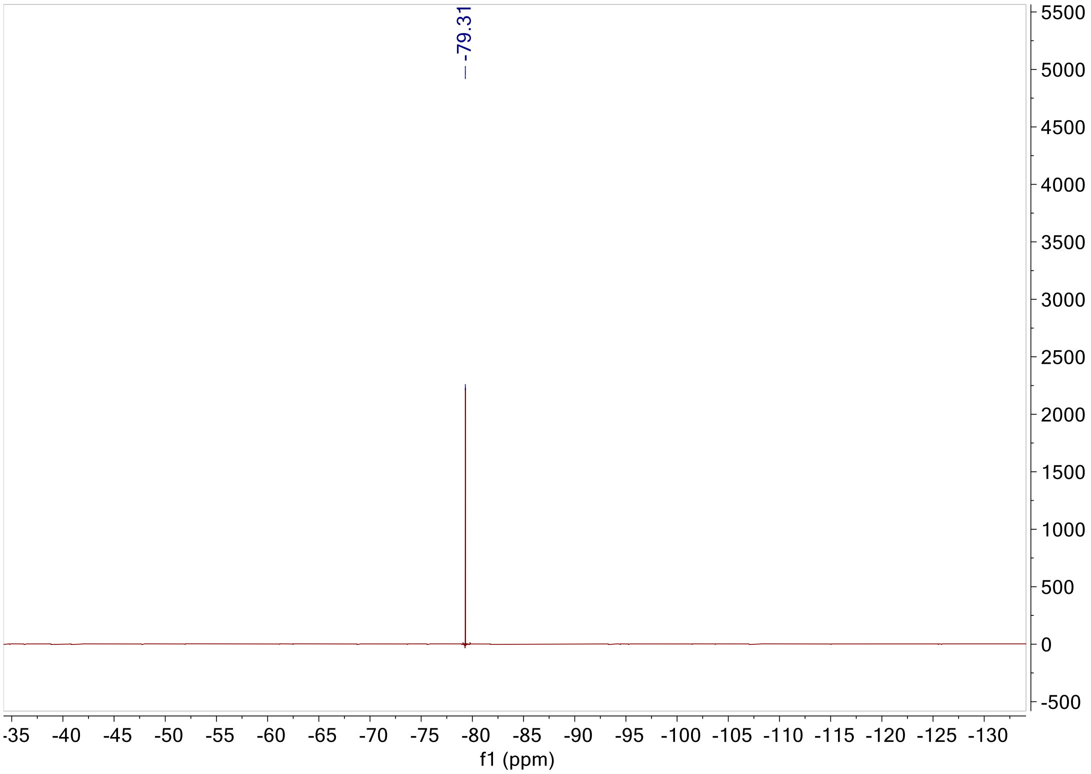

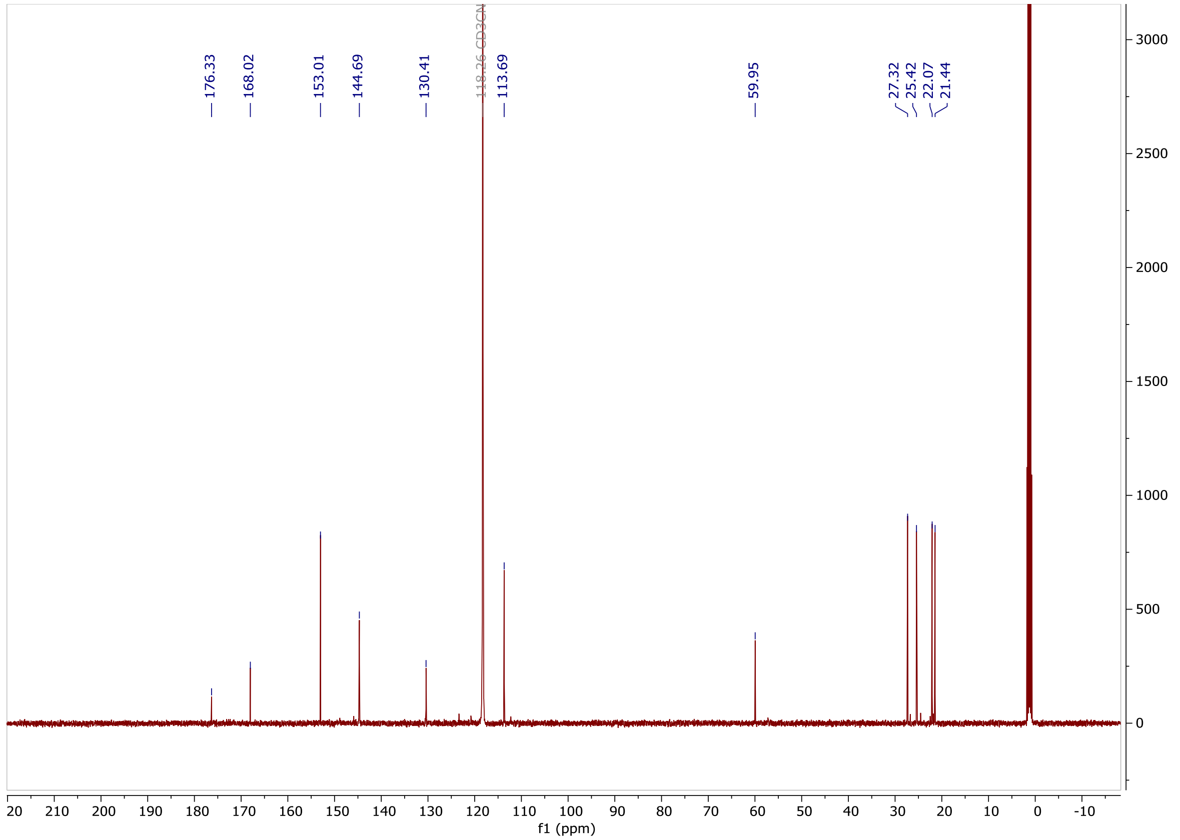
176.33
168.02
153.01
144.69
130.41
118.26 CD3CN
113.69
59.95
27.32
25.42
22.07
21.44
f1 (ppm)

^{1}H, ^{19}F and ^{13}C NMR data for 1b.

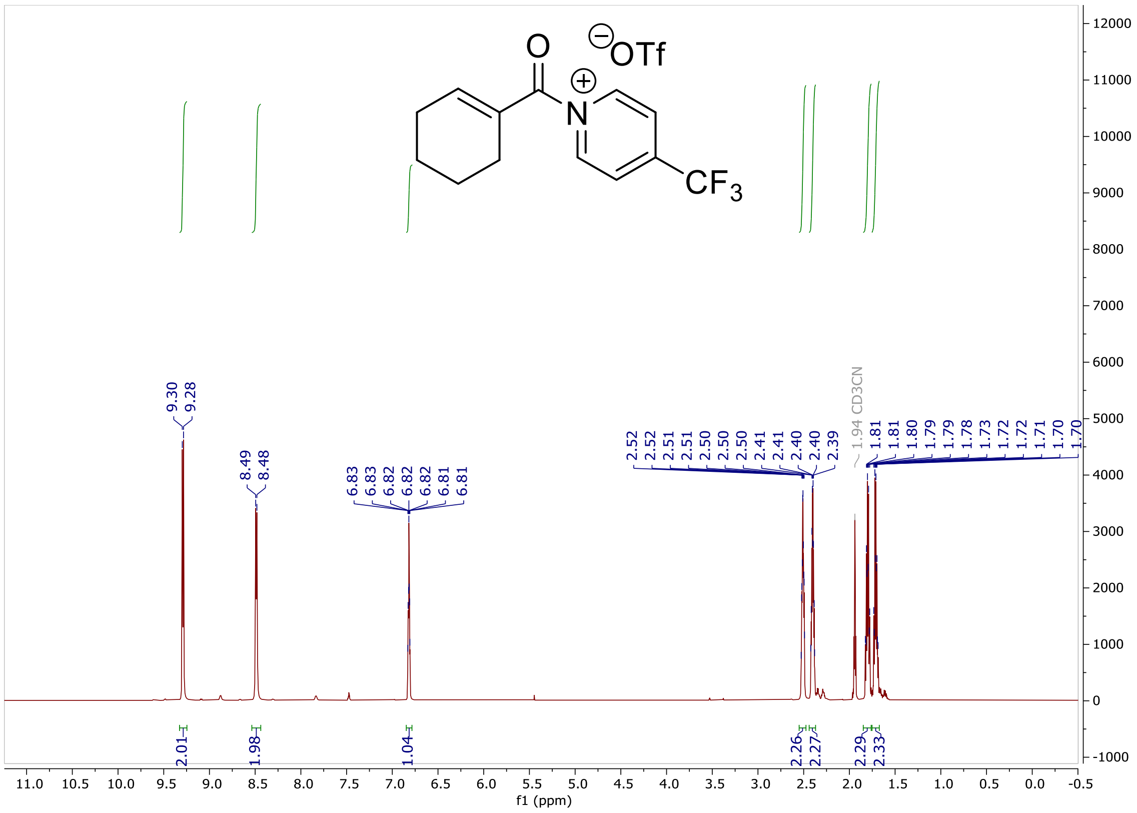

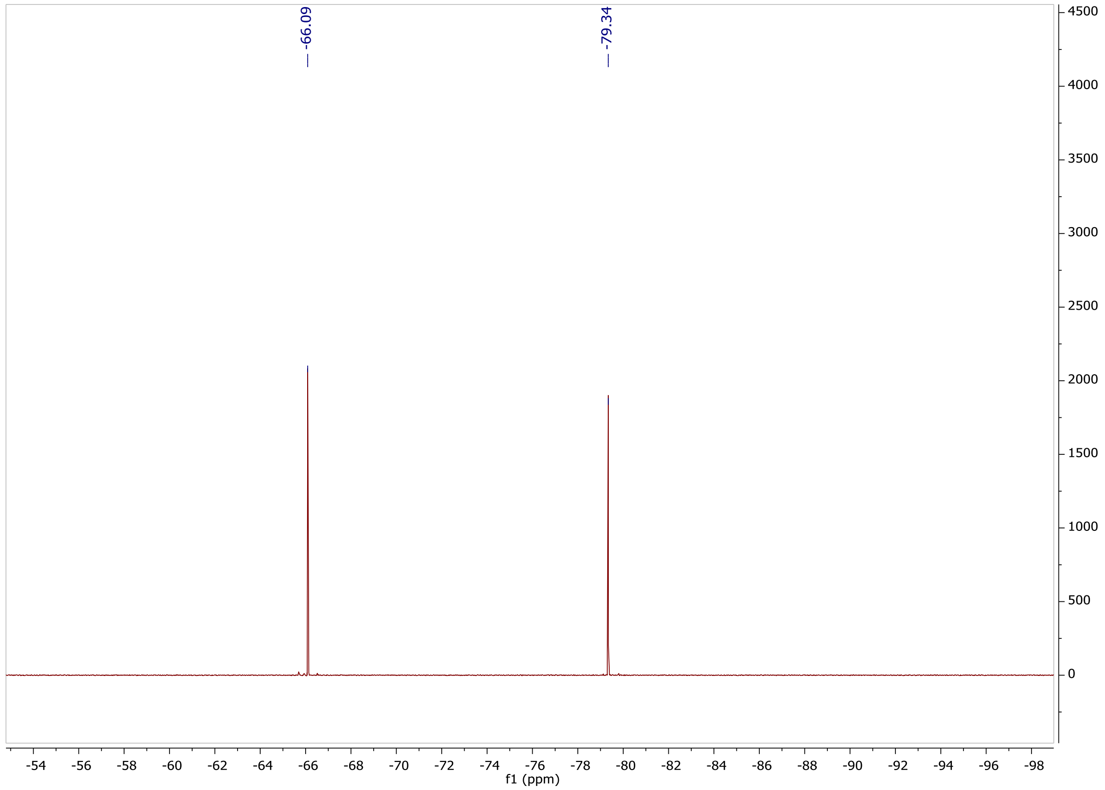

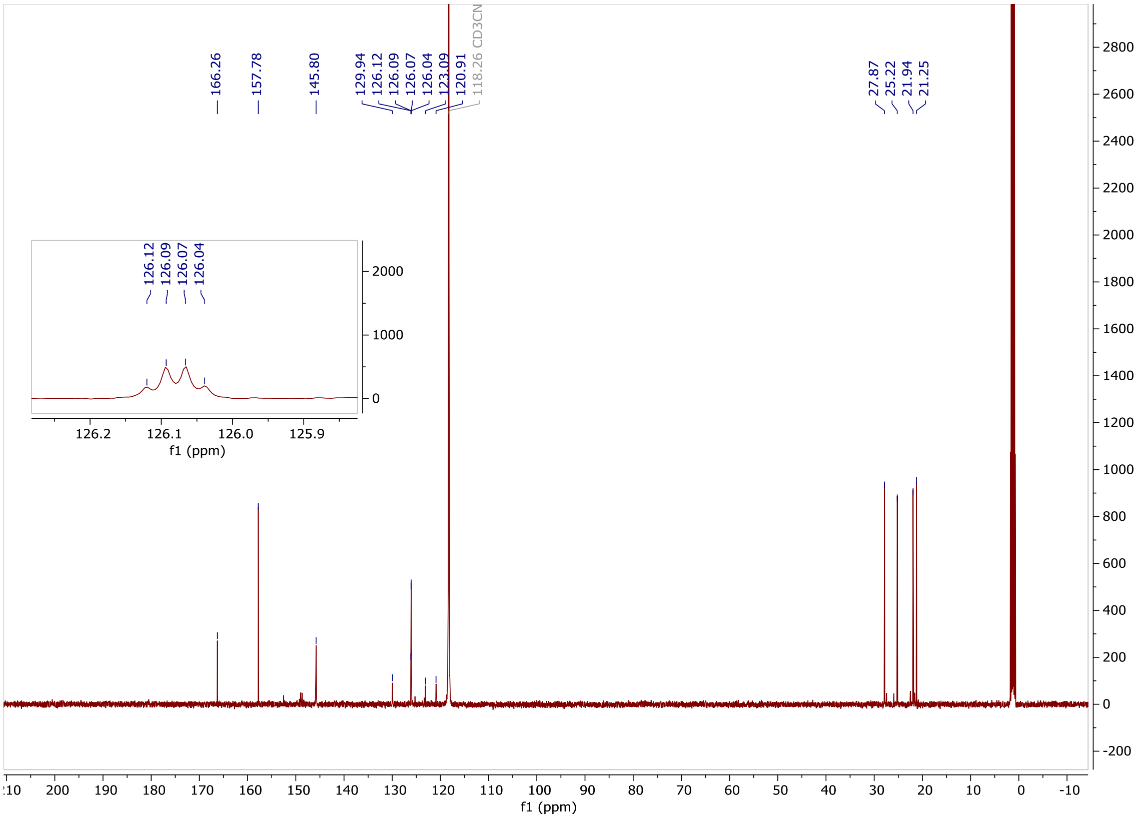
166.26
157.78
145.80
129.94
126.12
126.09
126.07
126.04
123.09
120.91
118.26 CD3CN
27.87
25.22
21.94
21.25
f1 (ppm)

^{1}H, ^{19}F and ^{13}C NMR data for 1c.

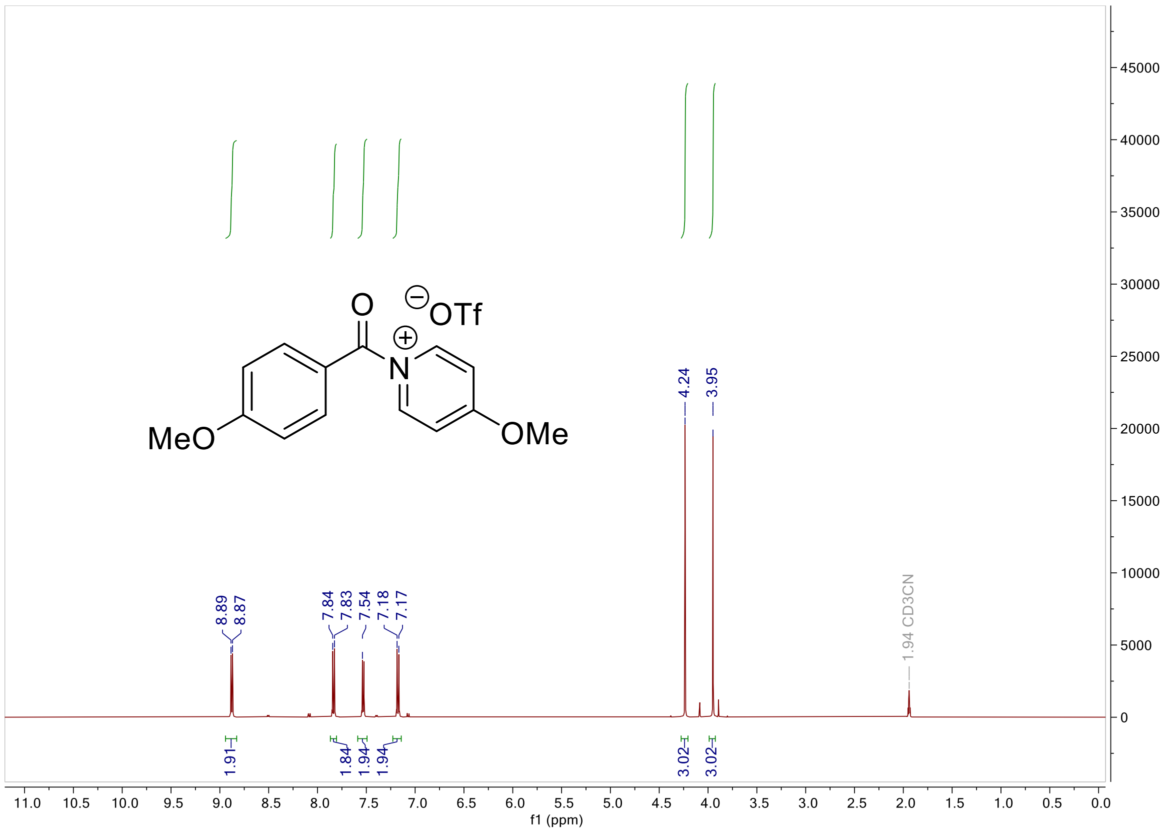

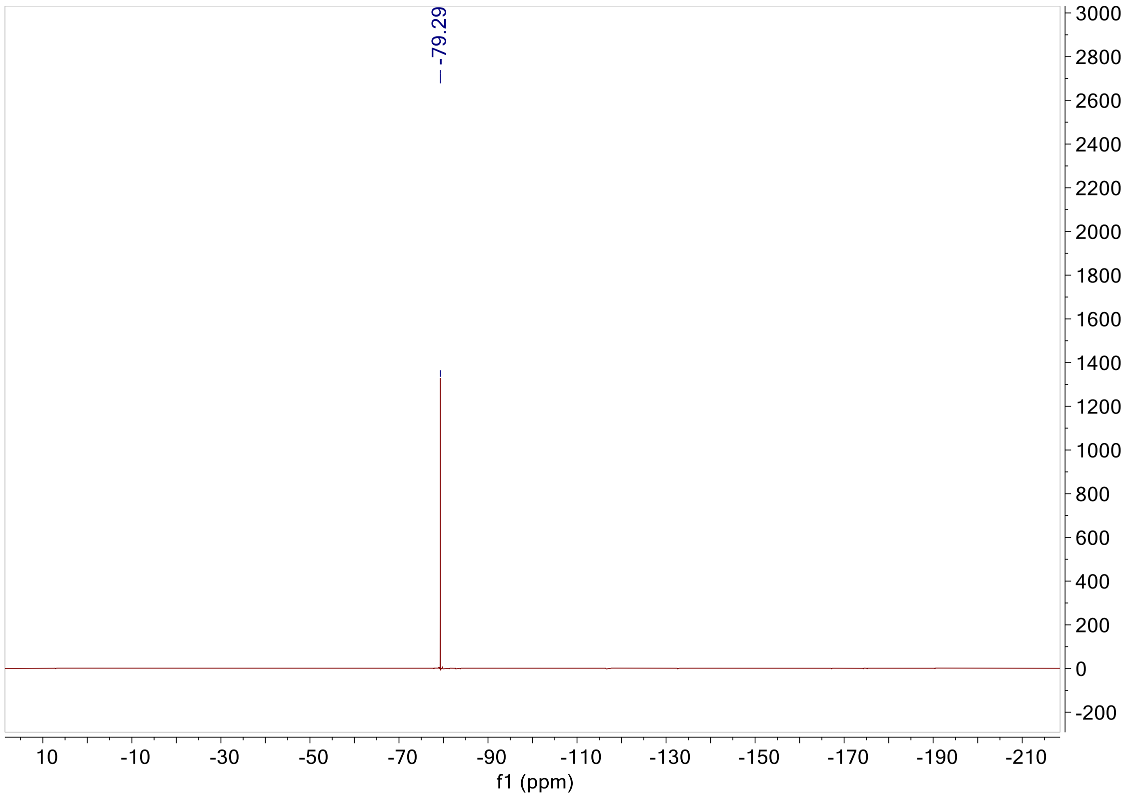

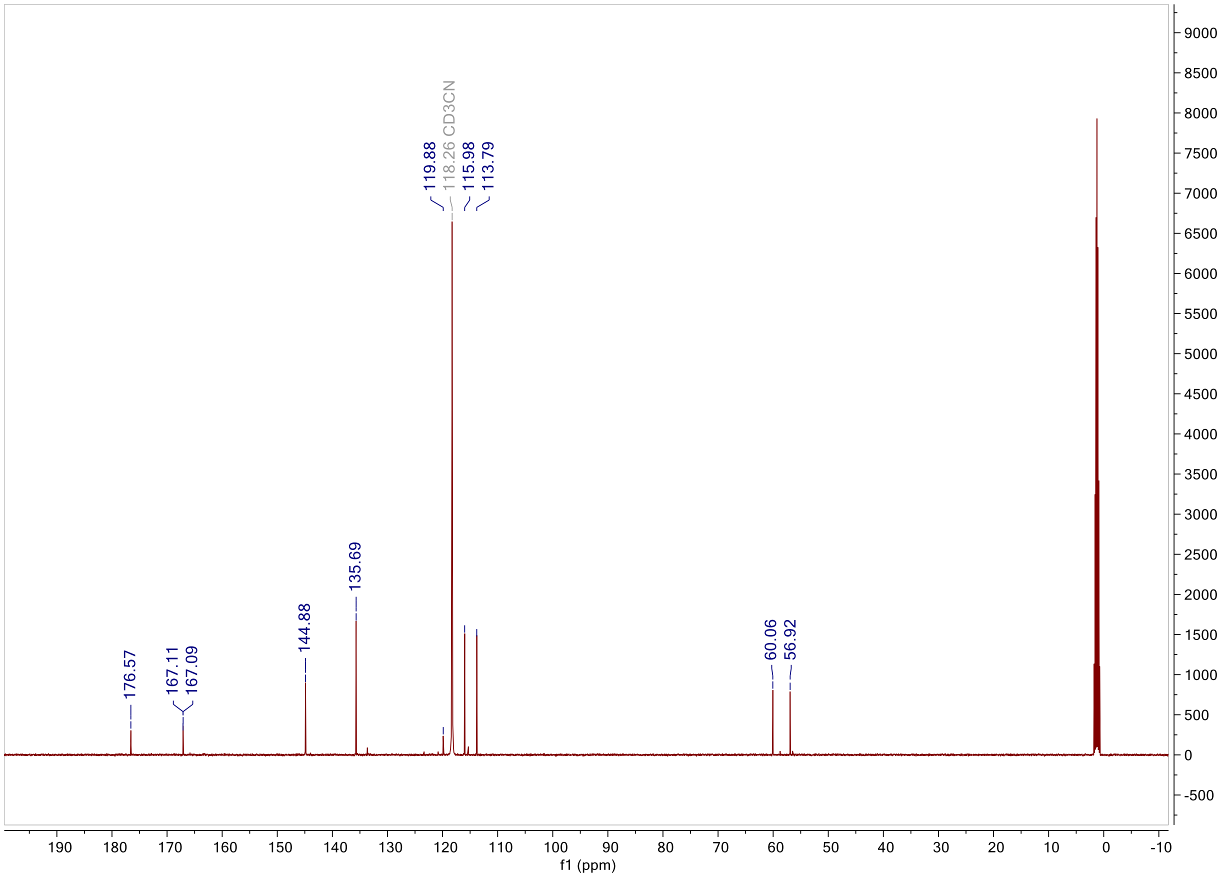
176.57
167.11
167.09
144.88
135.69
119.88
118.26 CD3CN
115.98
113.79
60.06
56.92
f1 (ppm)

^{1}H, ^{19}F and ^{13}C NMR data for 1d.

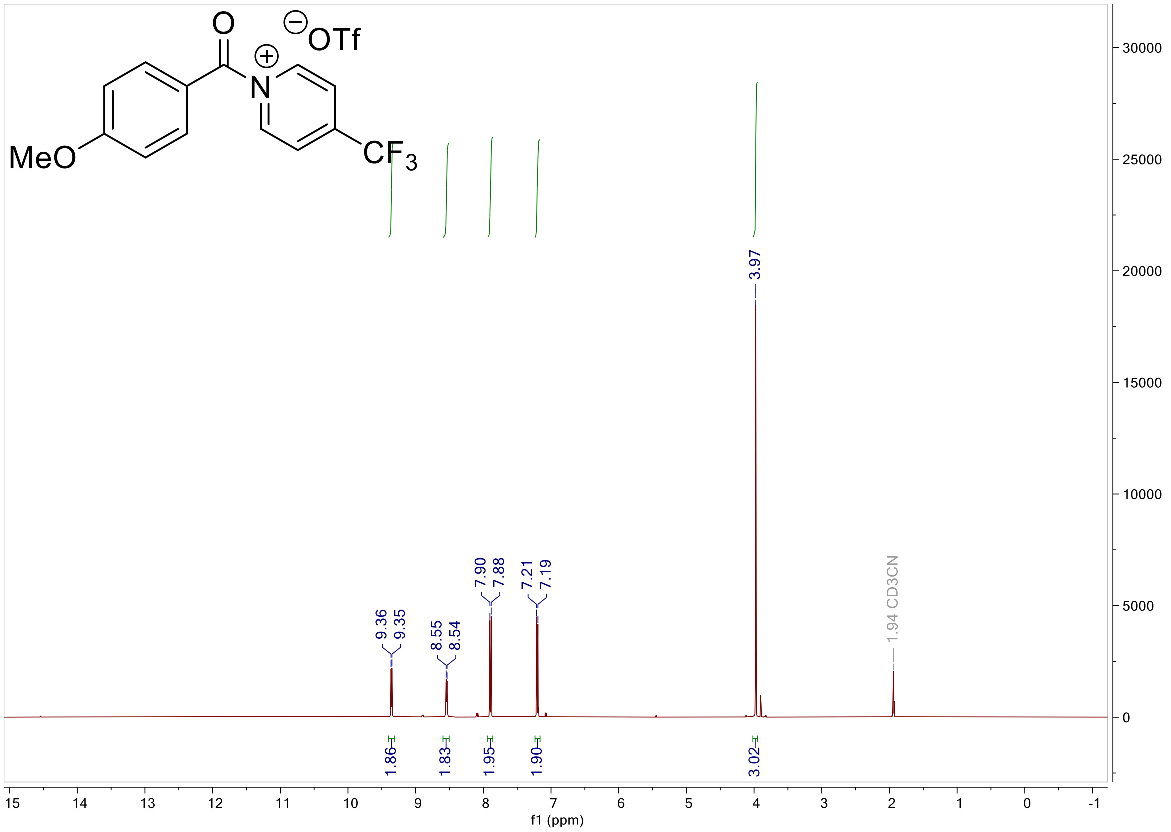

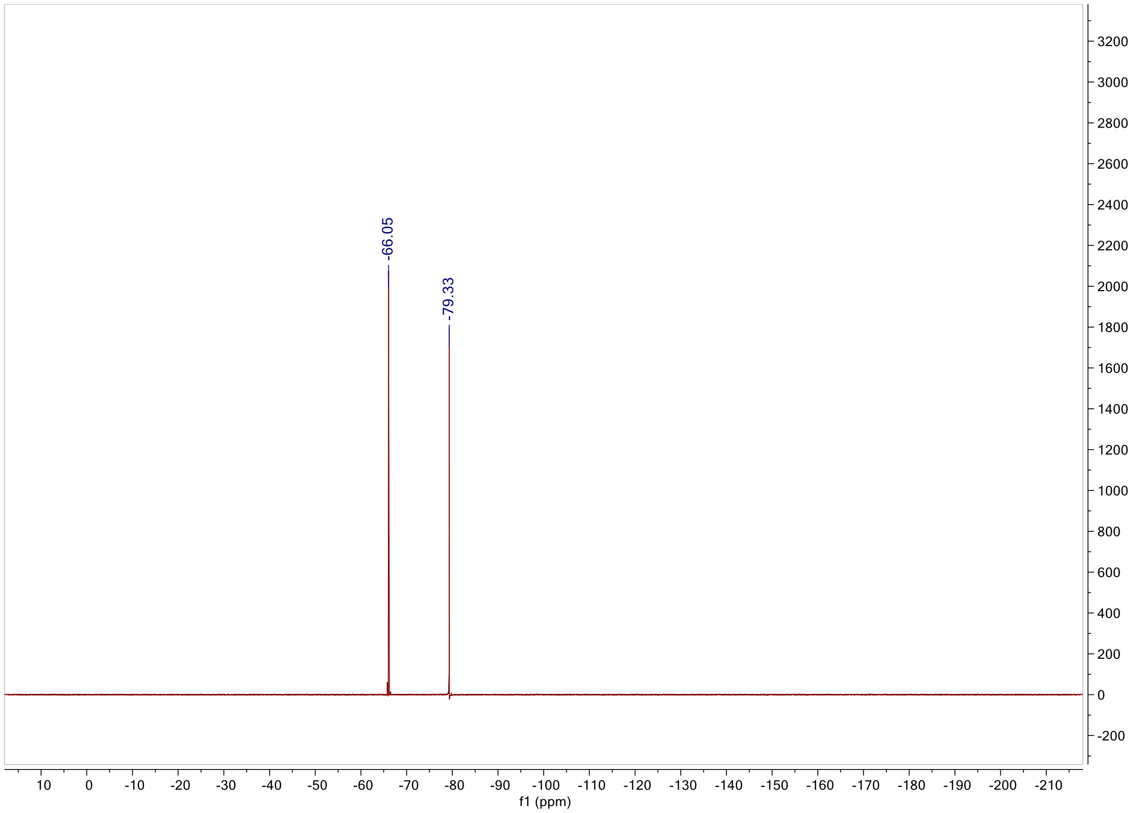

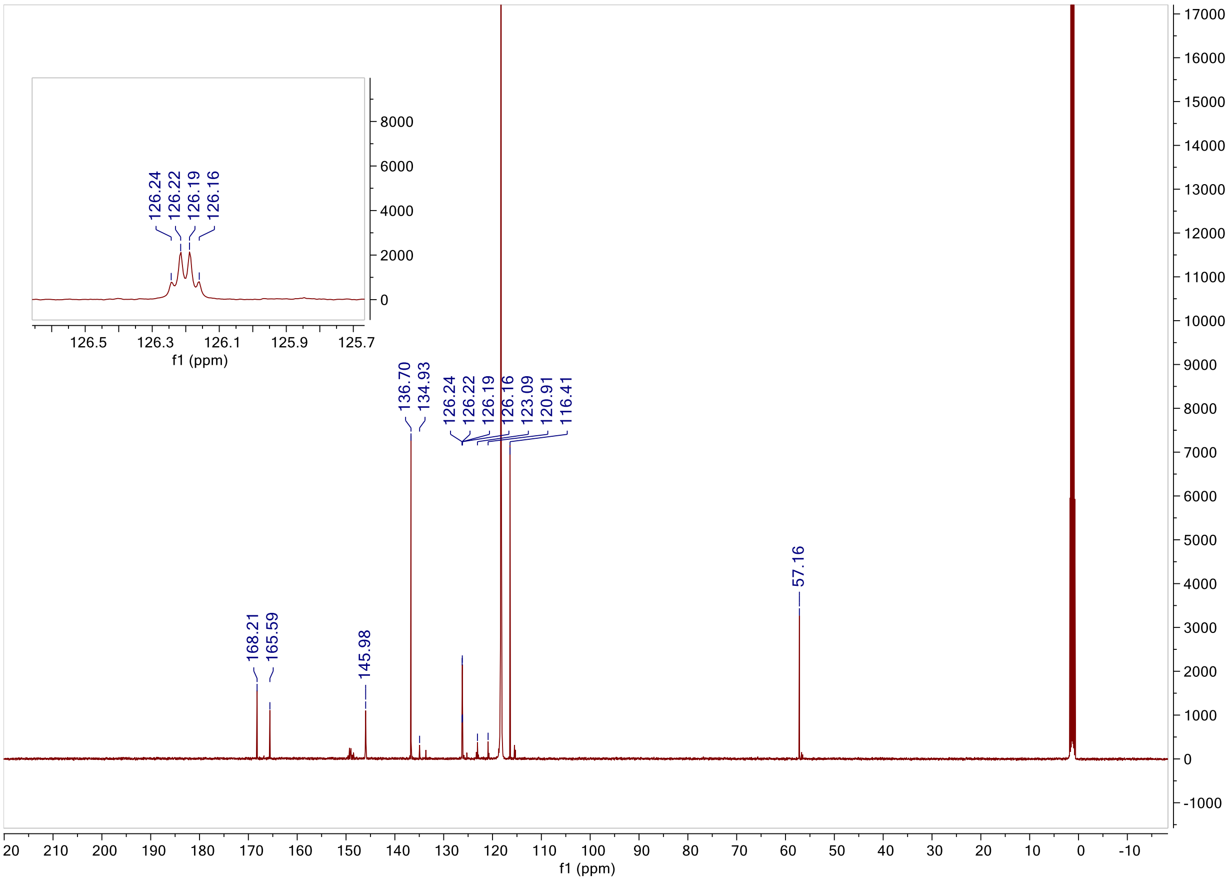
126.24
126.22
126.19
126.16
8000
6000
4000
2000
0
126.5
126.3
126.1
125.9
125.7
f1 (ppm)
136.70
134.93
126.24
126.22
126.19
126.16
123.09
120.91
116.41
57.16
168.21
165.59
145.98
17000
16000
15000
14000
13000
12000
11000
10000
9000
8000
7000
6000
5000
4000
3000
2000
1000
0
-1000
20
210
200
190
180
170
160
150
140
130
120
110
100
90
80
70
60
50
40
30
20
10
0
-10
f1 (ppm)

^{1}H, ^{19}F and ^{13}C NMR data for 1e.

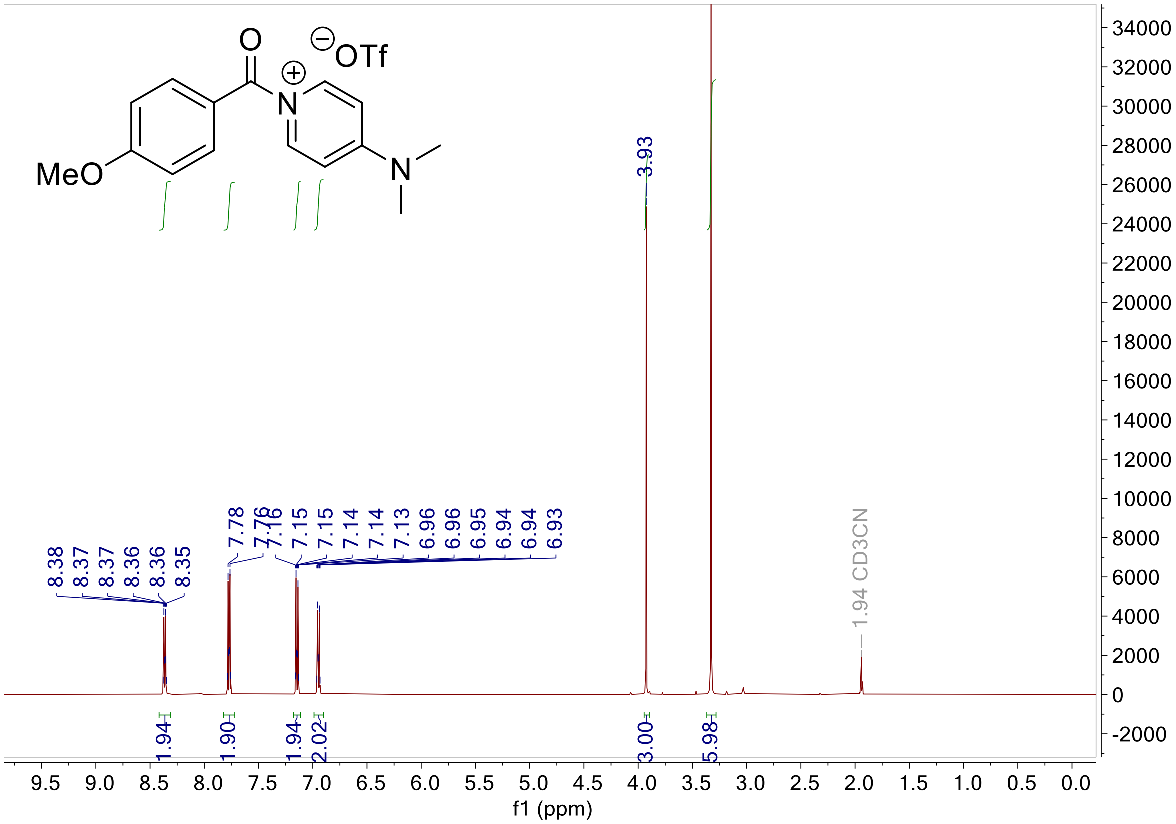

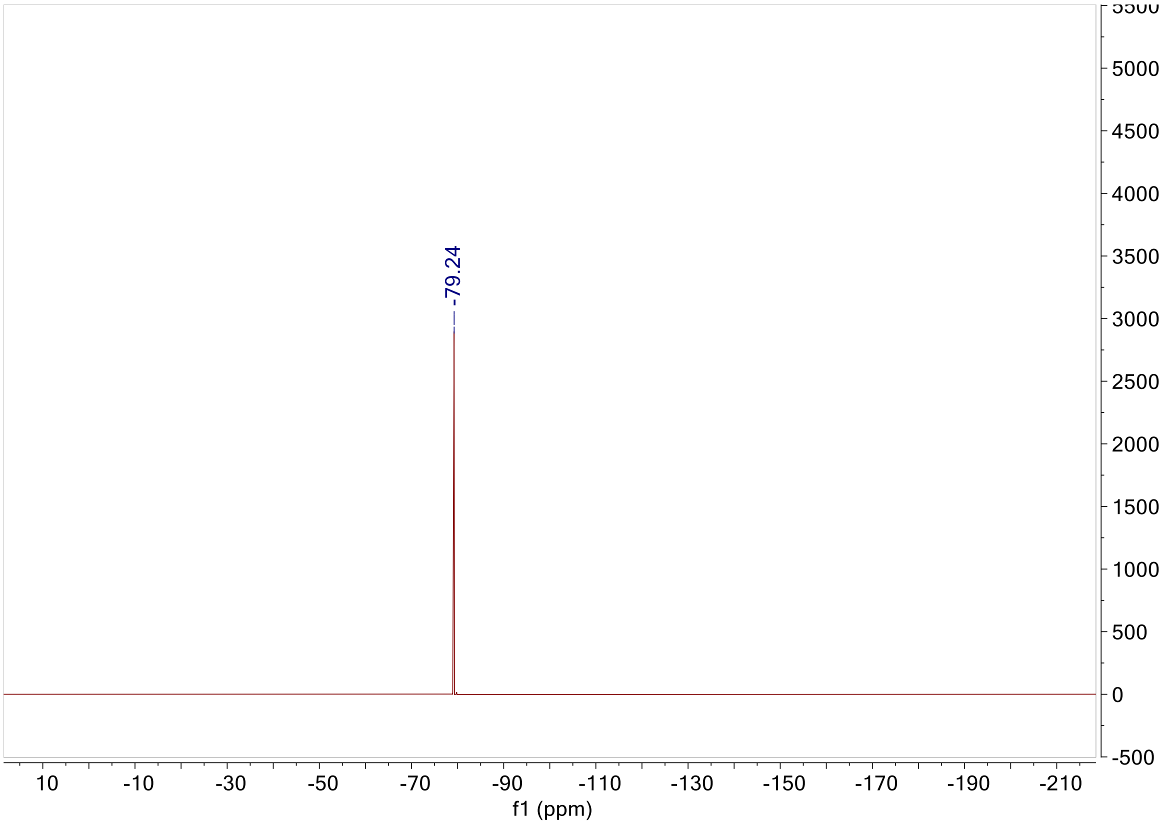

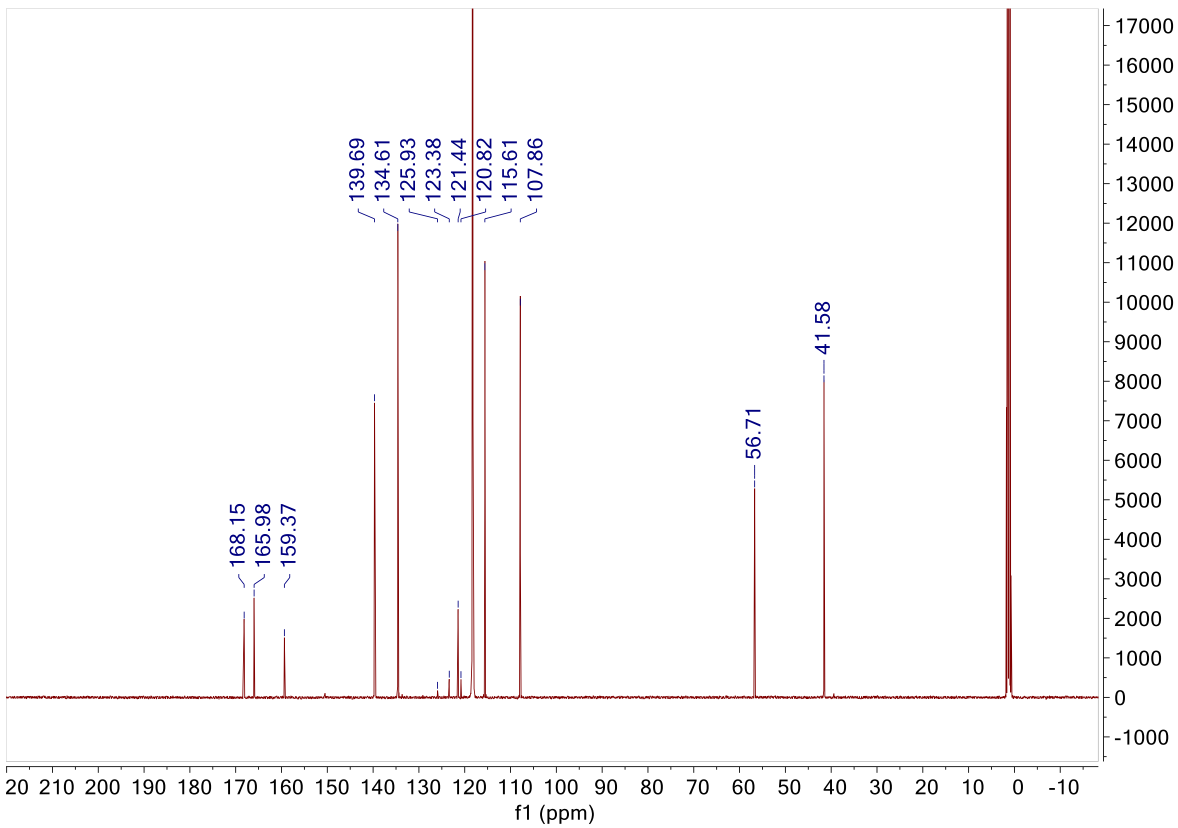

168.15
165.98
159.37
139.69
134.61
125.93
123.38
121.44
120.82
115.61
107.86
56.71
41.58
17000
16000
15000
14000
13000
12000
11000
10000
9000
8000
7000
6000
5000
4000
3000
2000
1000
0
-1000
20 210 200 190 180 170 160 150 140 130 120 110 100 90 80 70 60 50 40 30 20 10 0 -10
f1 (ppm)

^{1}H and ^{13}C NMR data for 2-2a.

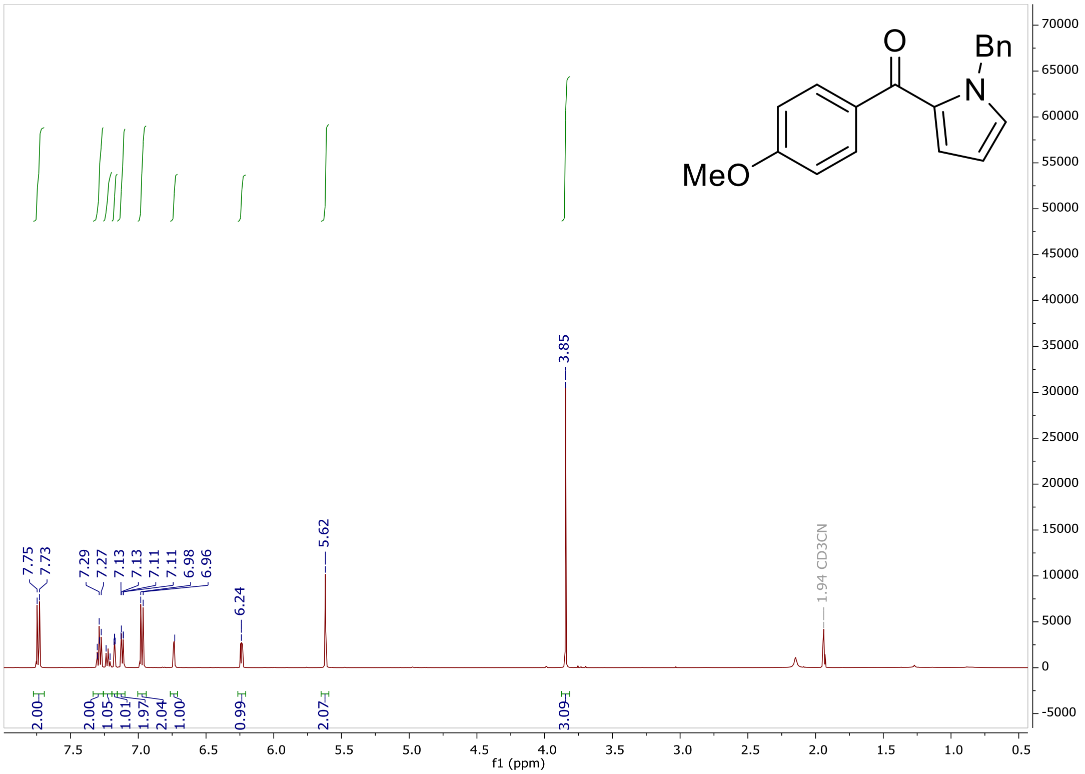

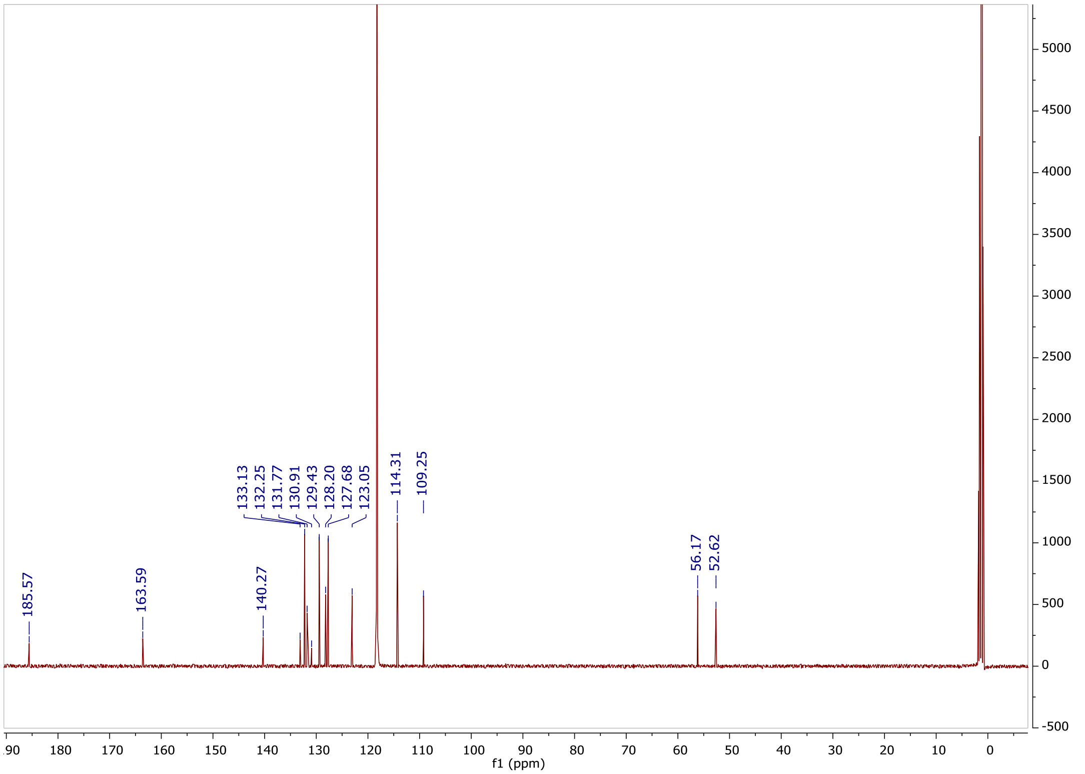

^{1}H and ^{13}C NMR data for 3-2a.

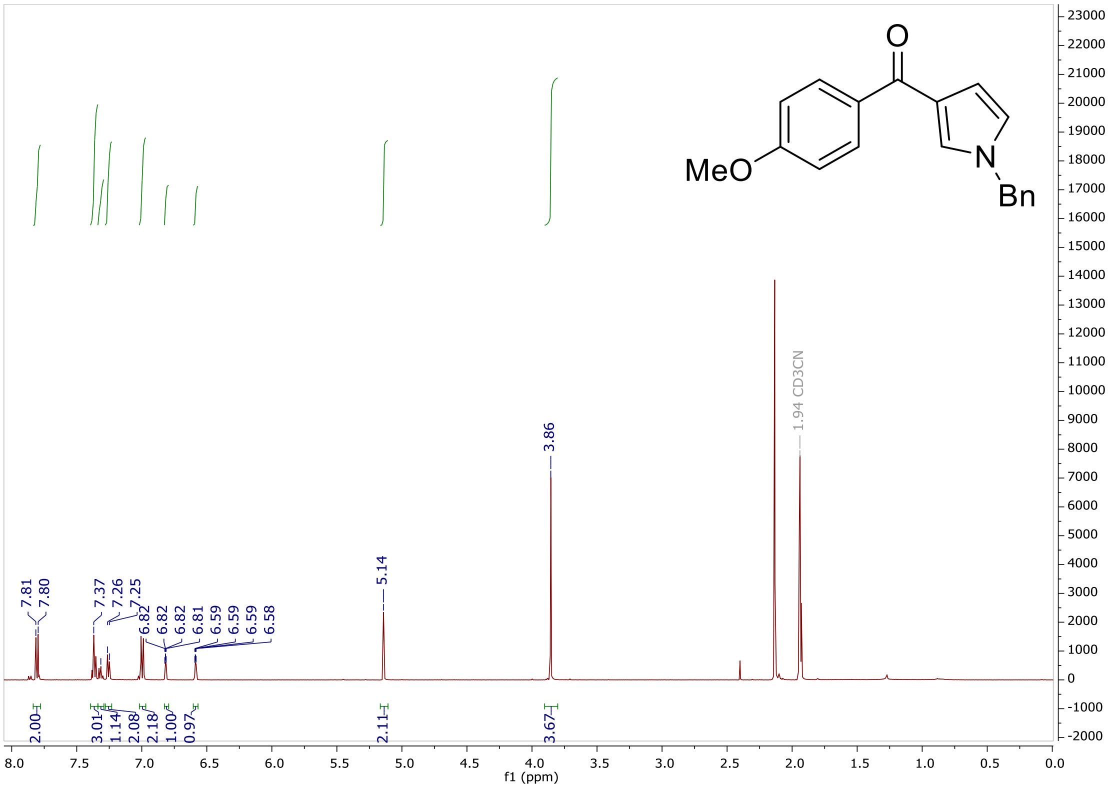

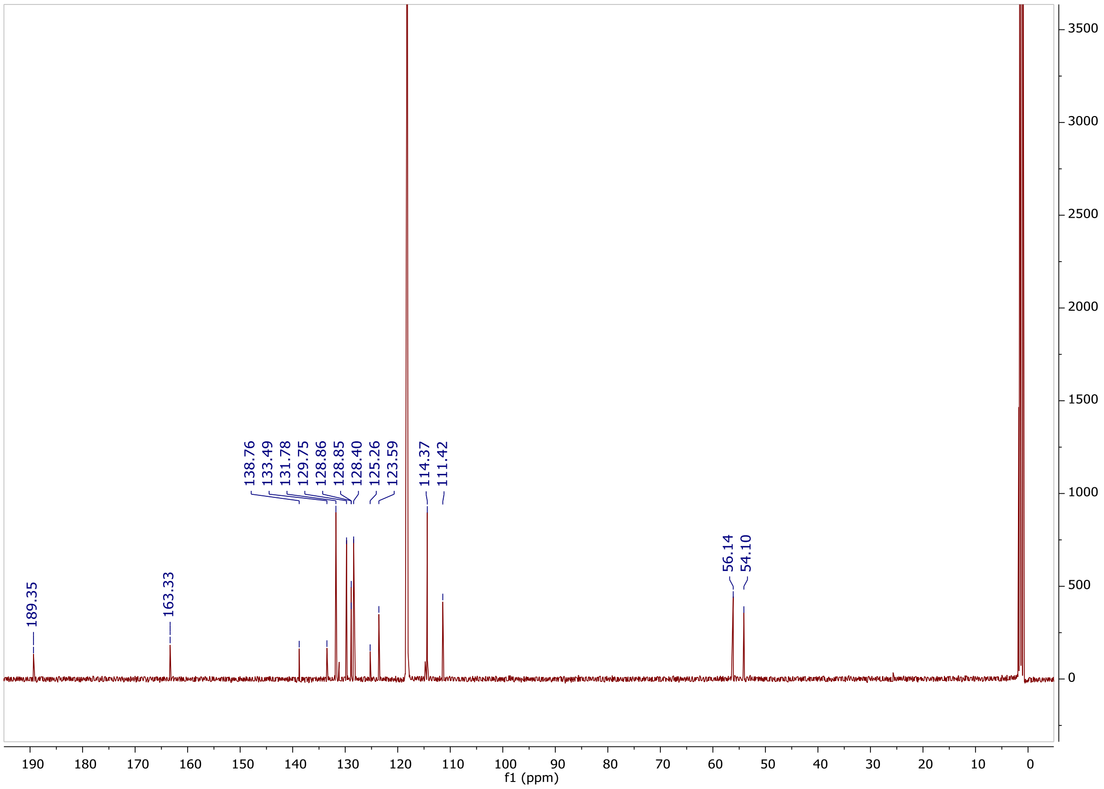

^{1}H and ^{13}C NMR data for 2-2b.

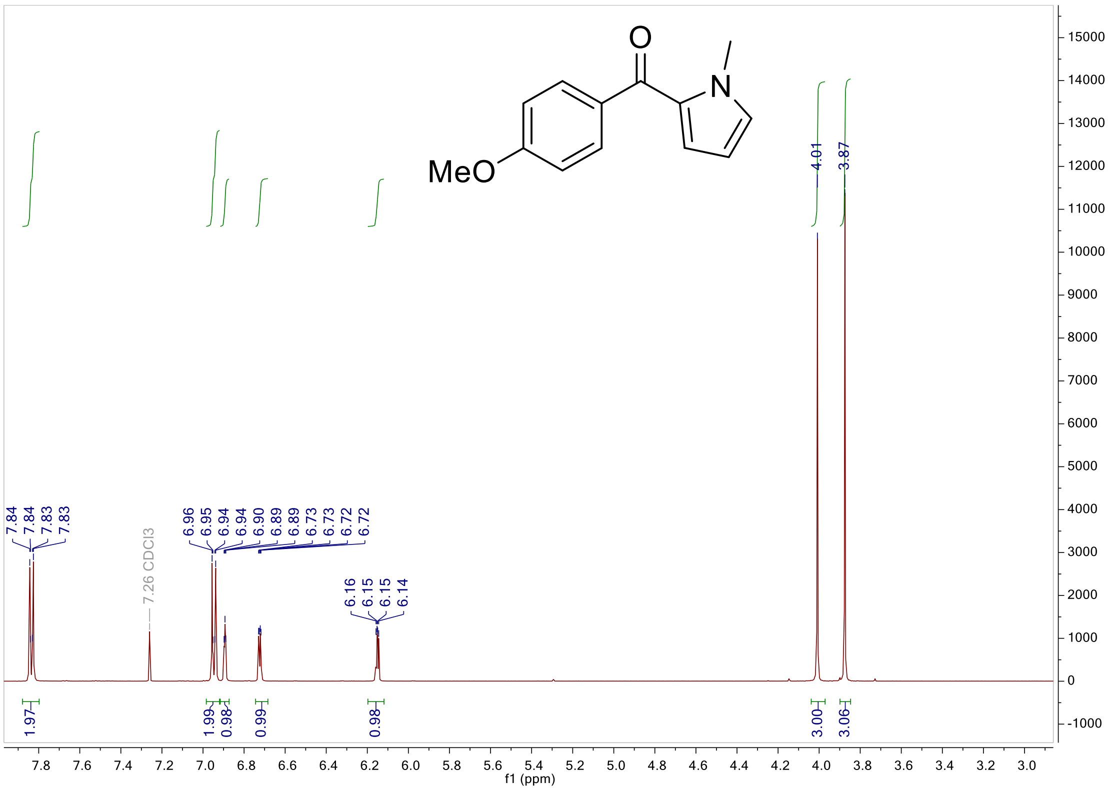

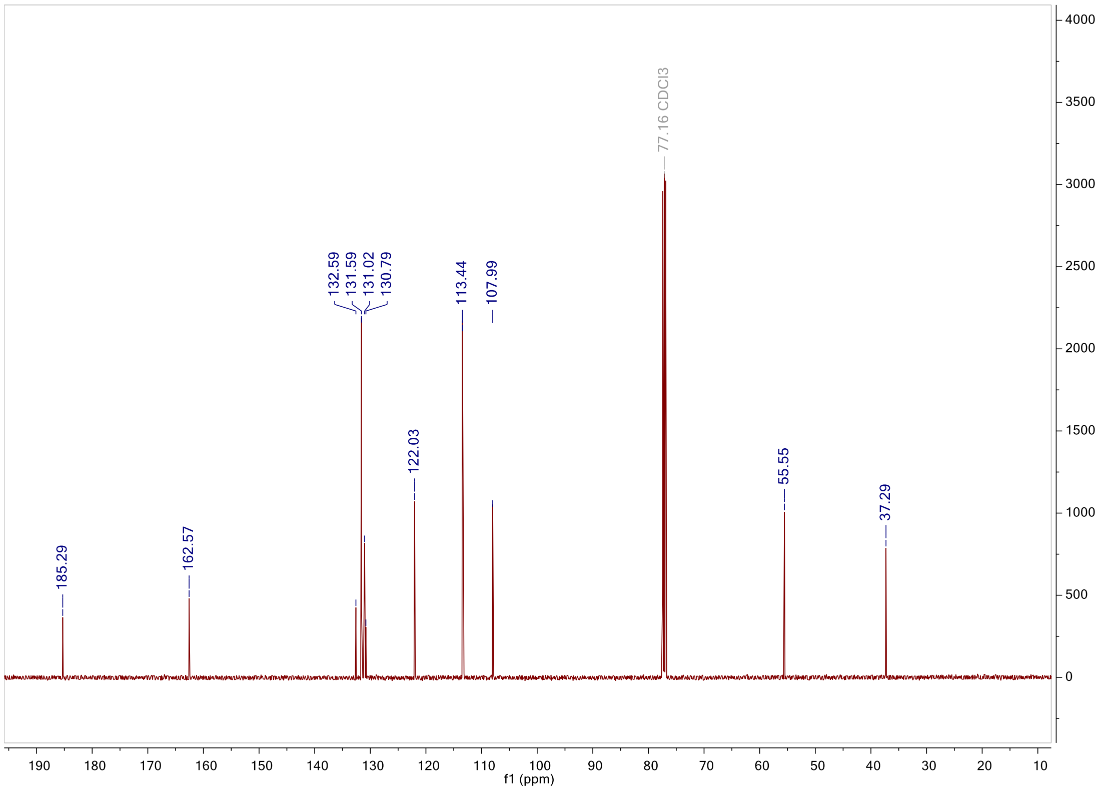

^{1}H and ^{13}C NMR data for 3-2b

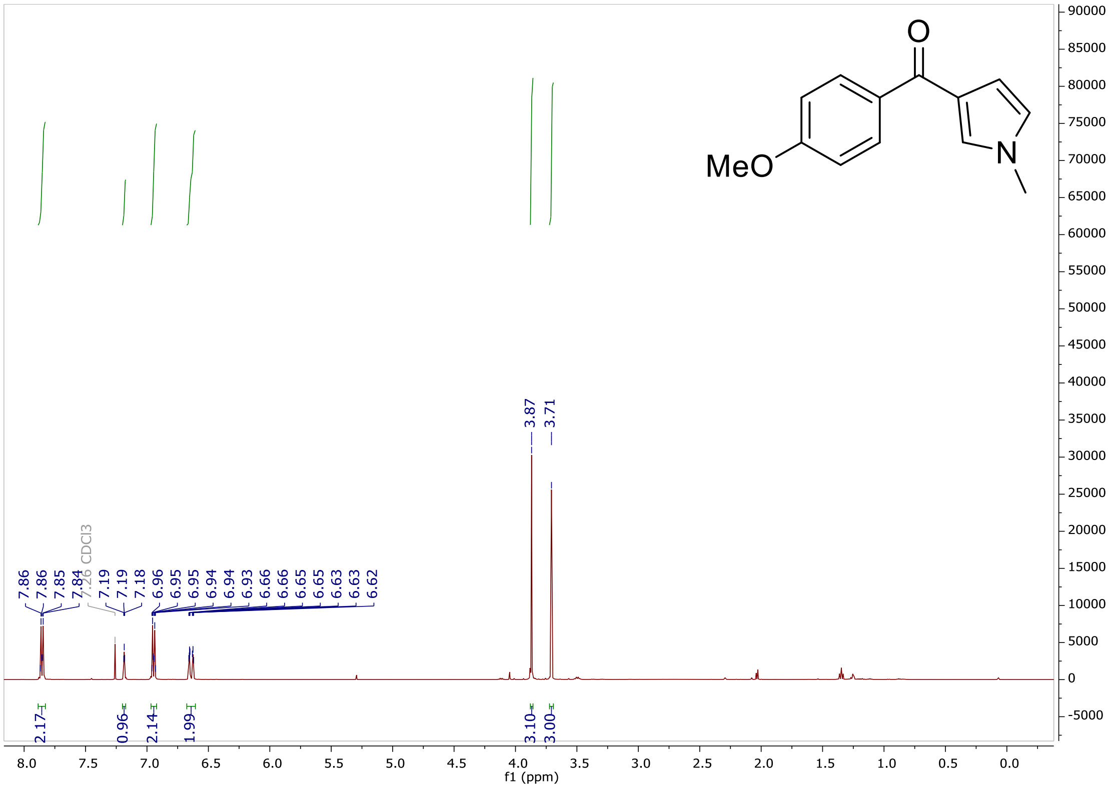

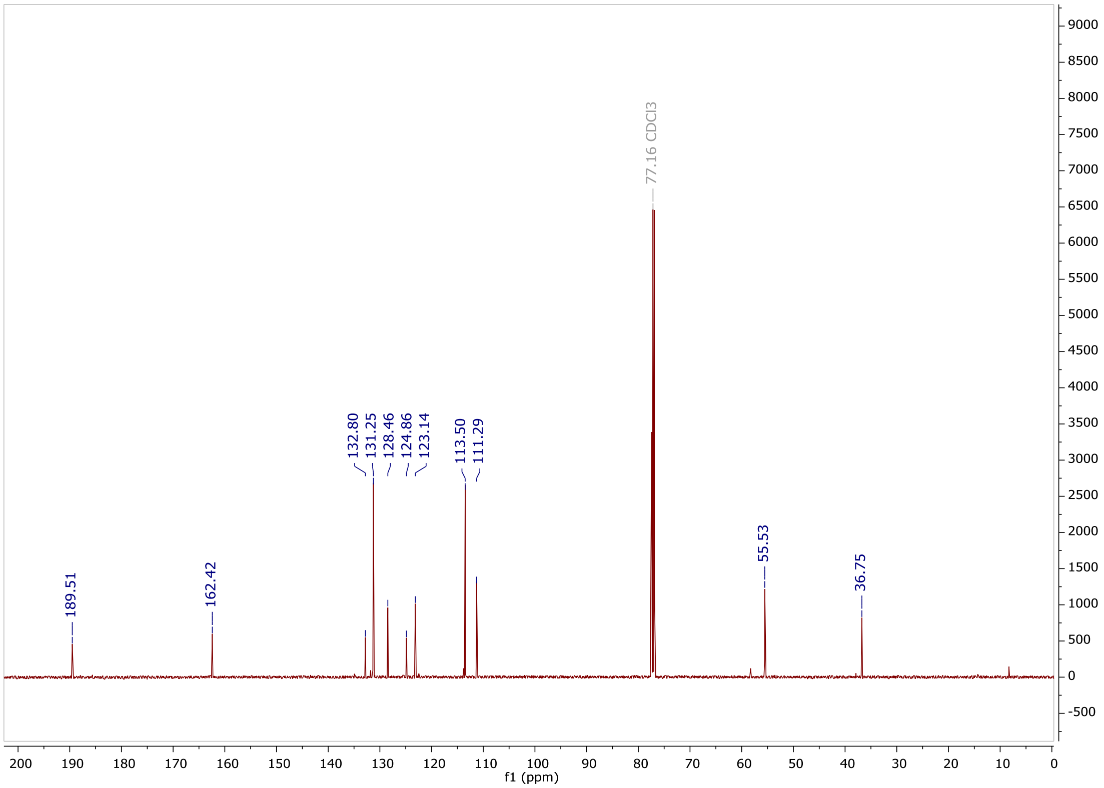

^{1}H and ^{13}C NMR data for 2-2c.

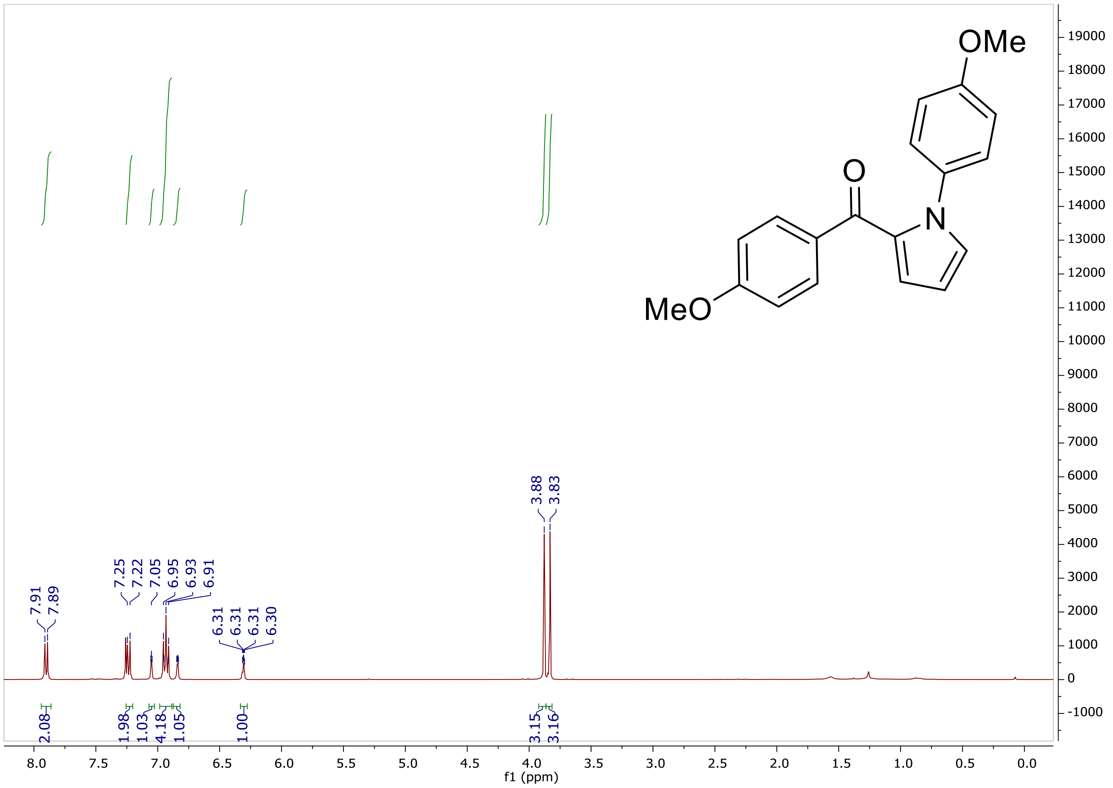

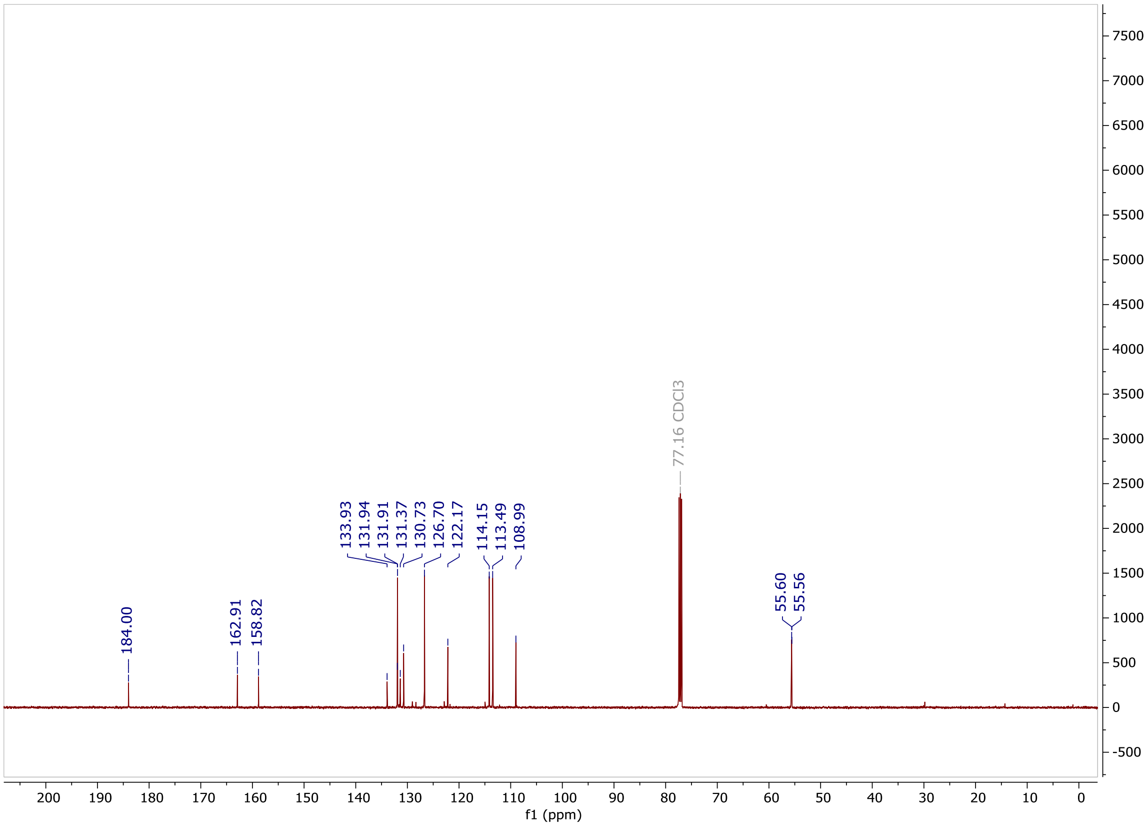

NOESY data for 2-2c (CDCl₃):

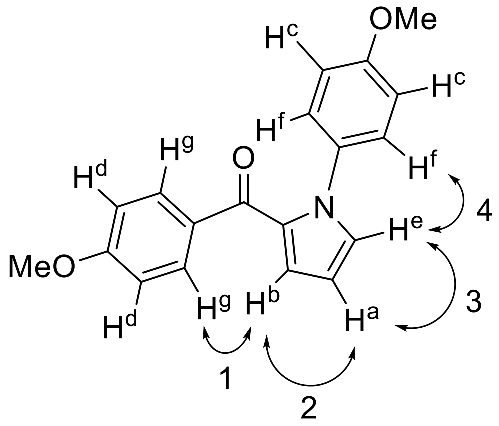

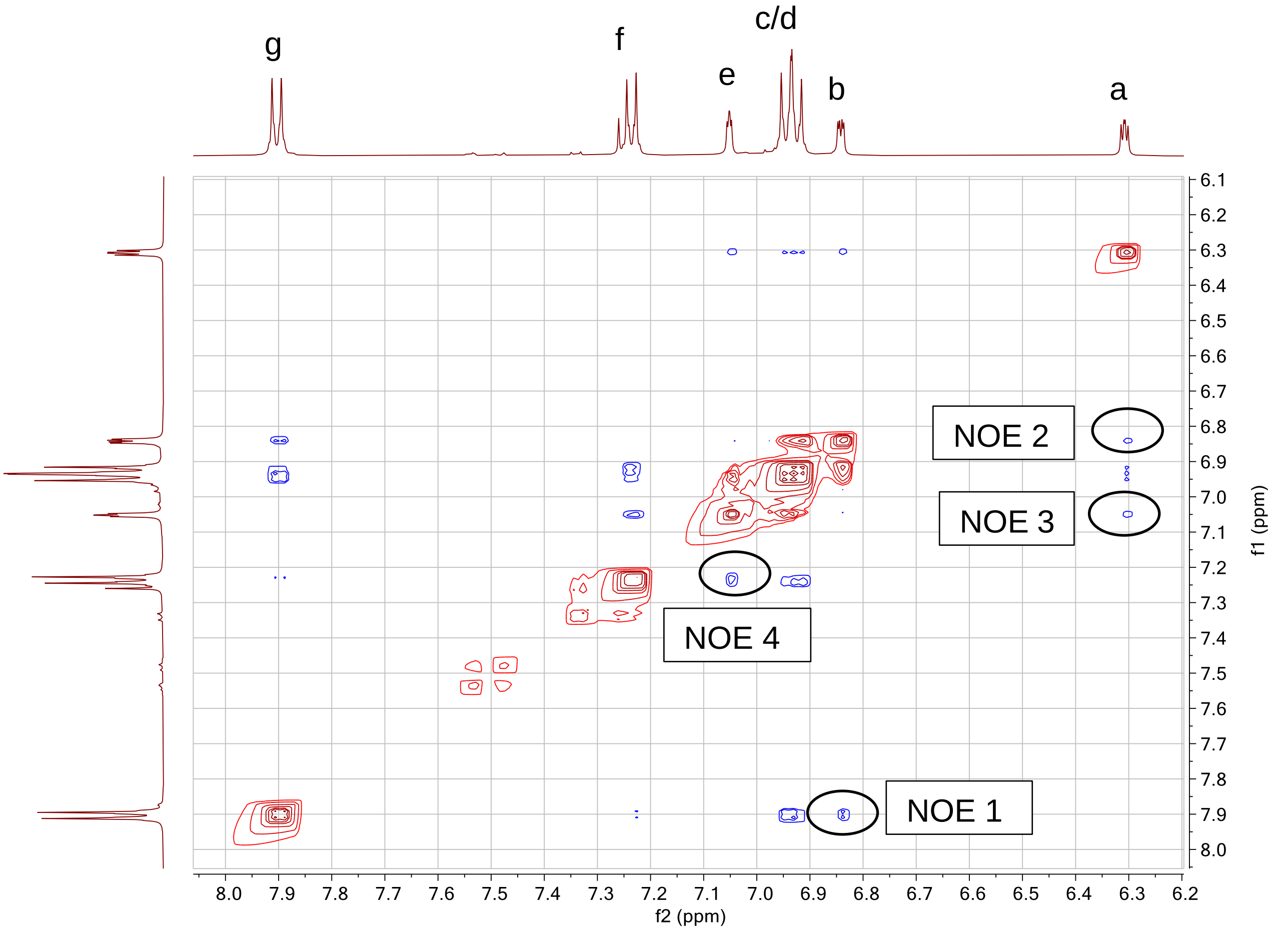

^{1}H and ^{13}C NMR data for 3-2c.

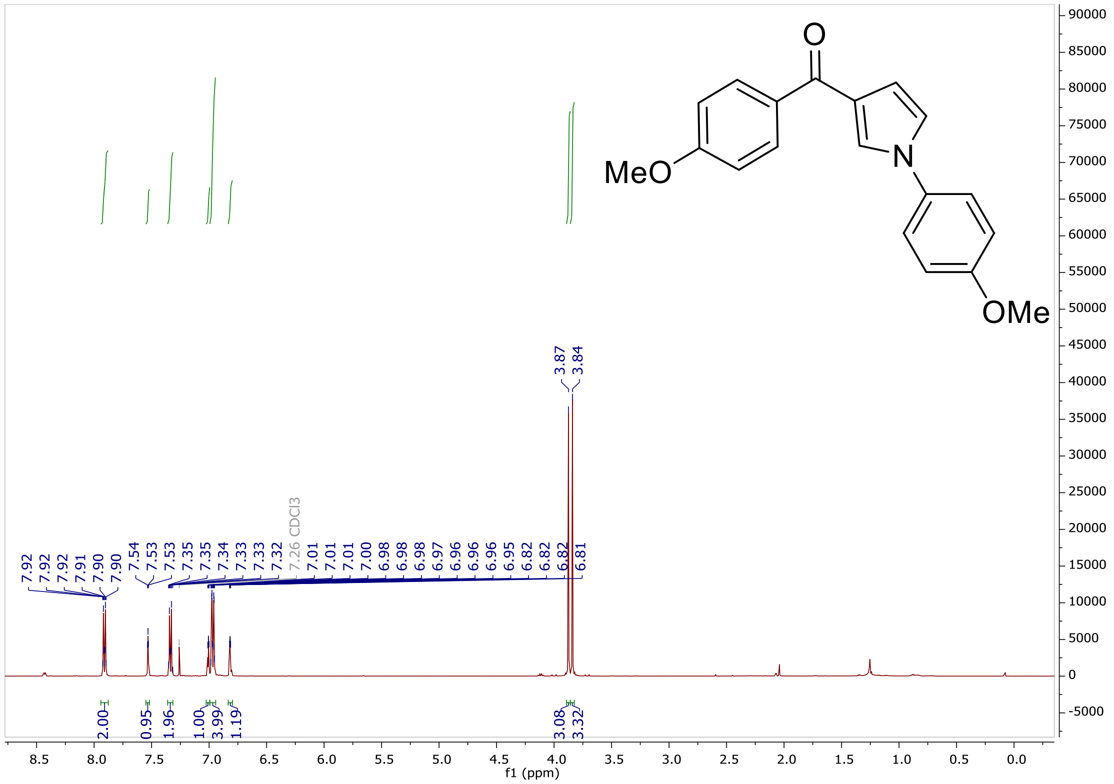

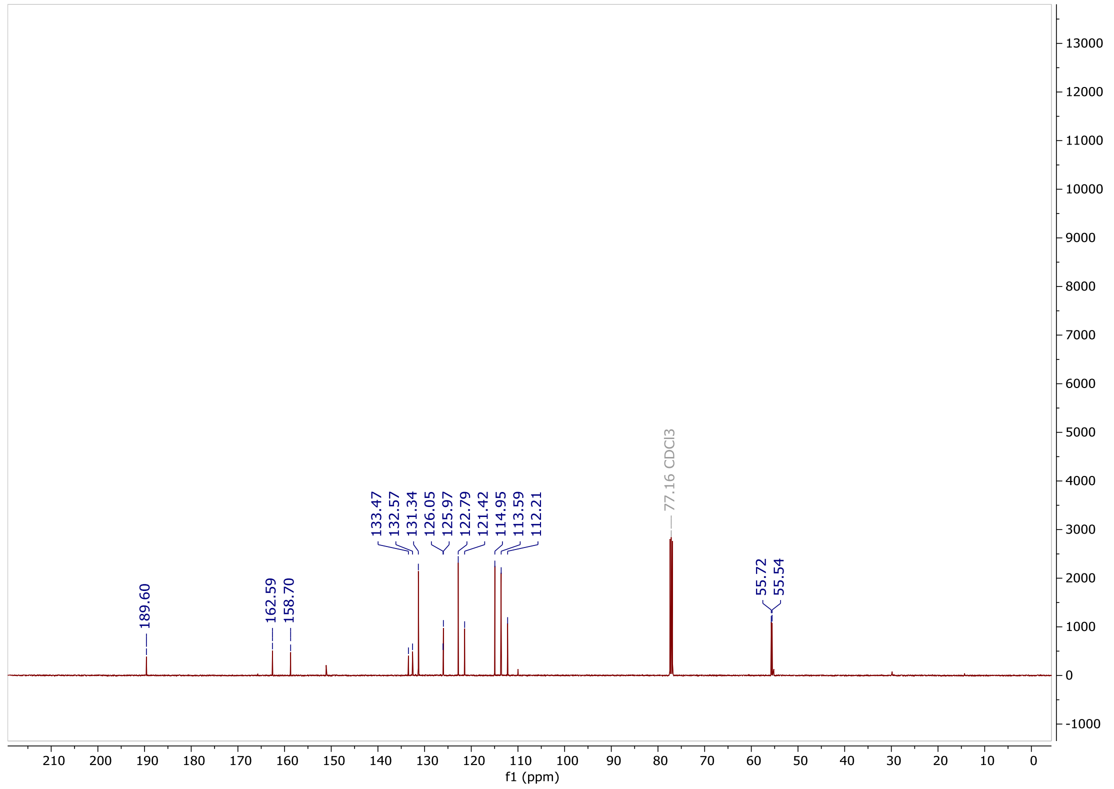

NOESY data for 3-2c.

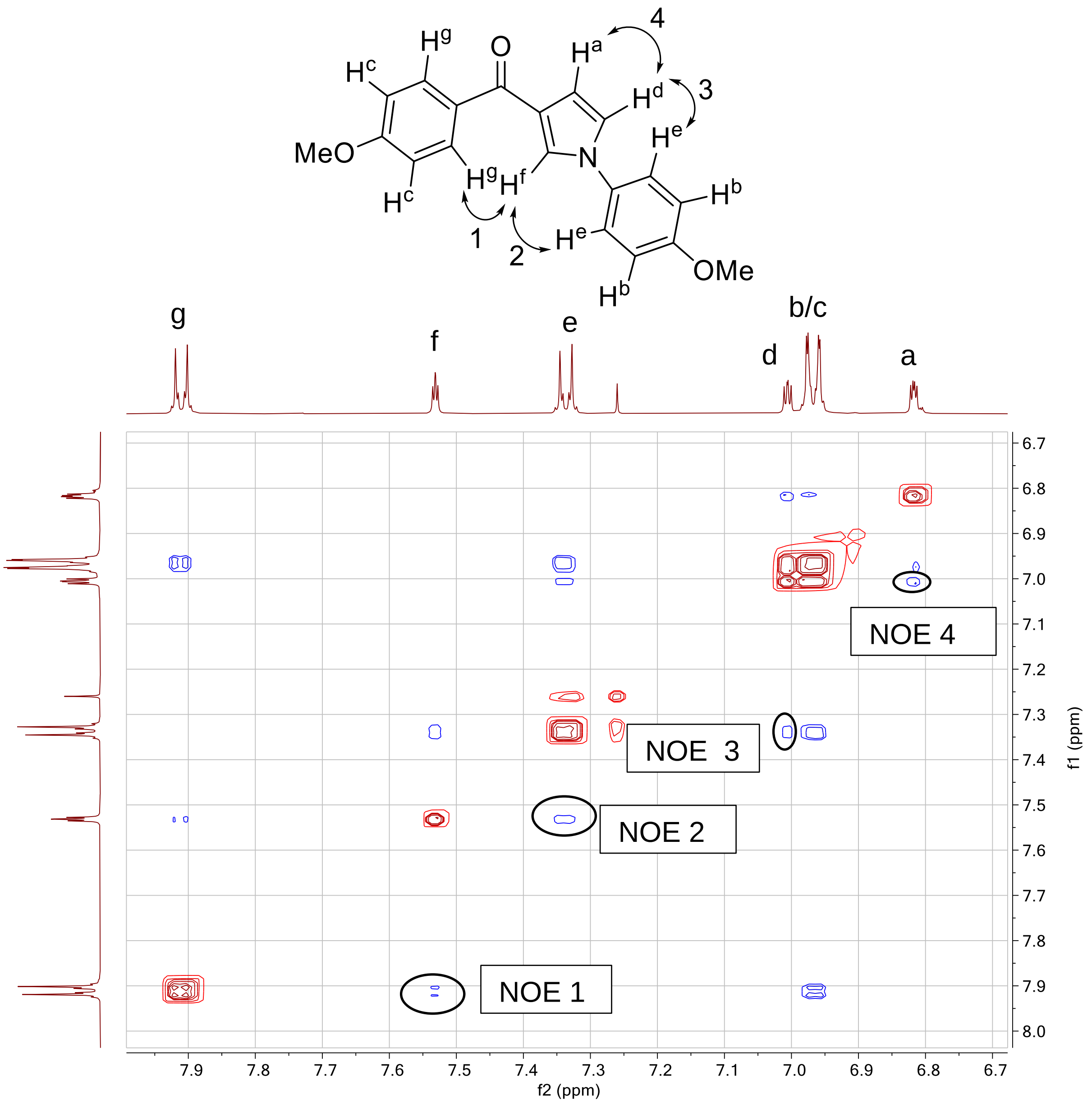

^{1}H and ^{13}C NMR data for 2d.

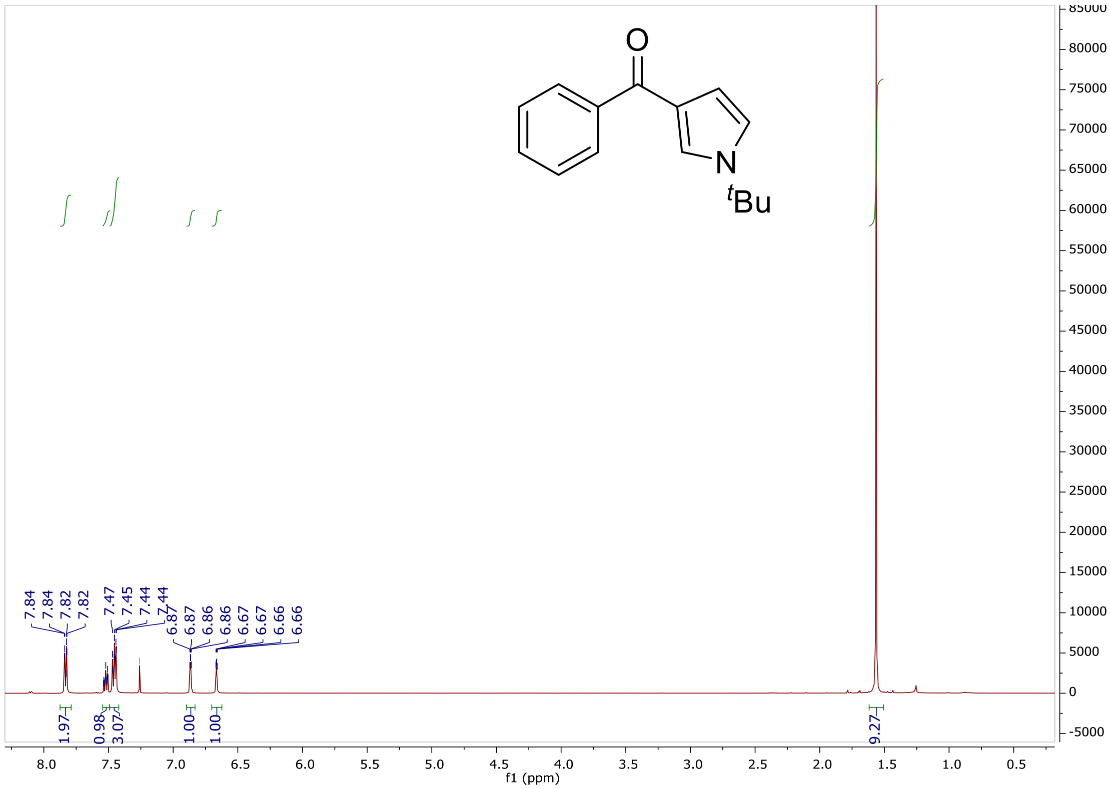

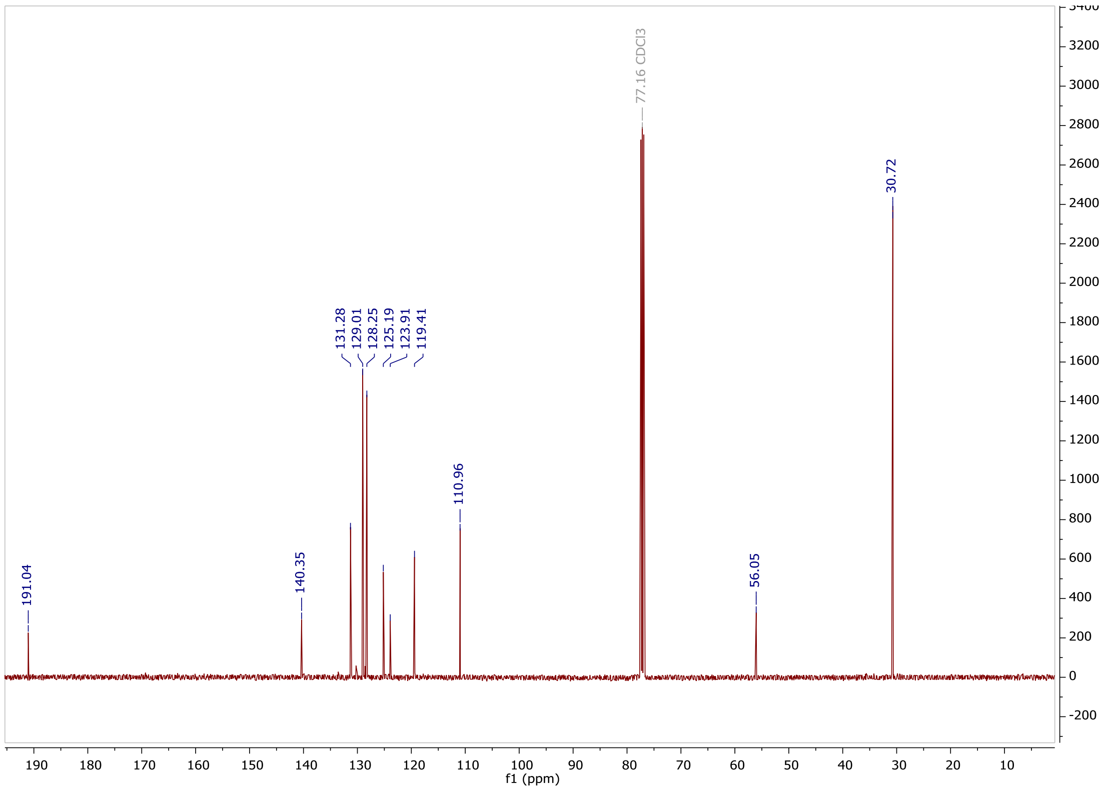

^{1}H and ^{13}C NMR data for 3-2e.

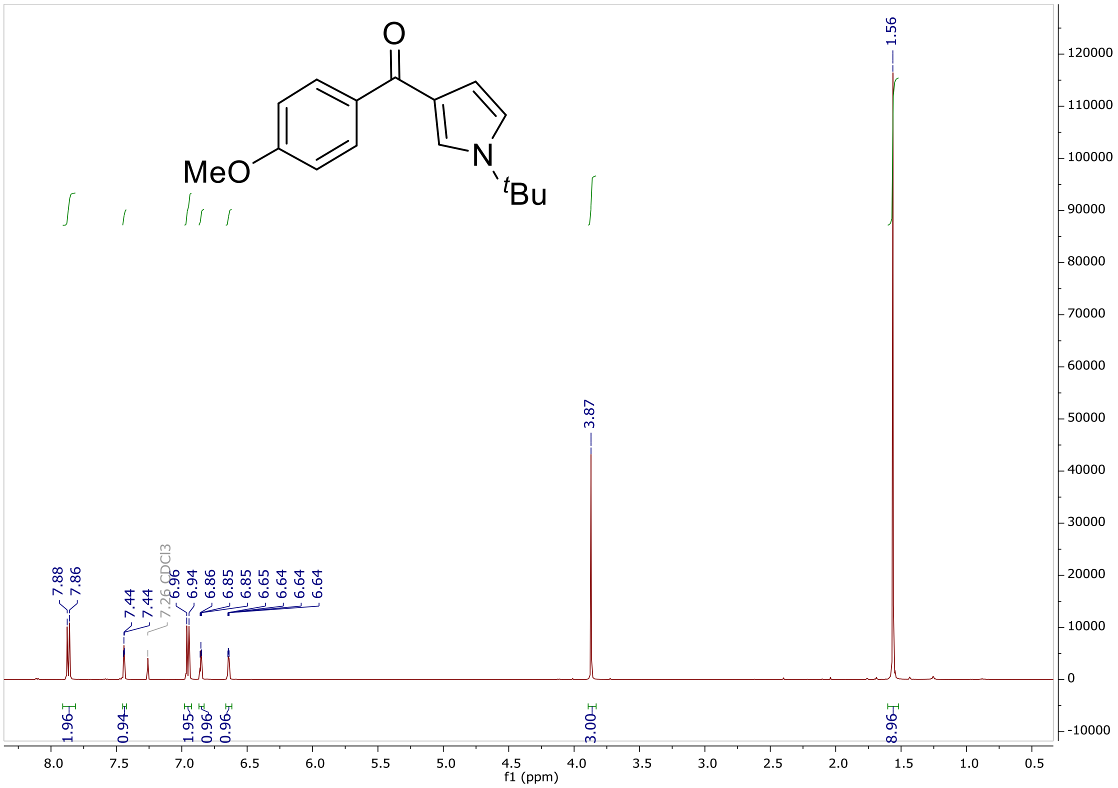

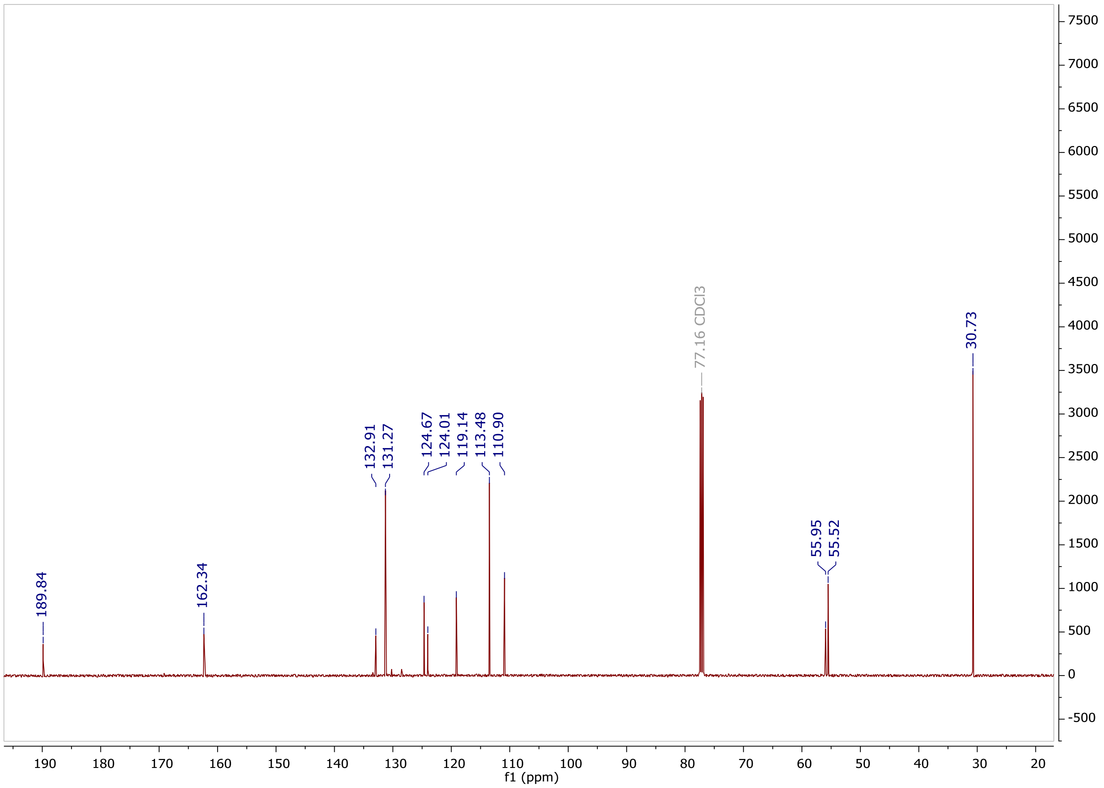

^{1}H and ^{13}C NMR data for 2-2e.

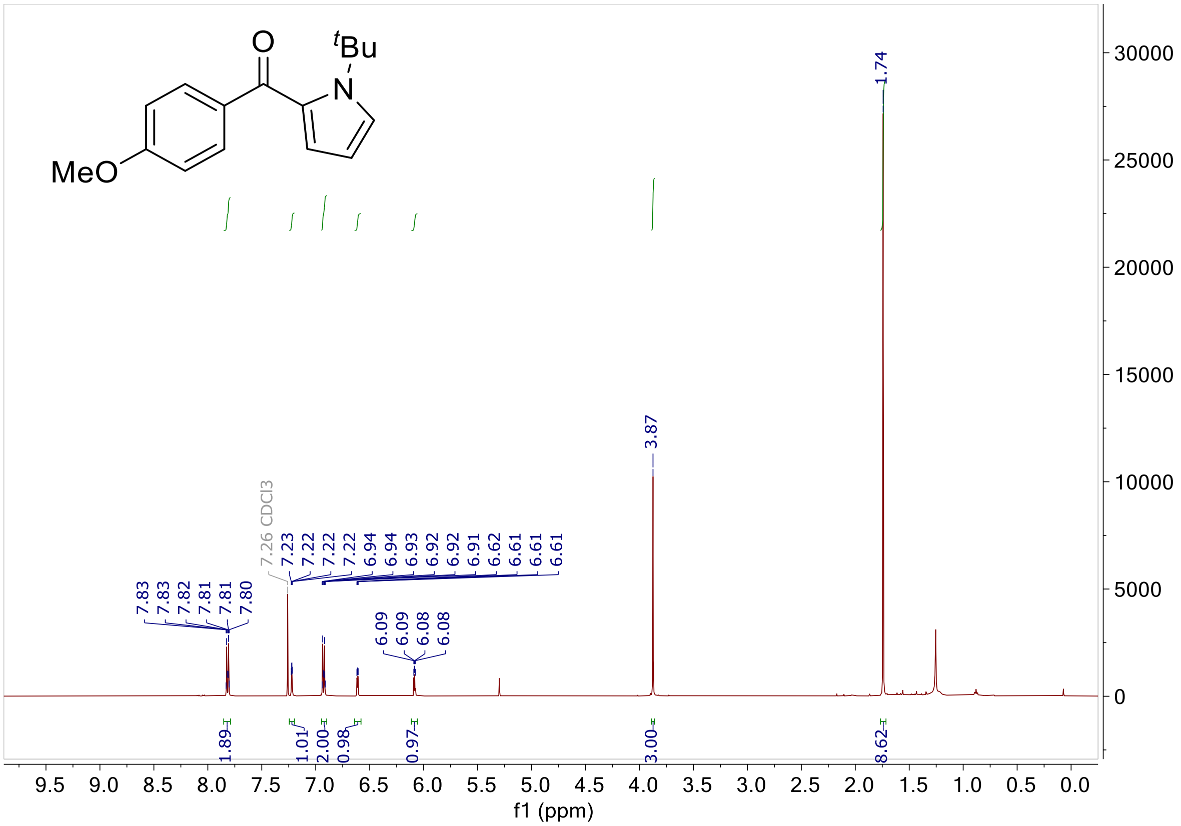

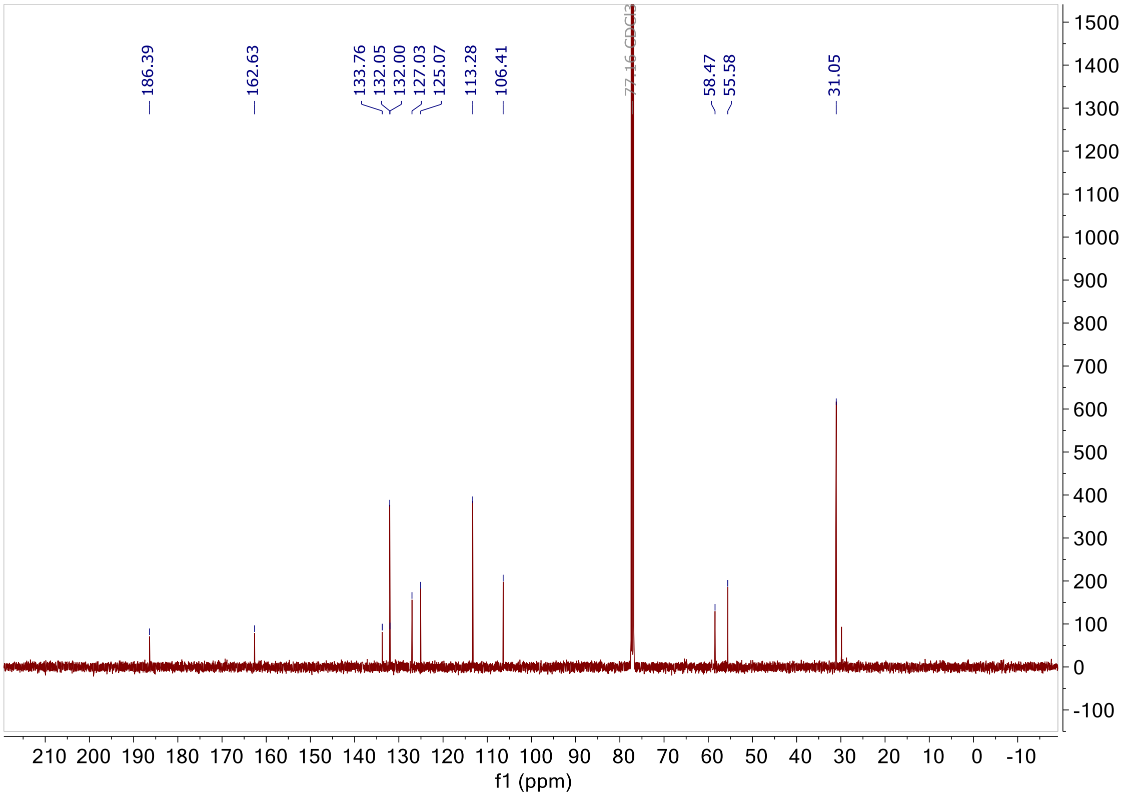

^{1}H and ^{13}C NMR data for 2f.

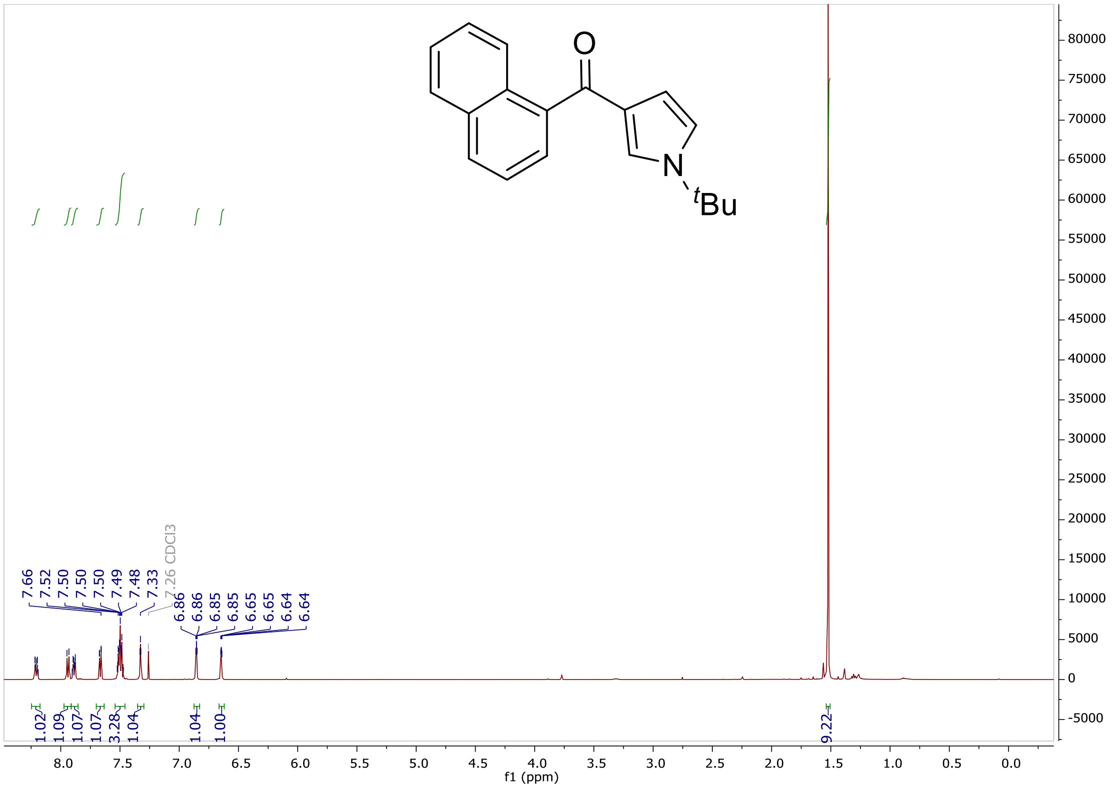

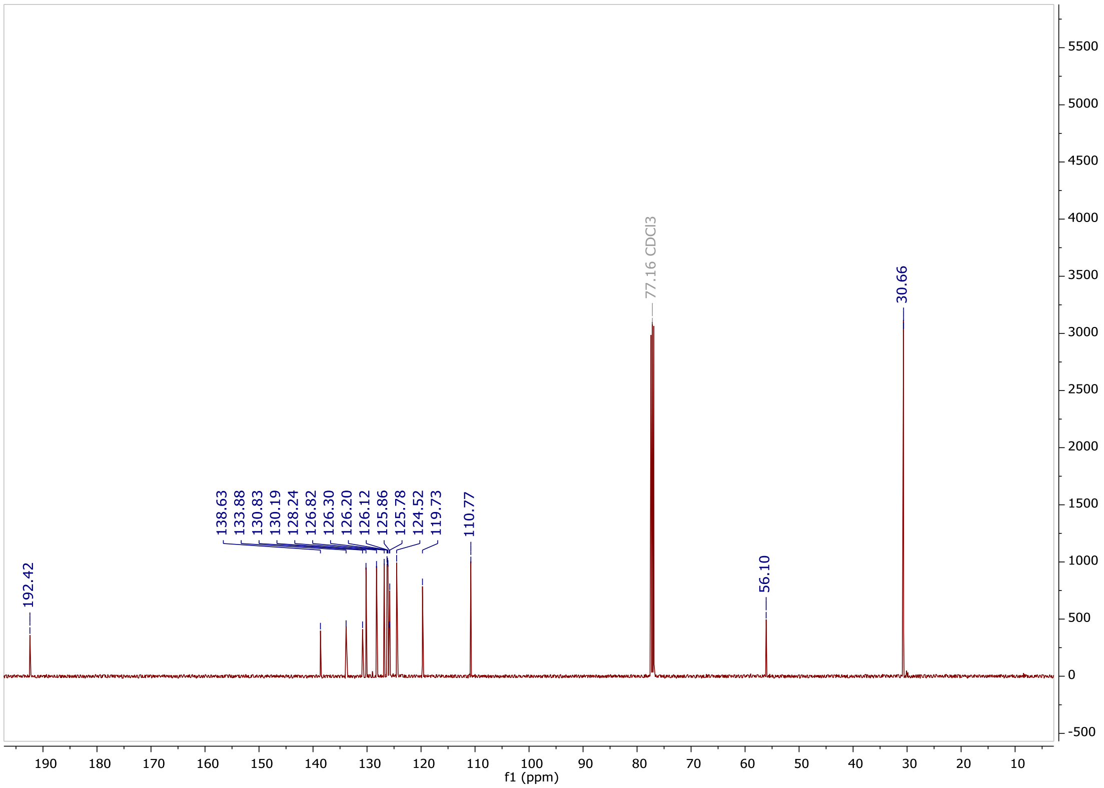

^{1}H and ^{13}C NMR data for 2g.

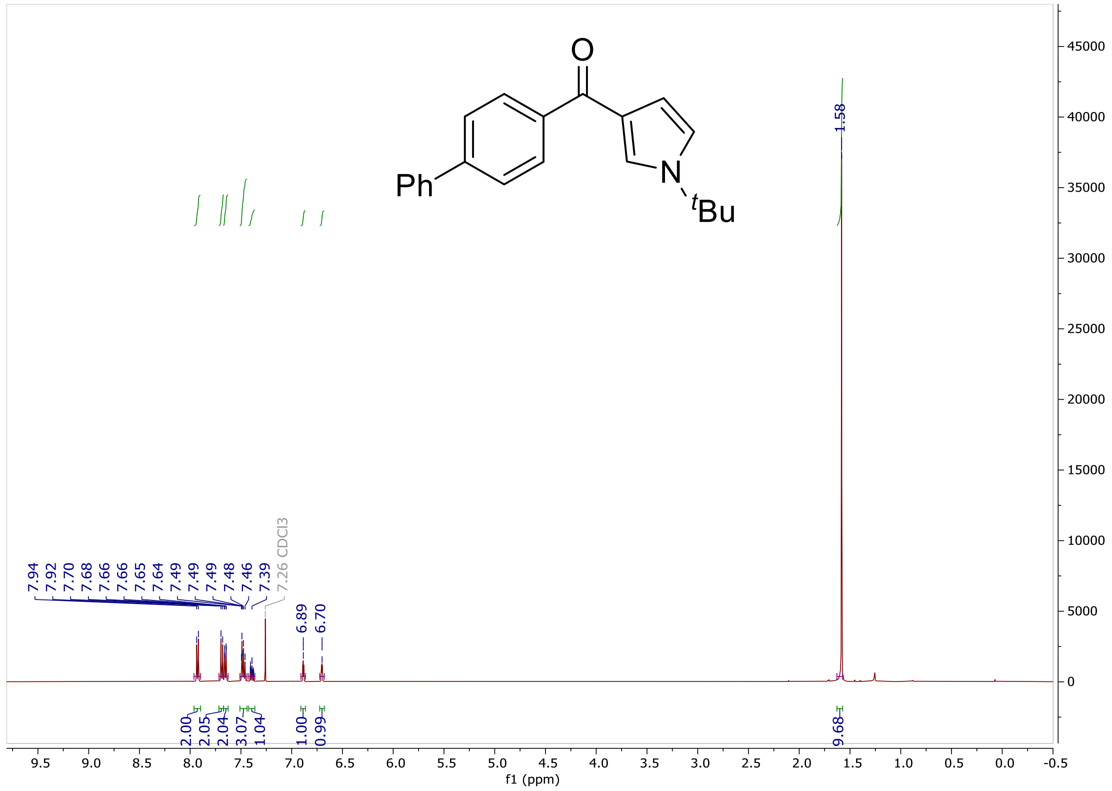

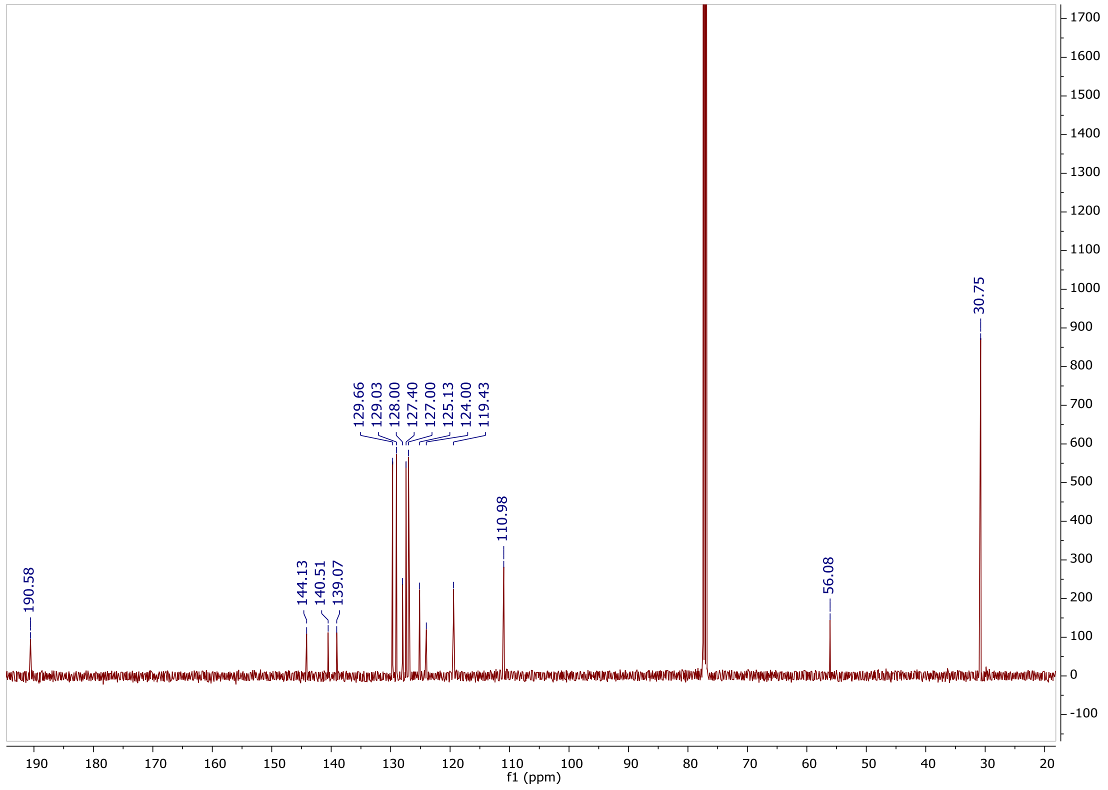

^{1}H and ^{13}C NMR data for 2h.

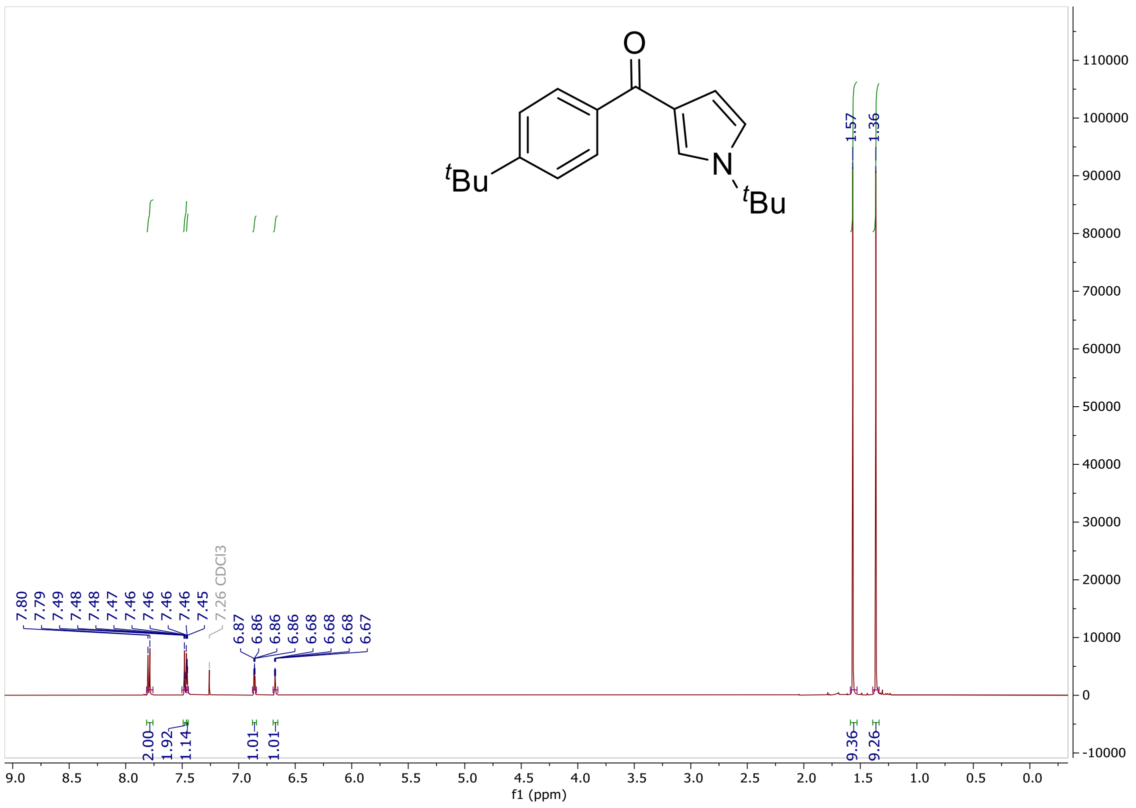

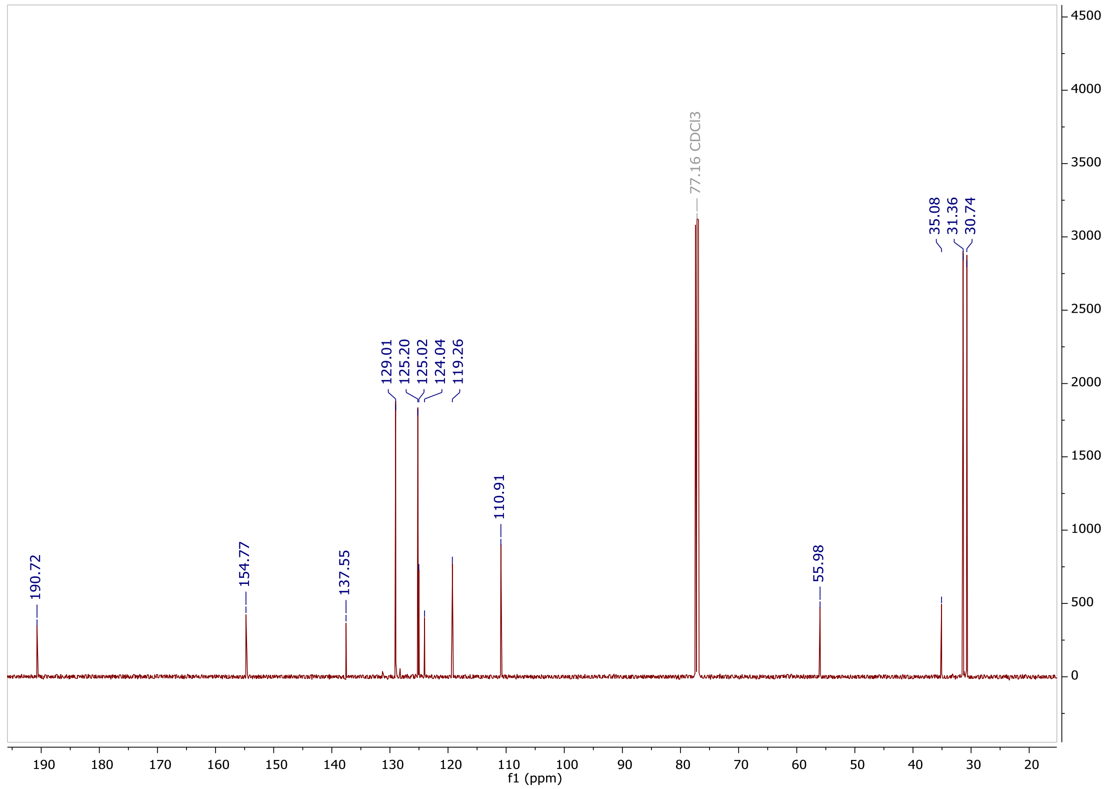

^{1}H and ^{13}C NMR data for 2i

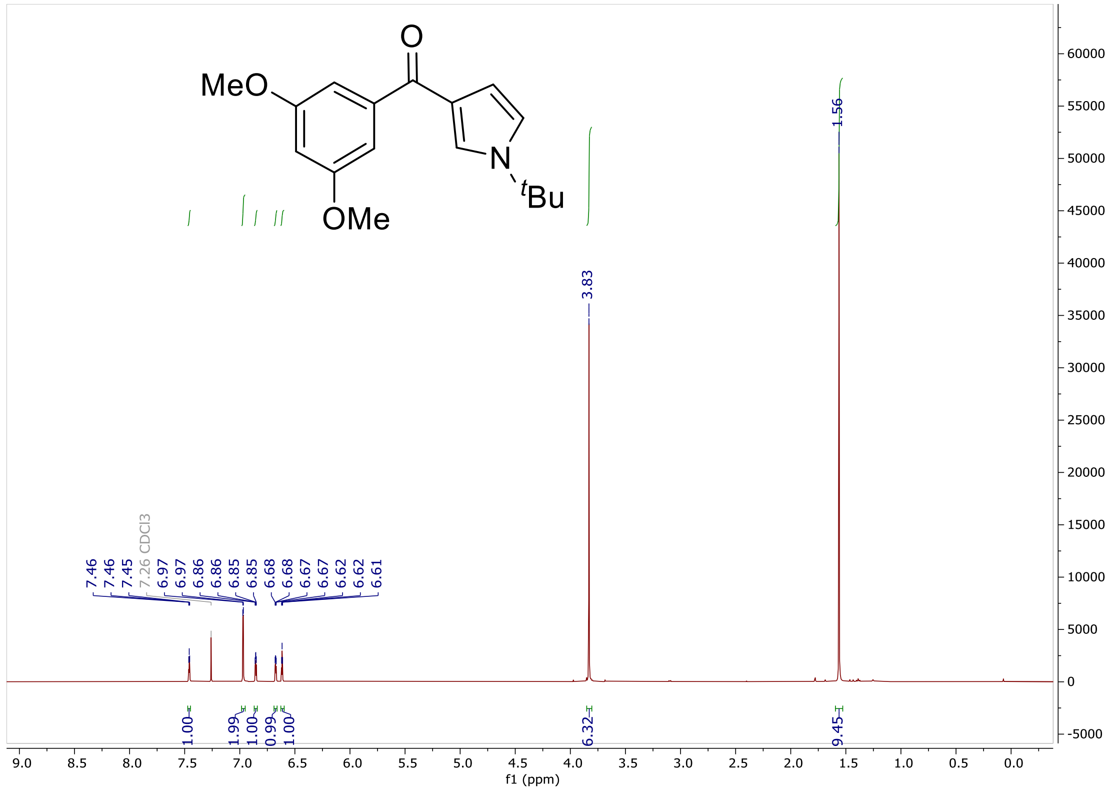

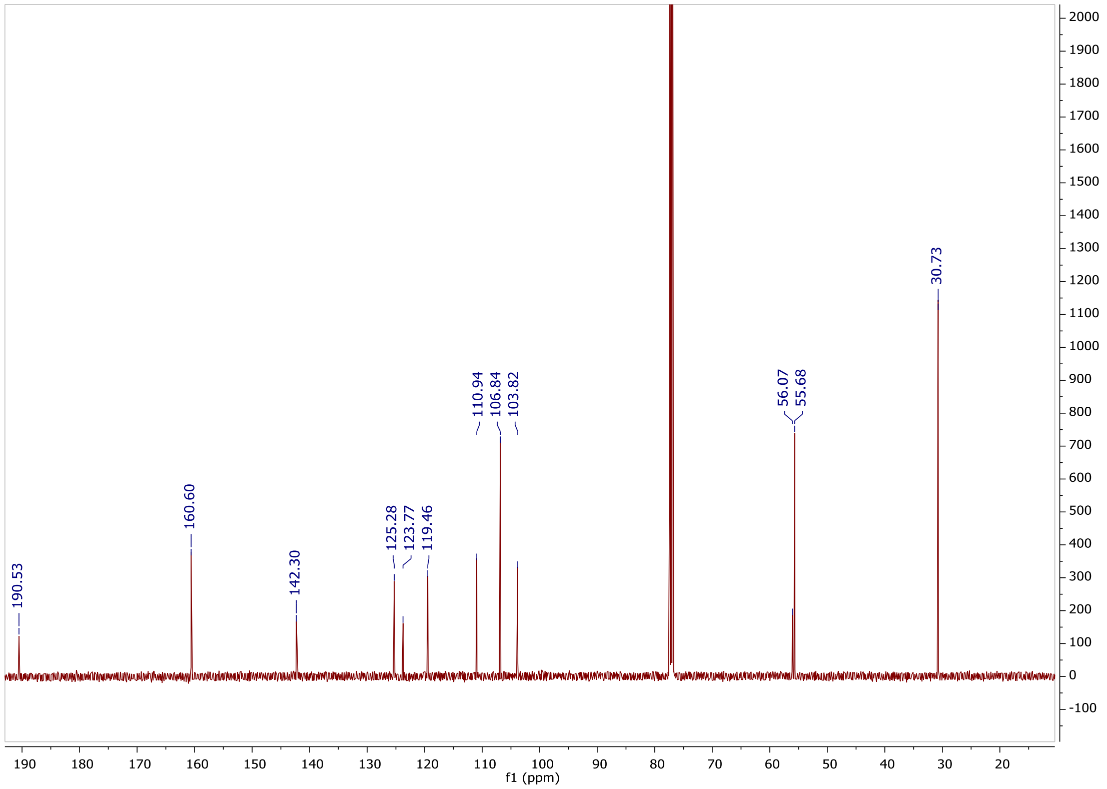

^{1}H and ^{13}C NMR data for 2j.

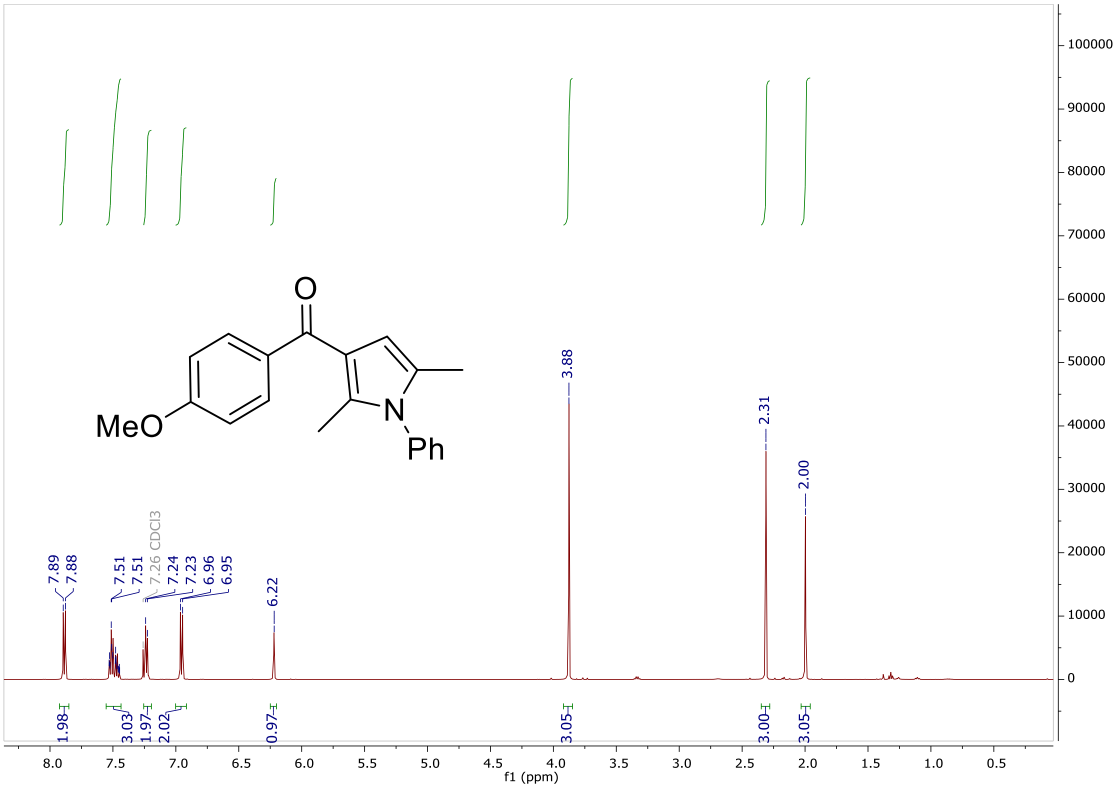

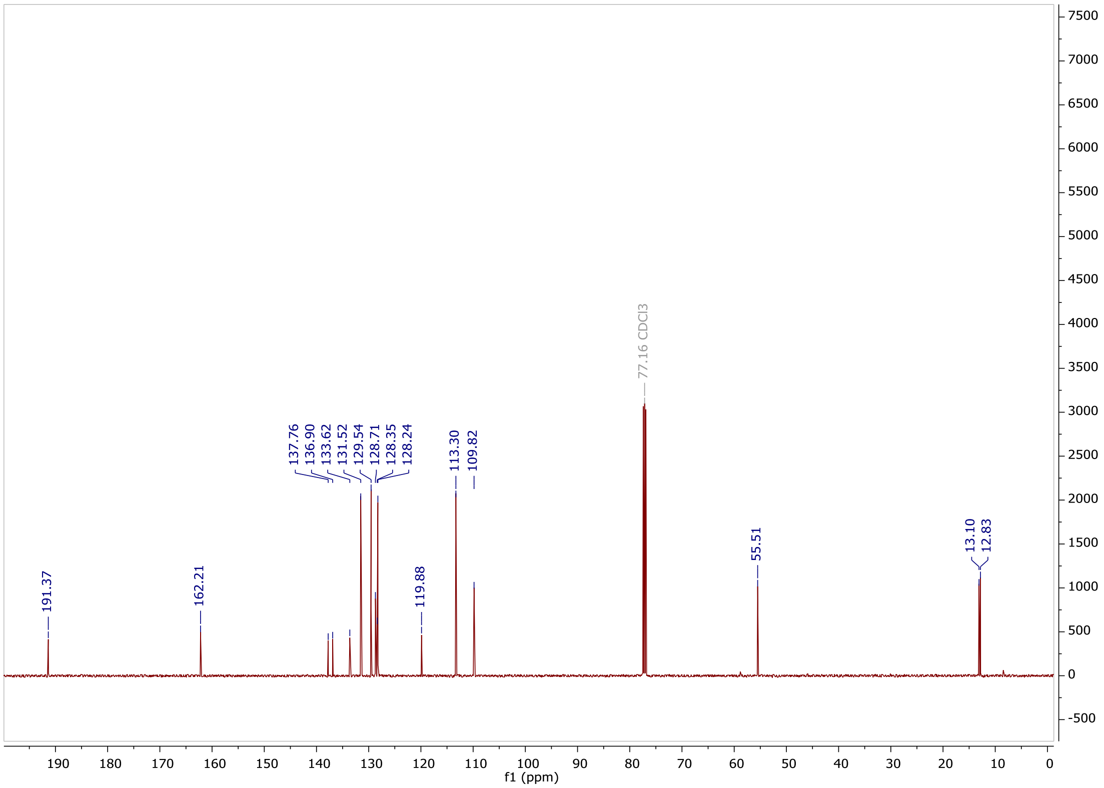

^{1}H and ^{13}C NMR data for 2k.

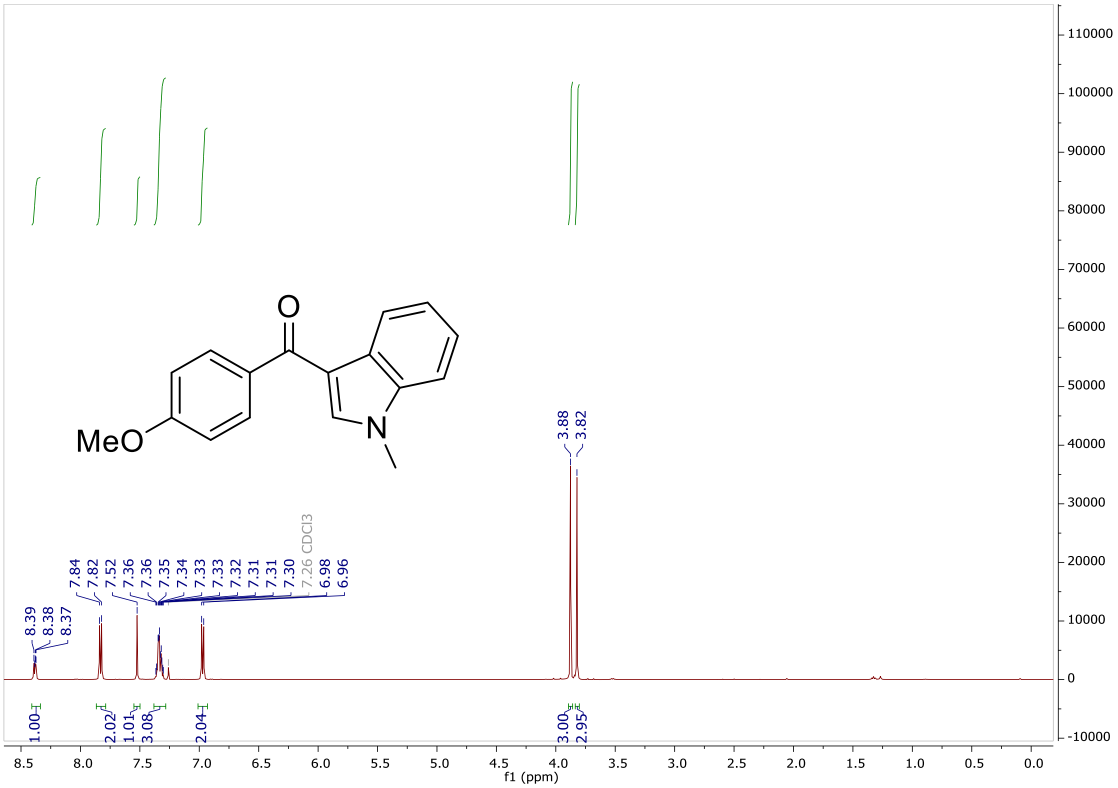

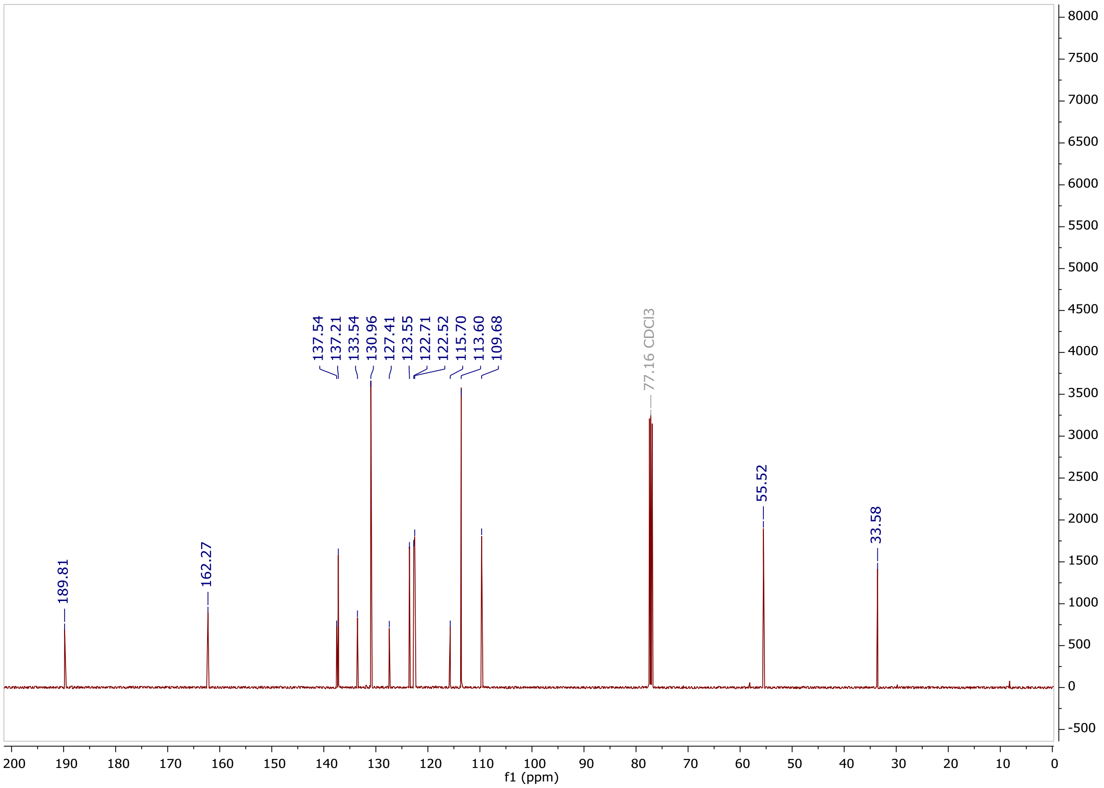

^{1}H and ^{13}C NMR data for 2l.

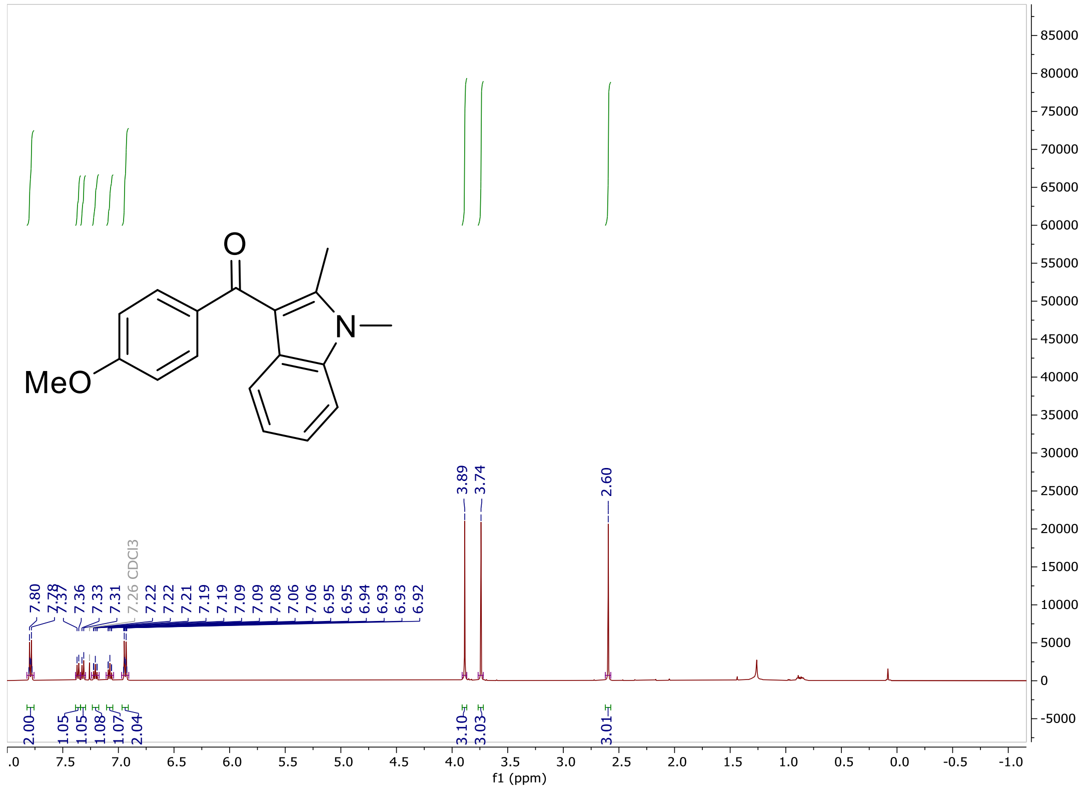

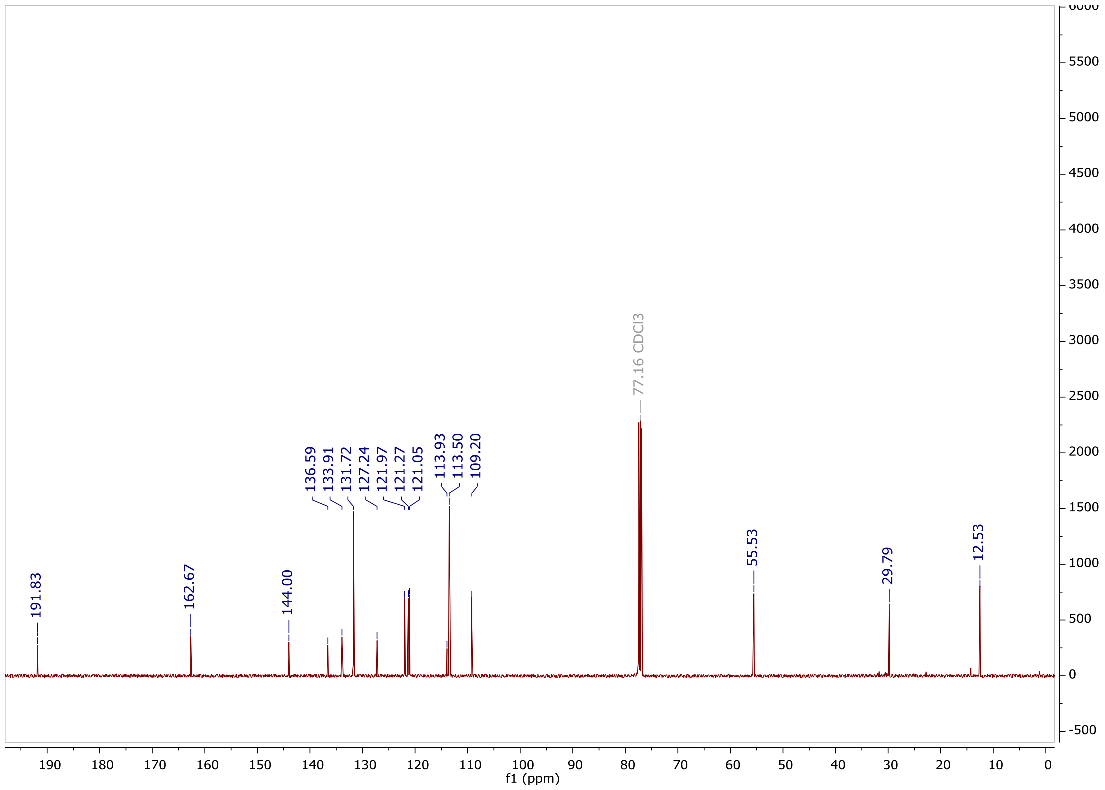

^{1}H and ^{13}C NMR data for 2m

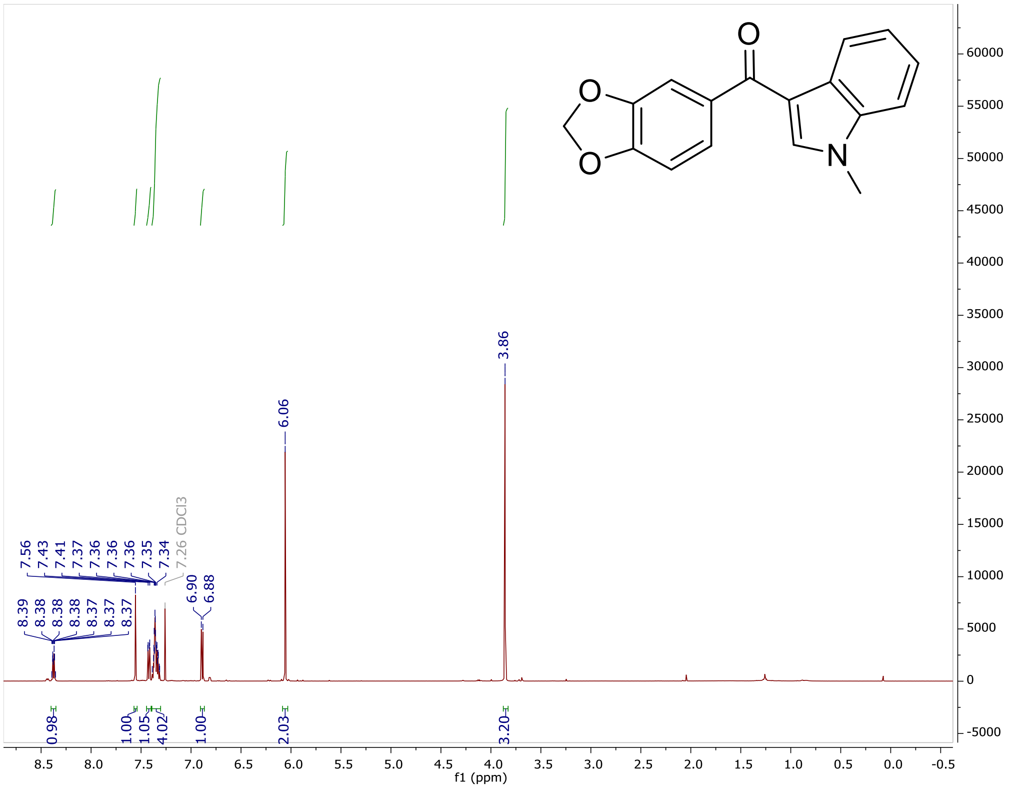

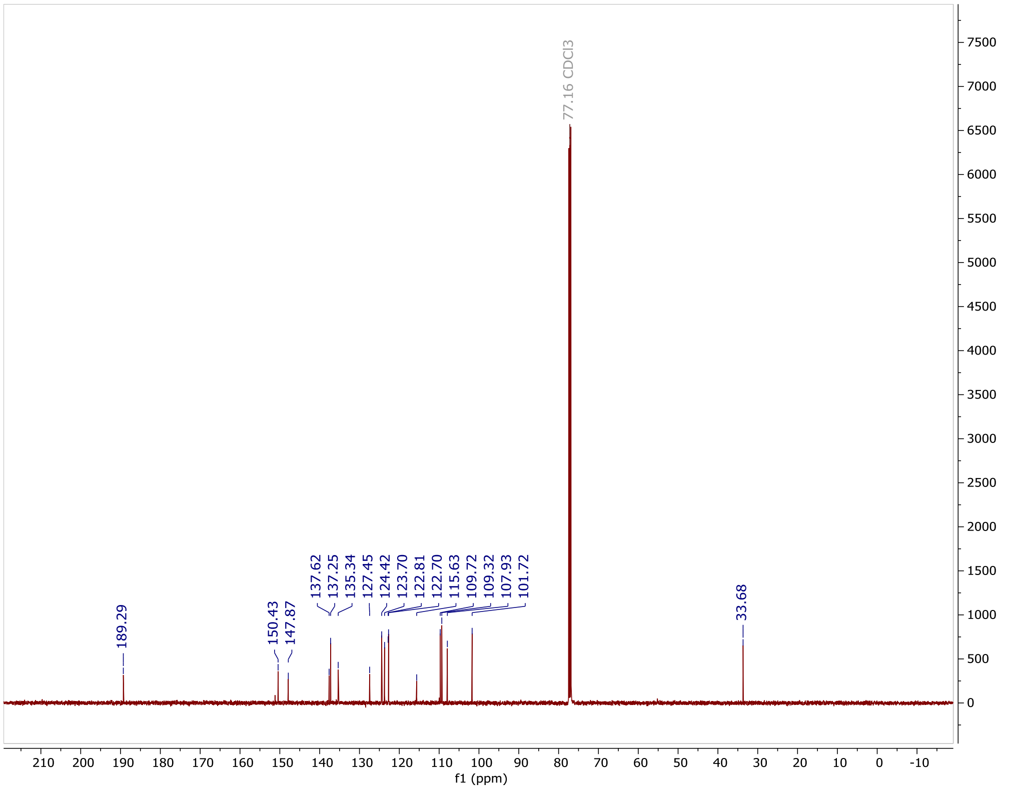

^{1}H and ^{13}C NMR data for 2n.

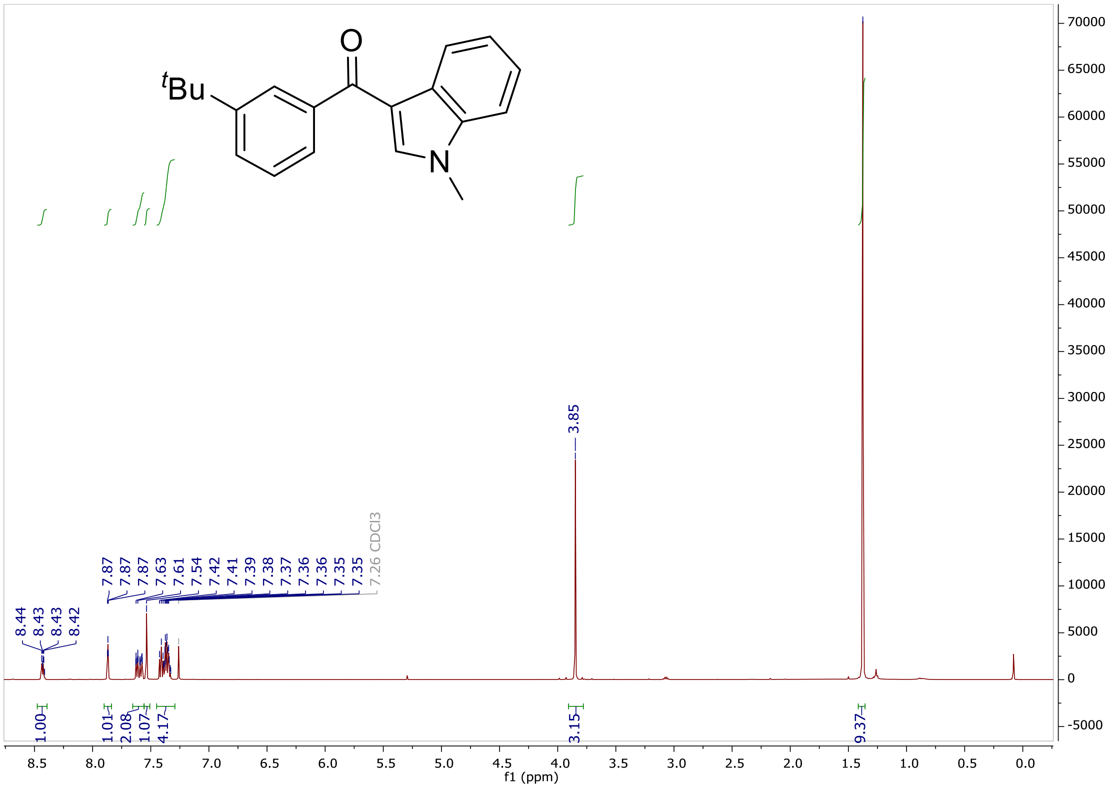

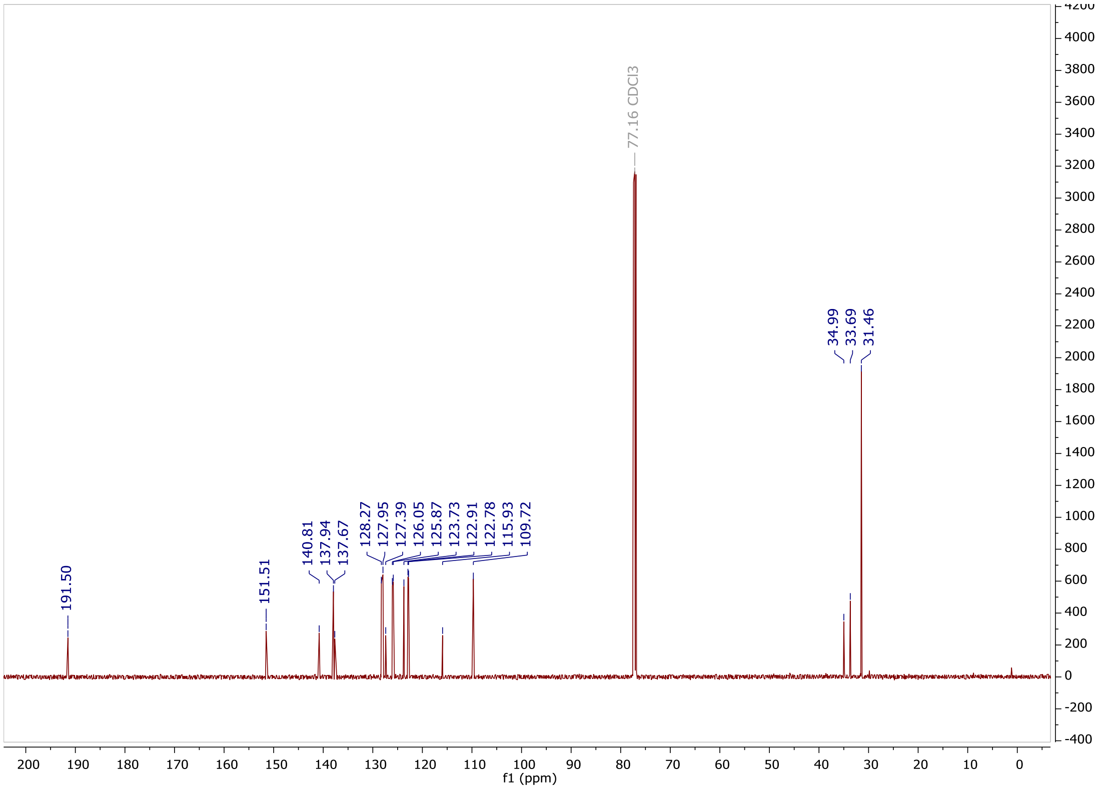

^{1}H and ^{13}C NMR data for 2o.

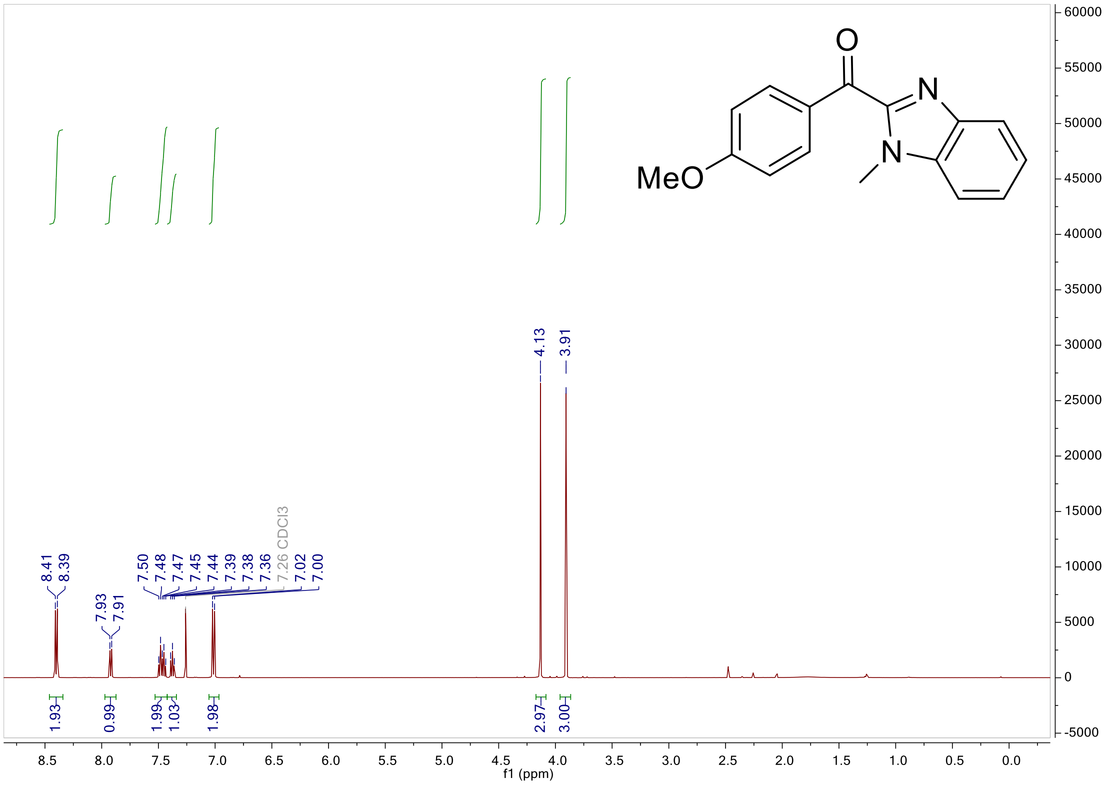

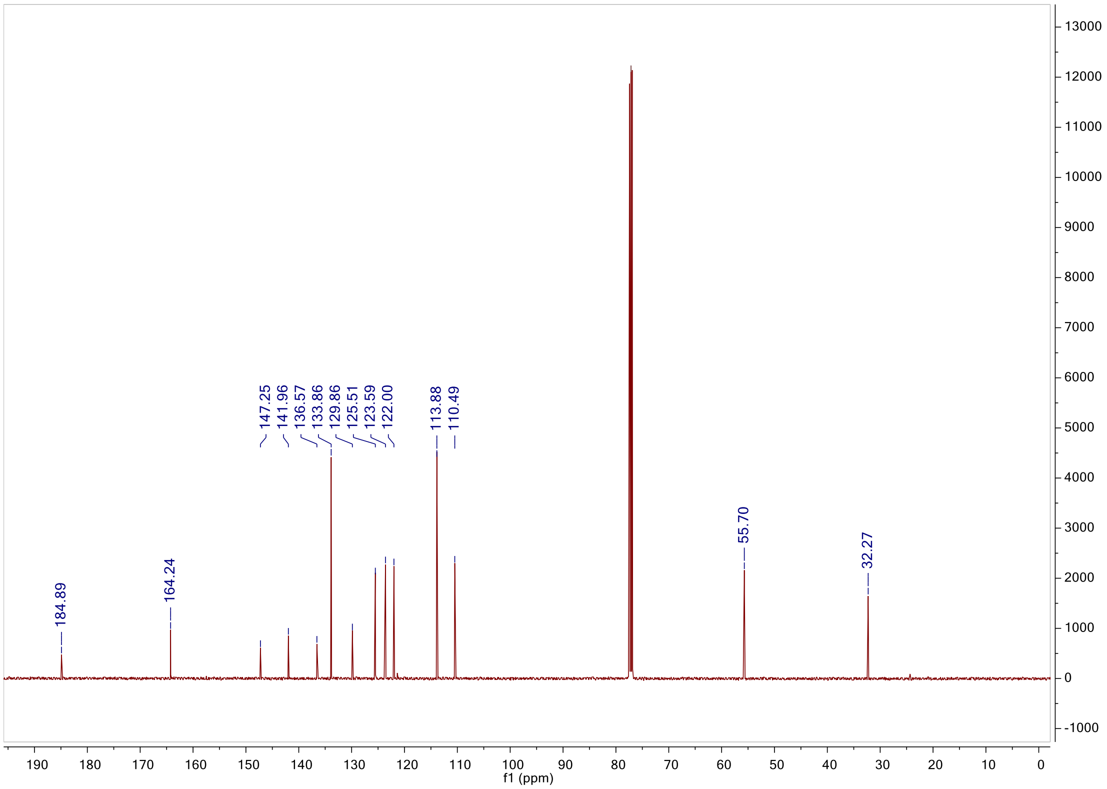

^{1}H and ^{13}C NMR data for 3a.

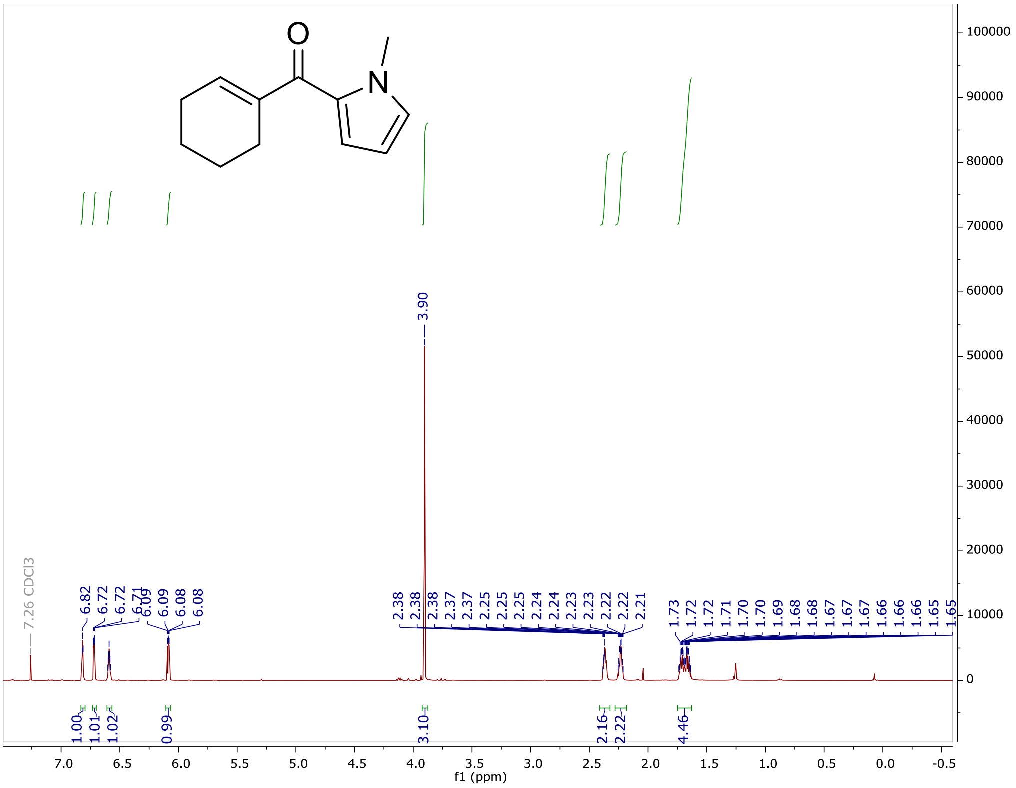

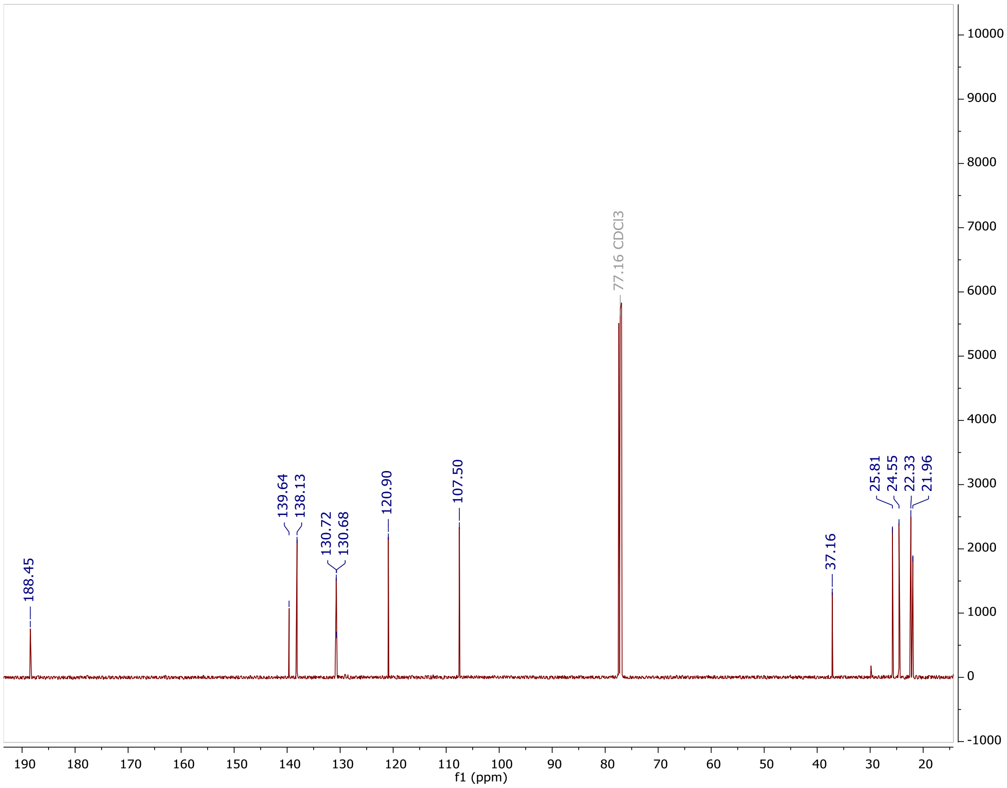

NOESY data of for 3a ($CDCl_3$):

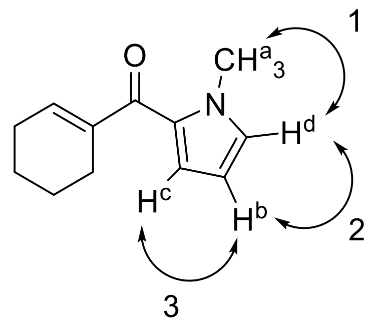

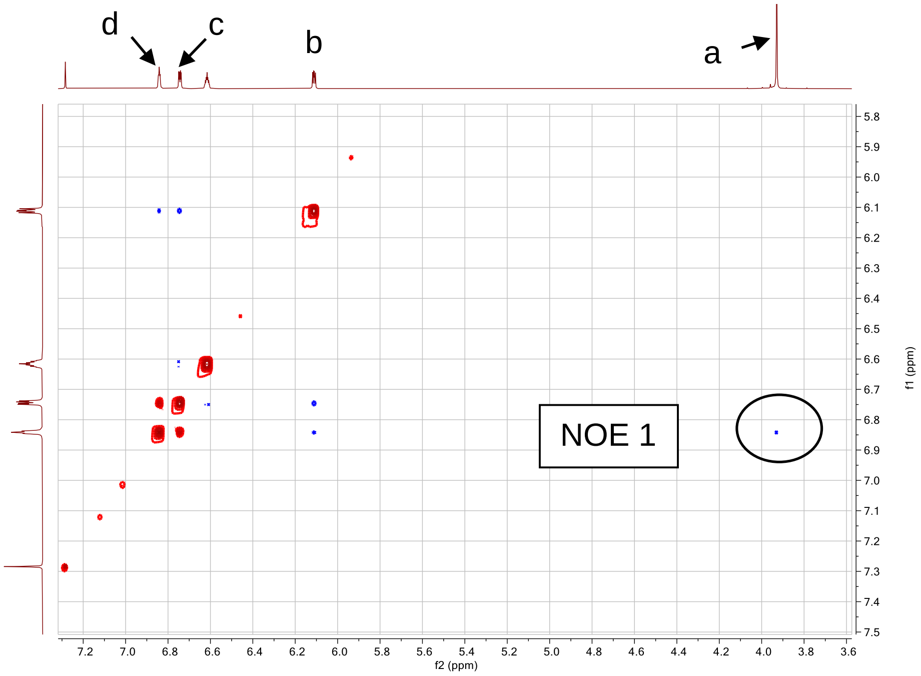

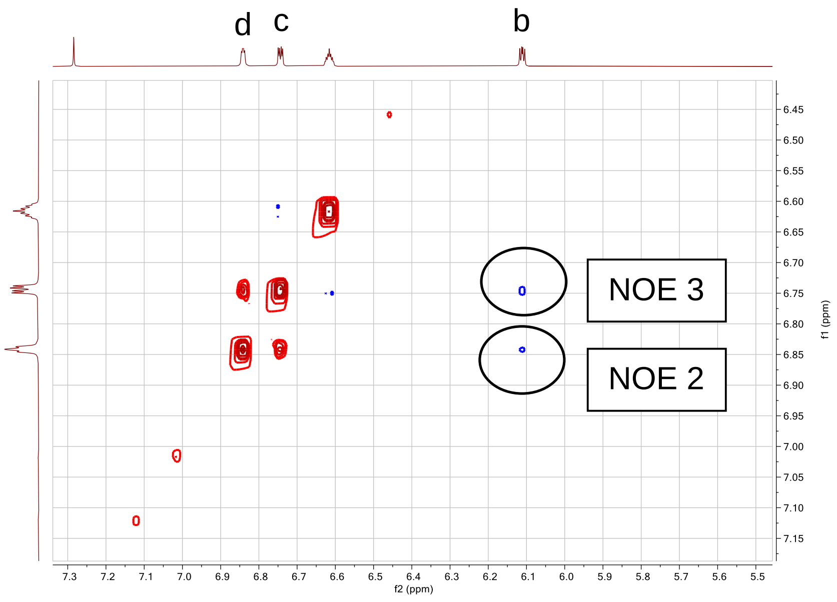

^{1}H and ^{13}C NMR data for 3b.

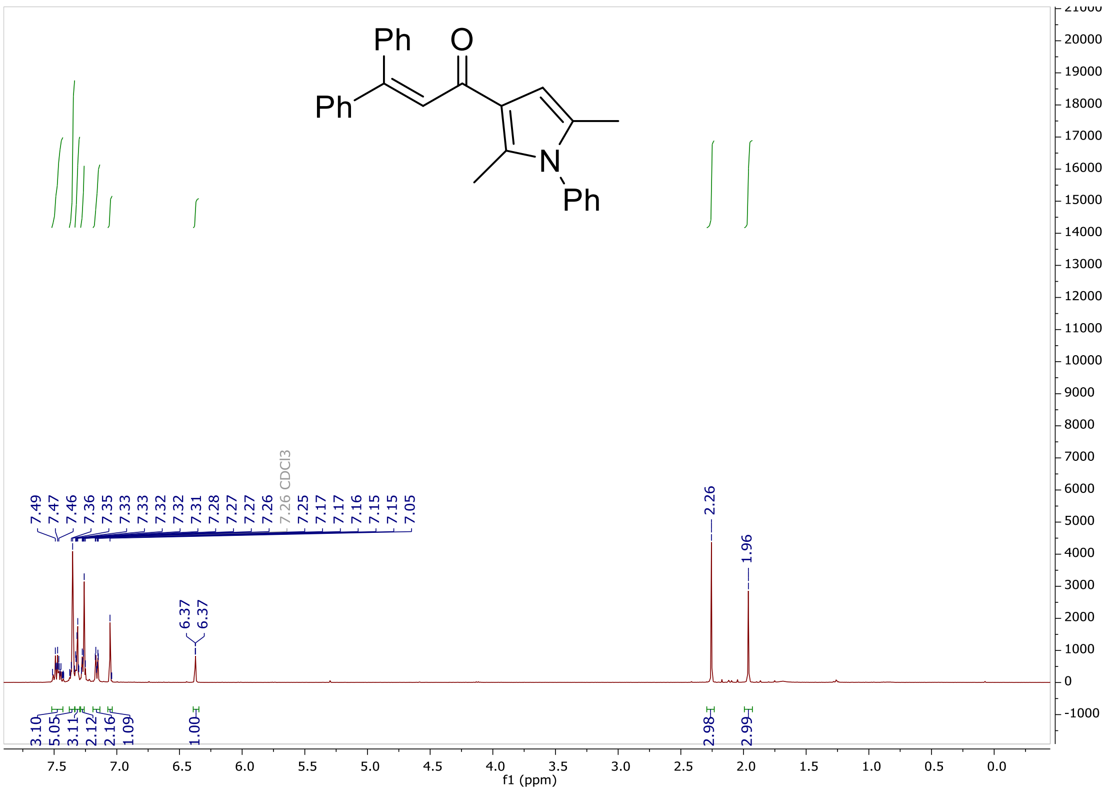

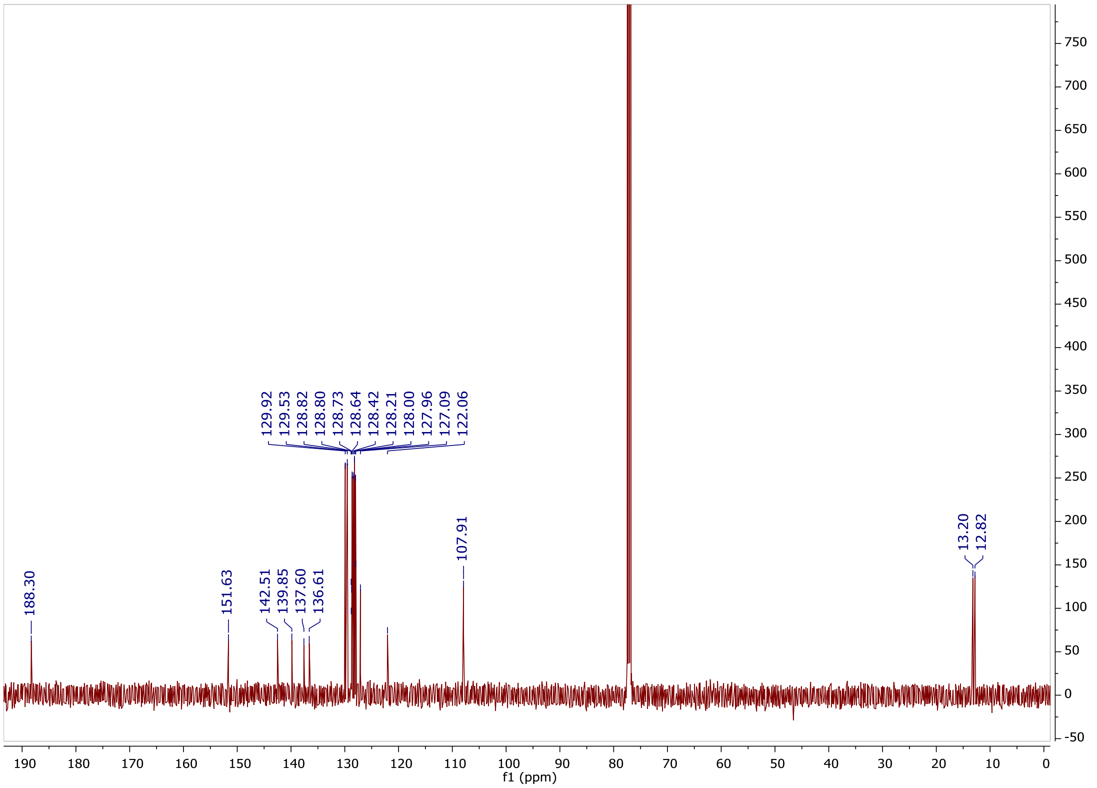

^{1}H and ^{13}C NMR data for 3c.

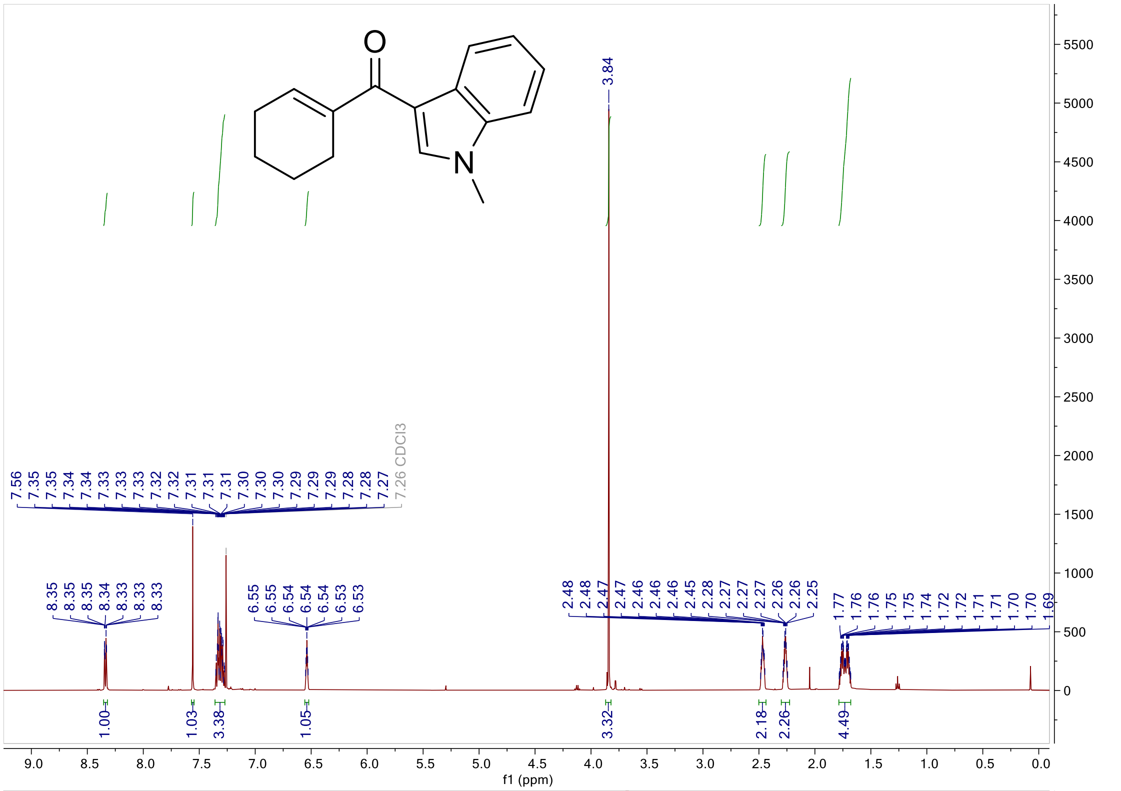

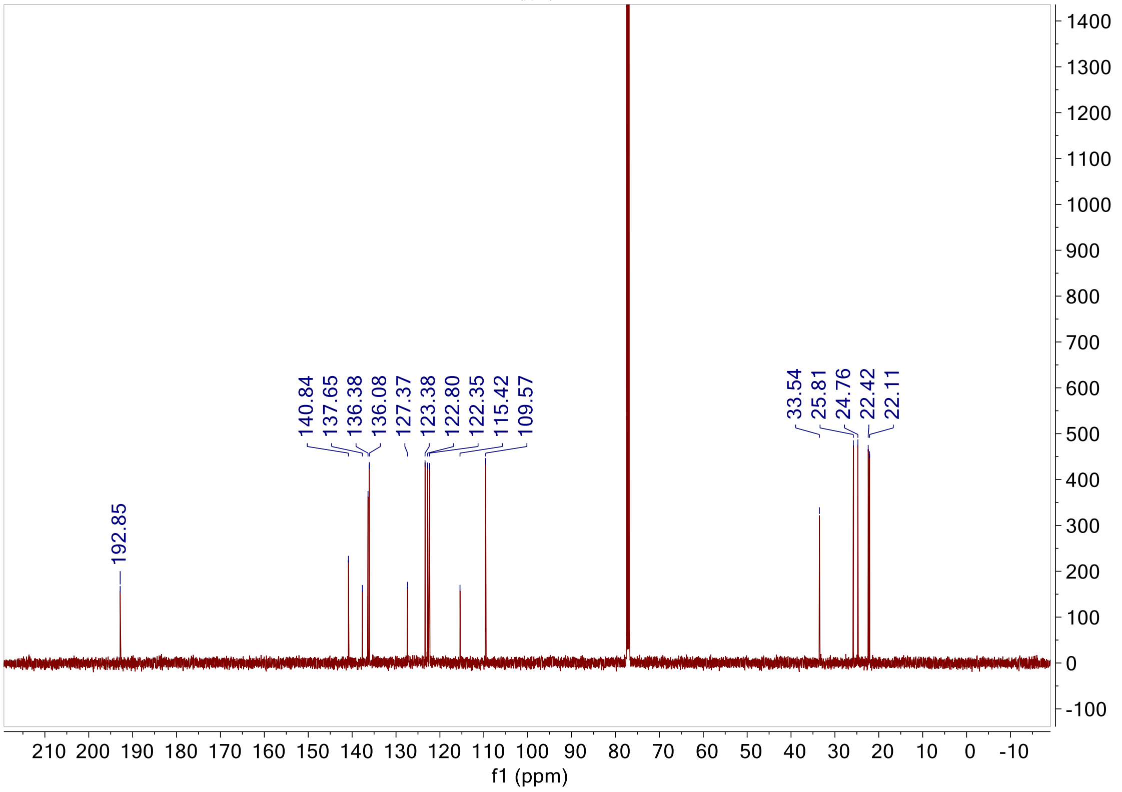

^{1}H and ^{13}C NMR data for 3d.

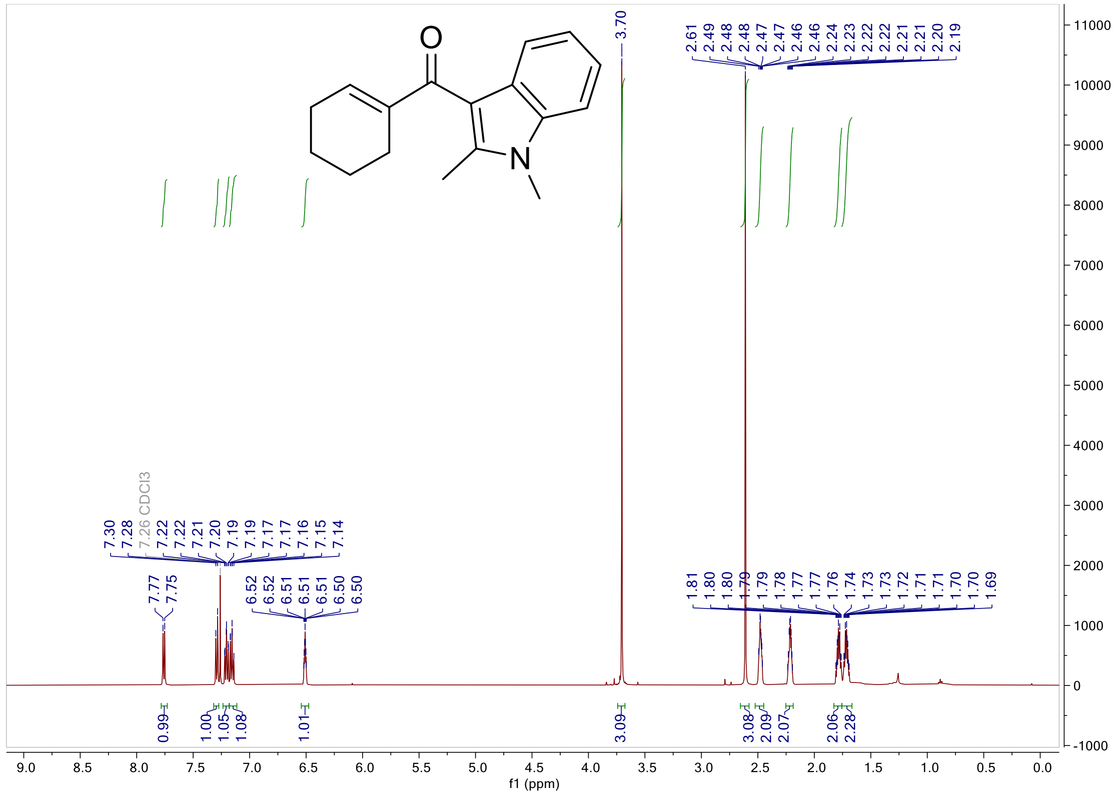

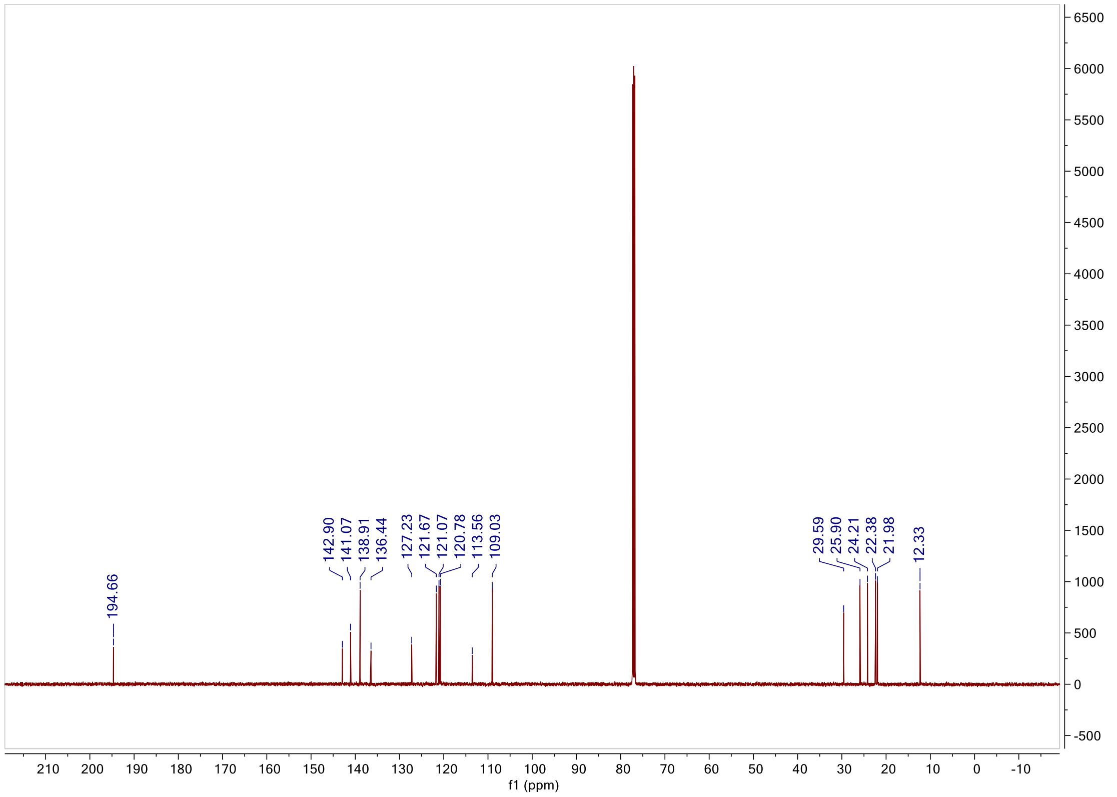

^{1}H and ^{13}C NMR data for 3e.

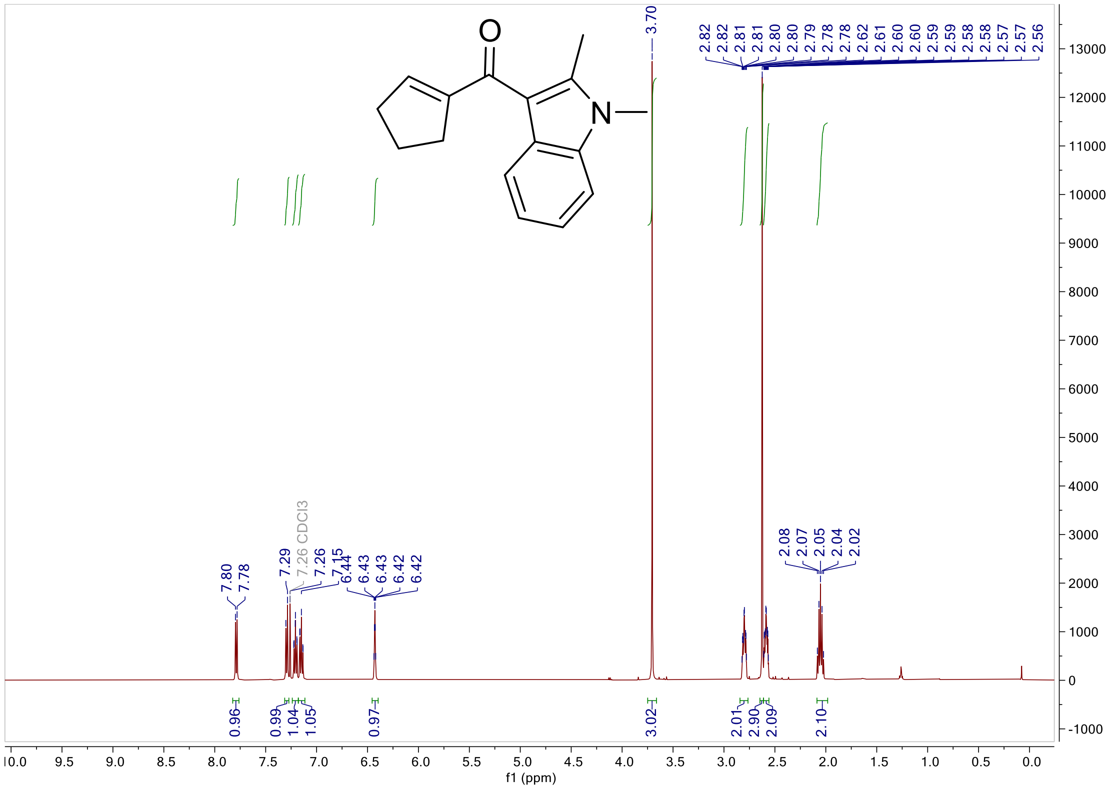

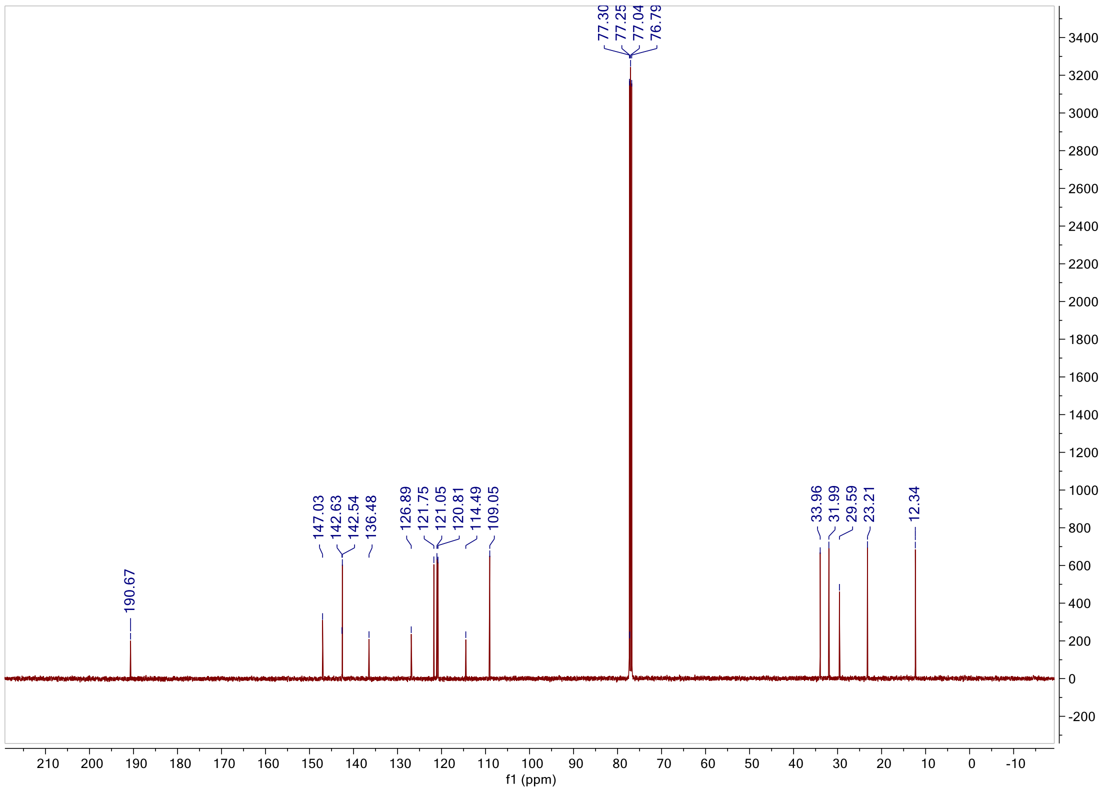

^{1}H and ^{13}C NMR data for 3f.

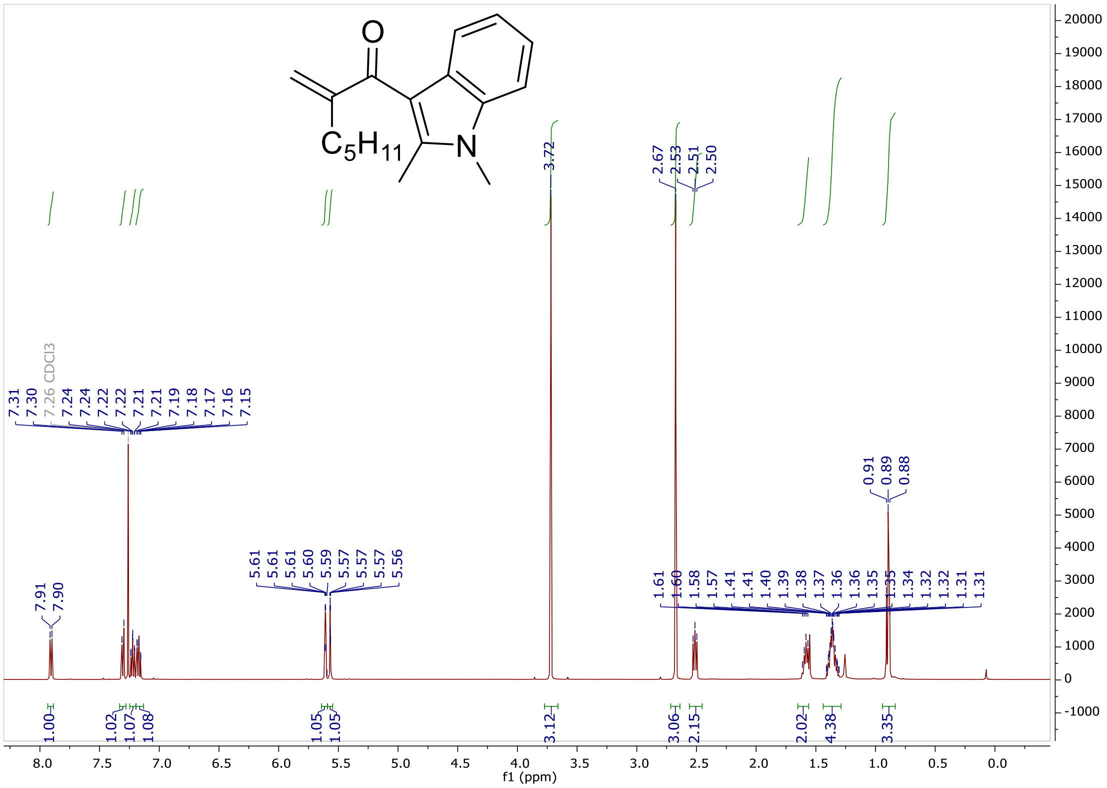

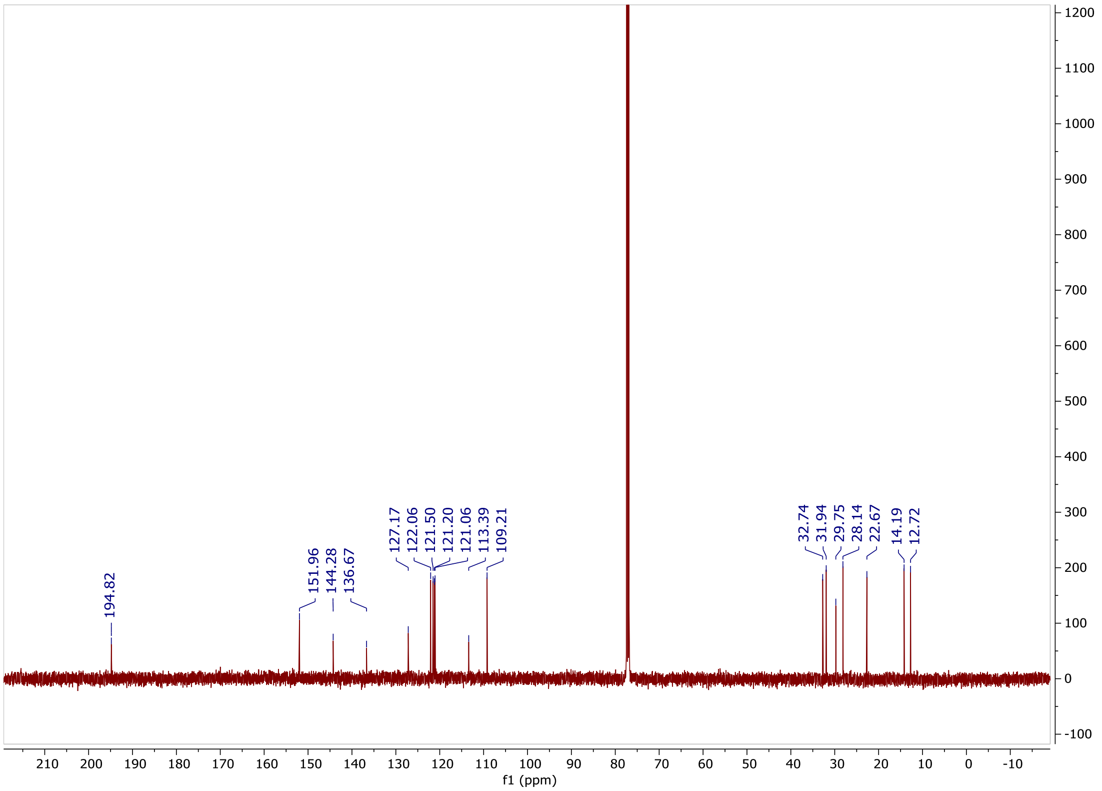

^{1}H and ^{13}C NMR data for 3g.

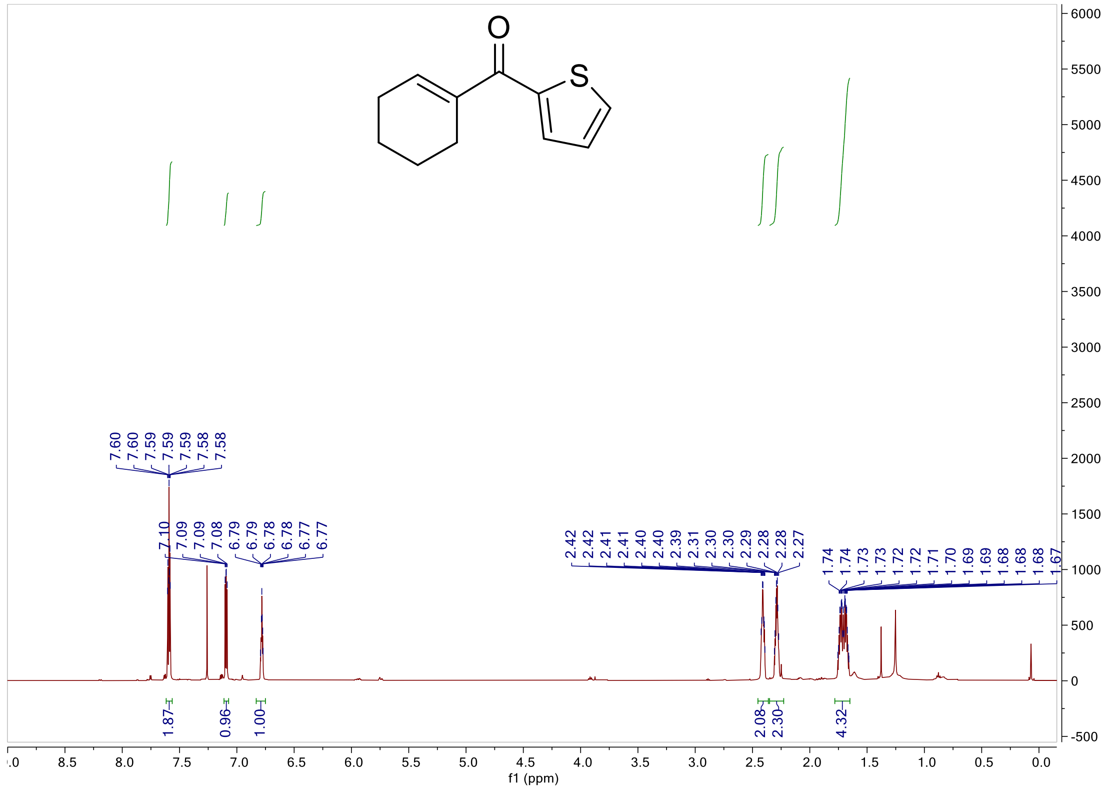

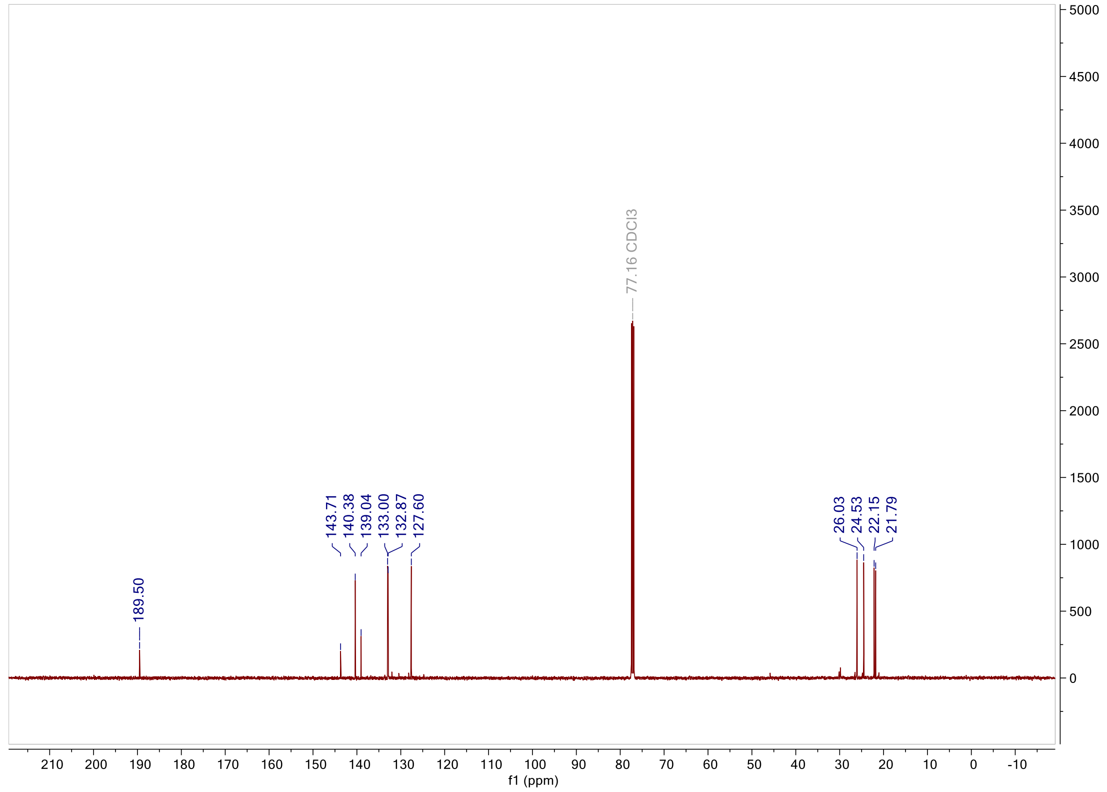

NOESY data for 3g ($CDCl_3$):

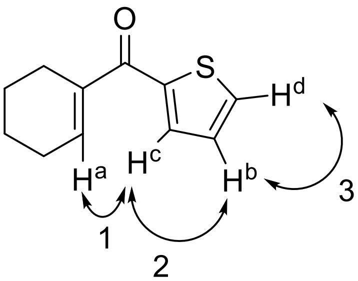

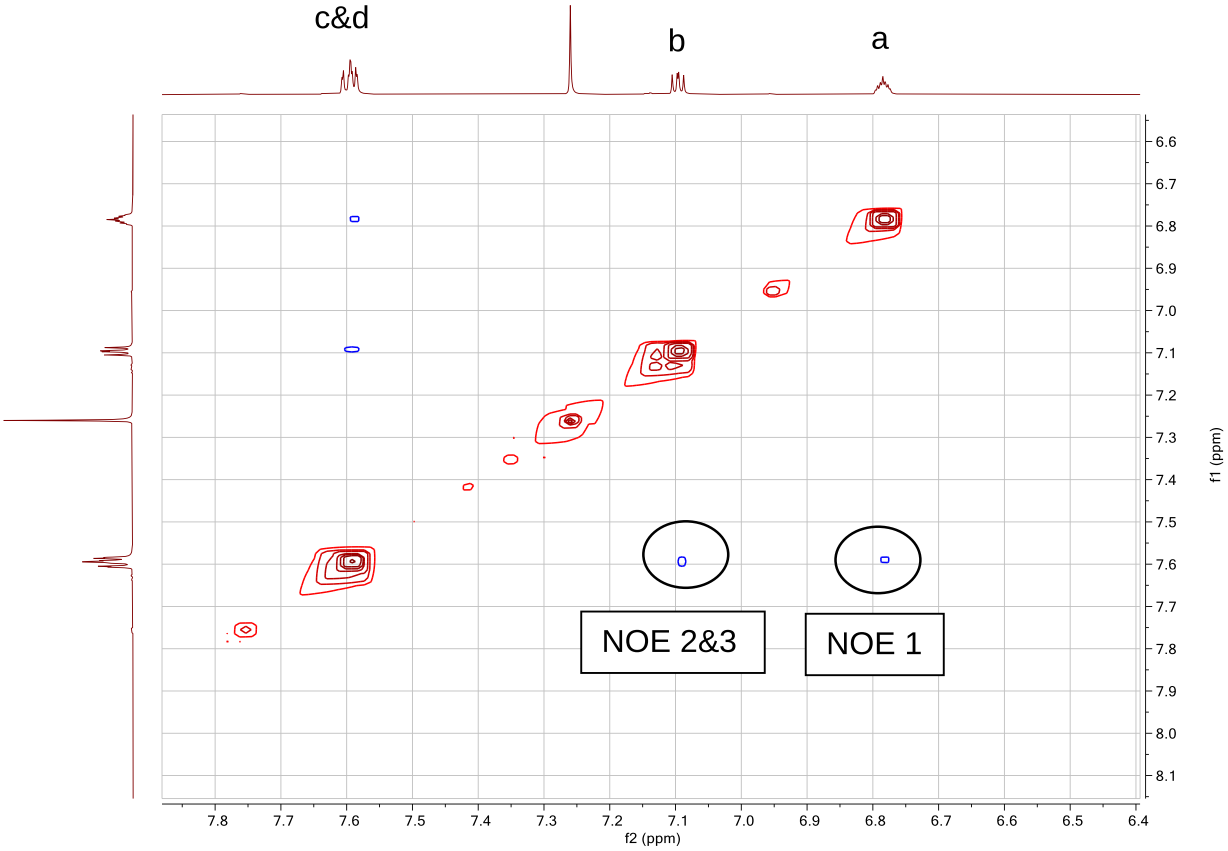

^{1}H and ^{13}C NMR data for 3h.

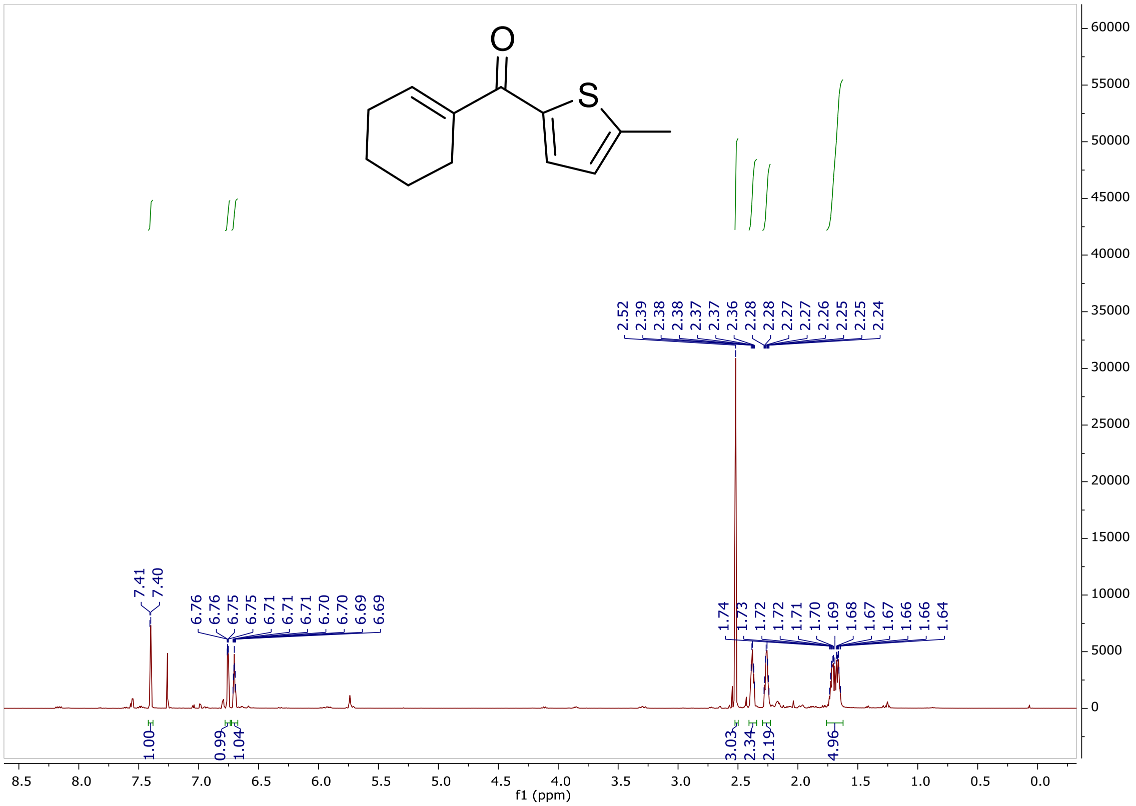

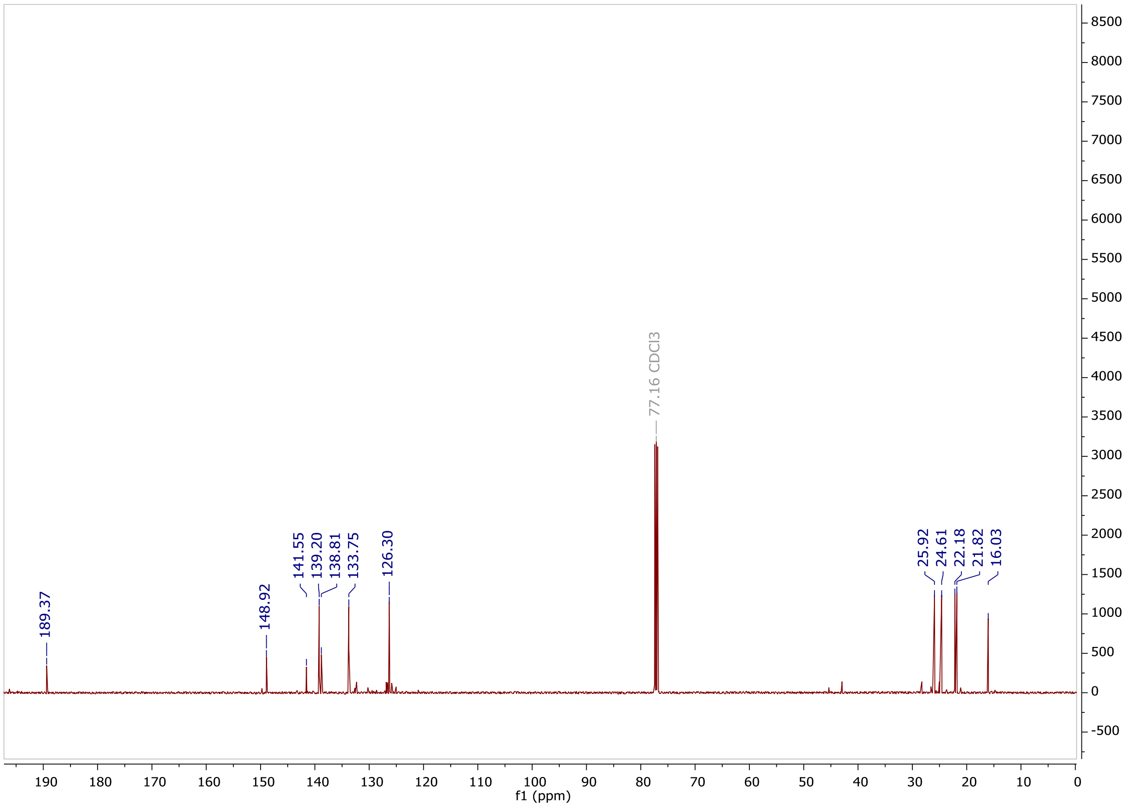

NOESY data for 3h ($CDCl_3$)

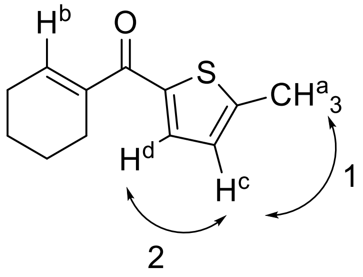

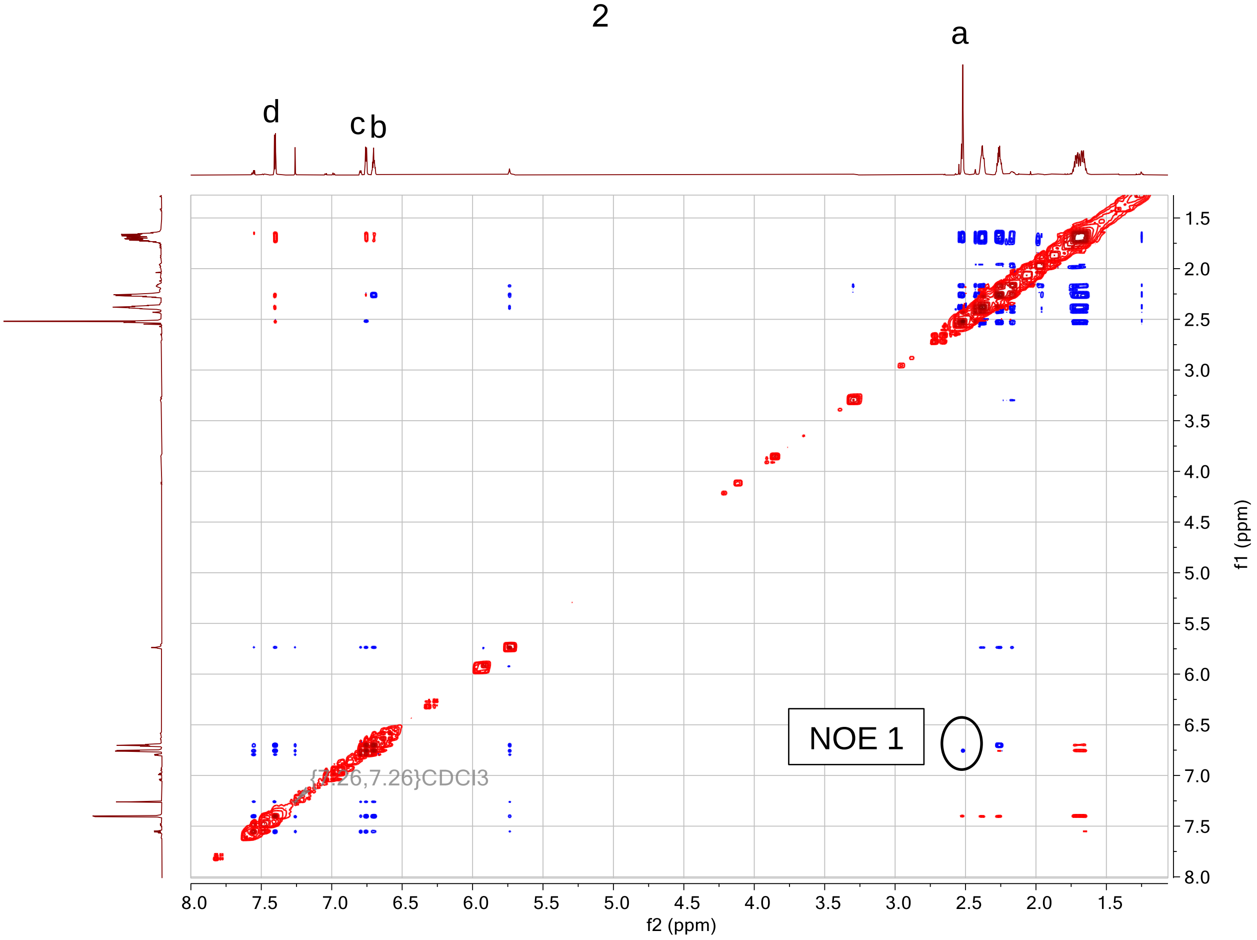

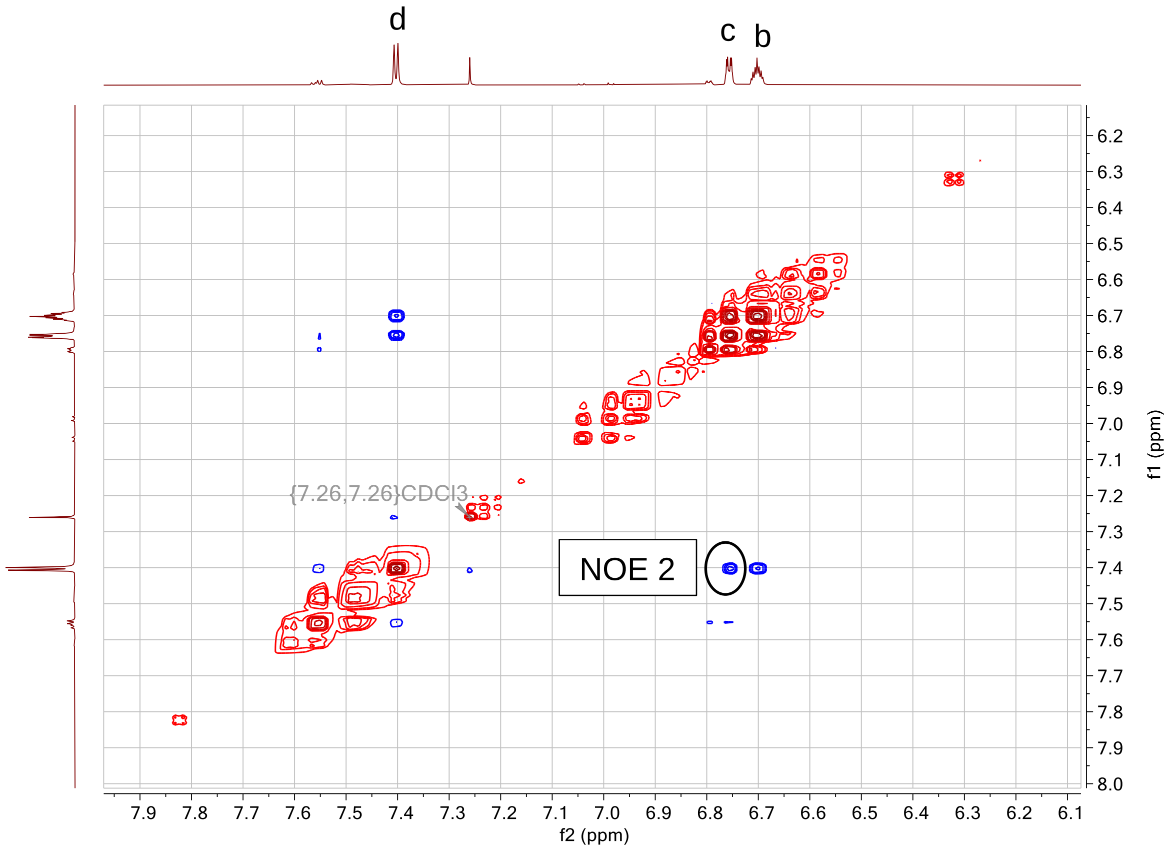
d
c
b
{7.26,7.26}CDCl3
NOE 2
f2 (ppm)
f1 (ppm)

^{1}H and ^{13}C NMR data for 3i.

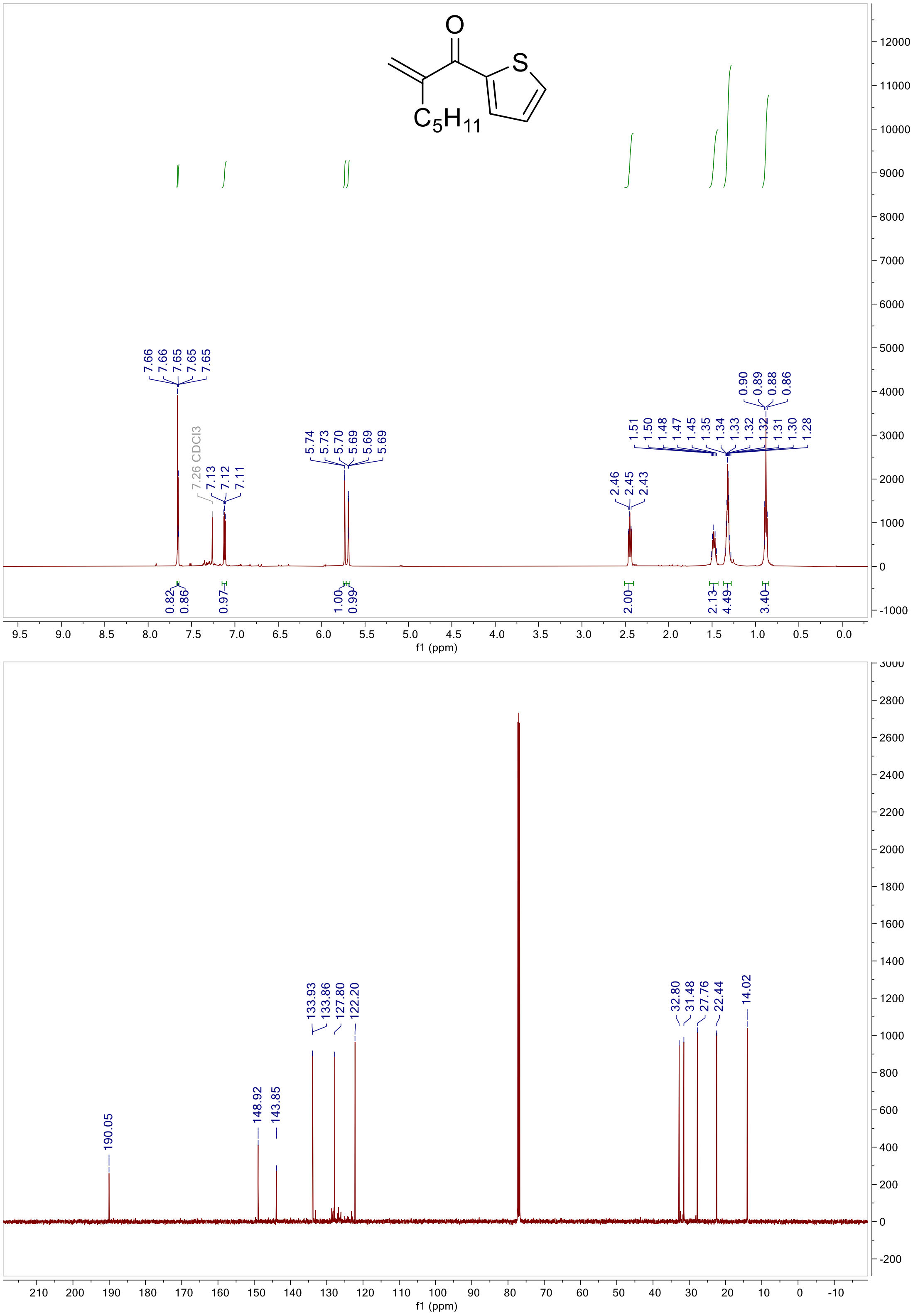

^{1}H and ^{13}C NMR data for 3j.

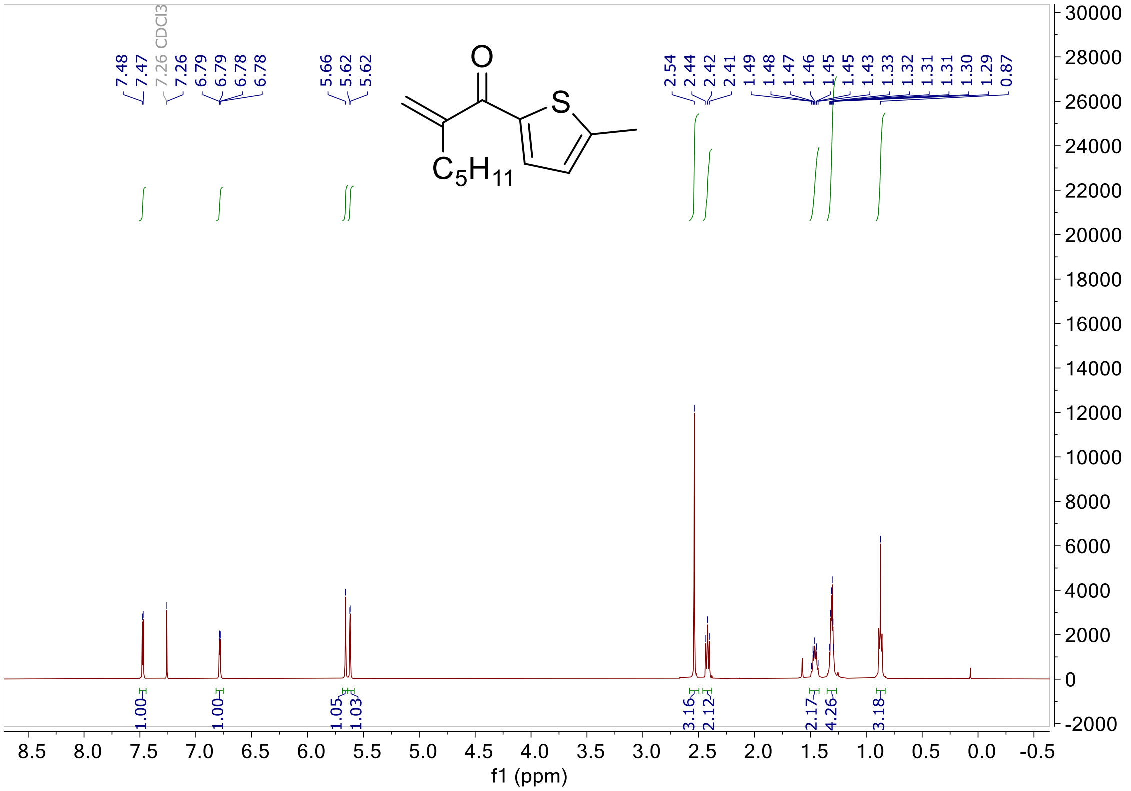

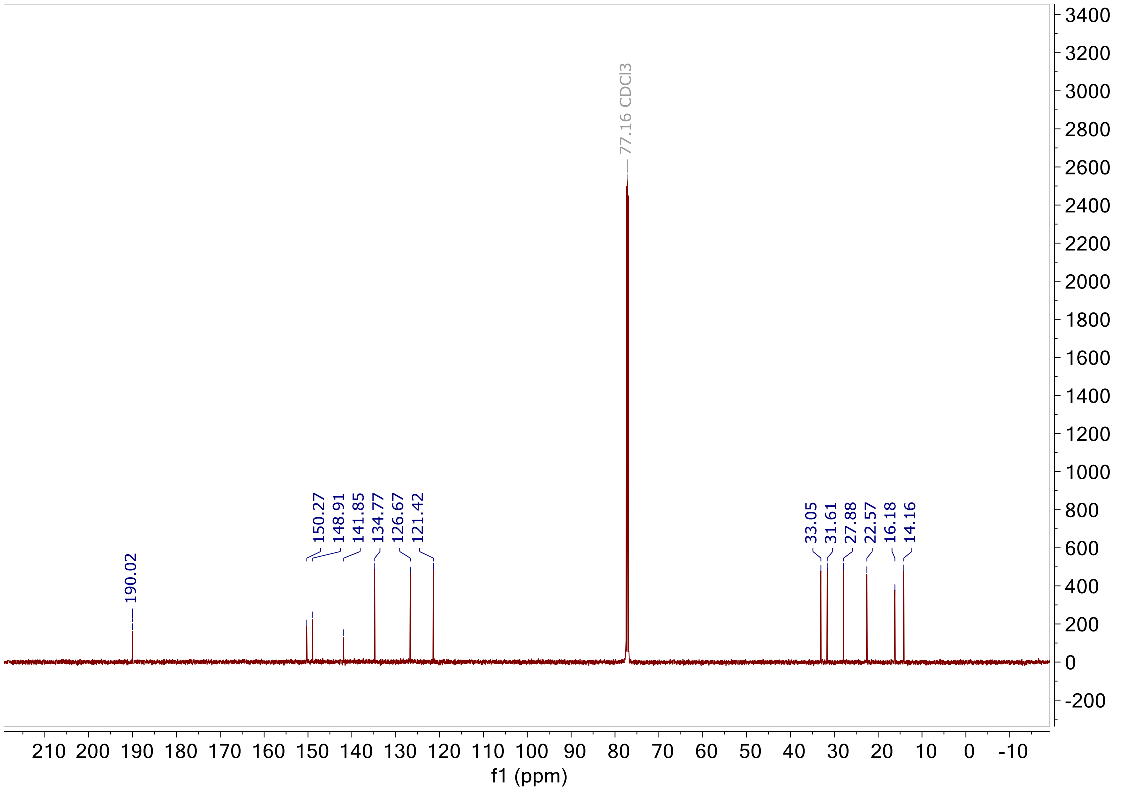

NOESY data for 3j ($CDCl_3$).

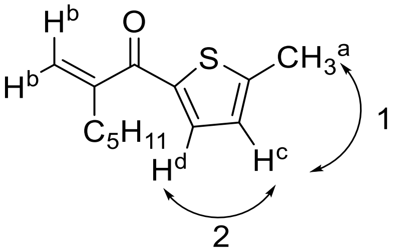

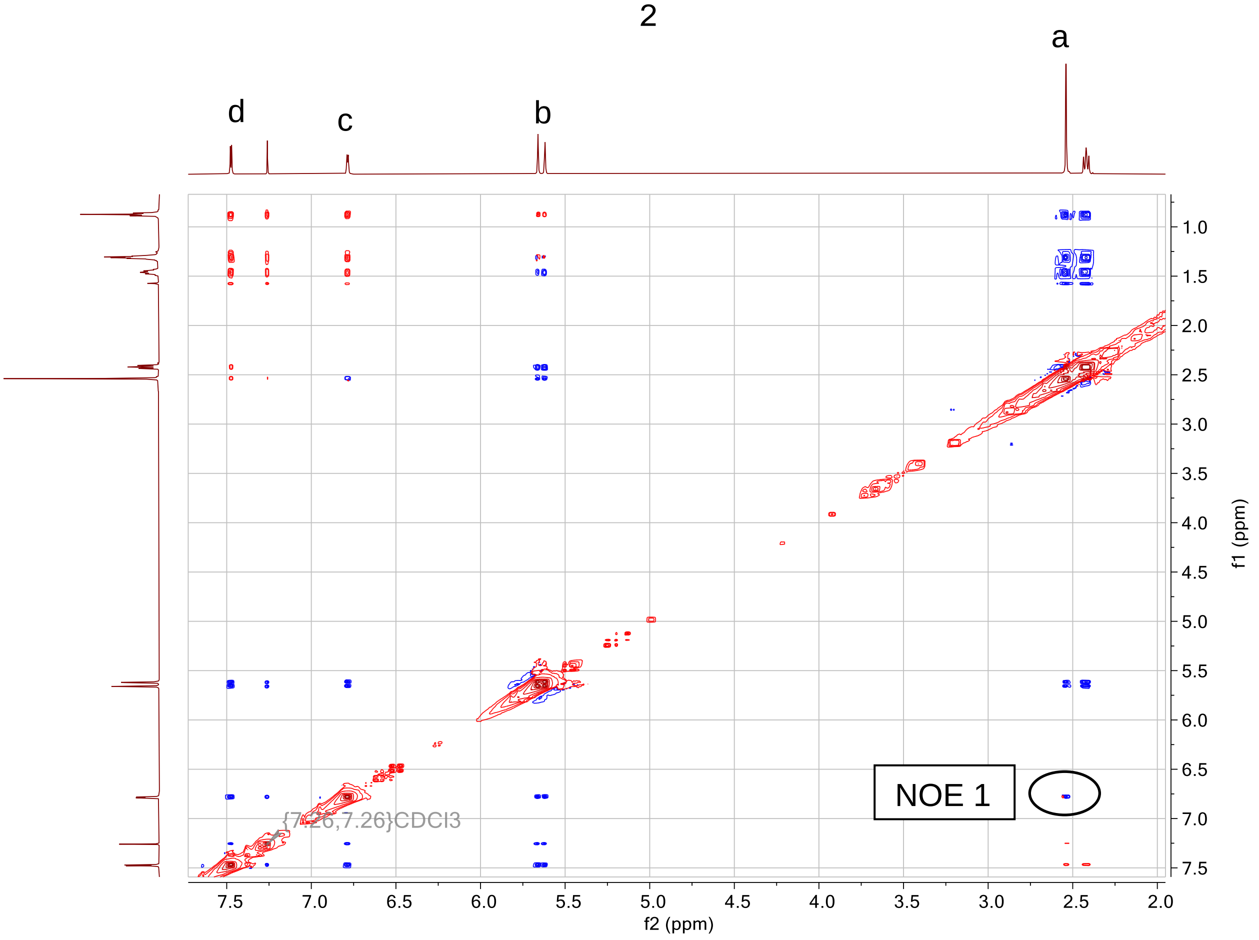

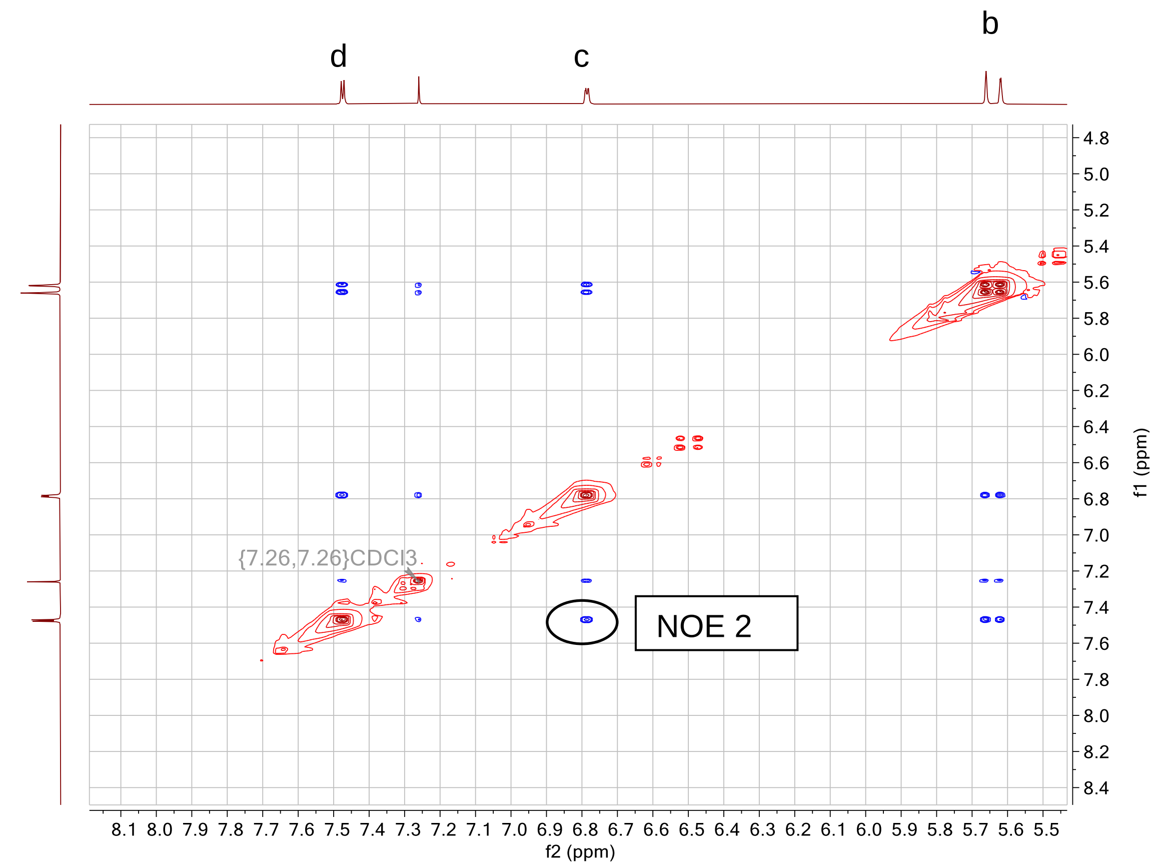
d
c
b
{7.26,7.26}CDCl3
NOE 2
8.1 8.0 7.9 7.8 7.7 7.6 7.5 7.4 7.3 7.2 7.1 7.0 6.9 6.8 6.7 6.6 6.5 6.4 6.3 6.2 6.1 6.0 5.9 5.8 5.7 5.6 5.5
f2 (ppm)
4.8 5.0 5.2 5.4 5.6 5.8 6.0 6.2 6.4 6.6 6.8 7.0 7.2 7.4 7.6 7.8 8.0 8.2 8.4
f1 (ppm)

^{1}H and ^{13}C NMR data for 3k.

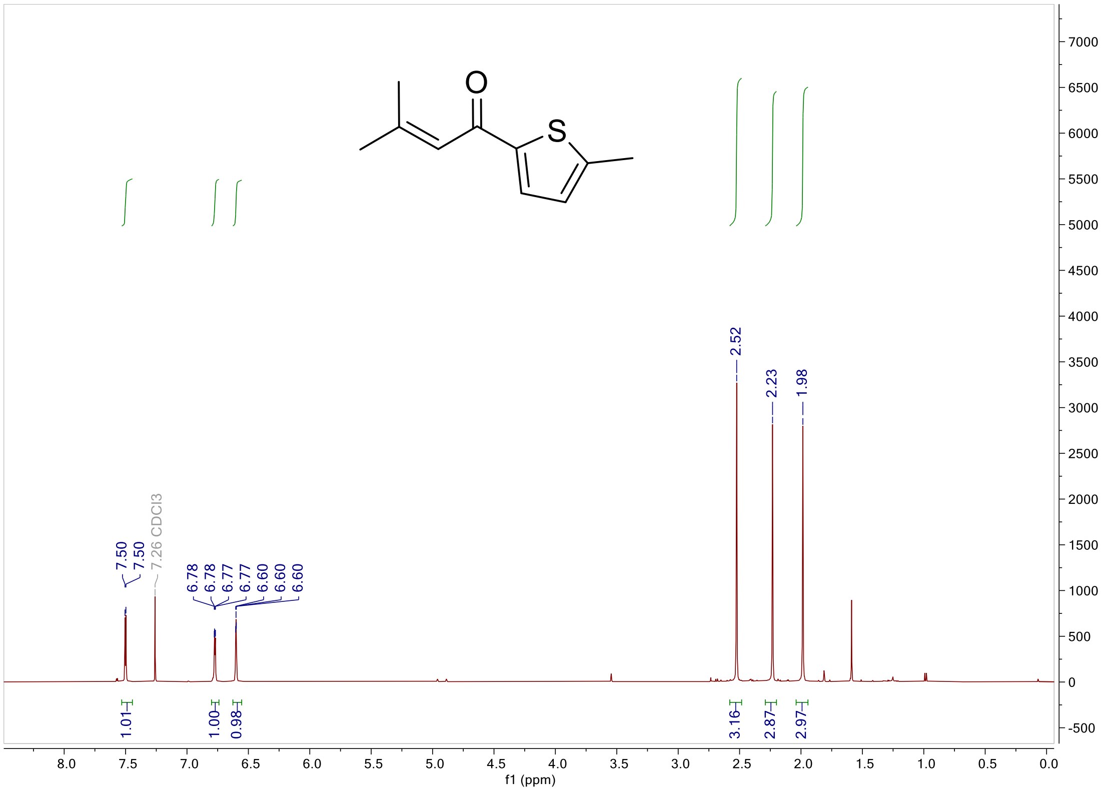

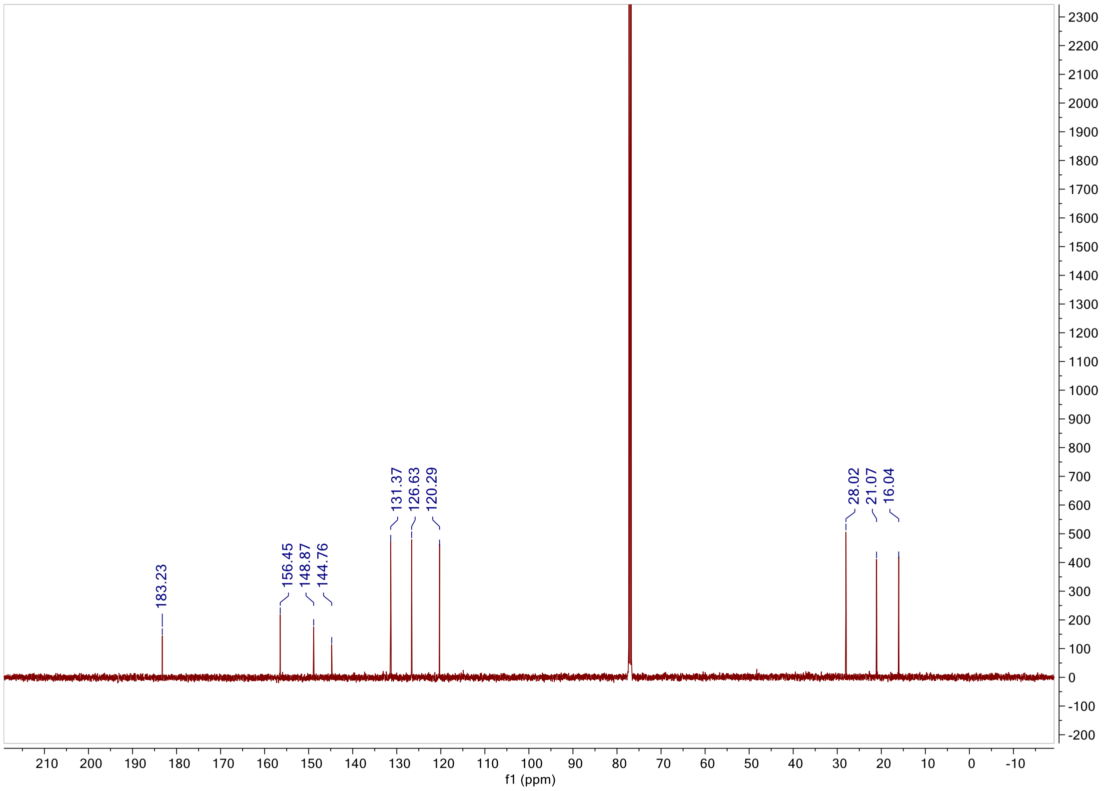

NOESY data for 3k ($CDCl_3$).

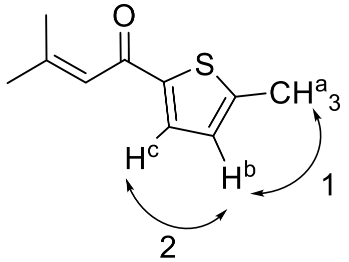

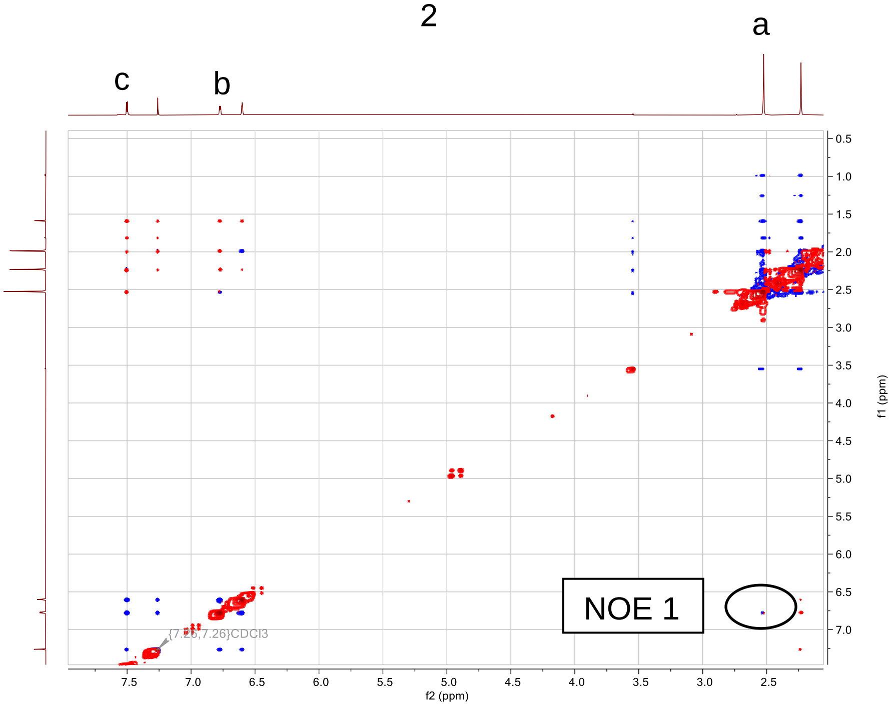

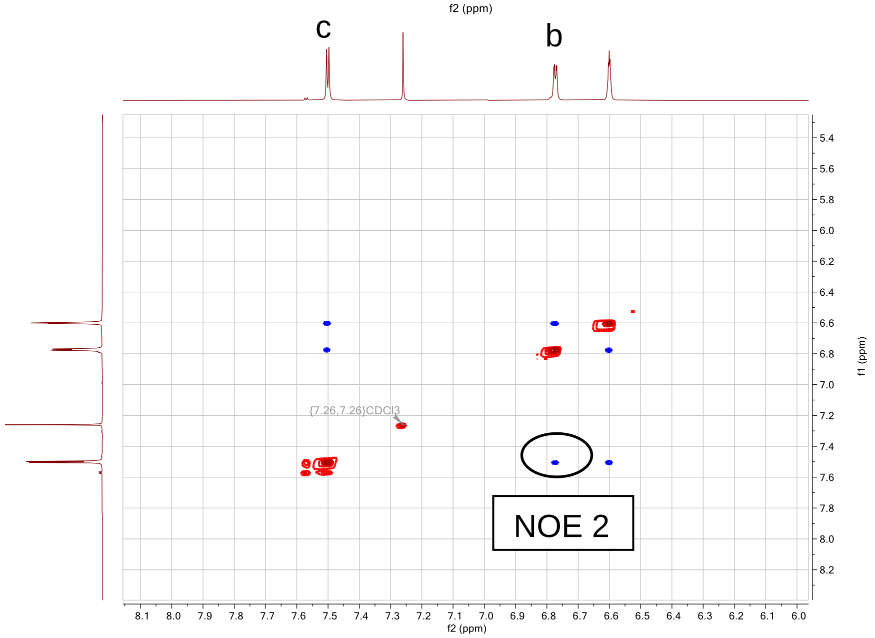

^{1}H and ^{13}C NMR data for 3l.

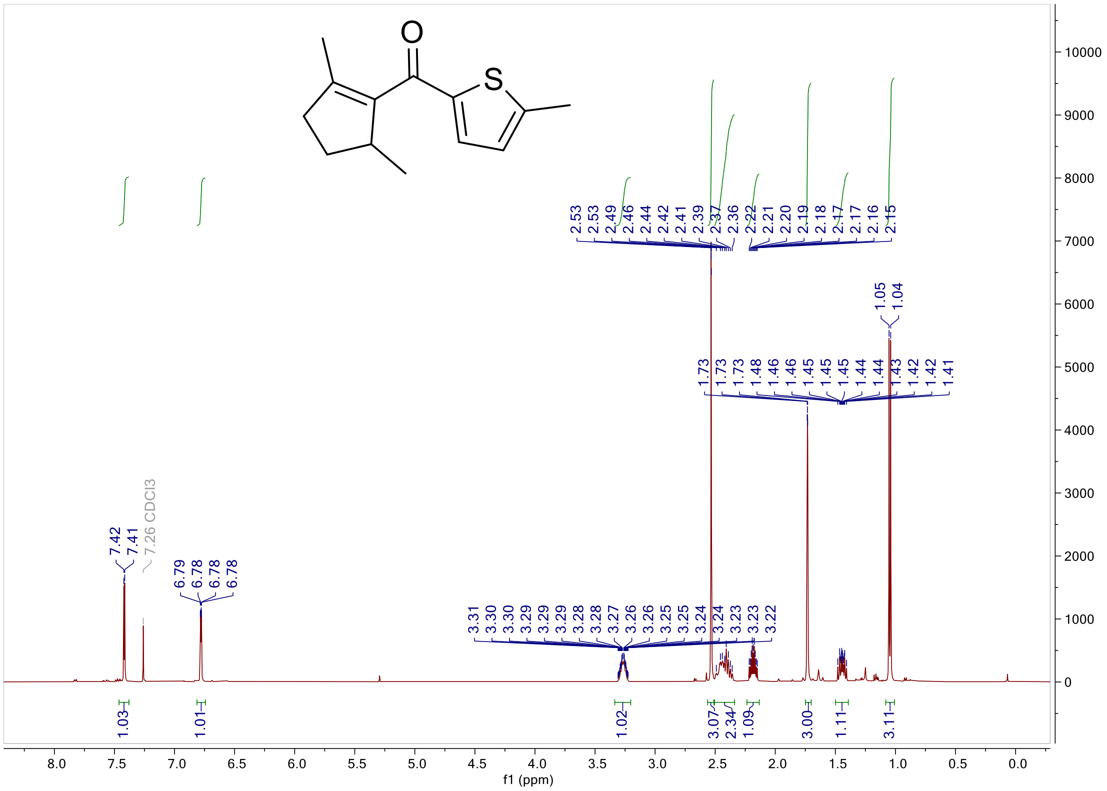

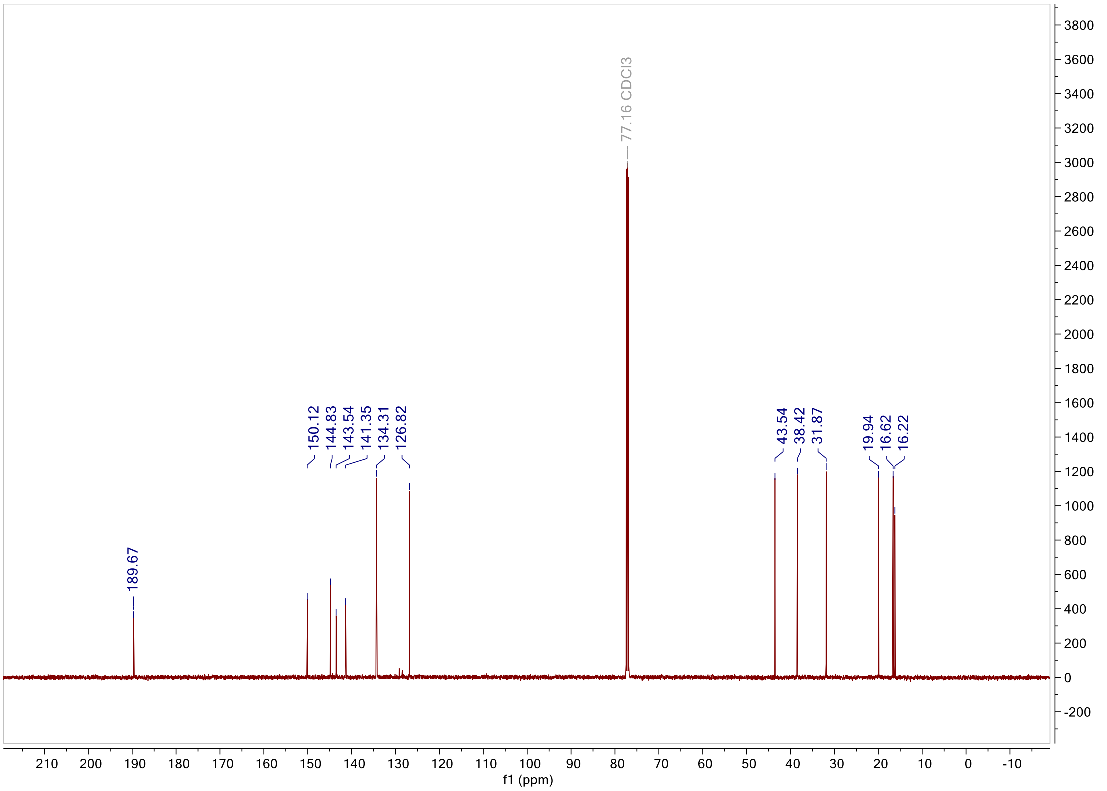

NOESY data for 3l ($CDCl_3$).

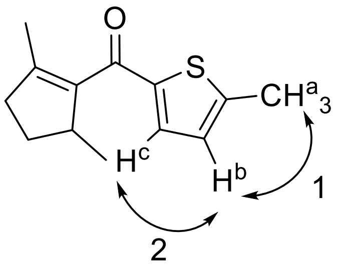

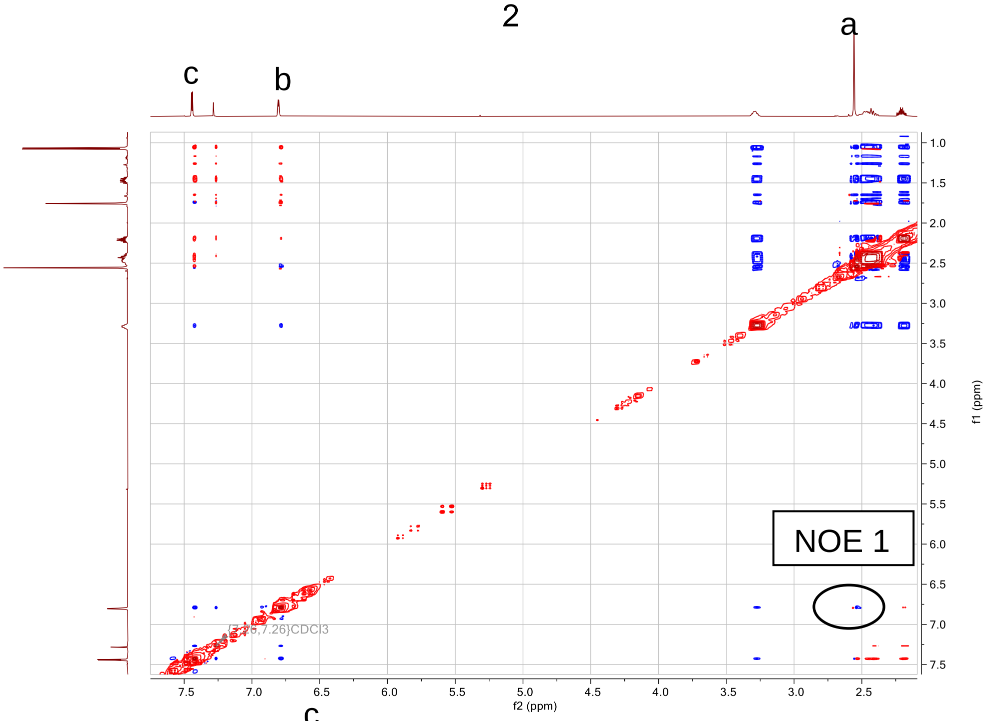

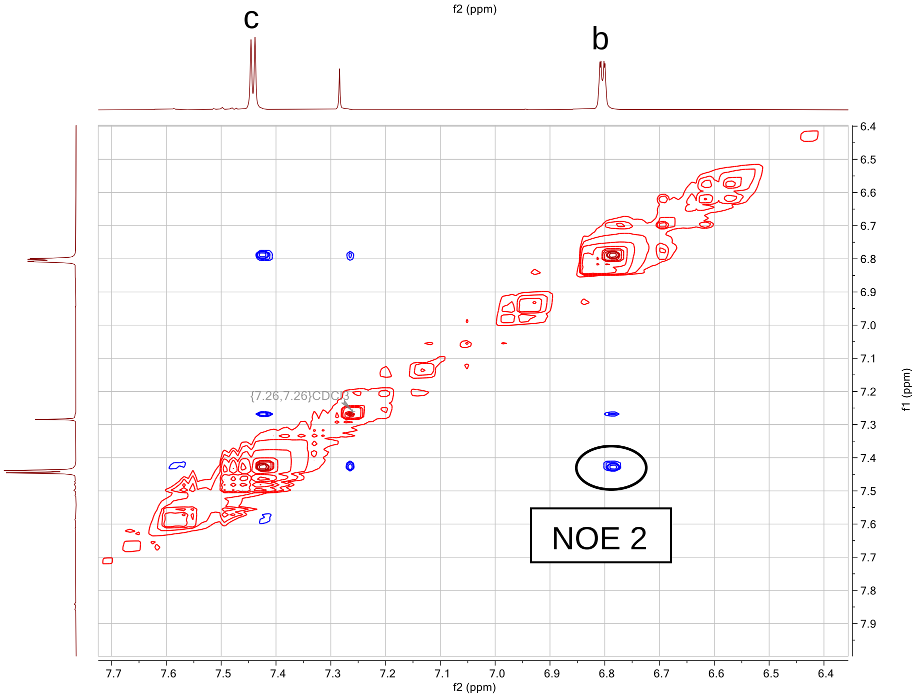

^{1}H and ^{13}C NMR data for 3m.

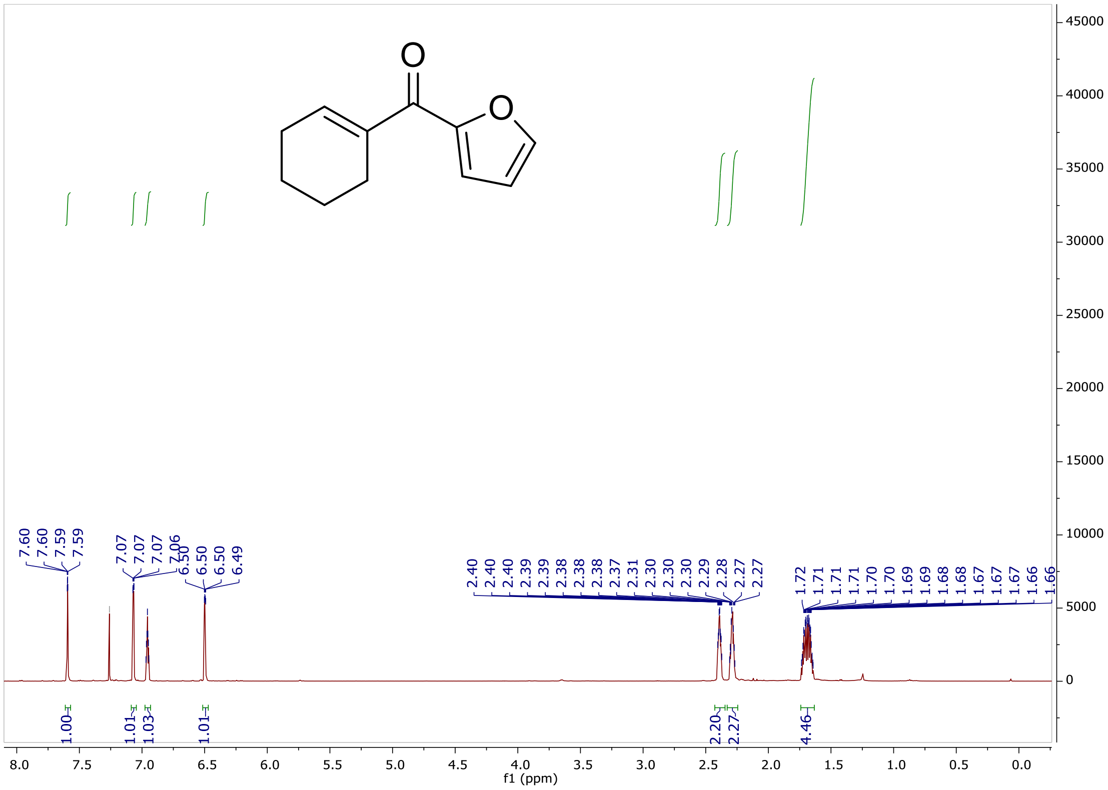

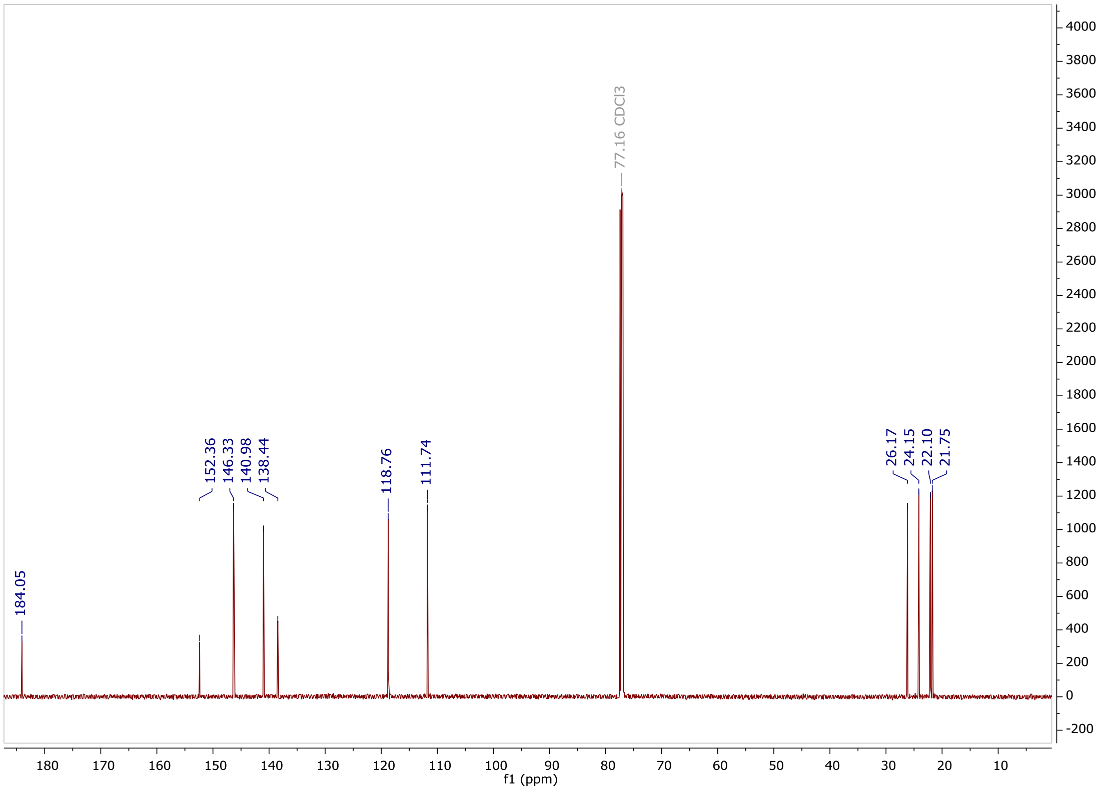

^{1}H and ^{13}C NMR data for 3n.

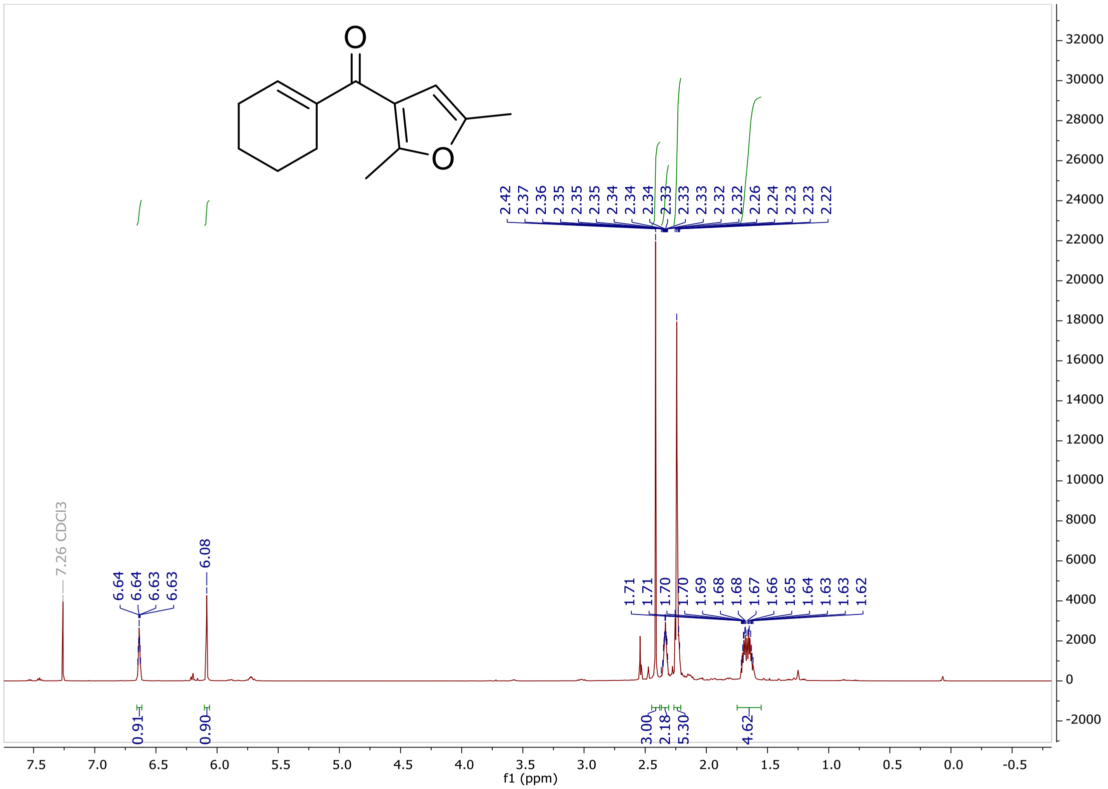

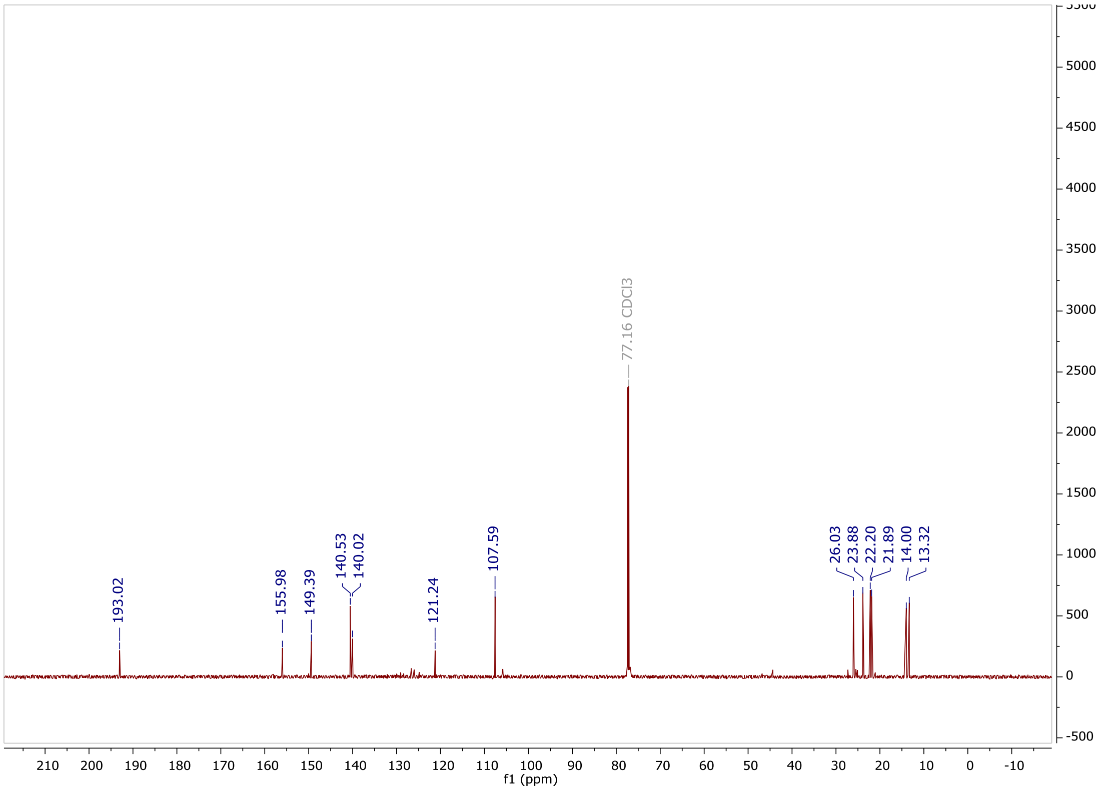

^{1}H and ^{13}C NMR data for 3o.

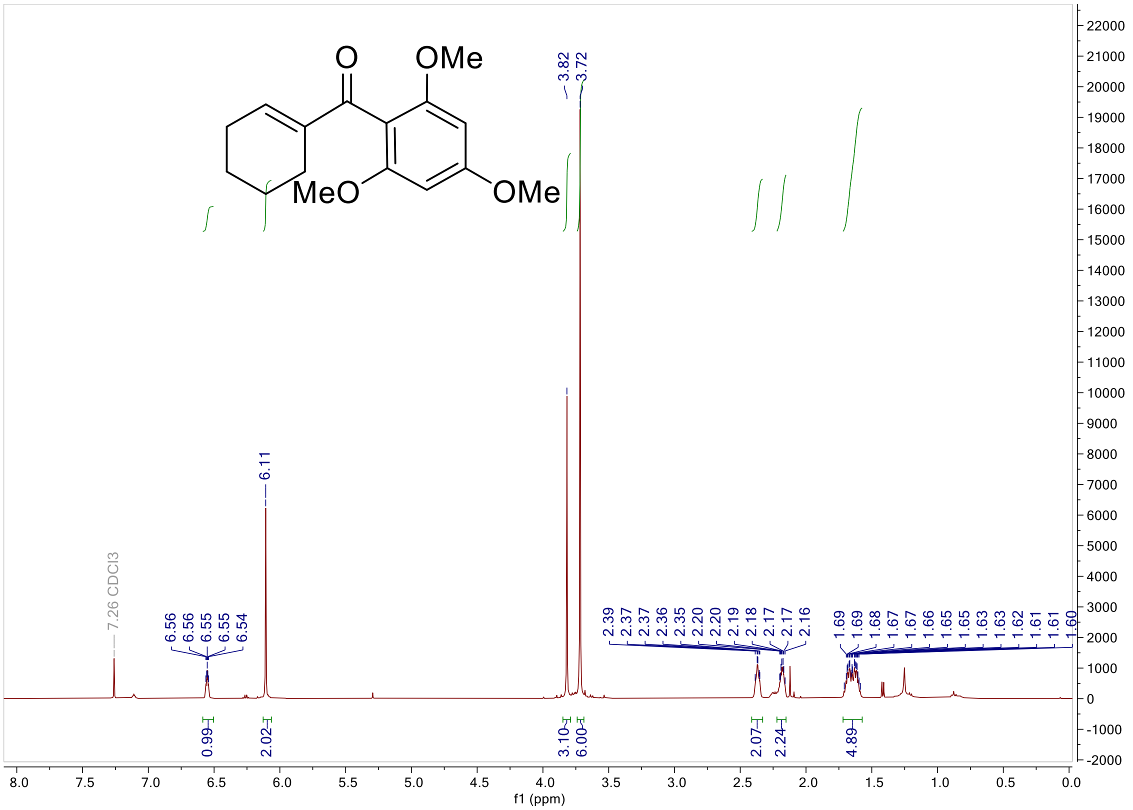

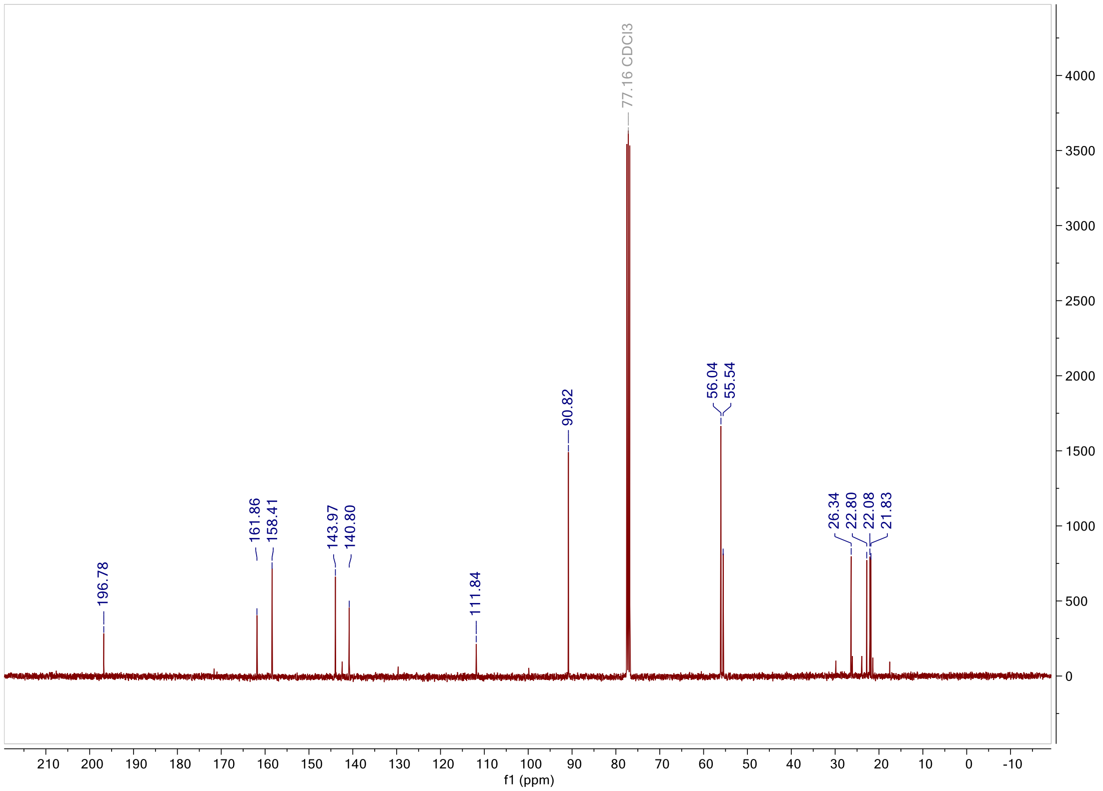

^{1}H and ^{13}C NMR data for 3p.

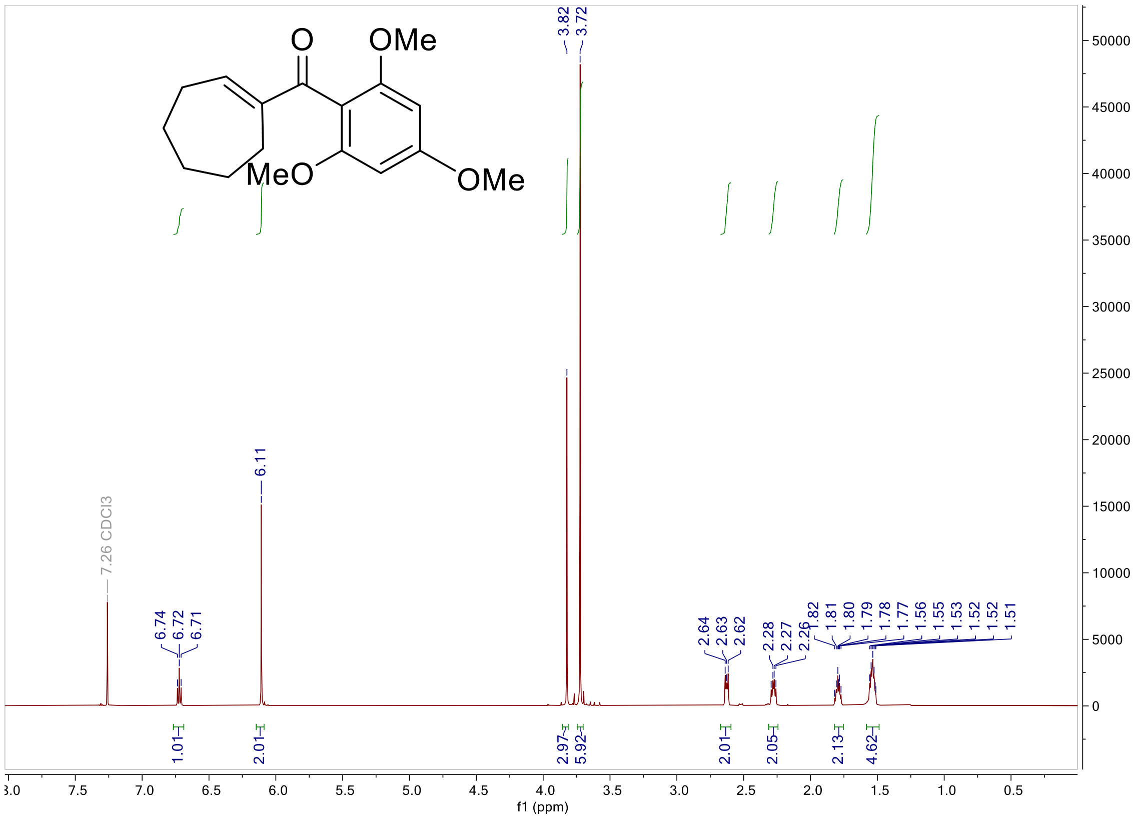

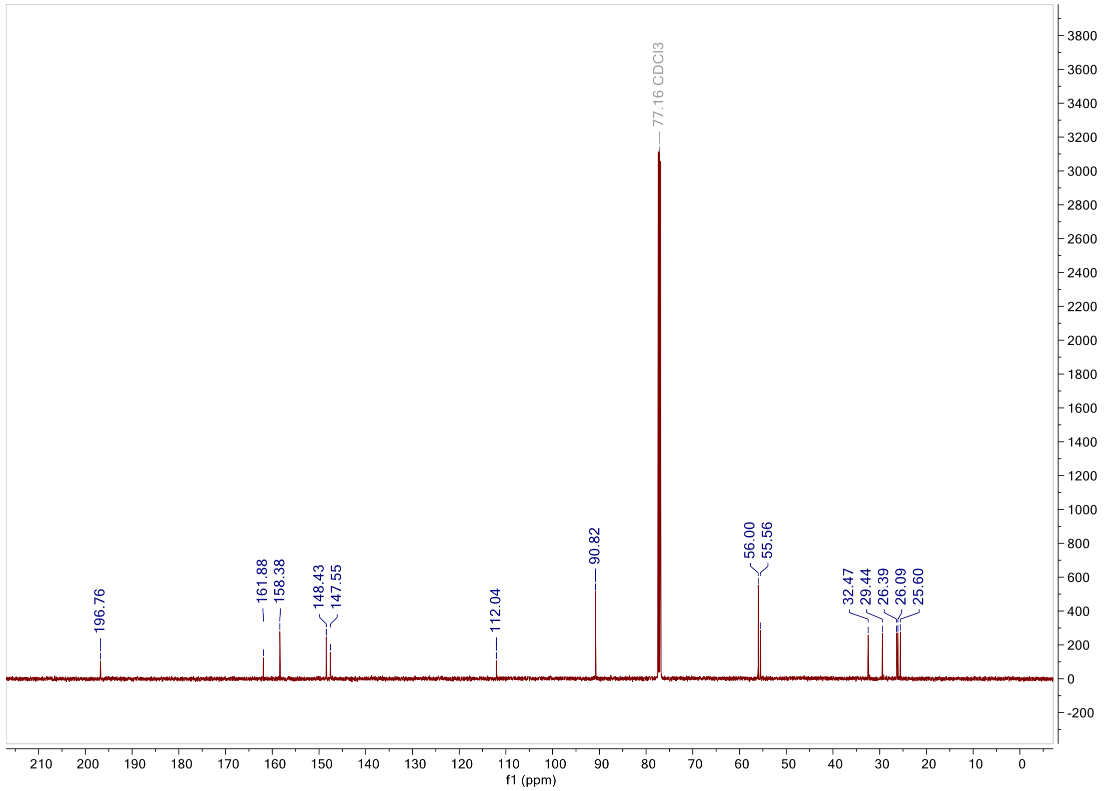

^{1}H and ^{13}C NMR data for 3q.

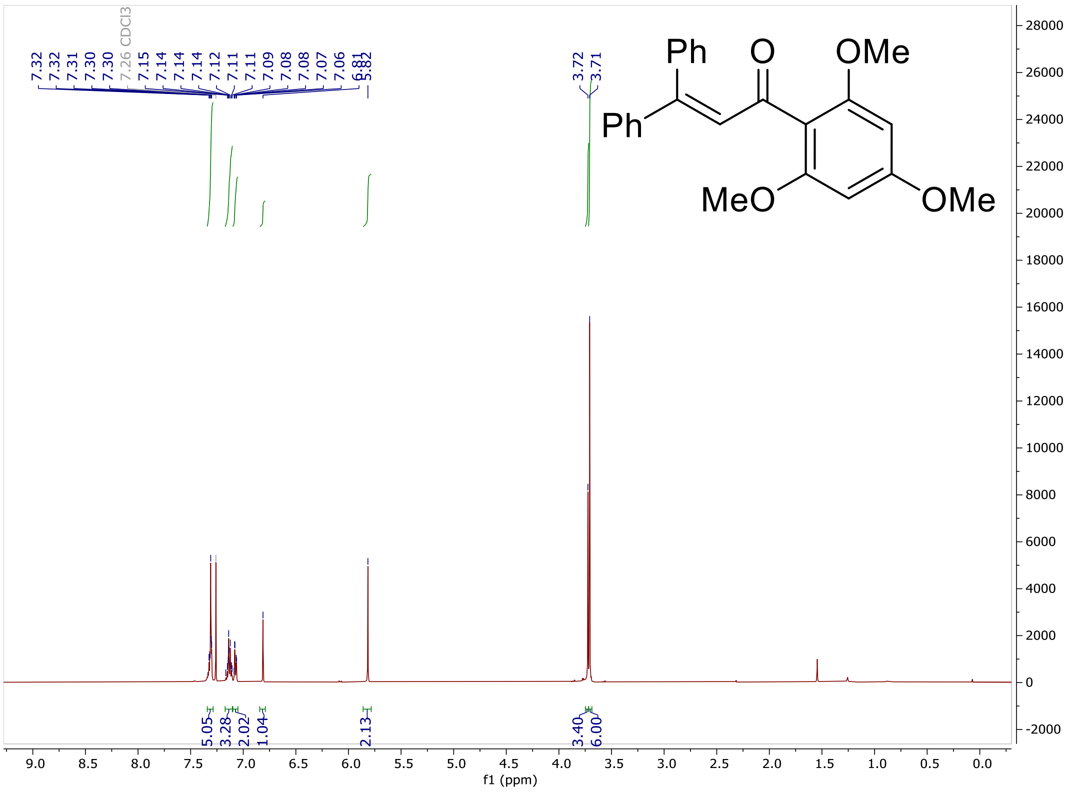

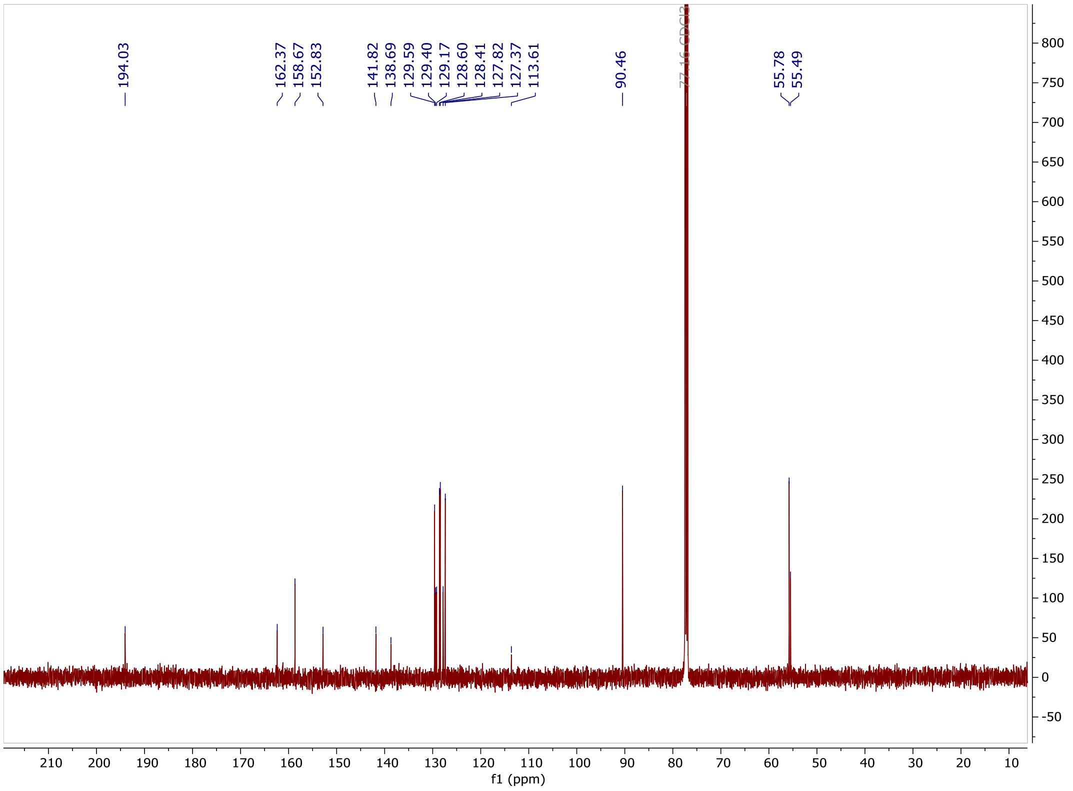

^{1}H and ^{13}C NMR data for 3r.

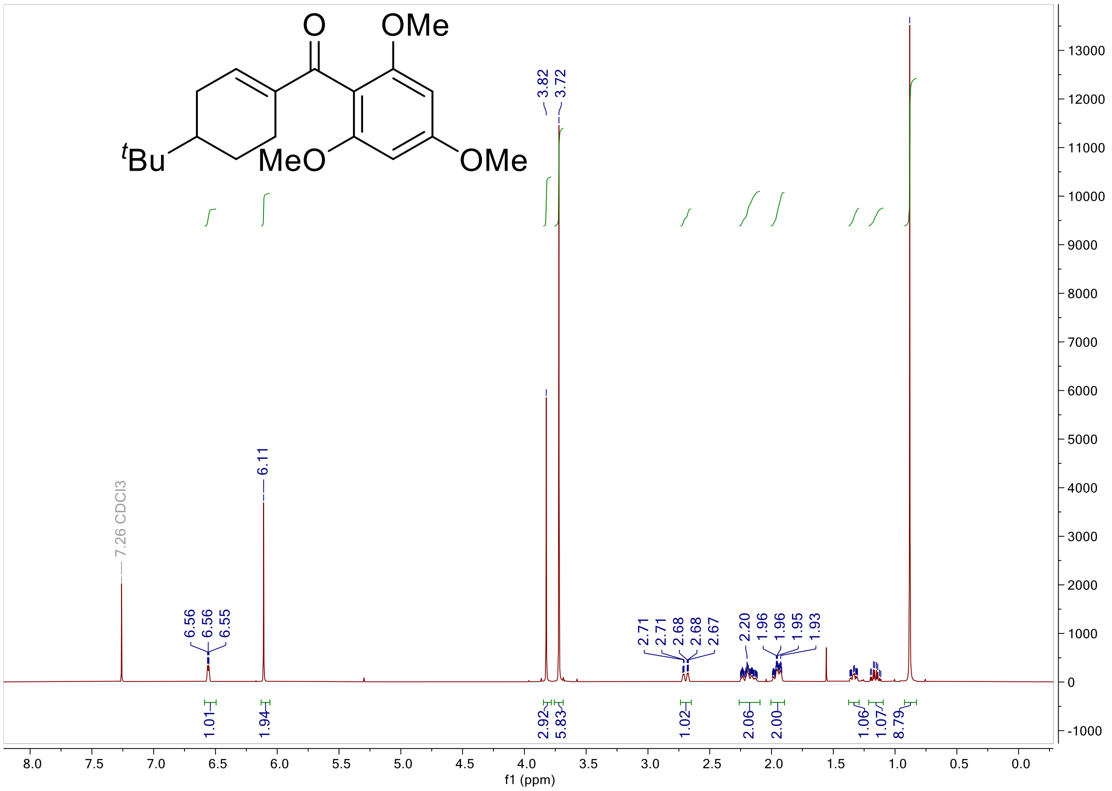

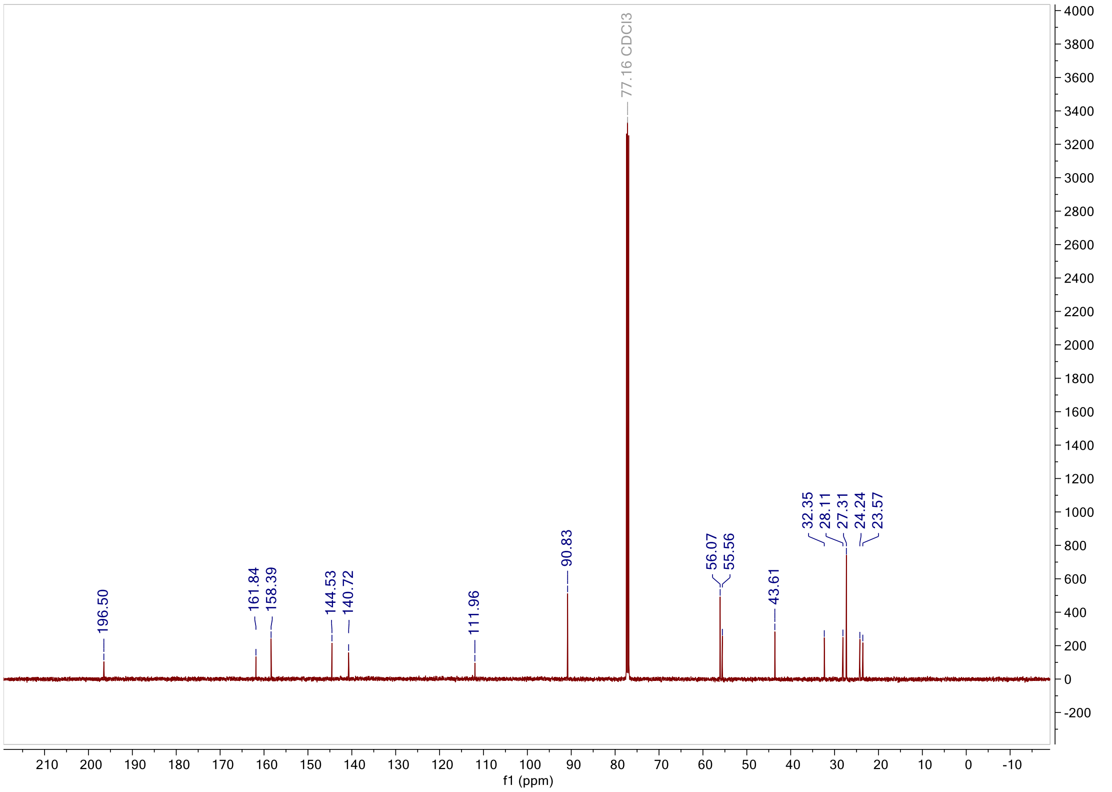

^{1}H and ^{13}C NMR data for 3s.

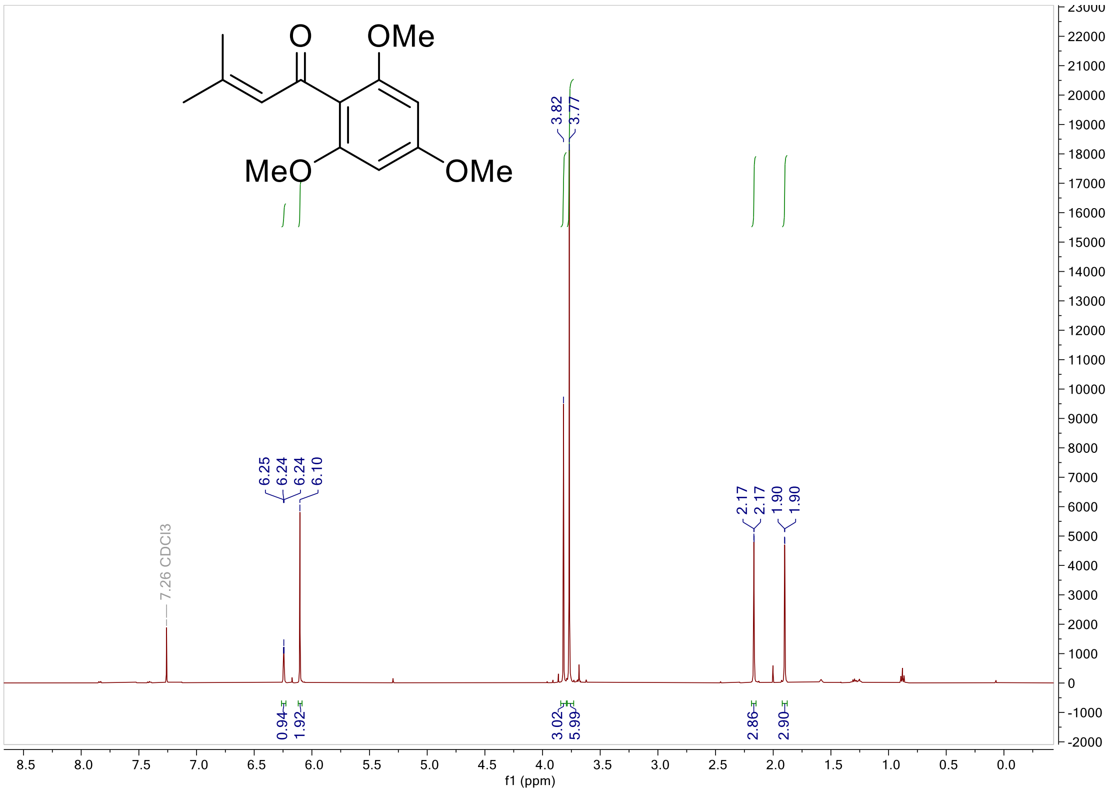

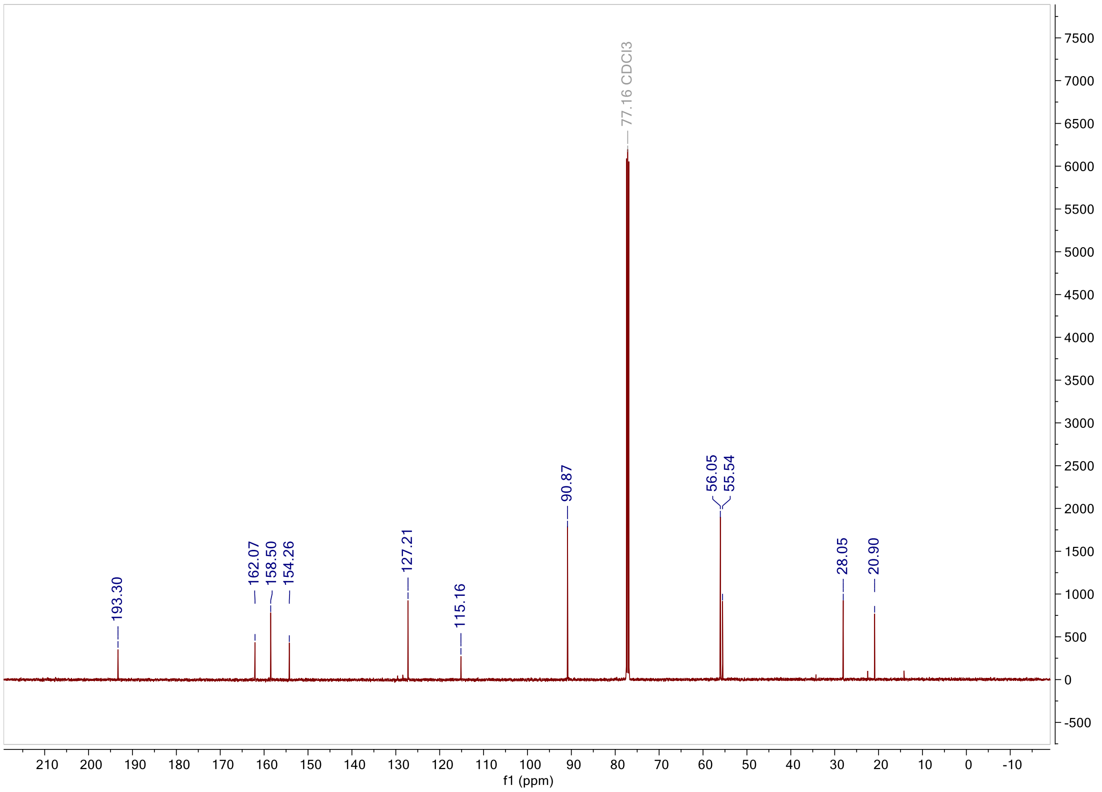

^{1}H and ^{13}C NMR data for 3t.

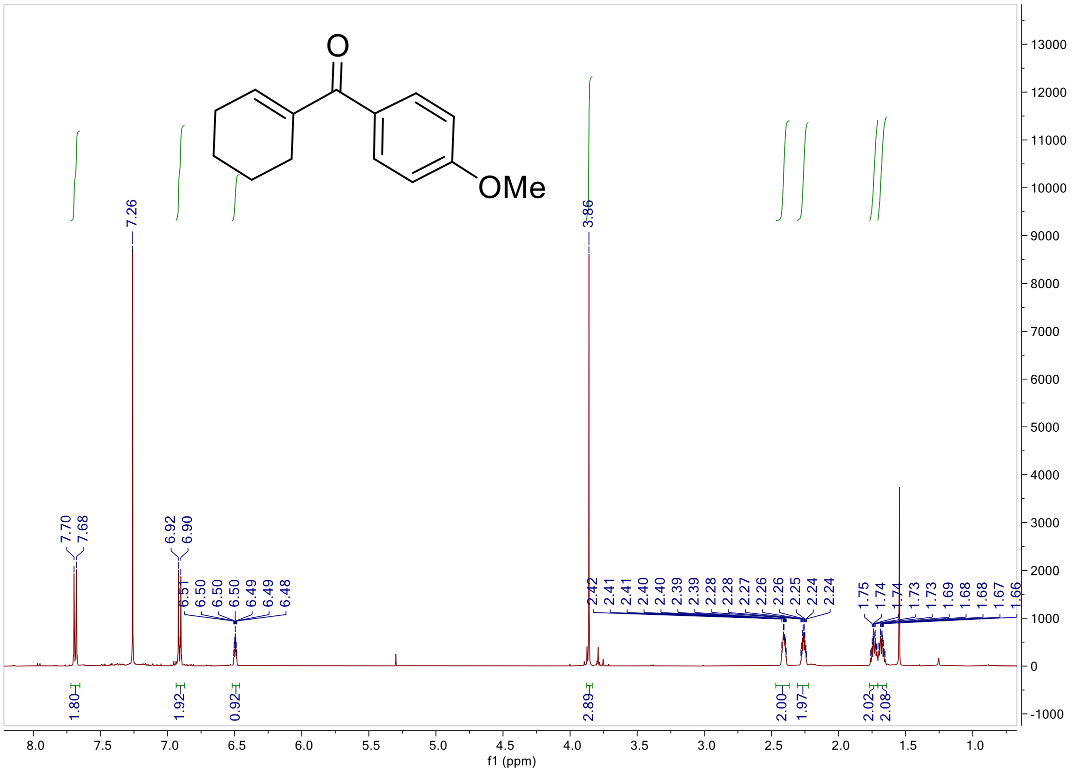

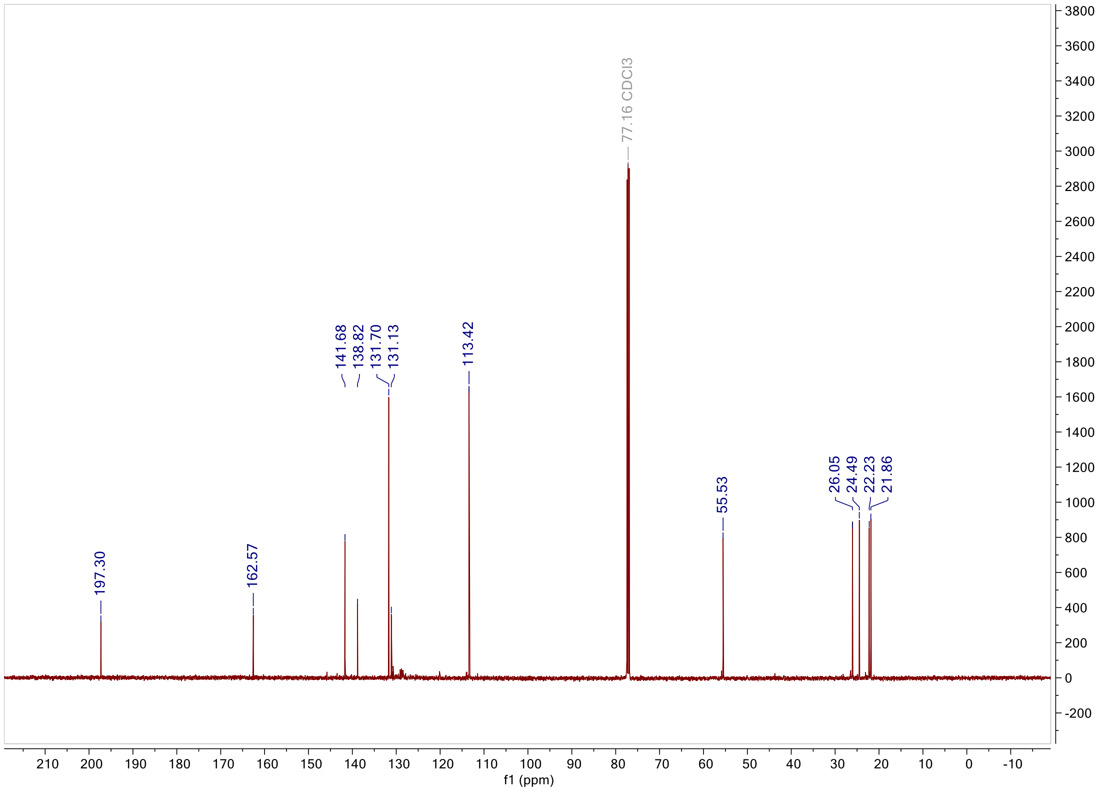

^{1}H and ^{13}C NMR data for 3u.

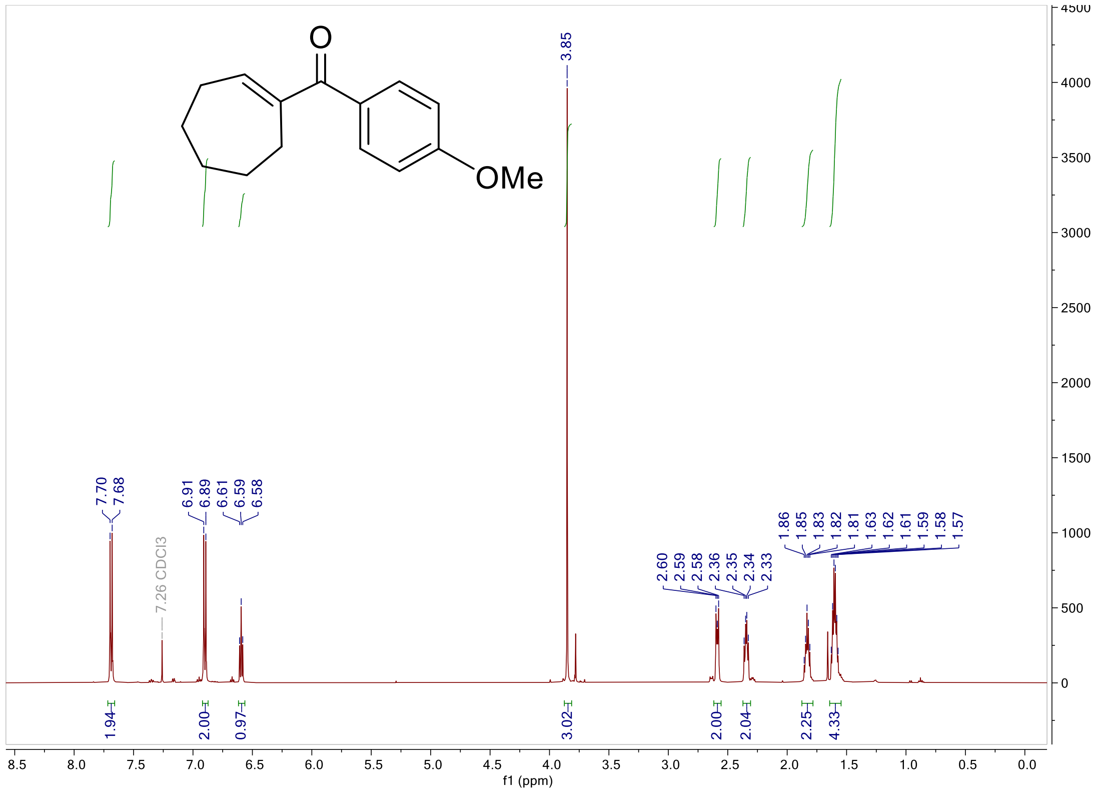

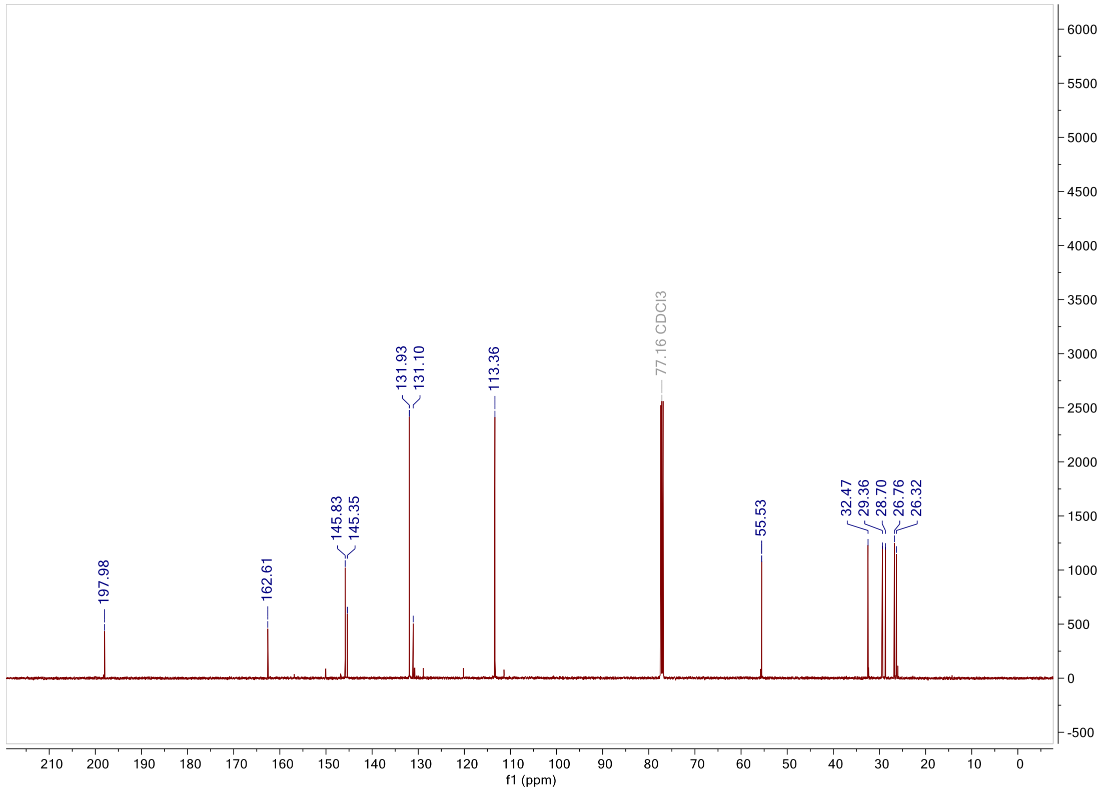

^{1}H and ^{13}C NMR data for 3v.

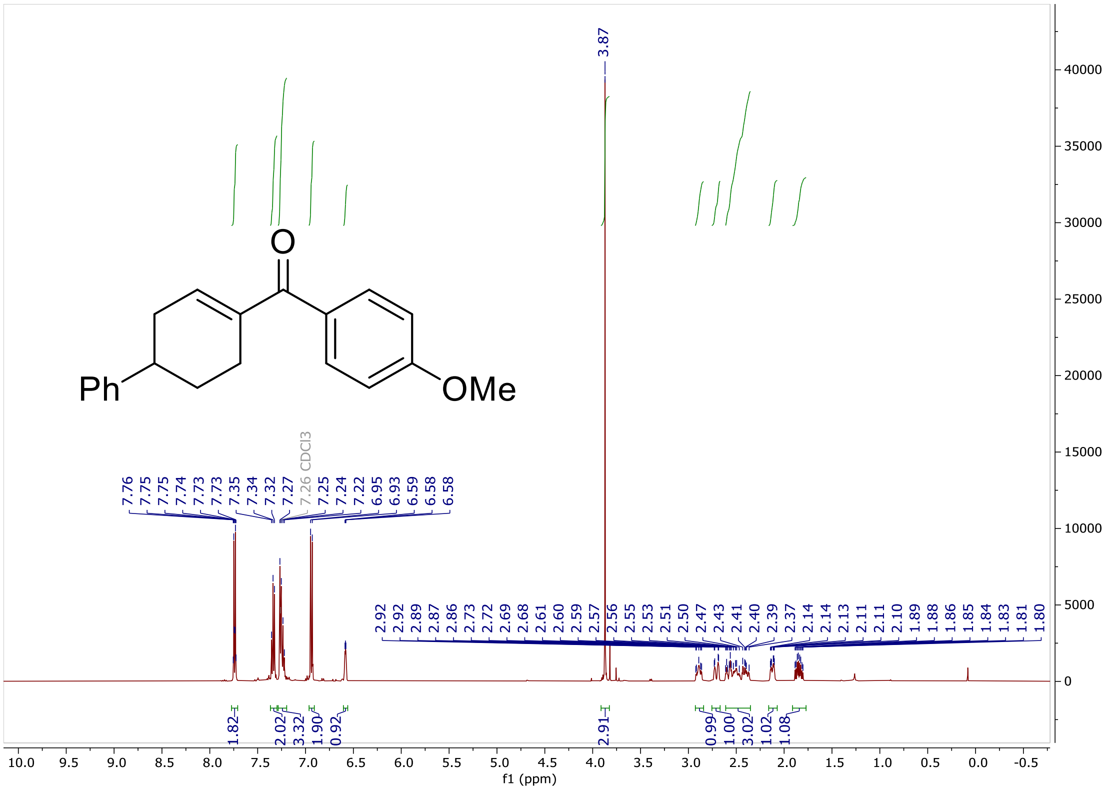

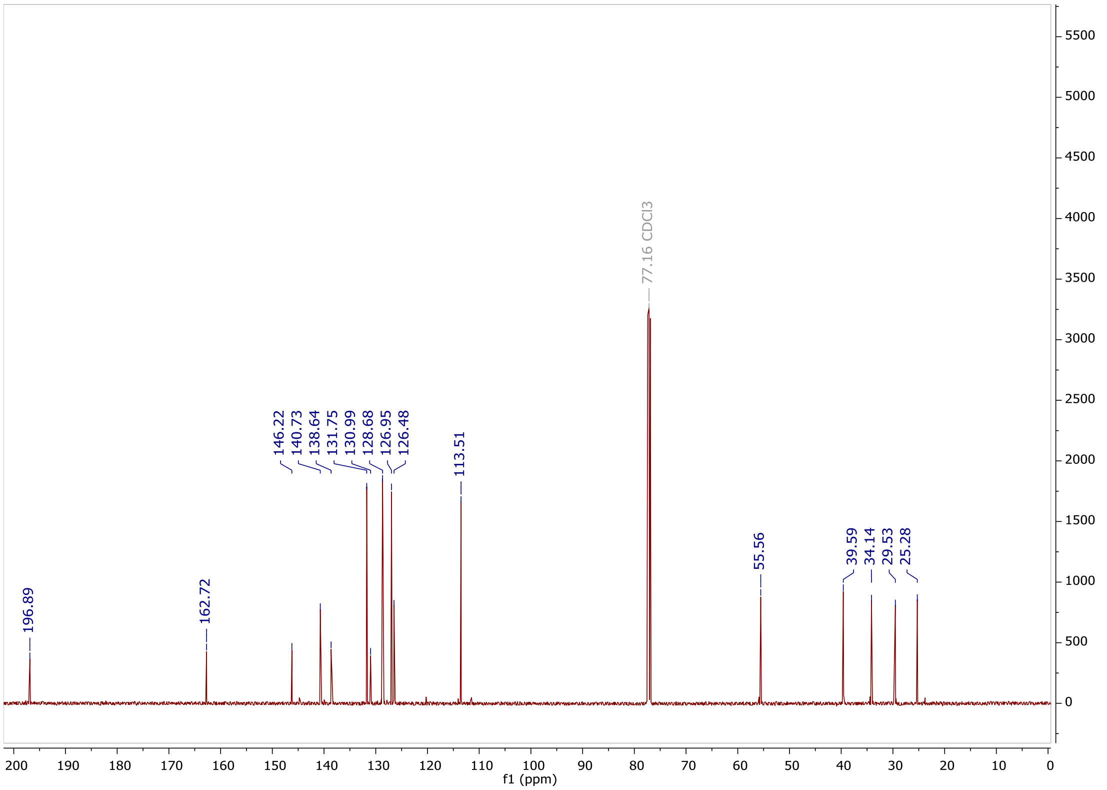

^{1}H and ^{13}C NMR data for 3w.

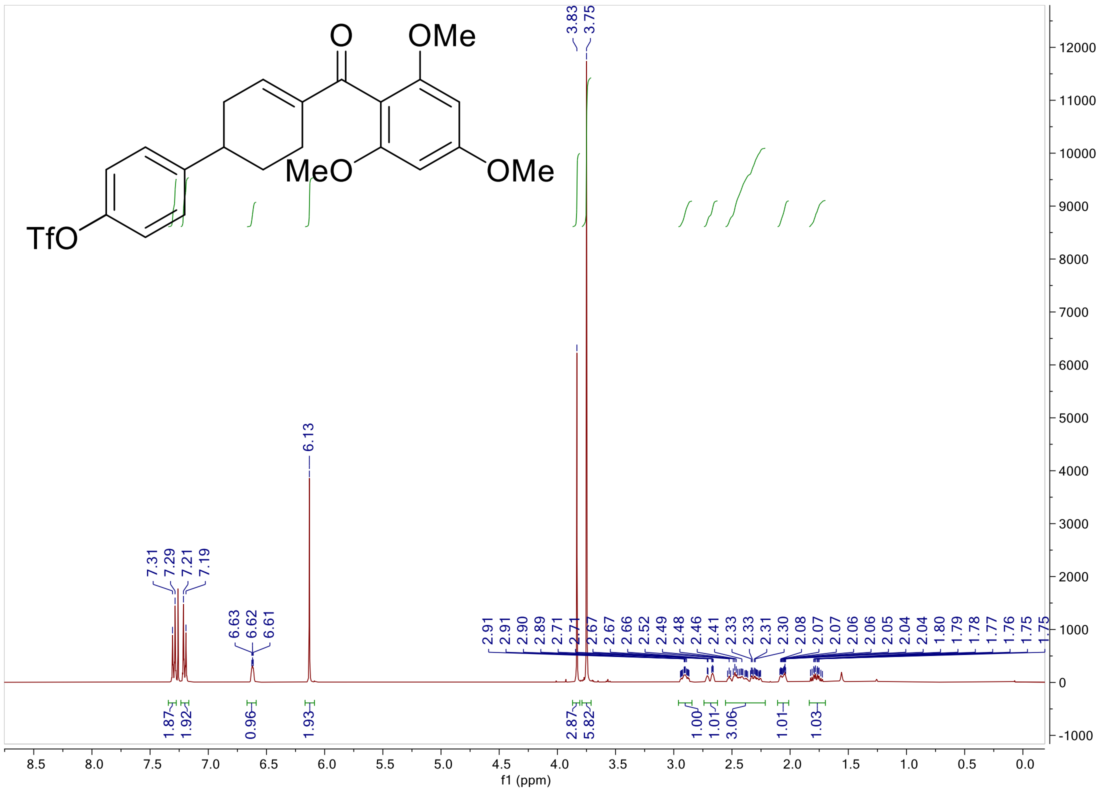

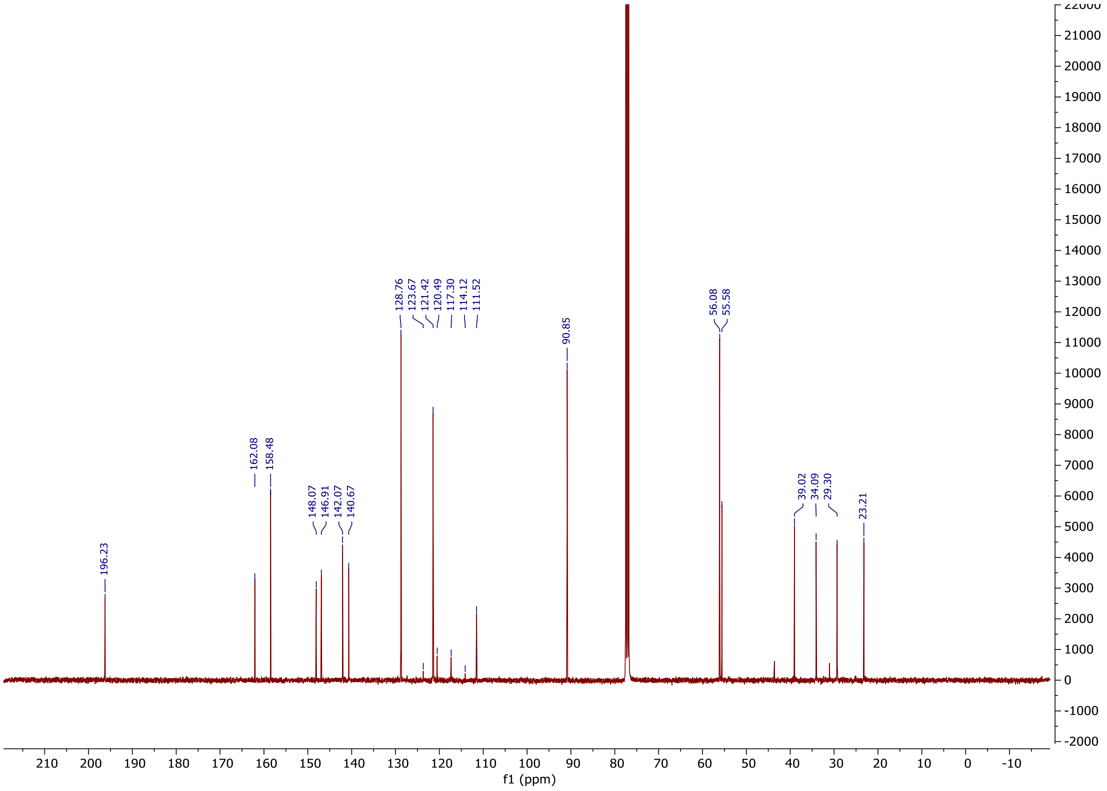

^{1}H and ^{13}C NMR data for 2-3x.

^{1}H NMR (CD_3CN):

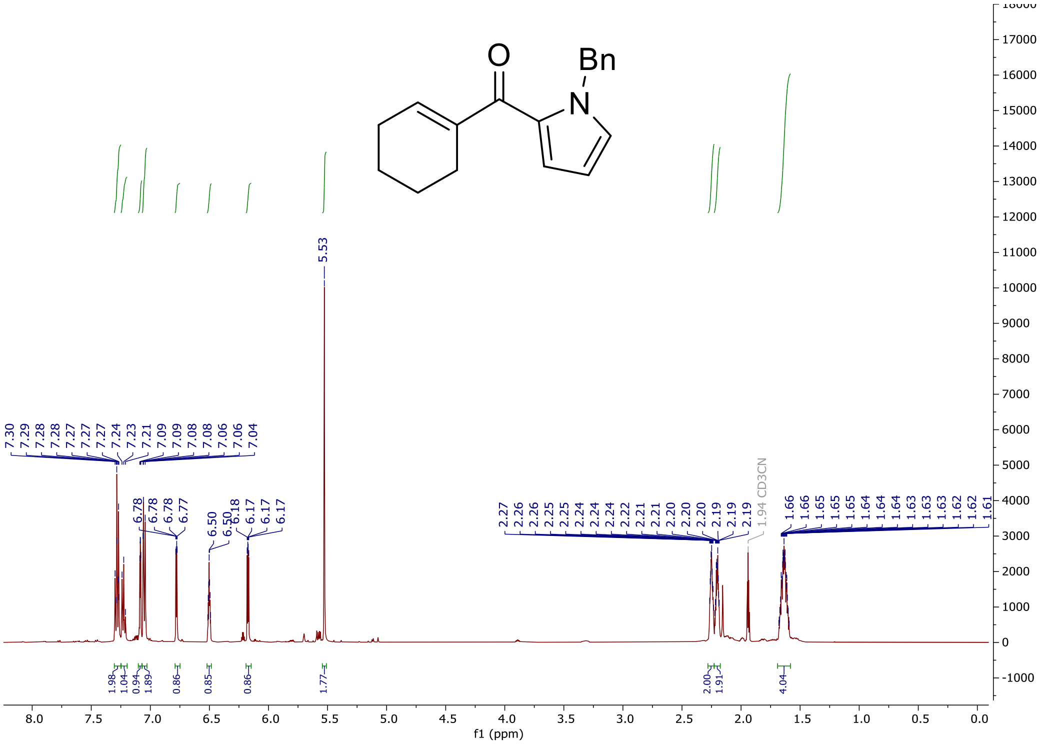

^{1}H NMR ($CDCl_3$):

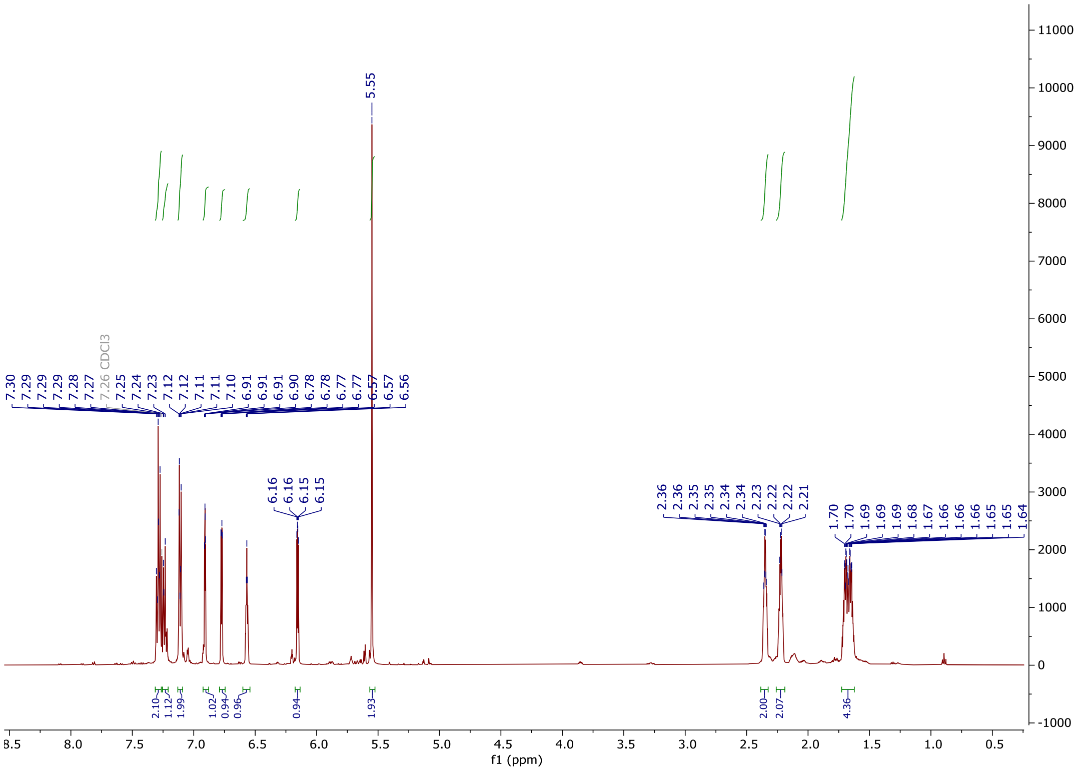

^{13}C NMR ($CDCl_3$):

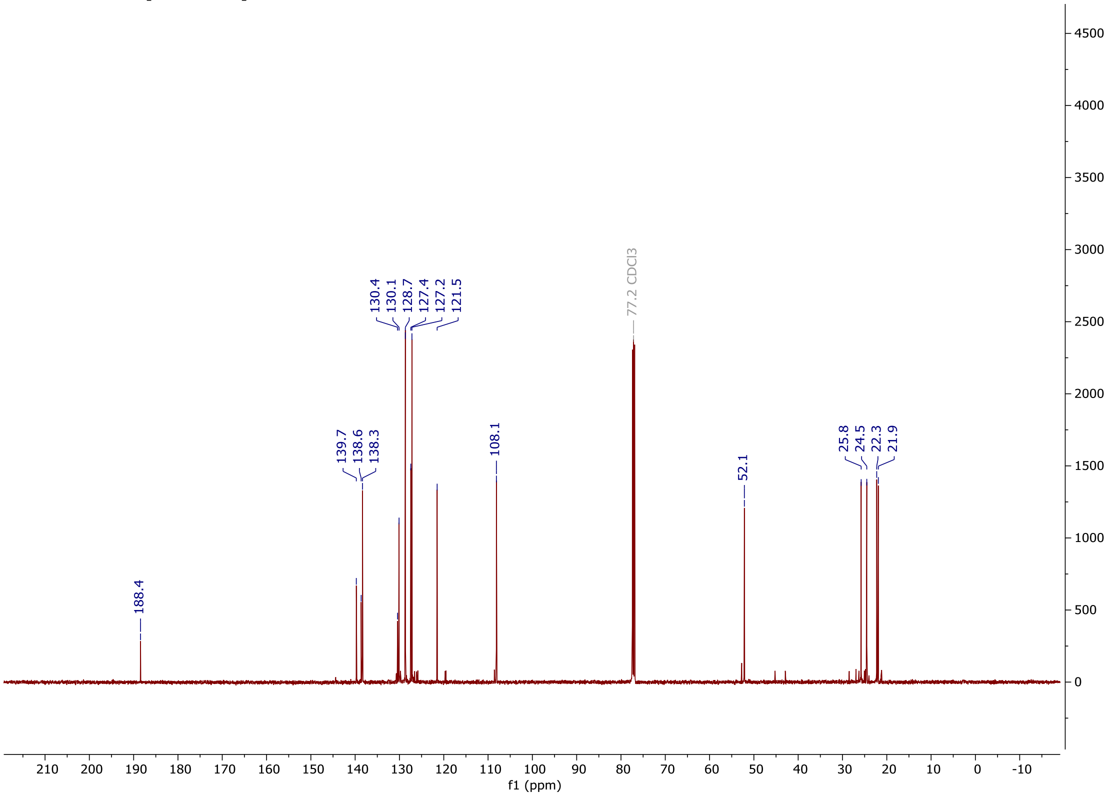

NOESY data for 2-3x.

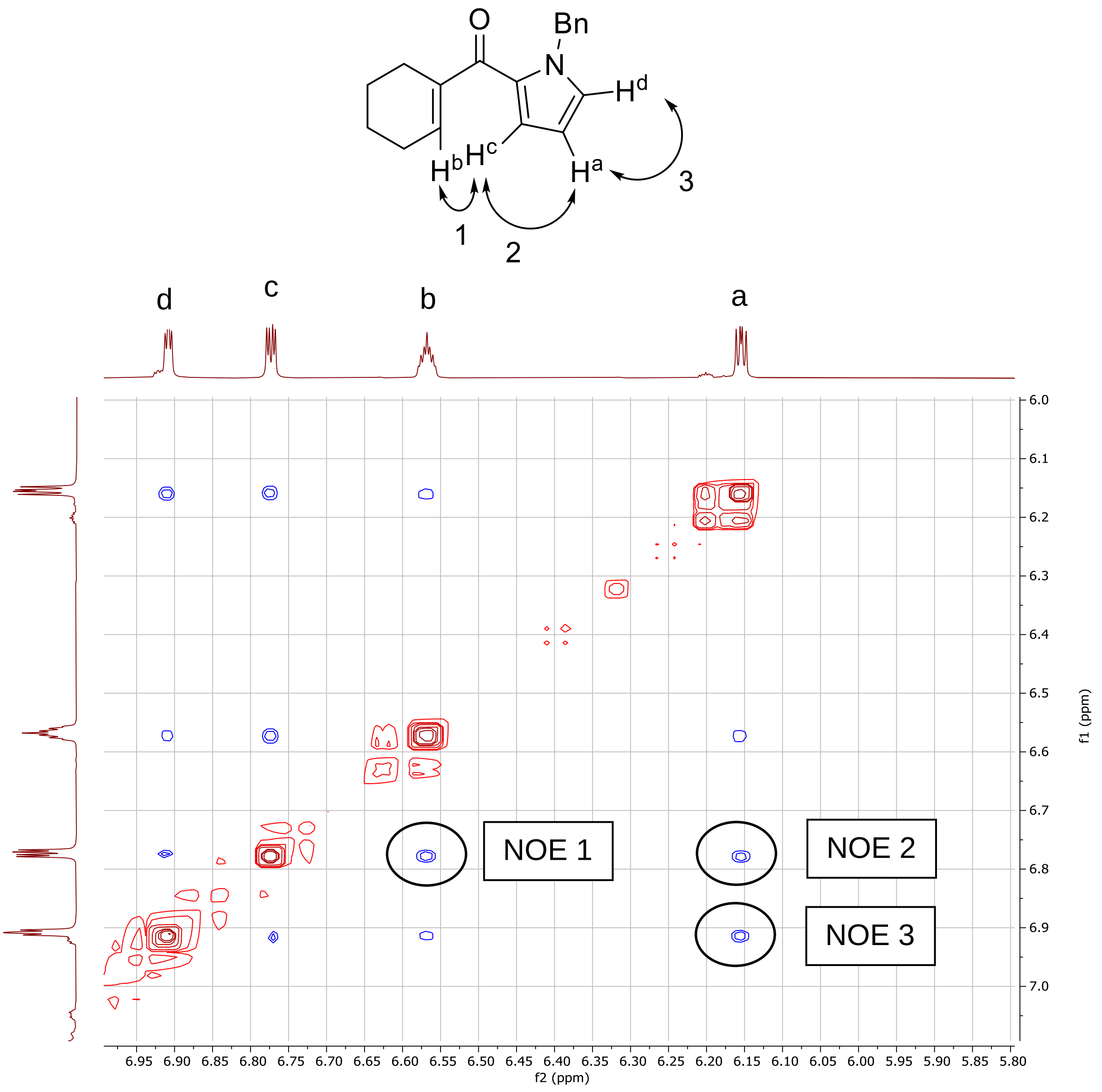

^{1}H and ^{13}C NMR data for 3-3x.

^{1}H NMR (CD_3CN):

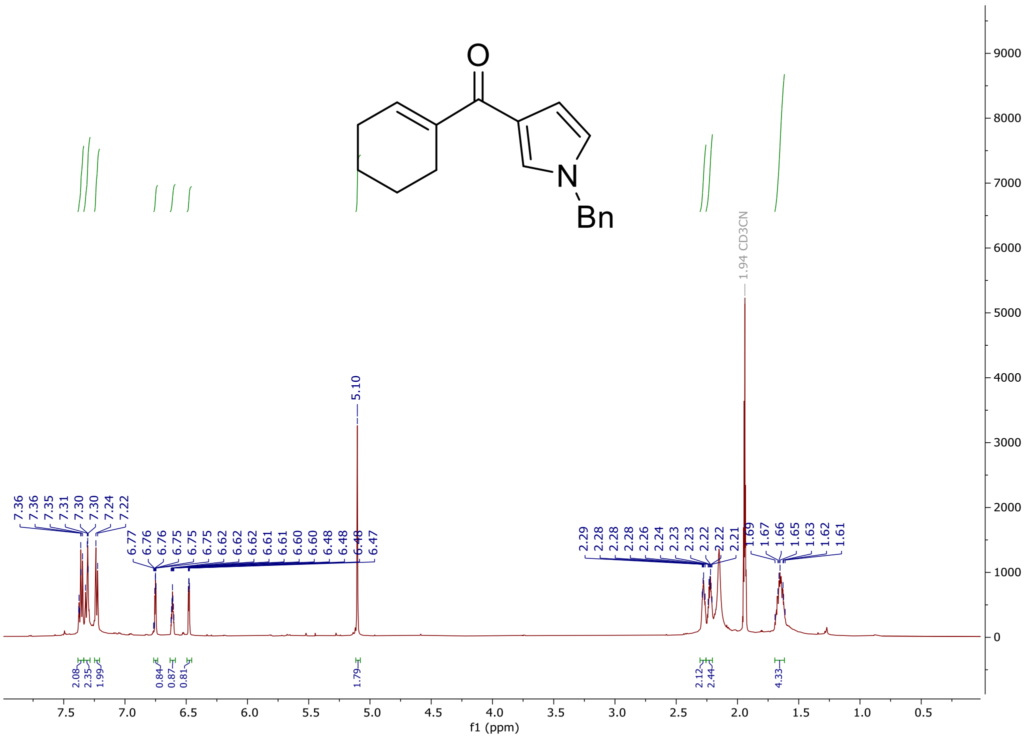

^{1}H NMR ($CDCl_3$):

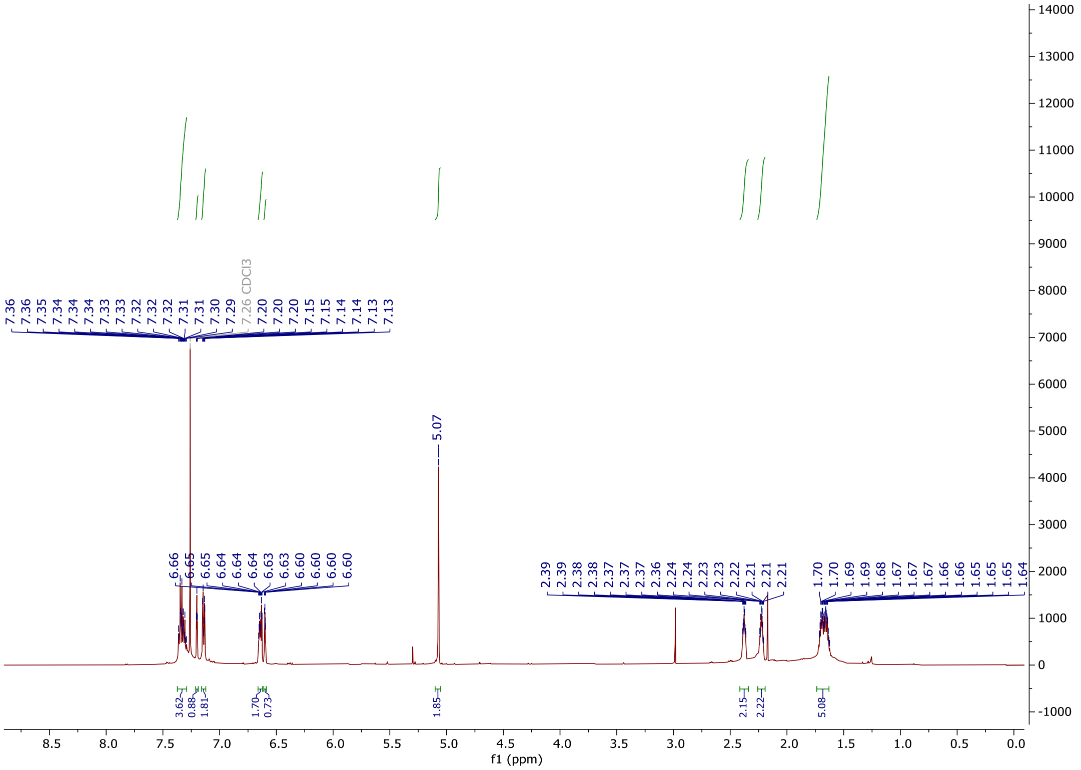

^{13}C NMR ($CDCl_3$):

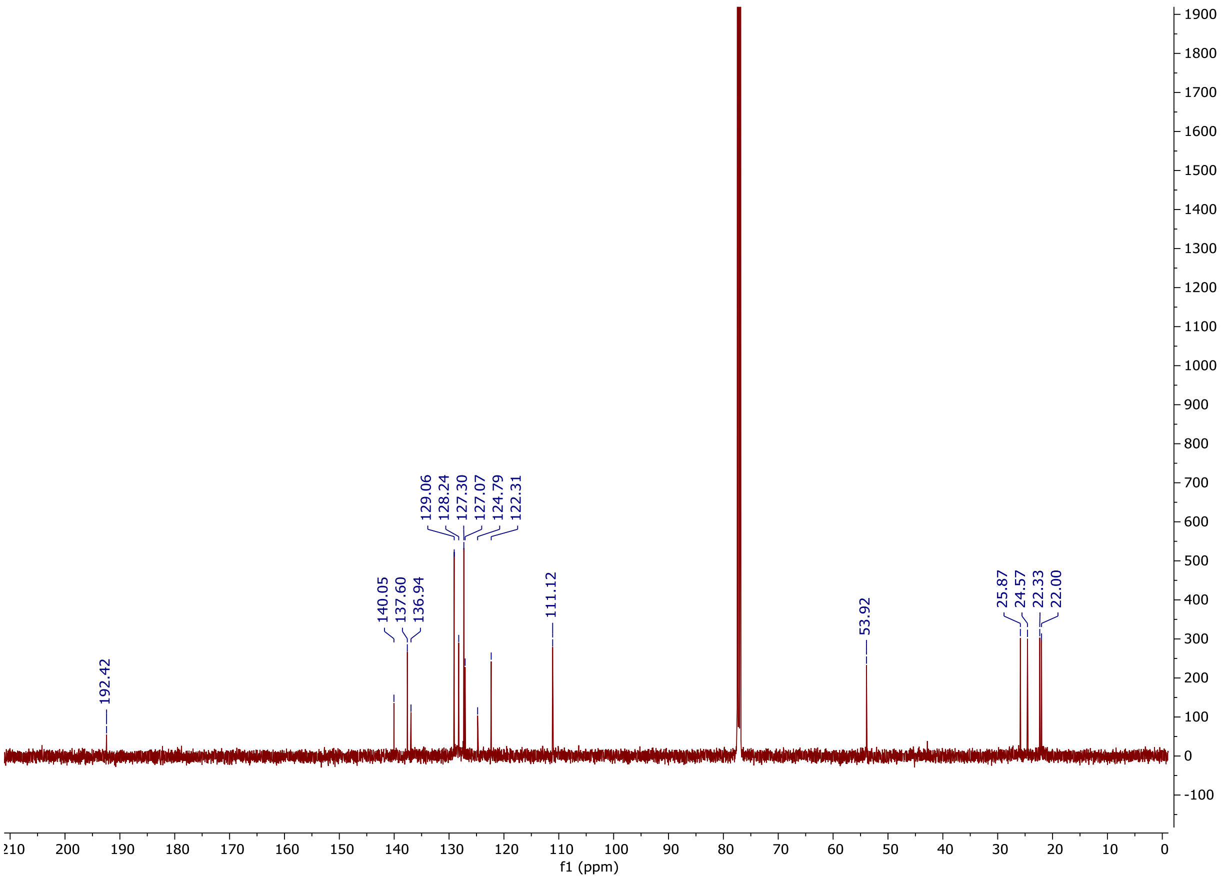

^{1}H and ^{13}C NMR data for 4a.

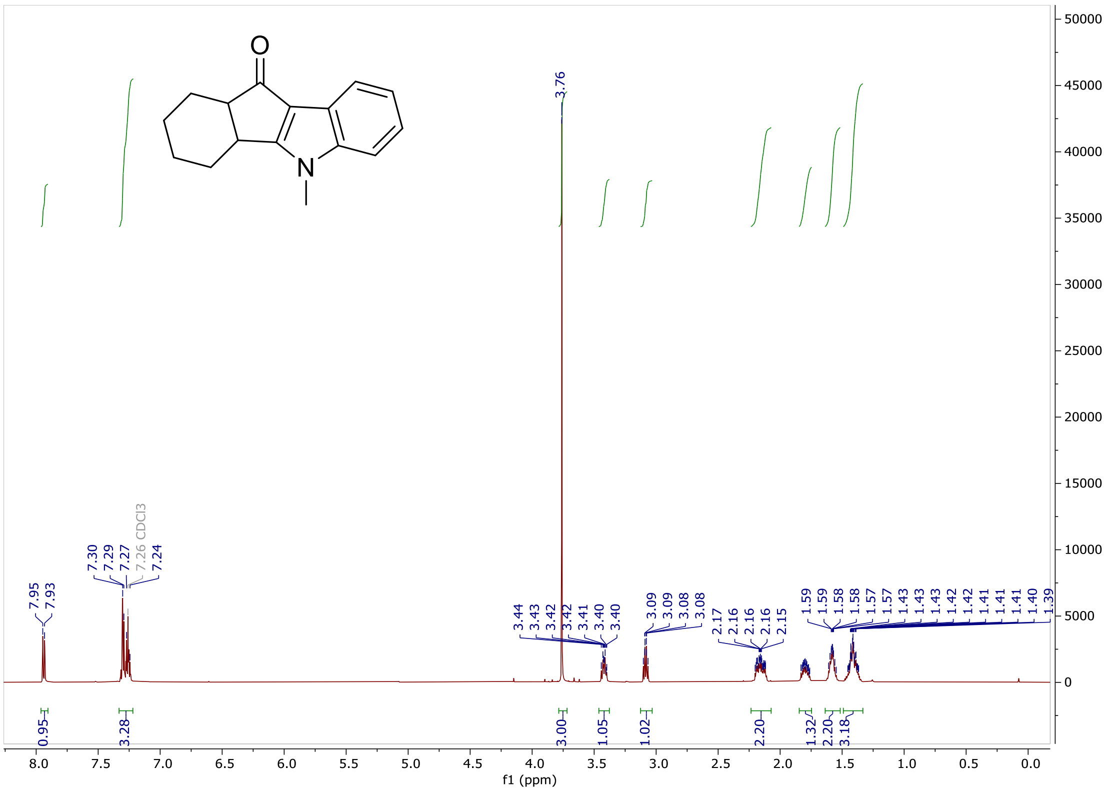

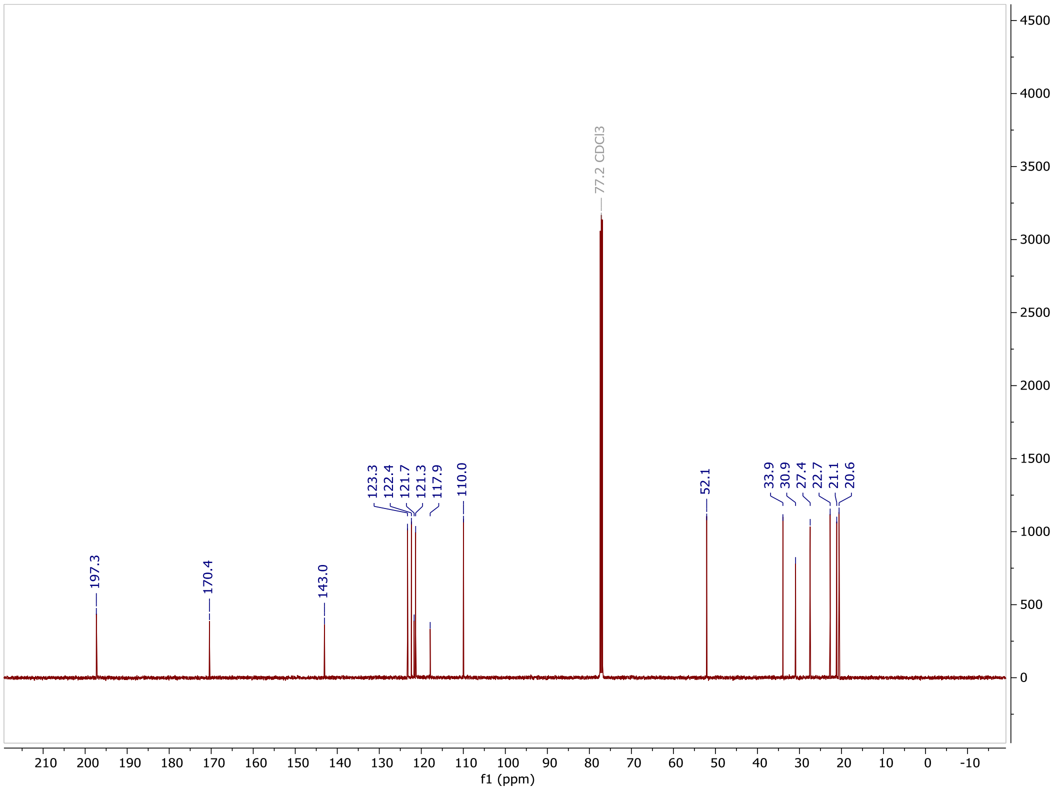

^{1}H and ^{13}C NMR data for 4b.

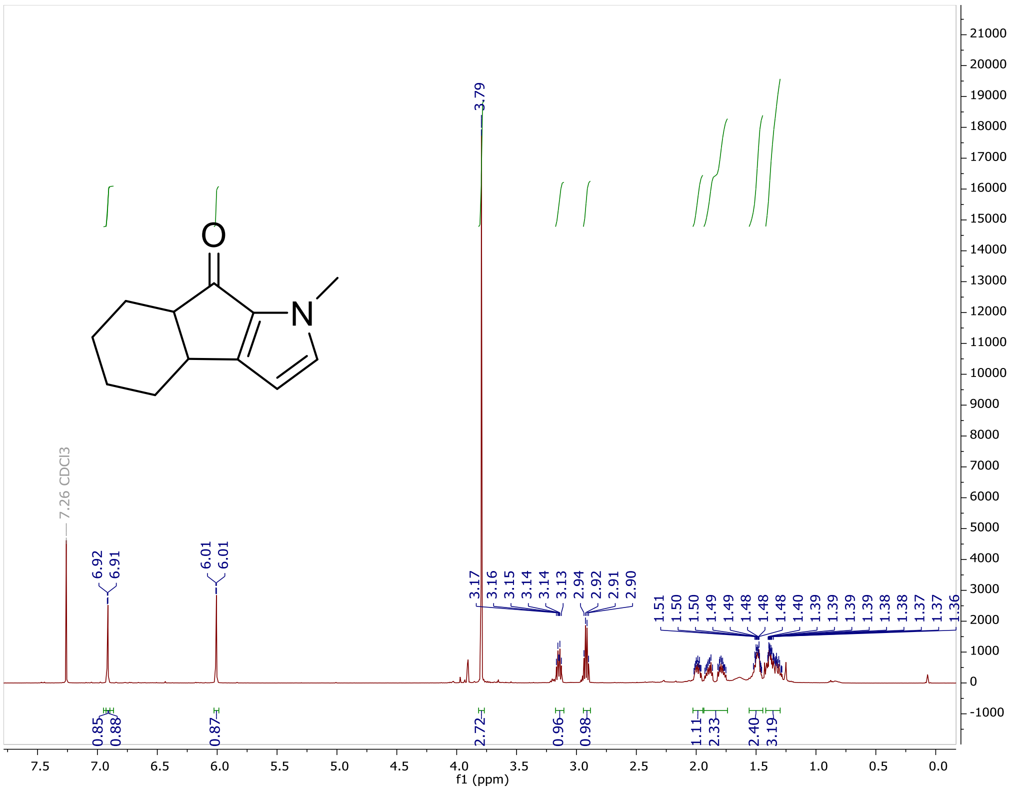

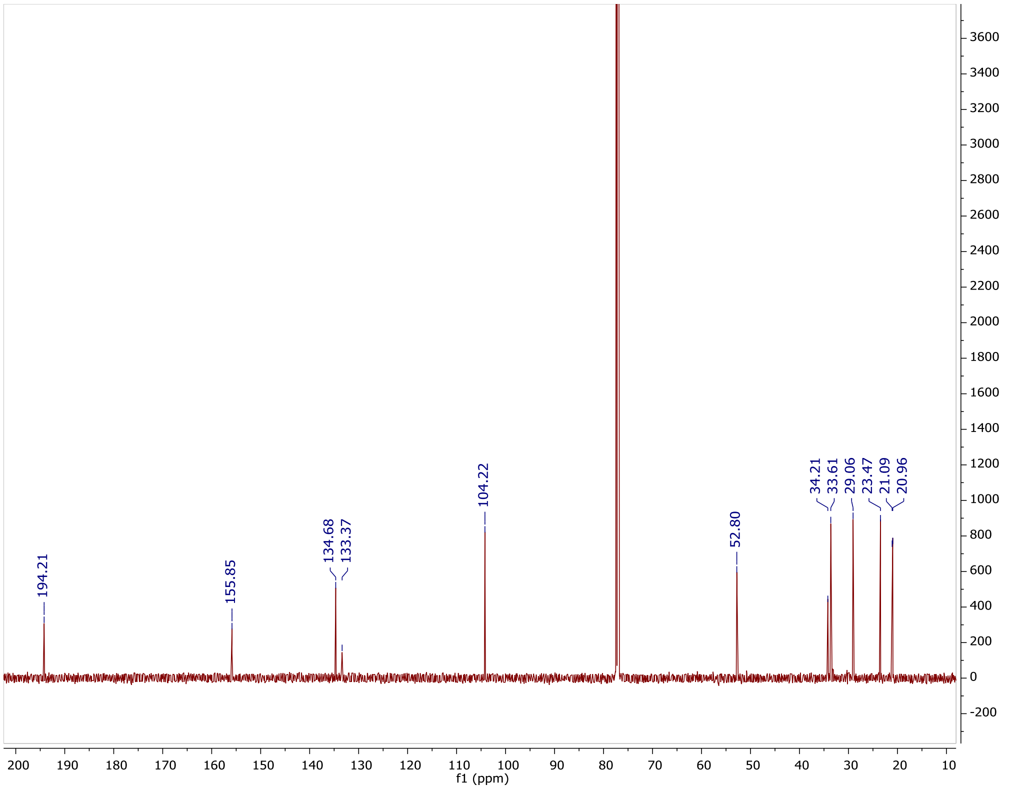

NOESY data for 4b (CDCl3):

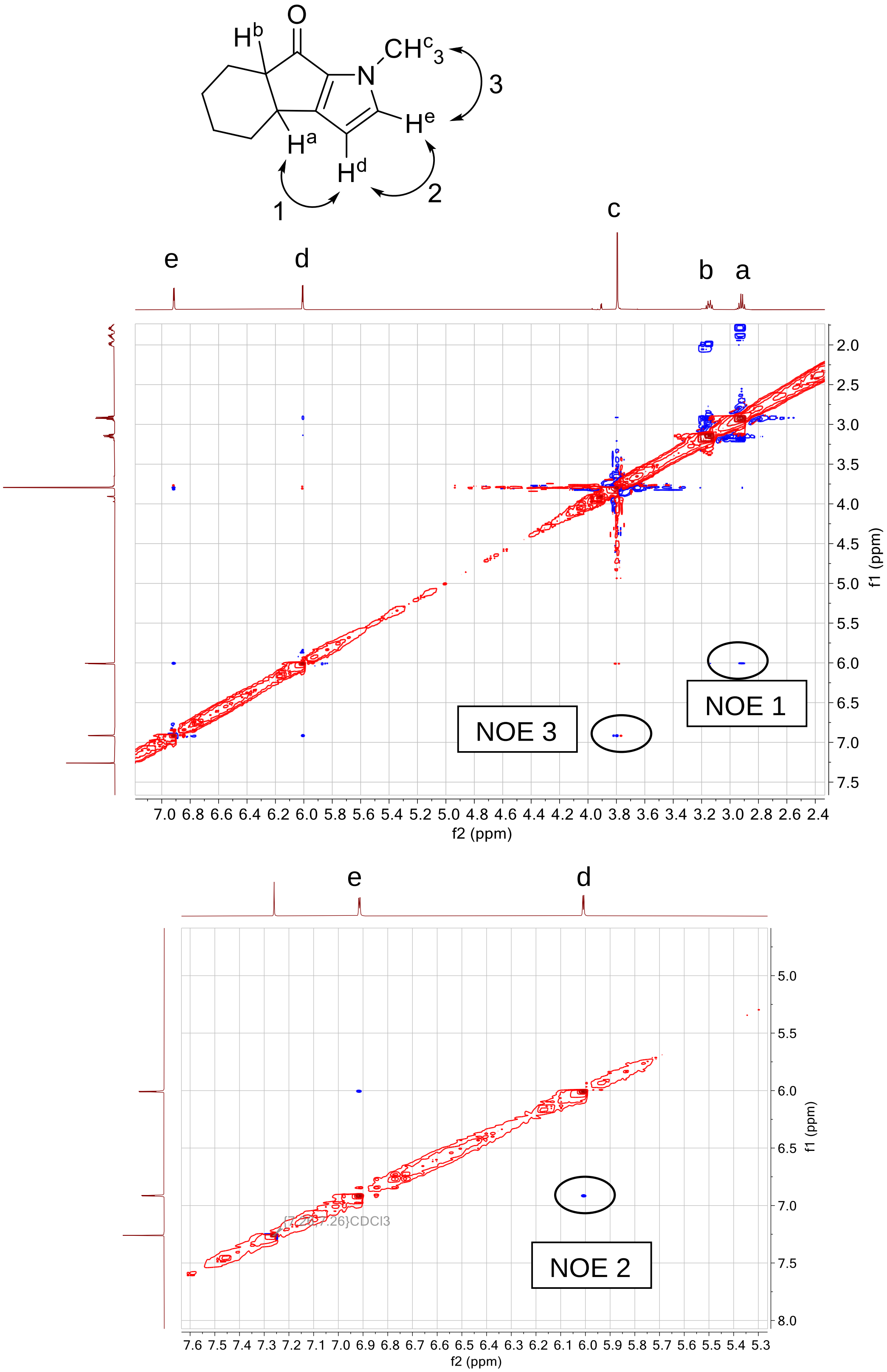

^{1}H and ^{13}C NMR data for 4c.

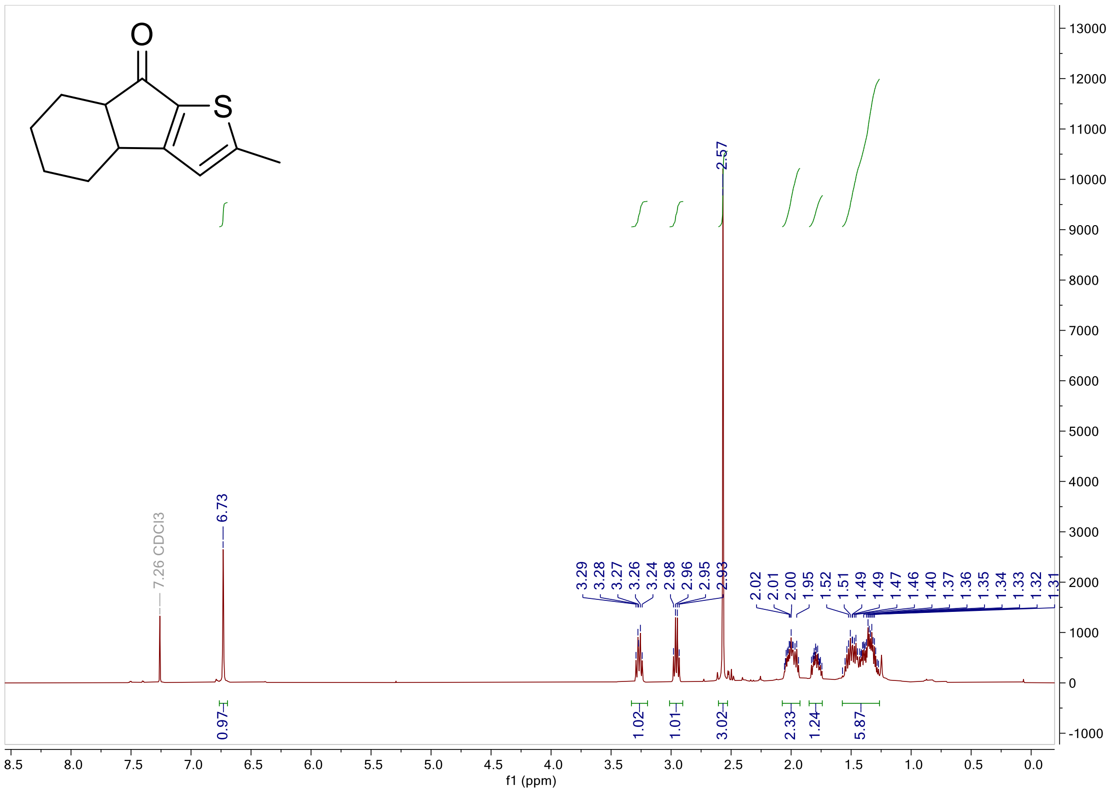

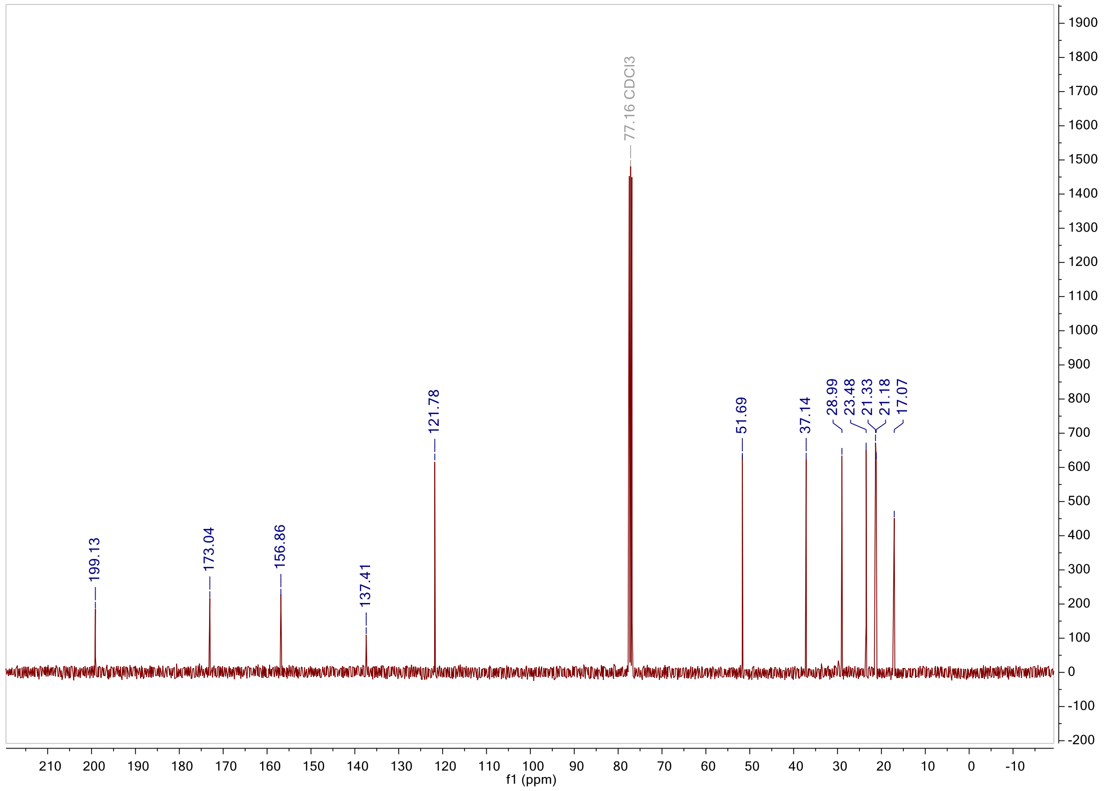

^{1}H, ^{19}F and ^{13}C NMR data for 5f.

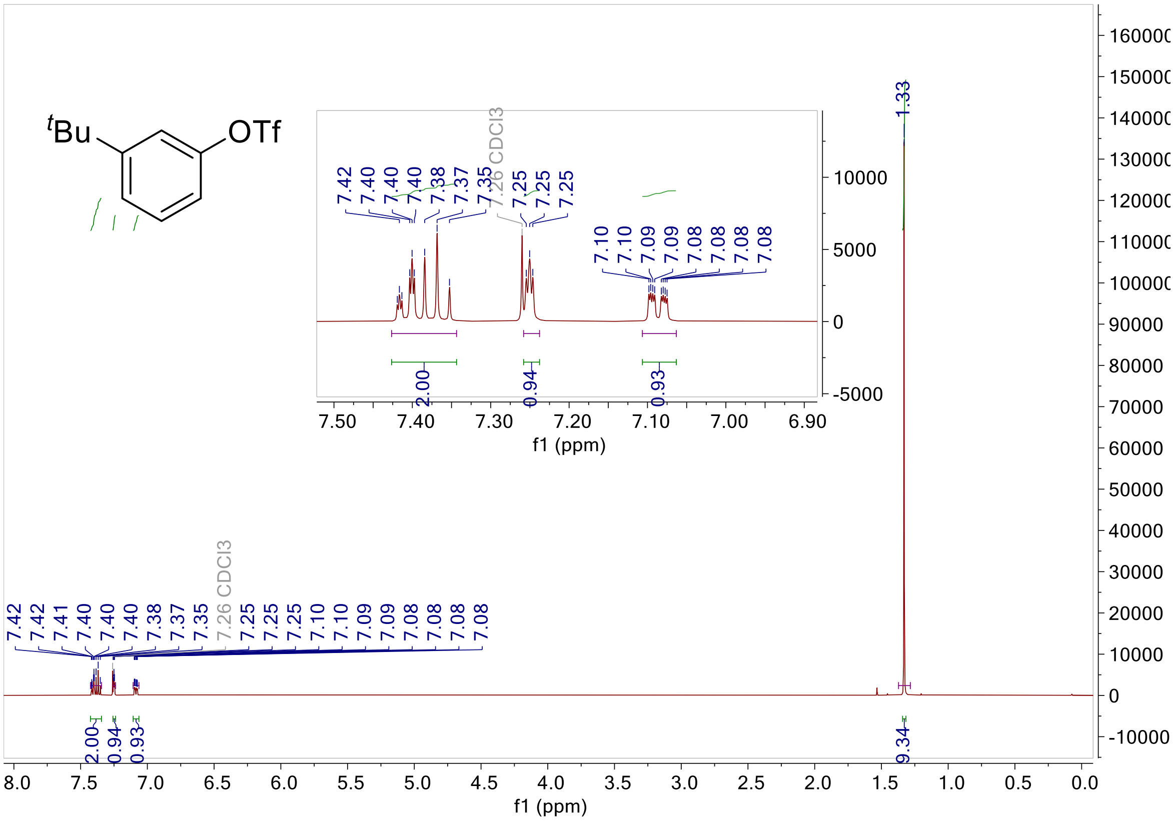

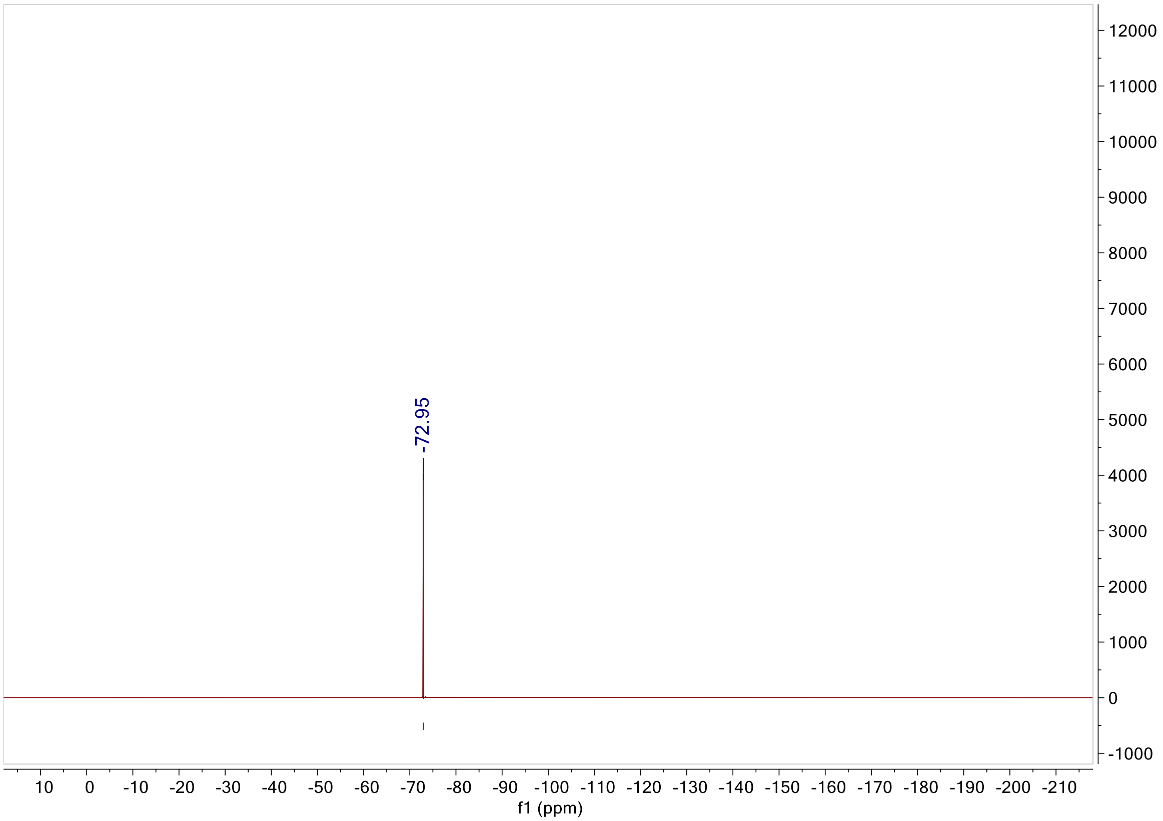

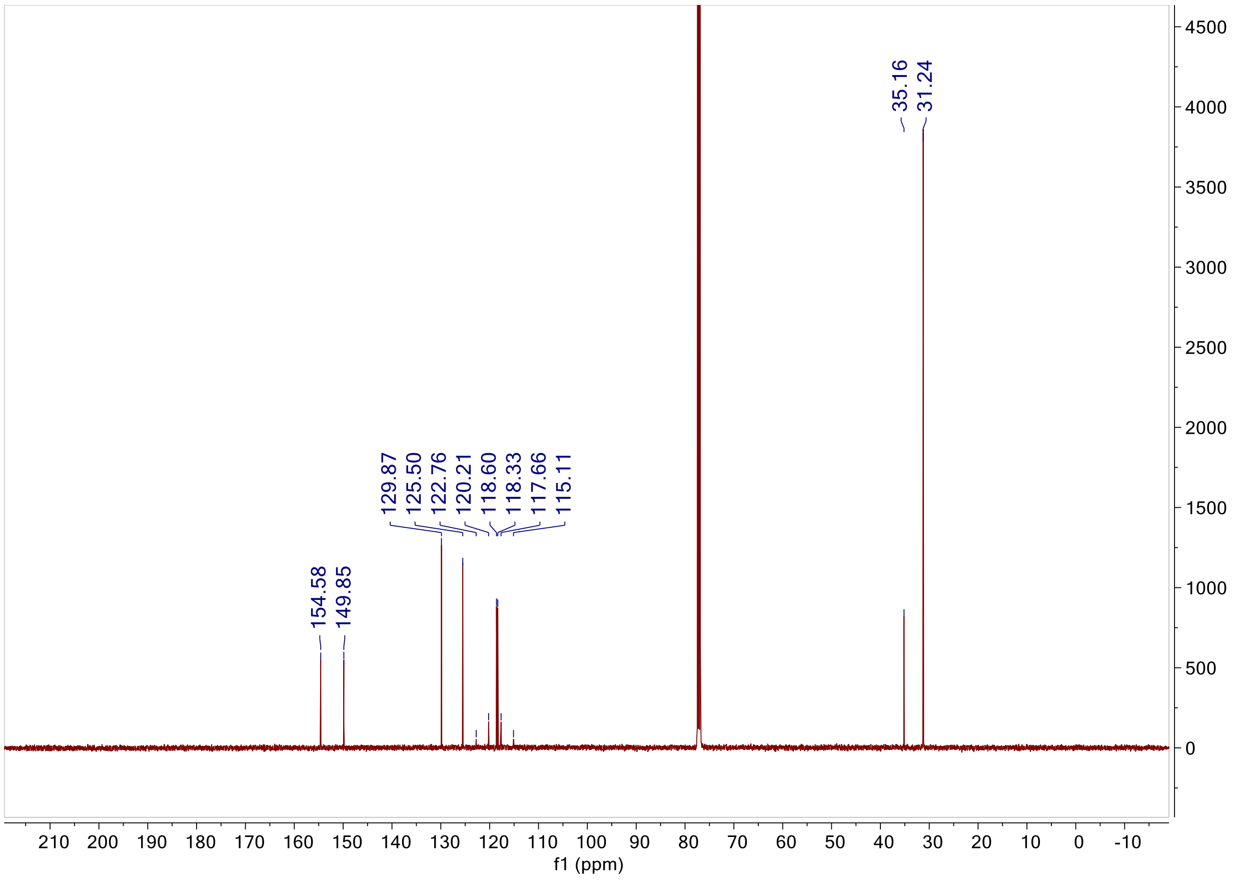
154.58
149.85
129.87
125.50
122.76
120.21
118.60
118.33
117.66
115.11
35.16
31.24
4500
4000
3500
3000
2500
2000
1500
1000
500
0
210 200 190 180 170 160 150 140 130 120 110 100 90 80 70 60 50 40 30 20 10 0 -10
f1 (ppm)

^{1}H, ^{19}F and ^{13}C NMR data for 5g.

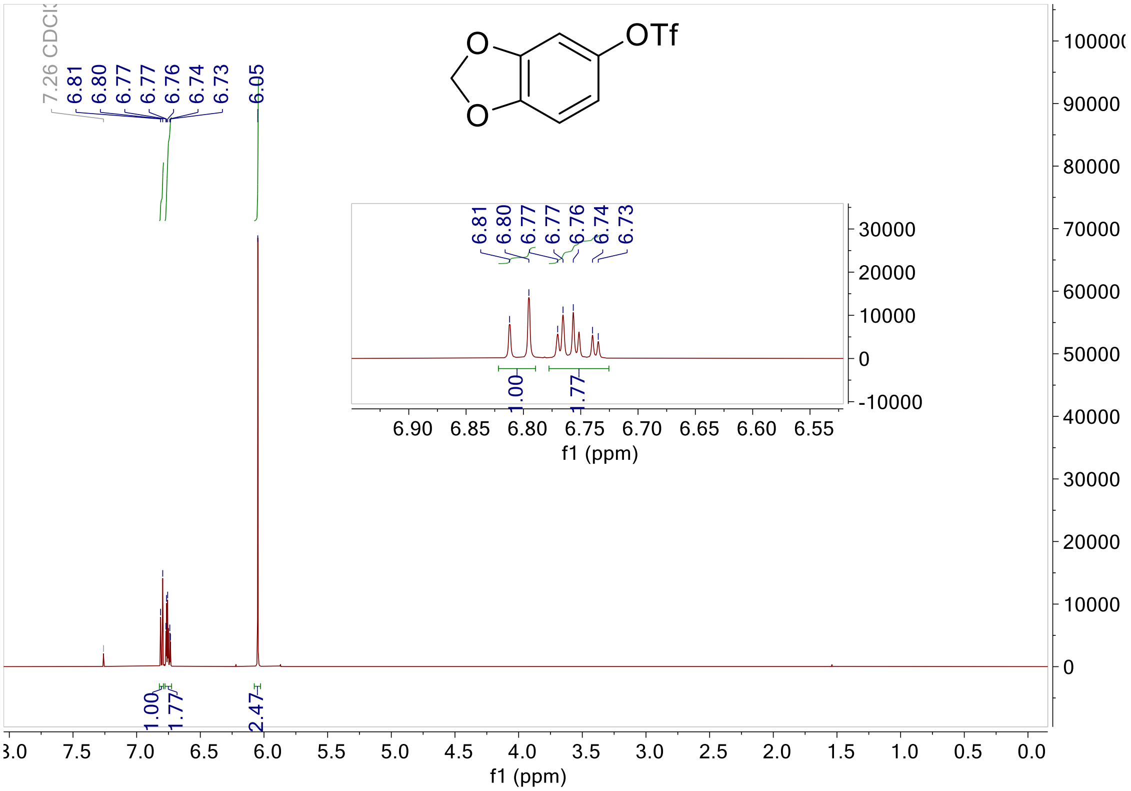

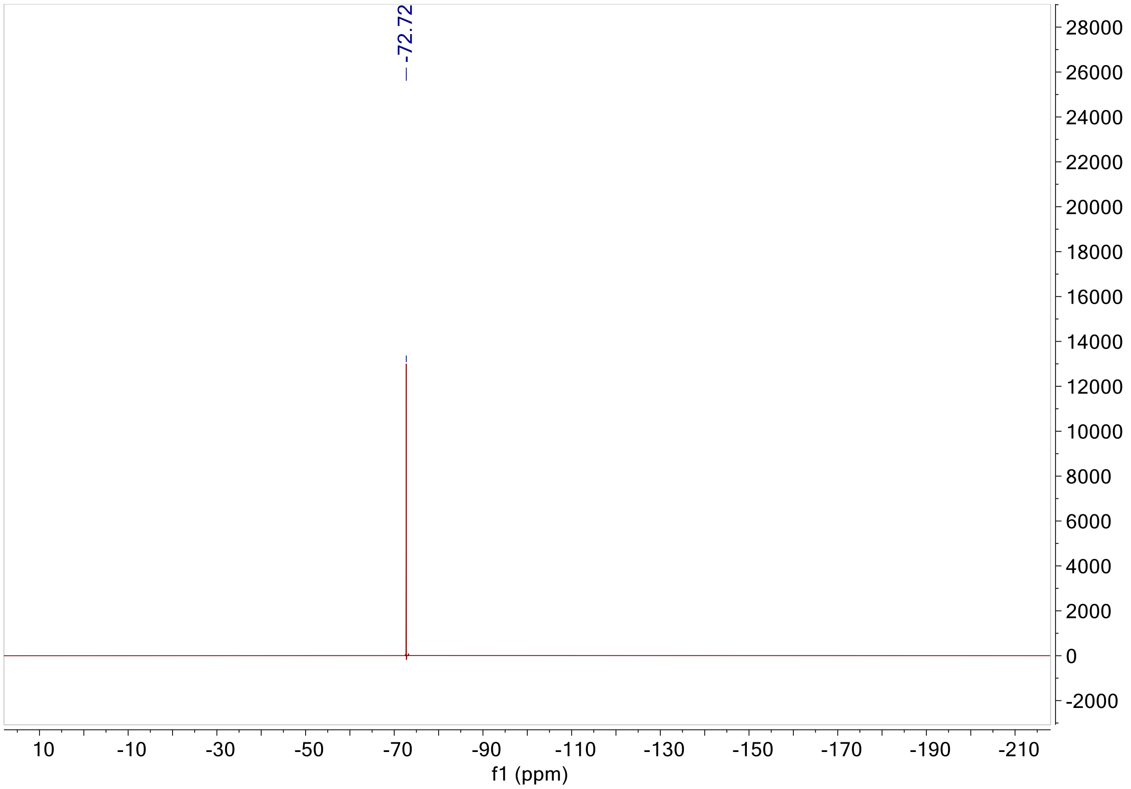

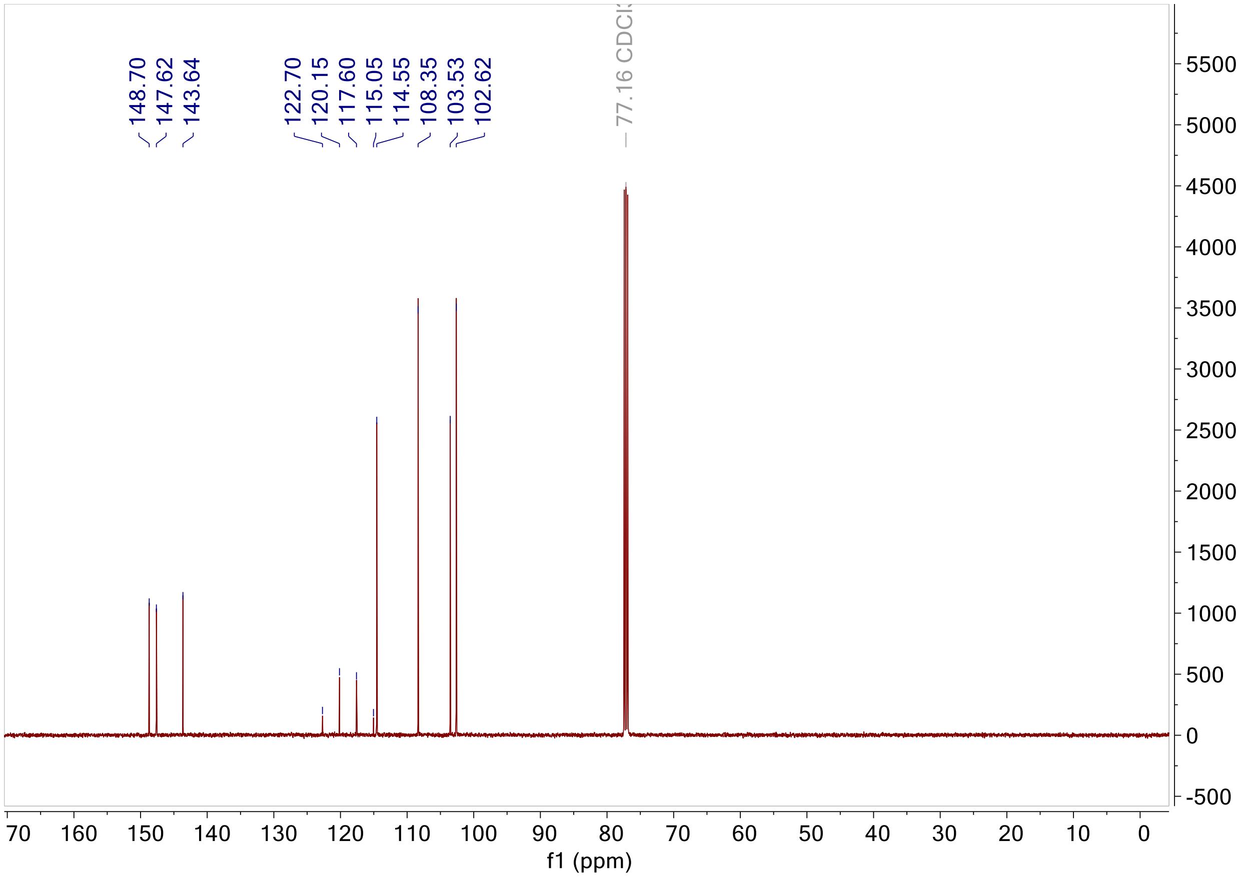
148.70
147.62
143.64
122.70
120.15
117.60
115.05
114.55
108.35
103.53
102.62
77.16 CDCl3
f1 (ppm)

^{1}H, ^{19}F and ^{13}C NMR data for 6a.

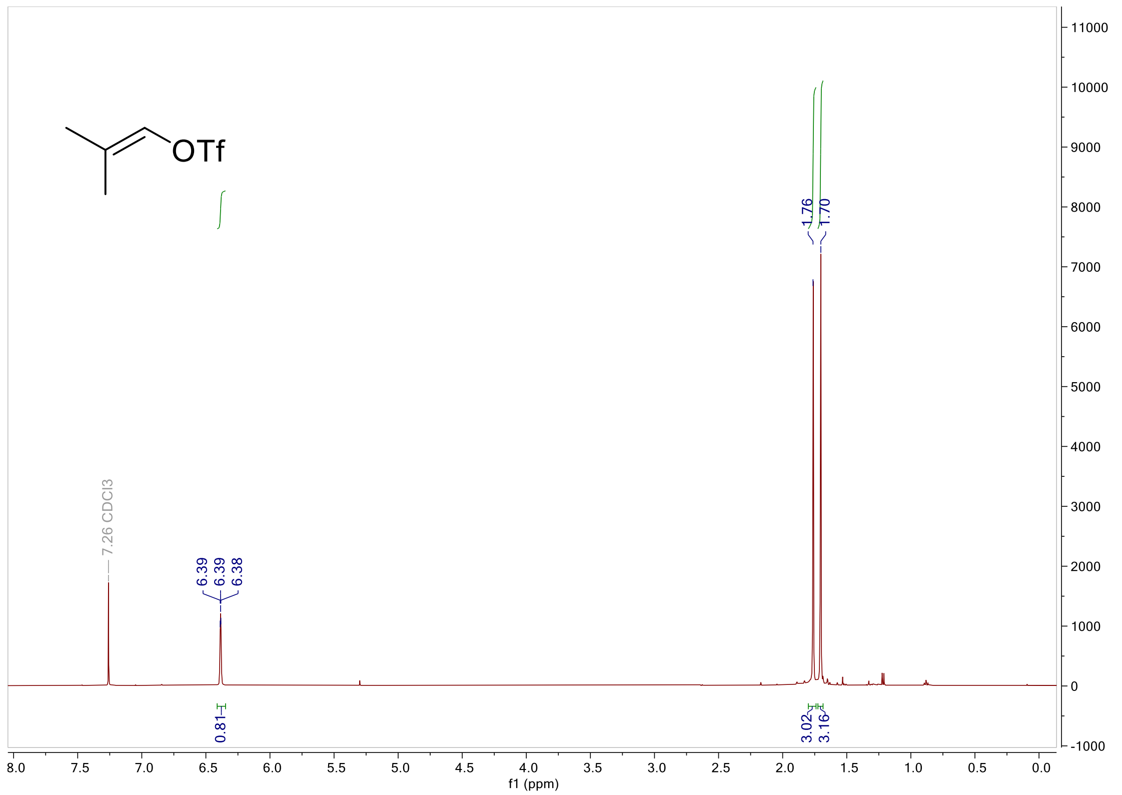

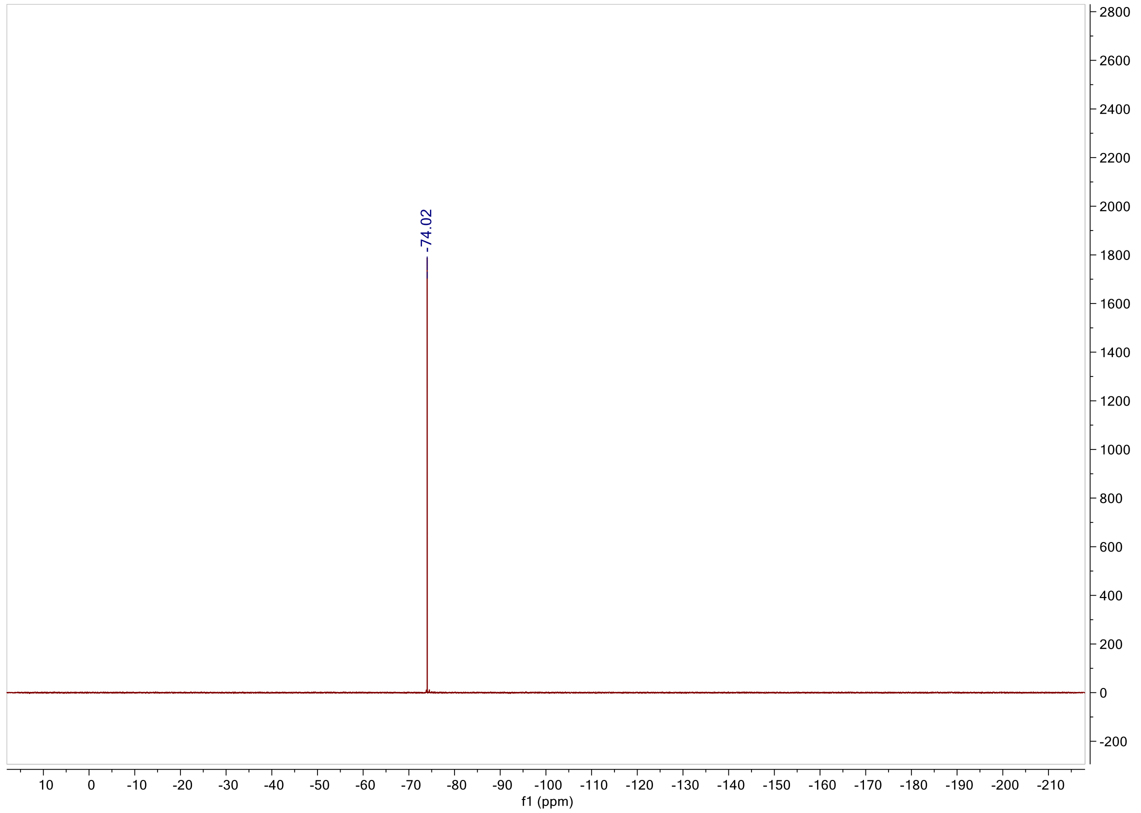

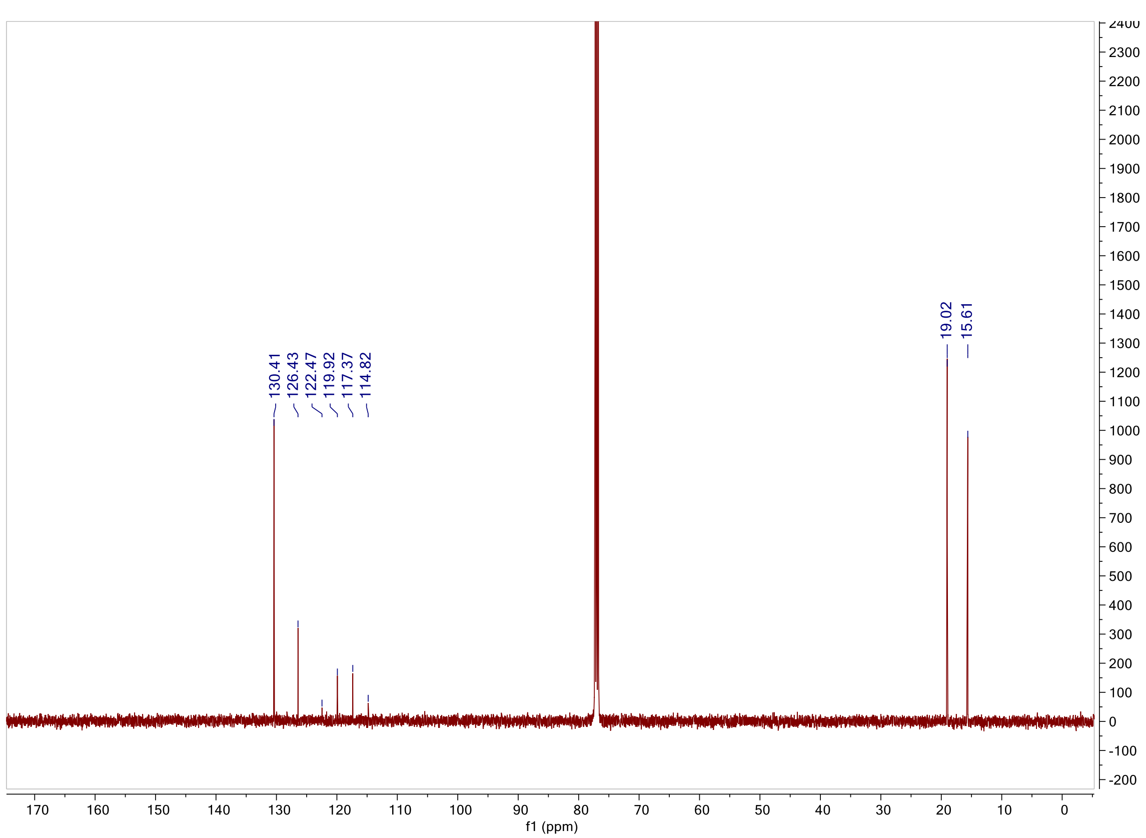
130.41
126.43
122.47
119.92
117.37
114.82
19.02
15.61
f1 (ppm)

^{1}H, ^{19}F and ^{13}C NMR data for 6b.

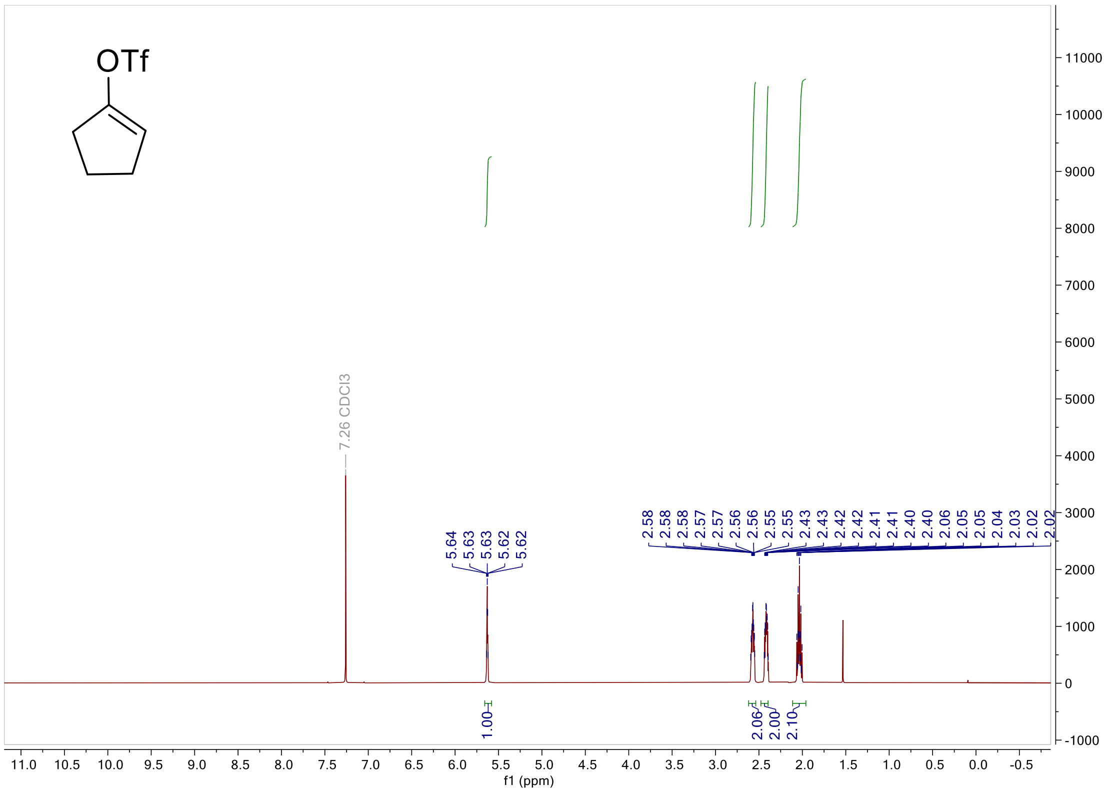

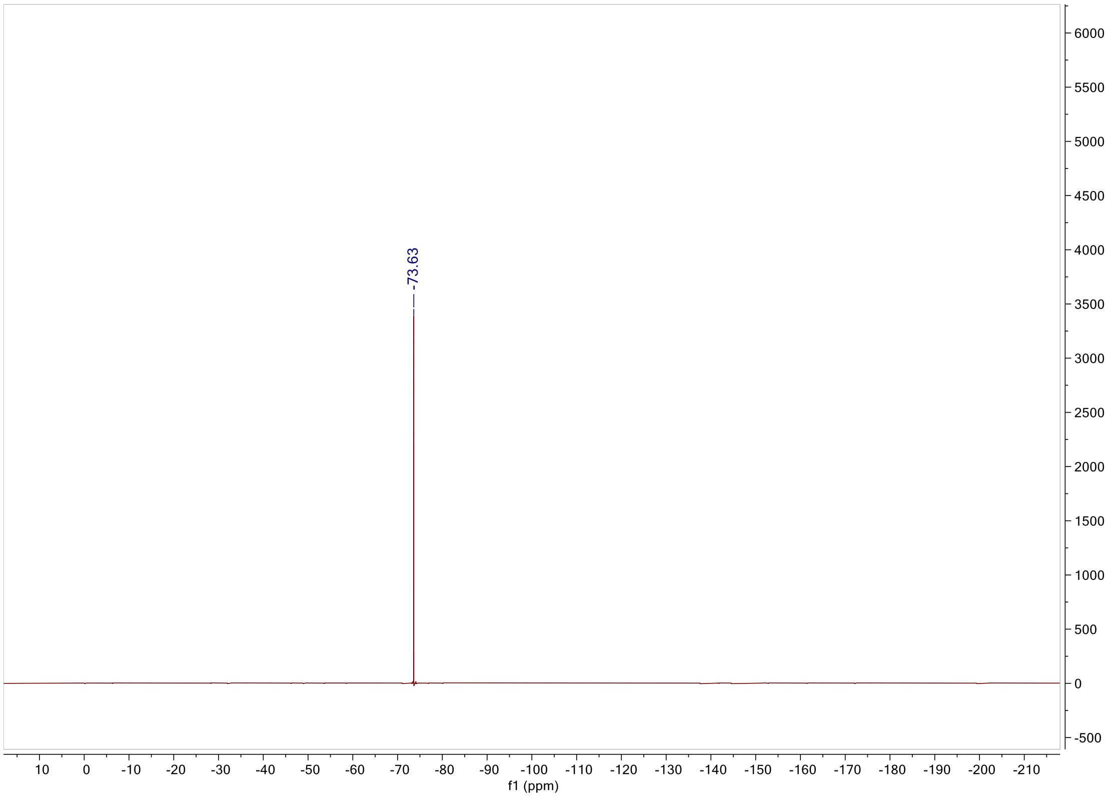

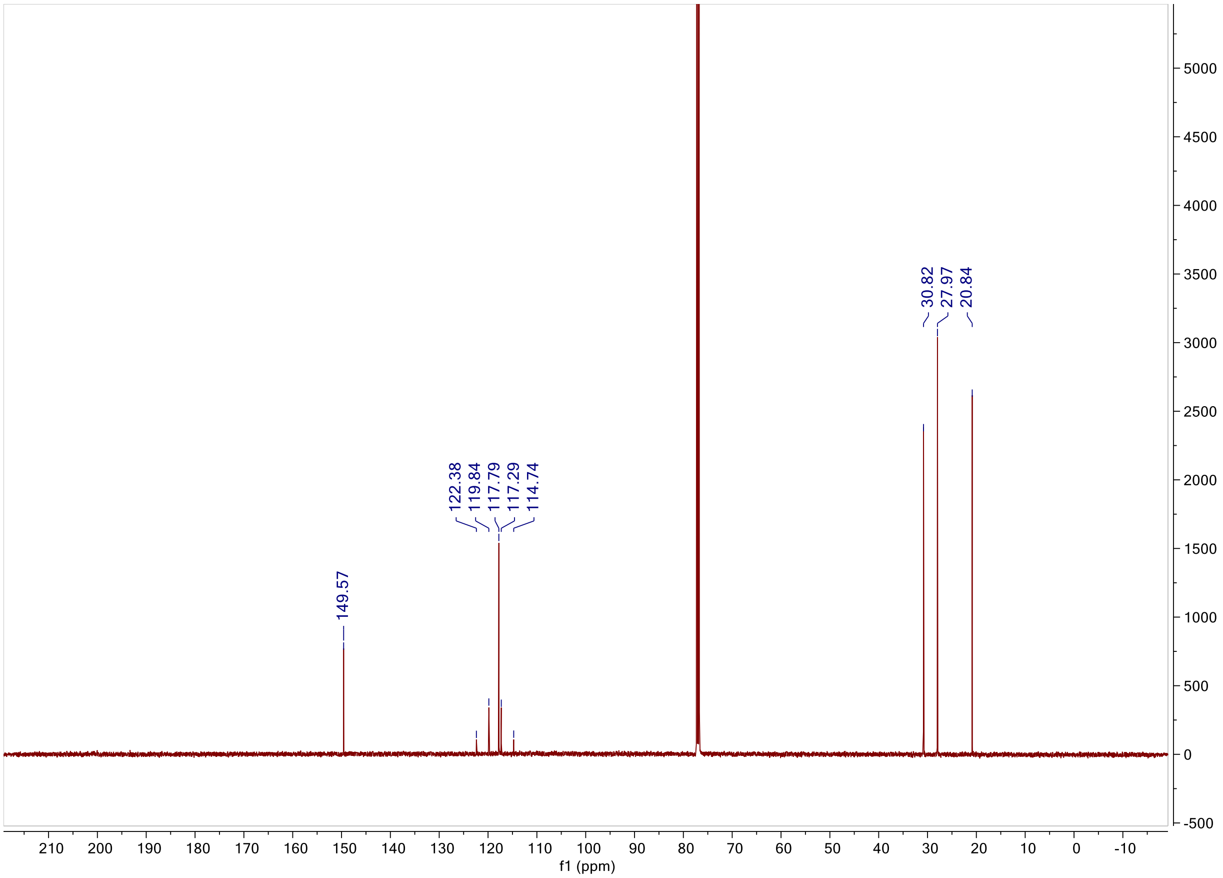
149.57
122.38
119.84
117.79
117.29
114.74
30.82
27.97
20.84
5000
4500
4000
3500
3000
2500
2000
1500
1000
500
0
-500
210 200 190 180 170 160 150 140 130 120 110 100 90 80 70 60 50 40 30 20 10 0 -10
f1 (ppm)

^{1}H, ^{19}F and ^{13}C NMR data for 6c.

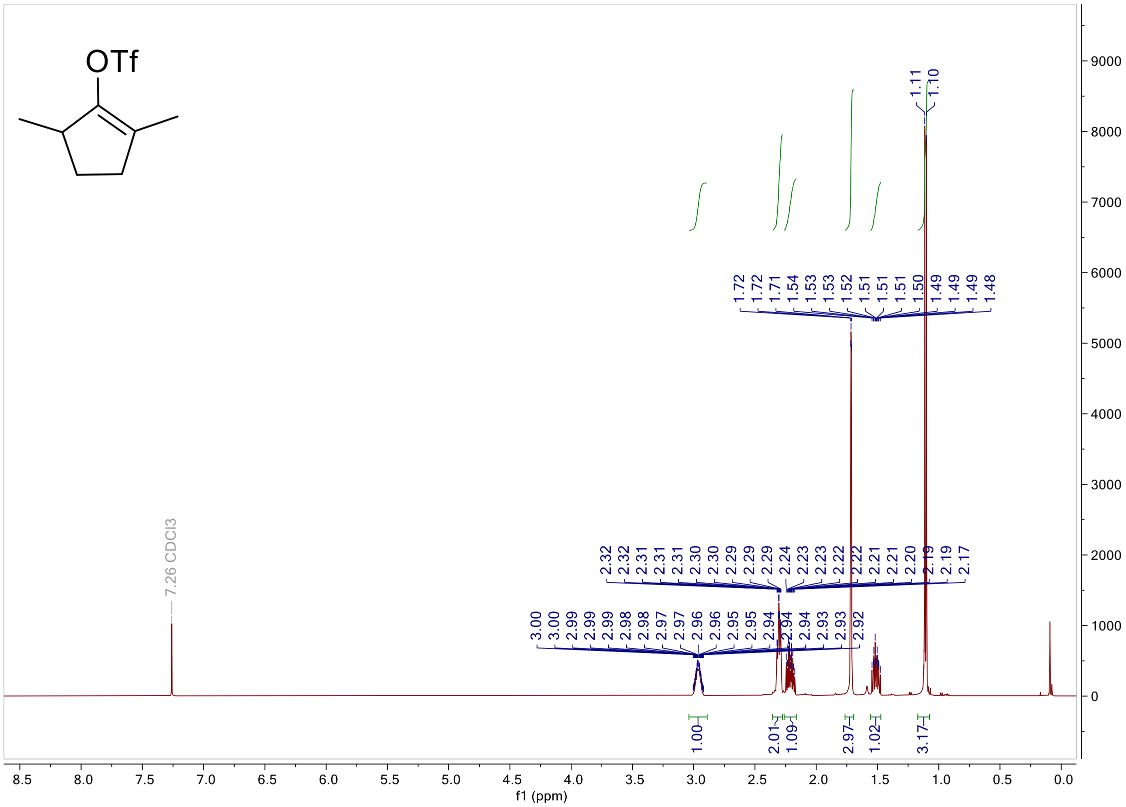

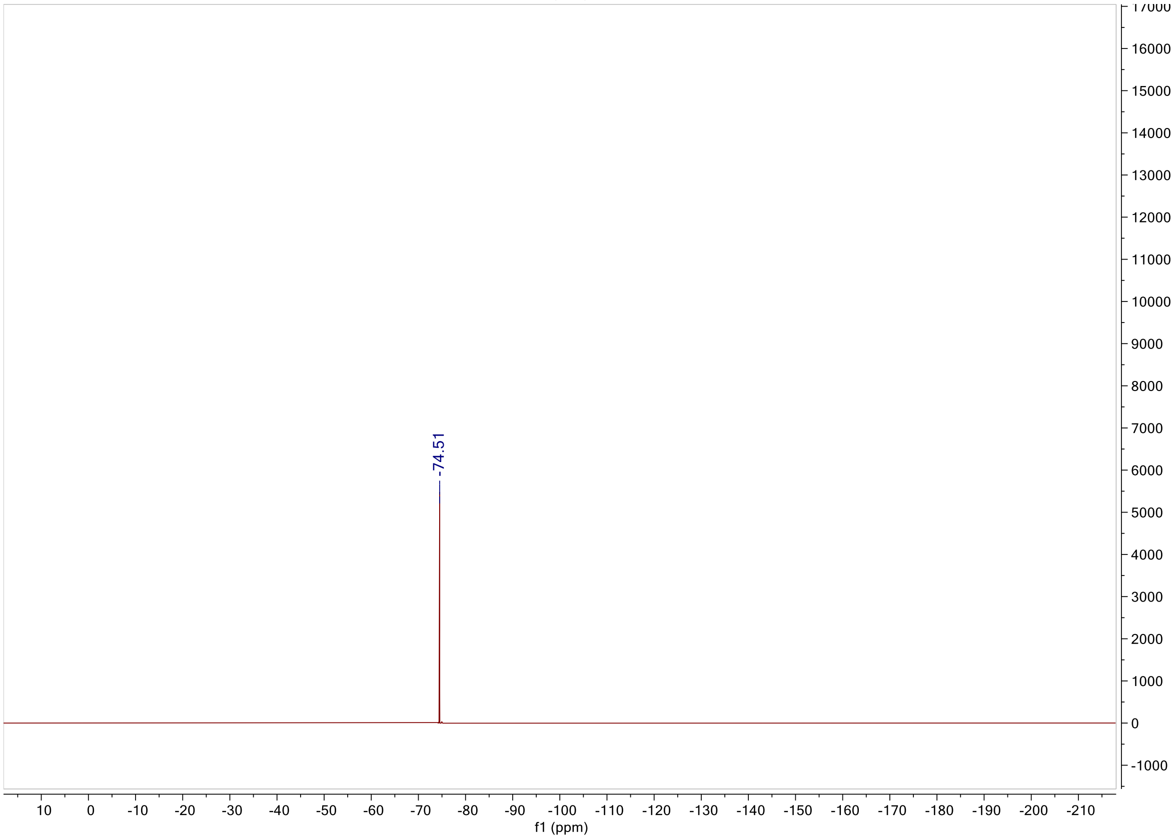

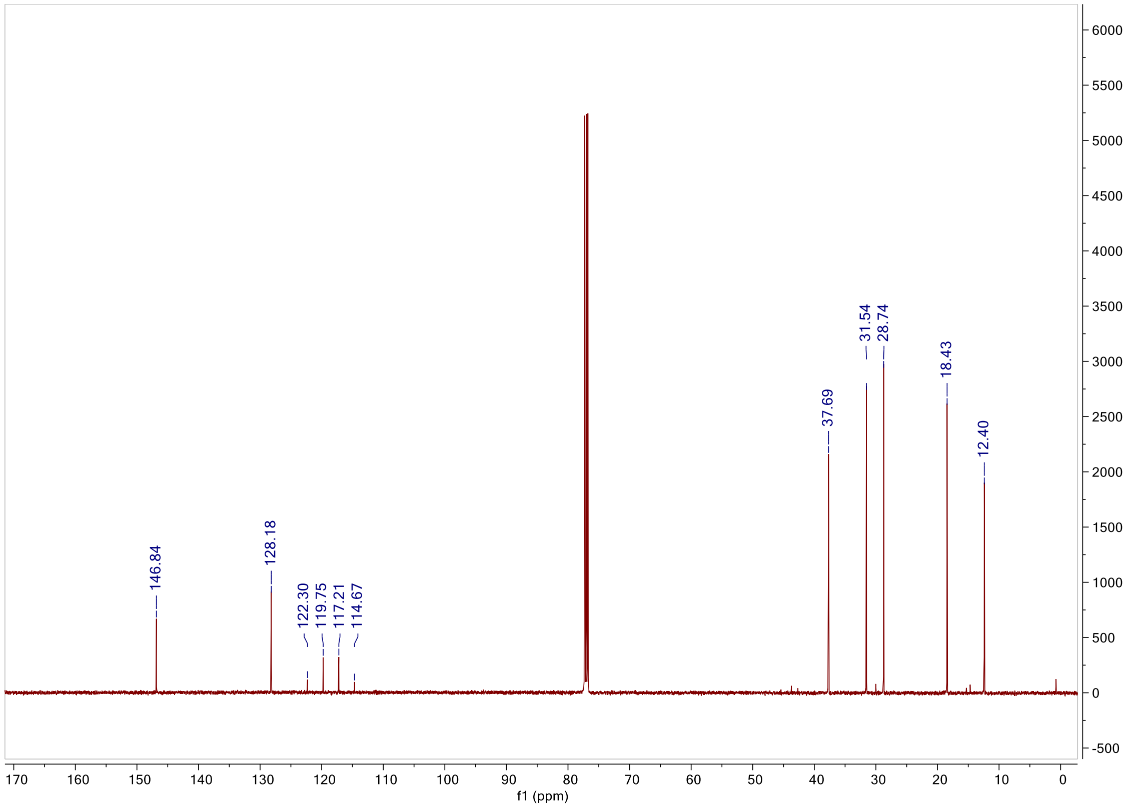
146.84
128.18
122.30
119.75
117.21
114.67
37.69
31.54
28.74
18.43
12.40
6000
5500
5000
4500
4000
3500
3000
2500
2000
1500
1000
500
0
-500
170 160 150 140 130 120 110 100 90 80 70 60 50 40 30 20 10 0
f1 (ppm)

^{1}H, ^{19}F and ^{13}C NMR data for 6d.

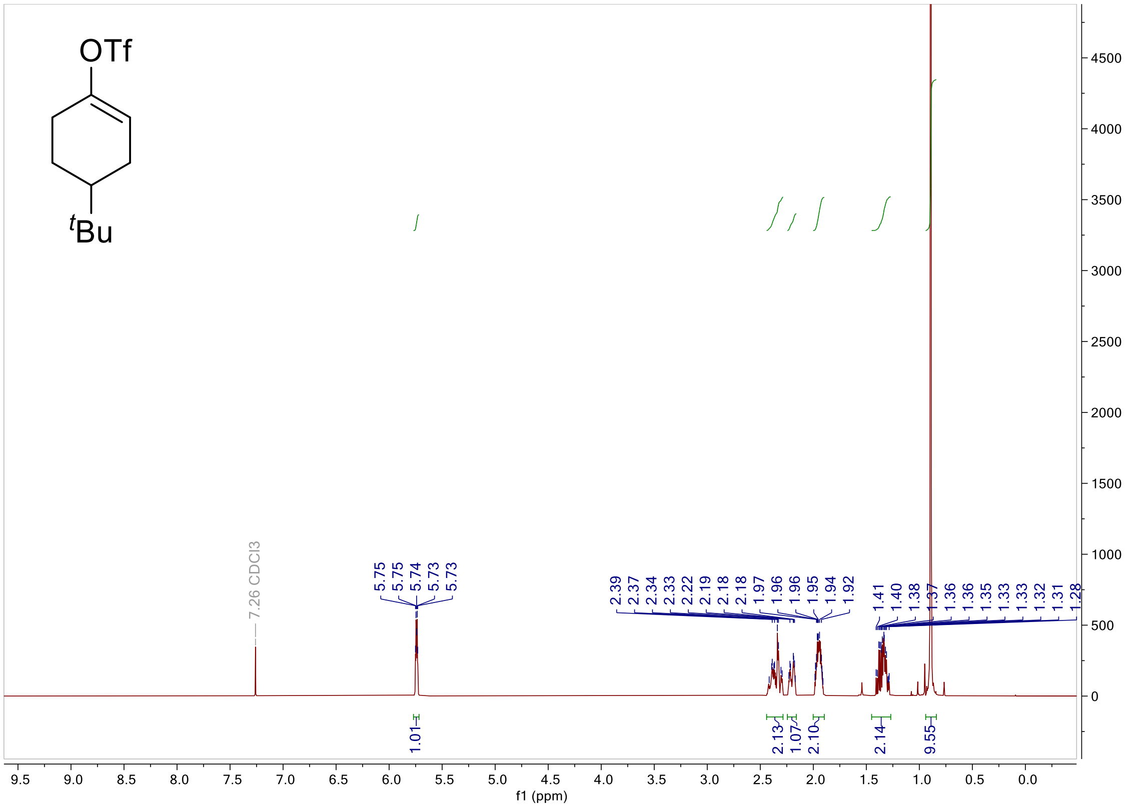

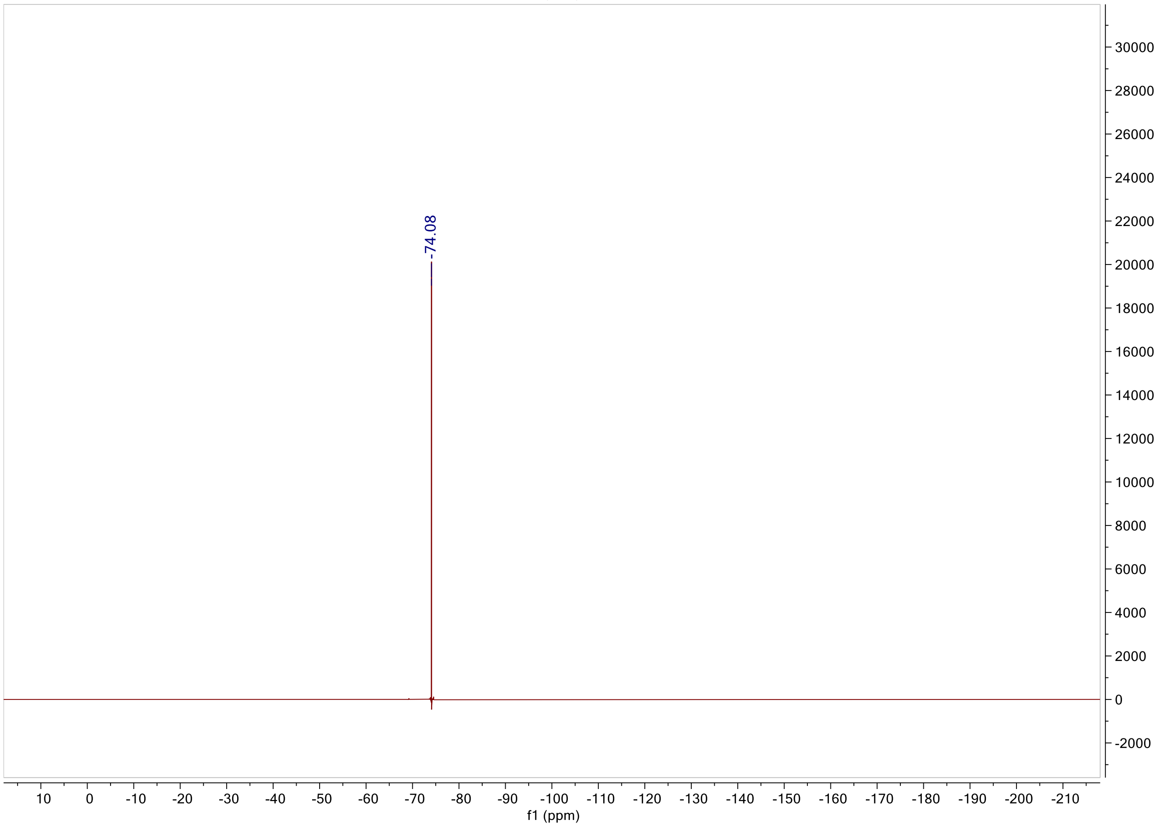

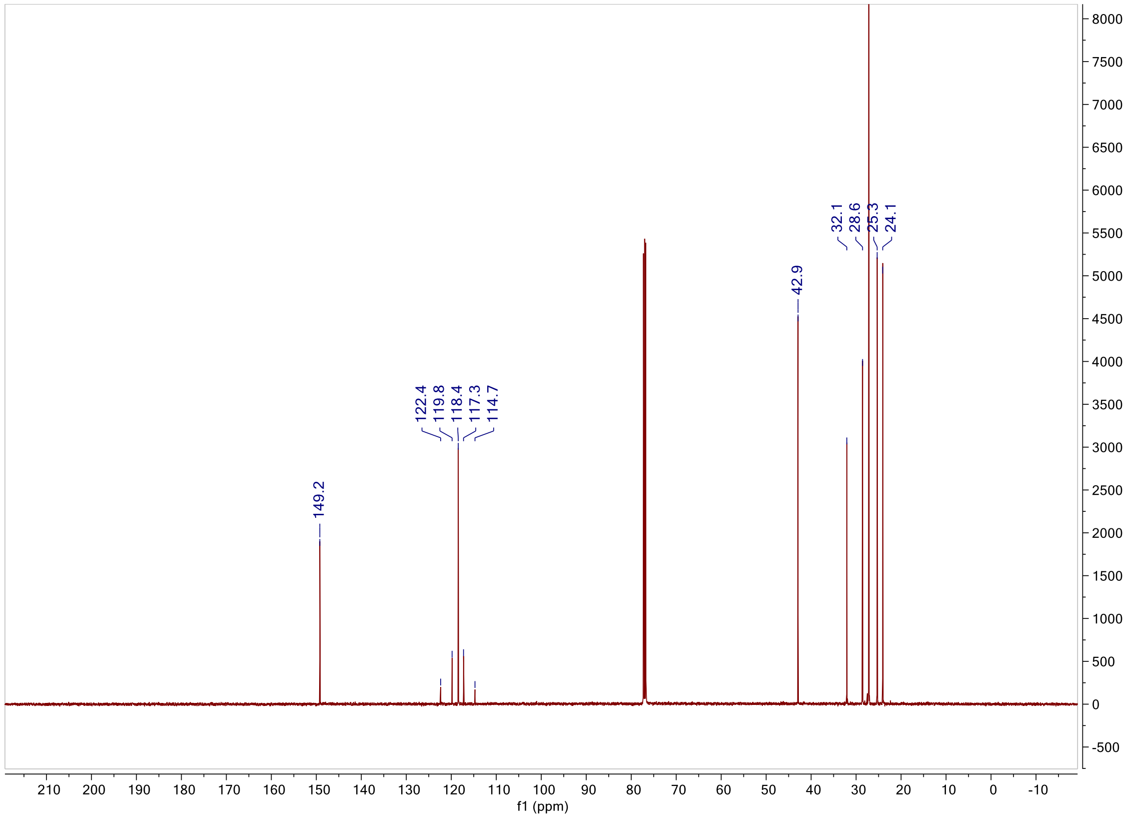

149.2
122.4
119.8
118.4
117.3
114.7
42.9
32.1
28.6
25.3
24.1
8000
7500
7000
6500
6000
5500
5000
4500
4000
3500
3000
2500
2000
1500
1000
500
0
-500
210 200 190 180 170 160 150 140 130 120 110 100 90 80 70 60 50 40 30 20 10 0 -10
f1 (ppm)

^{1}H, ^{19}F and ^{13}C NMR data for 6e.

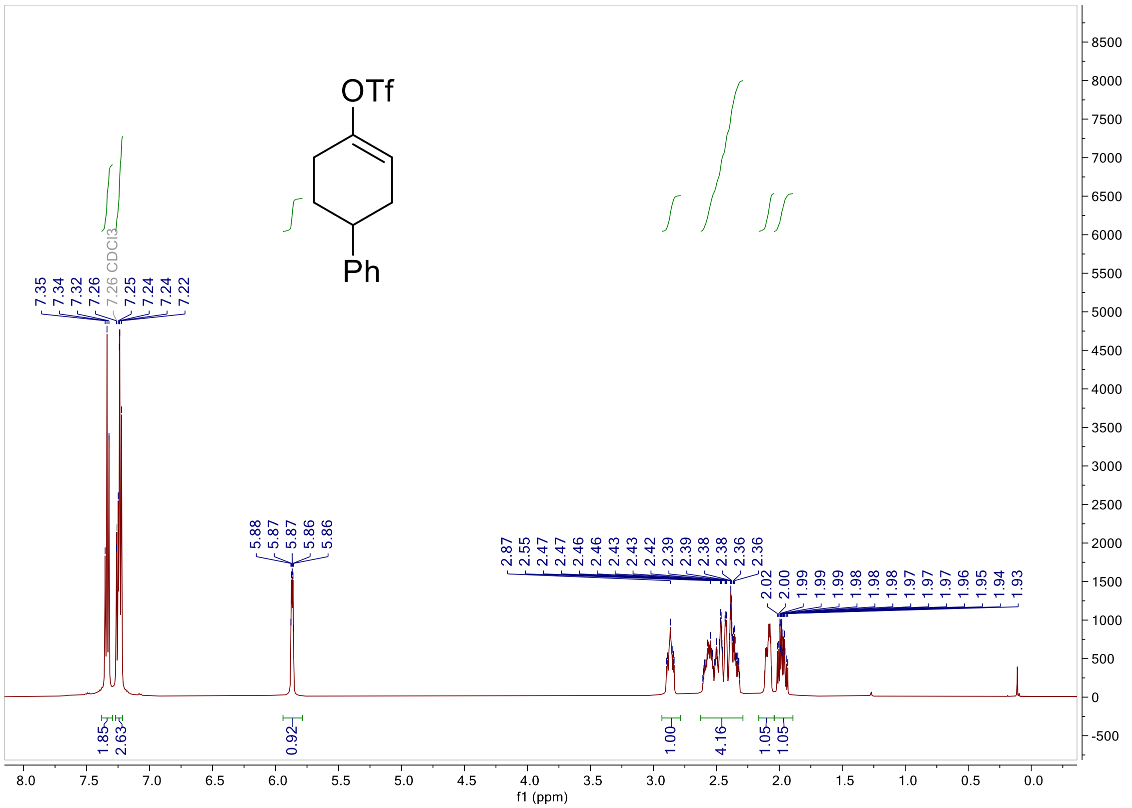

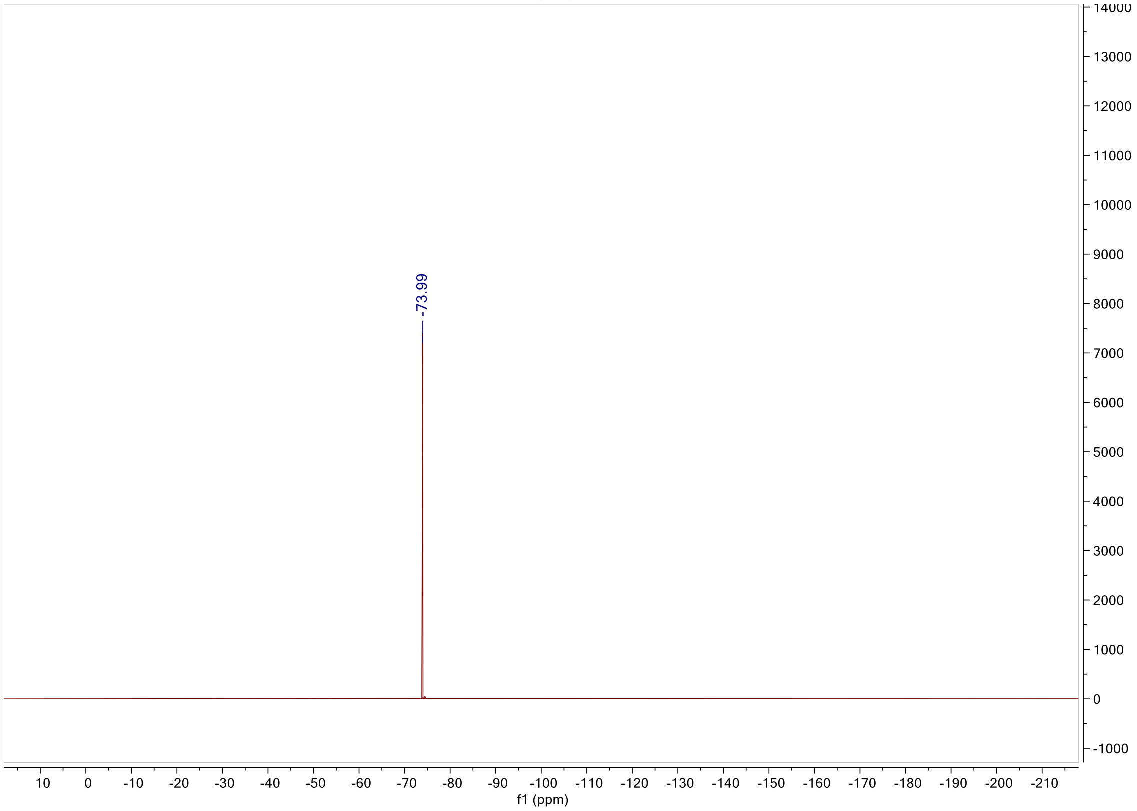

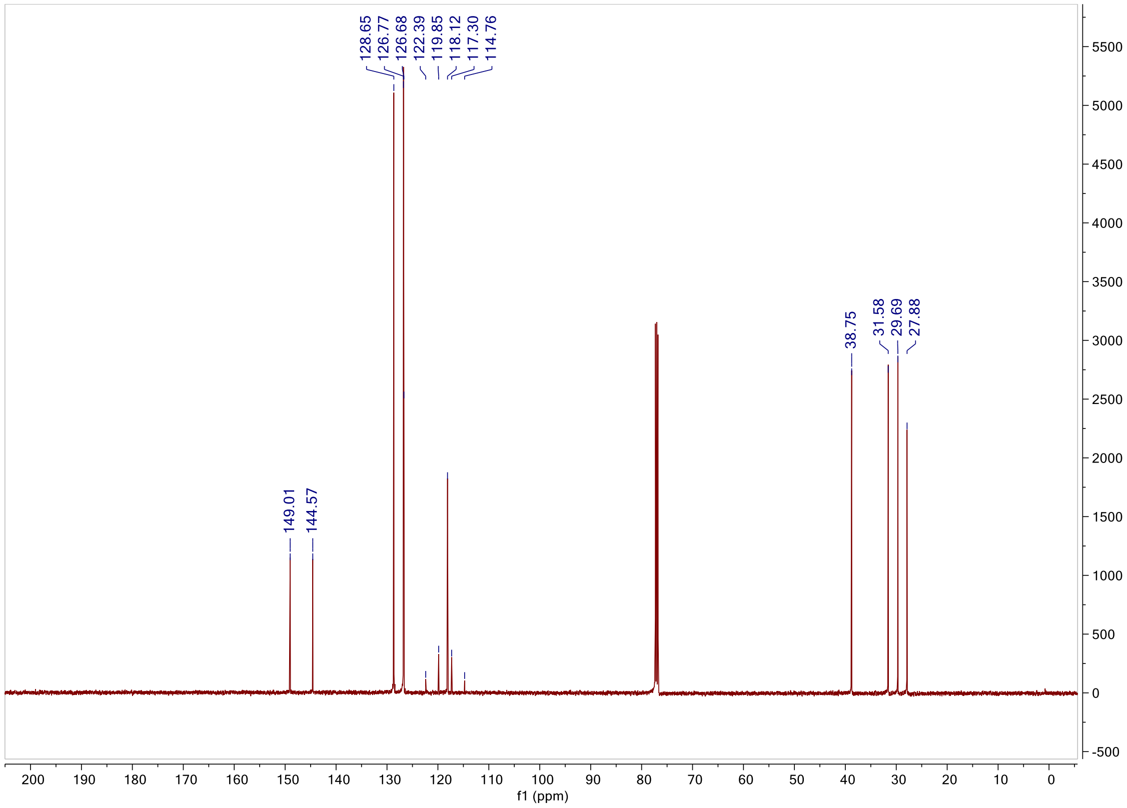
128.65
126.77
126.68
122.39
119.85
118.12
117.30
114.76
149.01
144.57
38.75
31.58
29.69
27.88
f1 (ppm)

^{1}H, ^{19}F and ^{13}C NMR data for 6f.

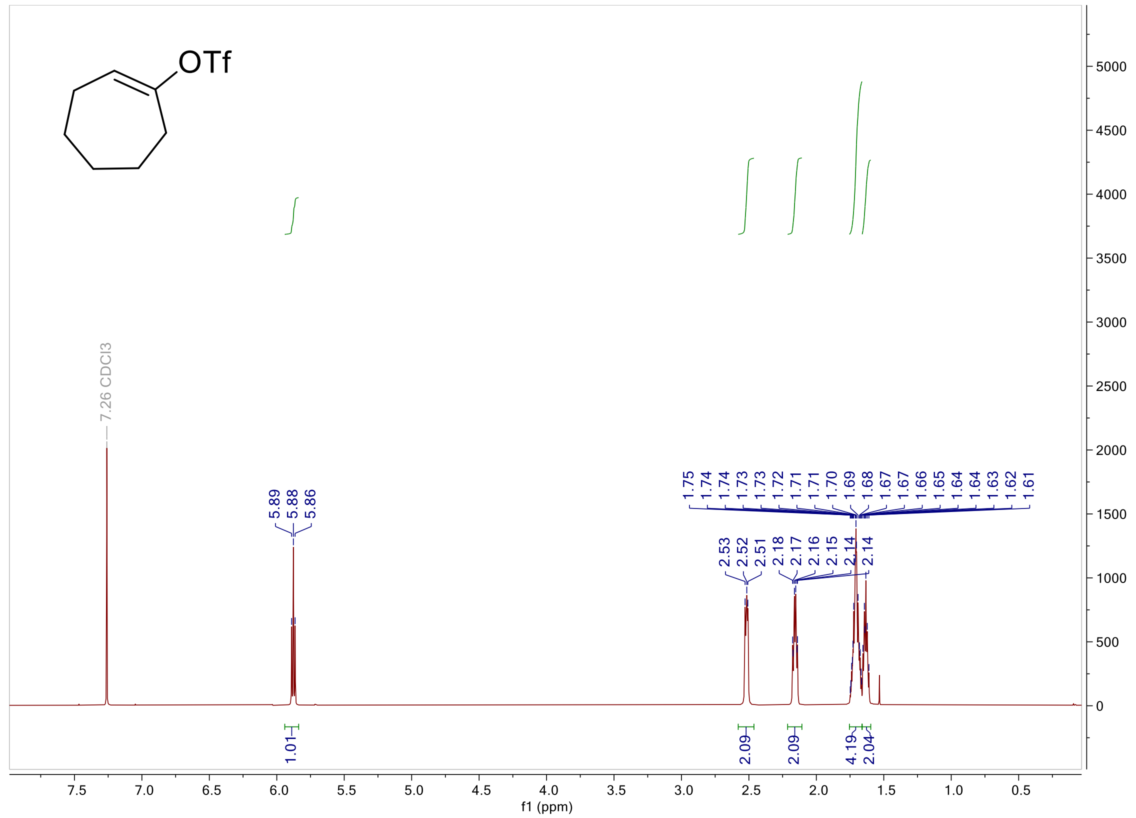

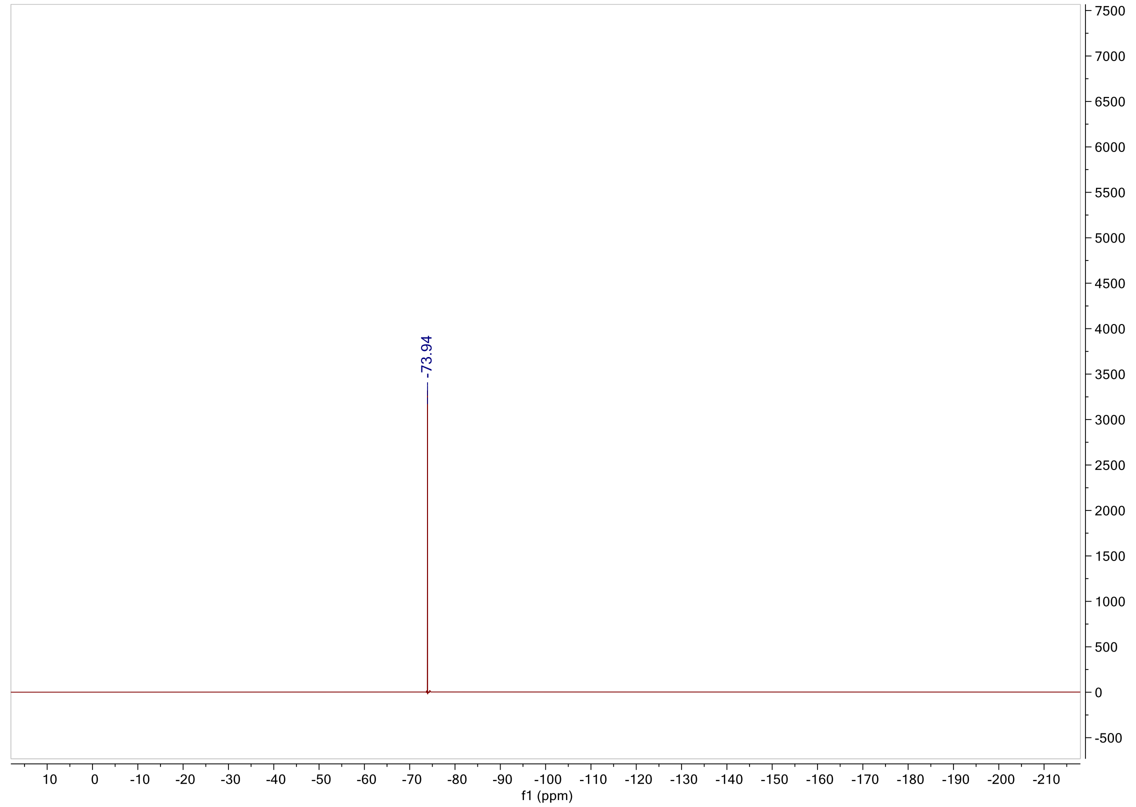

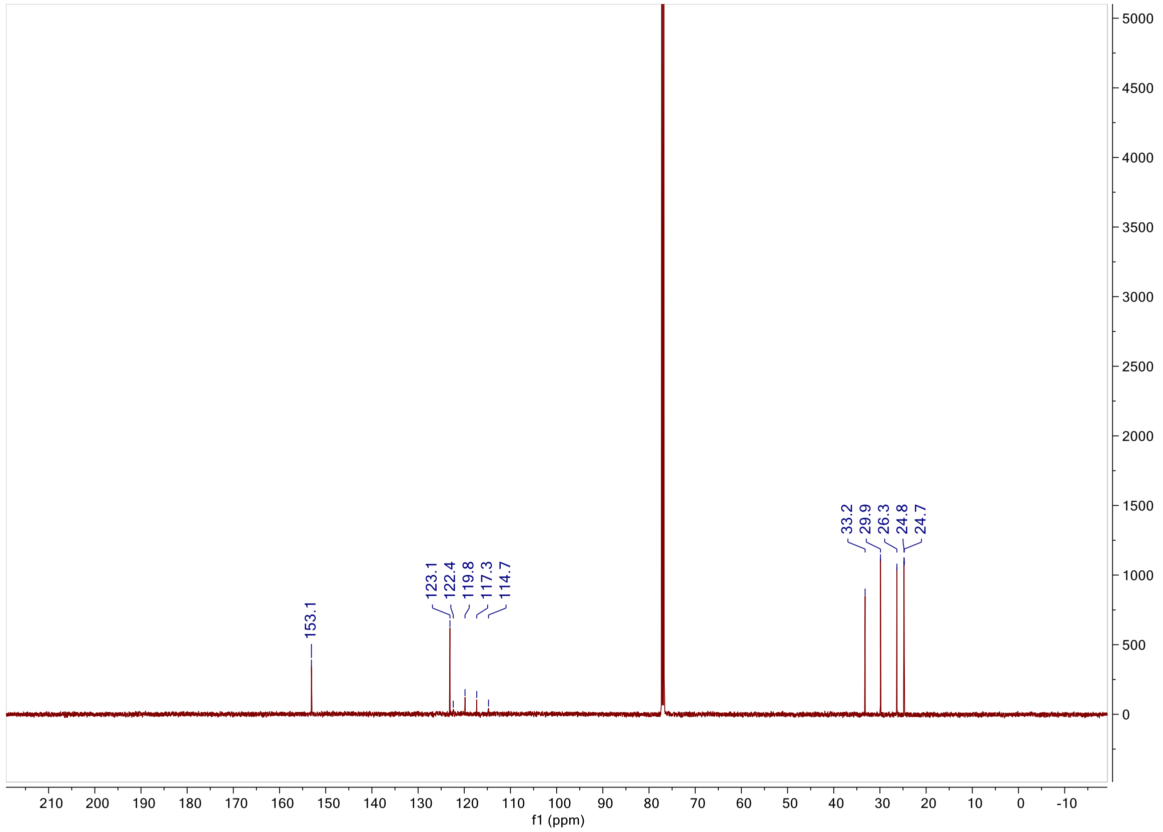
153.1
123.1
122.4
119.8
117.3
114.7
33.2
29.9
26.3
24.8
24.7
f1 (ppm)

^{1}H, ^{19}F and ^{13}C NMR data for 6g.

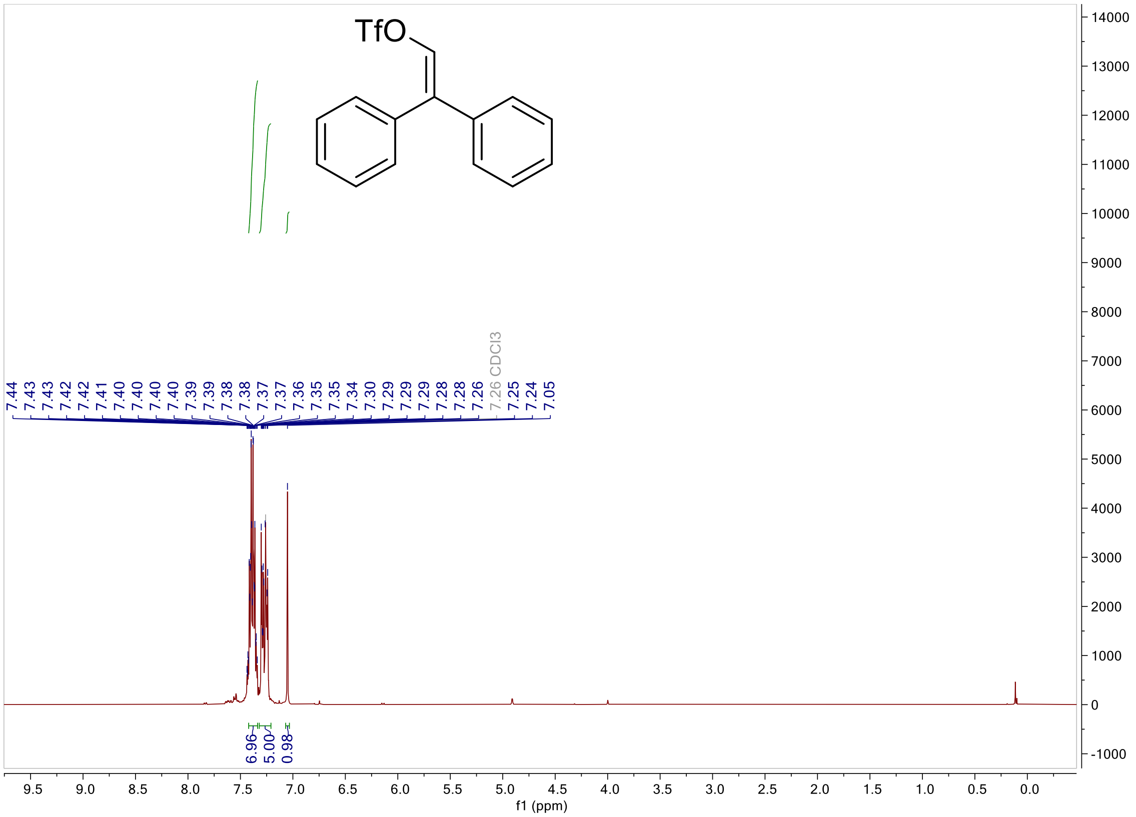

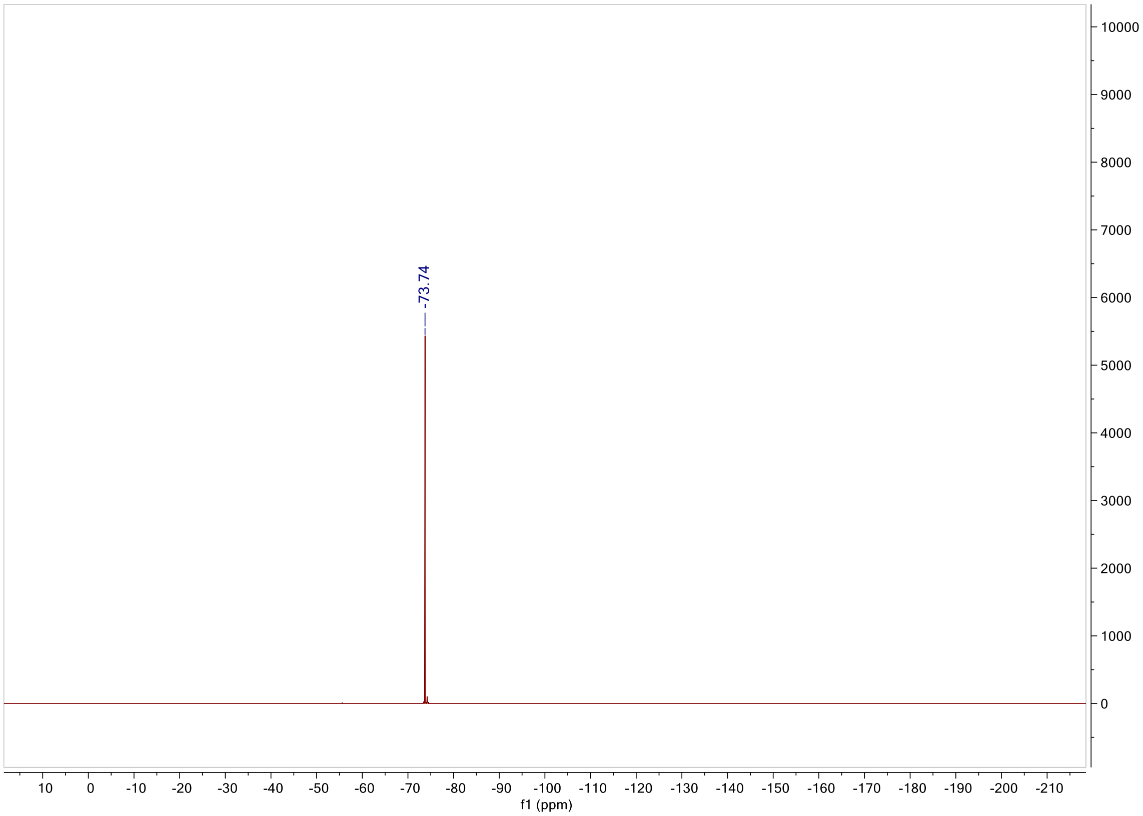

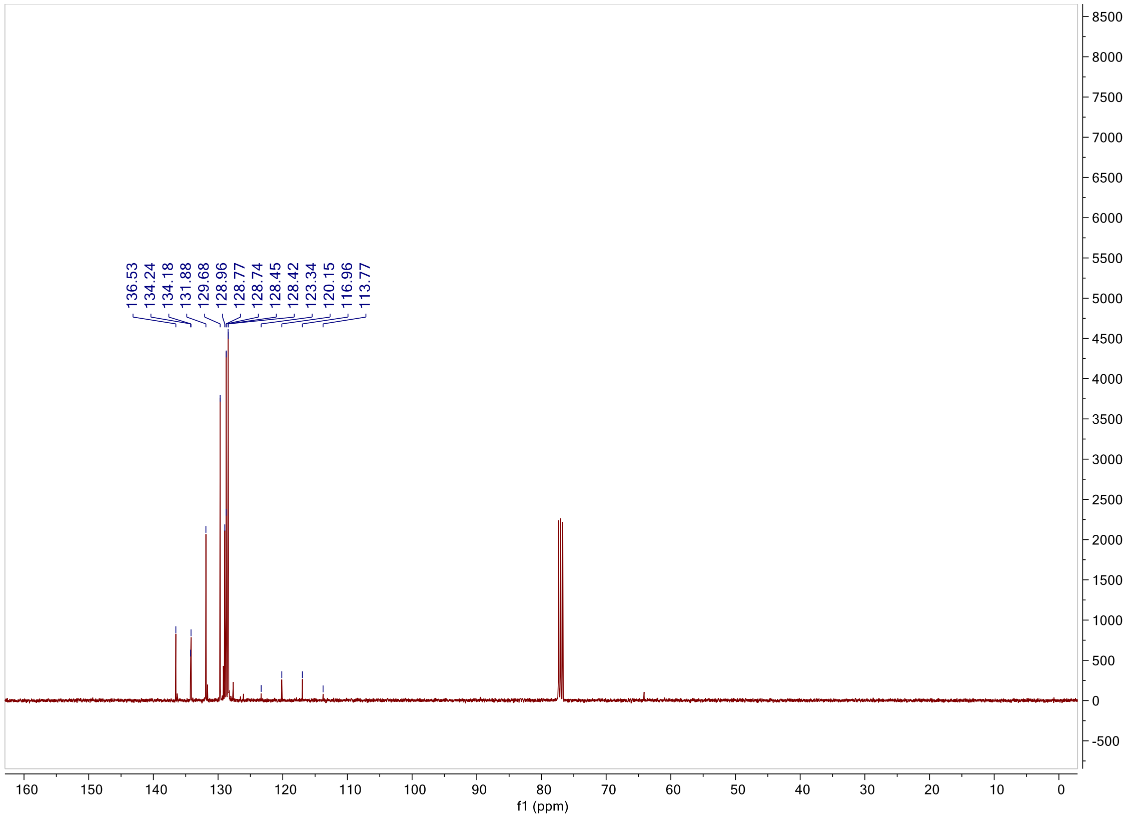
136.53
134.24
134.18
131.88
129.68
128.96
128.77
128.74
128.45
128.42
123.34
120.15
116.96
113.77
f1 (ppm)

^{1}H, ^{19}F and ^{13}C NMR data for 6h.

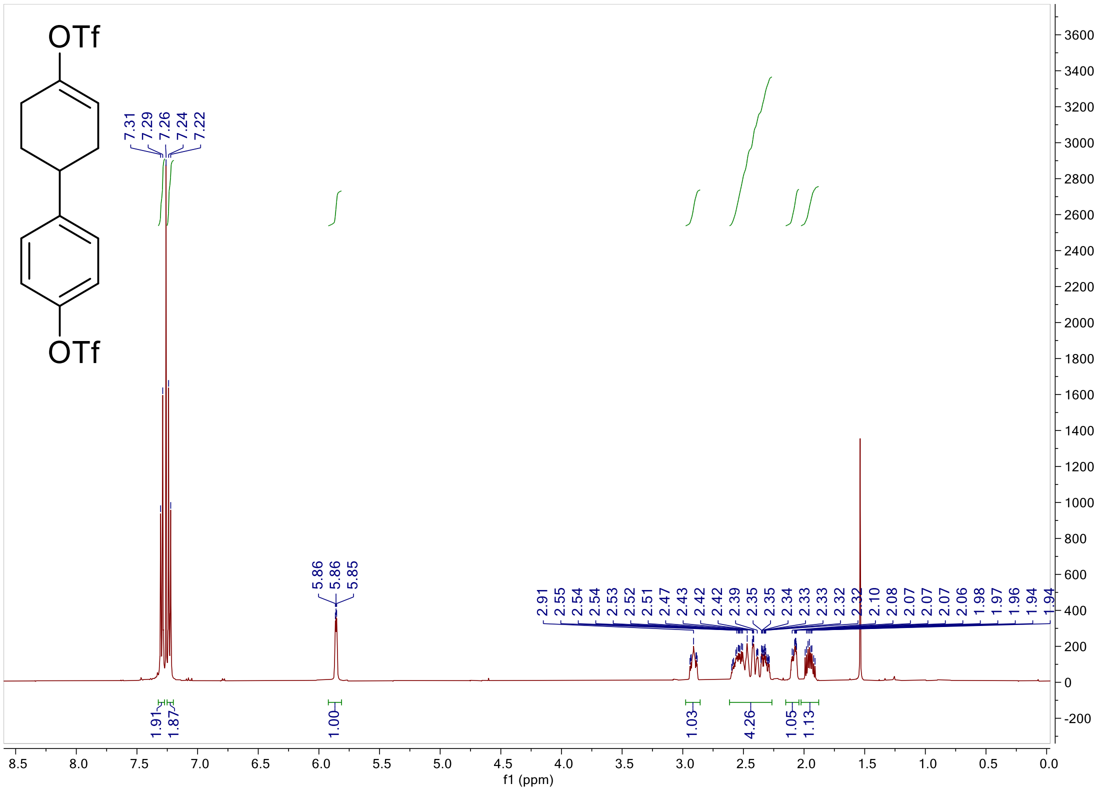

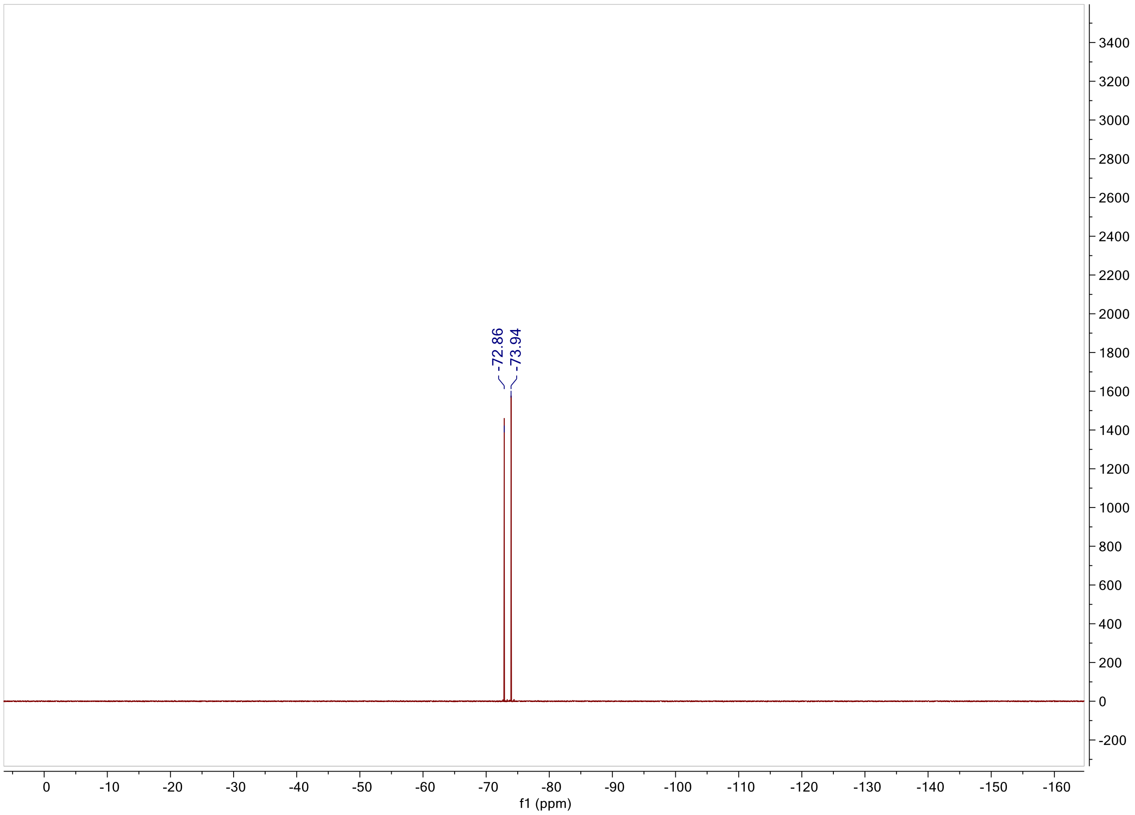

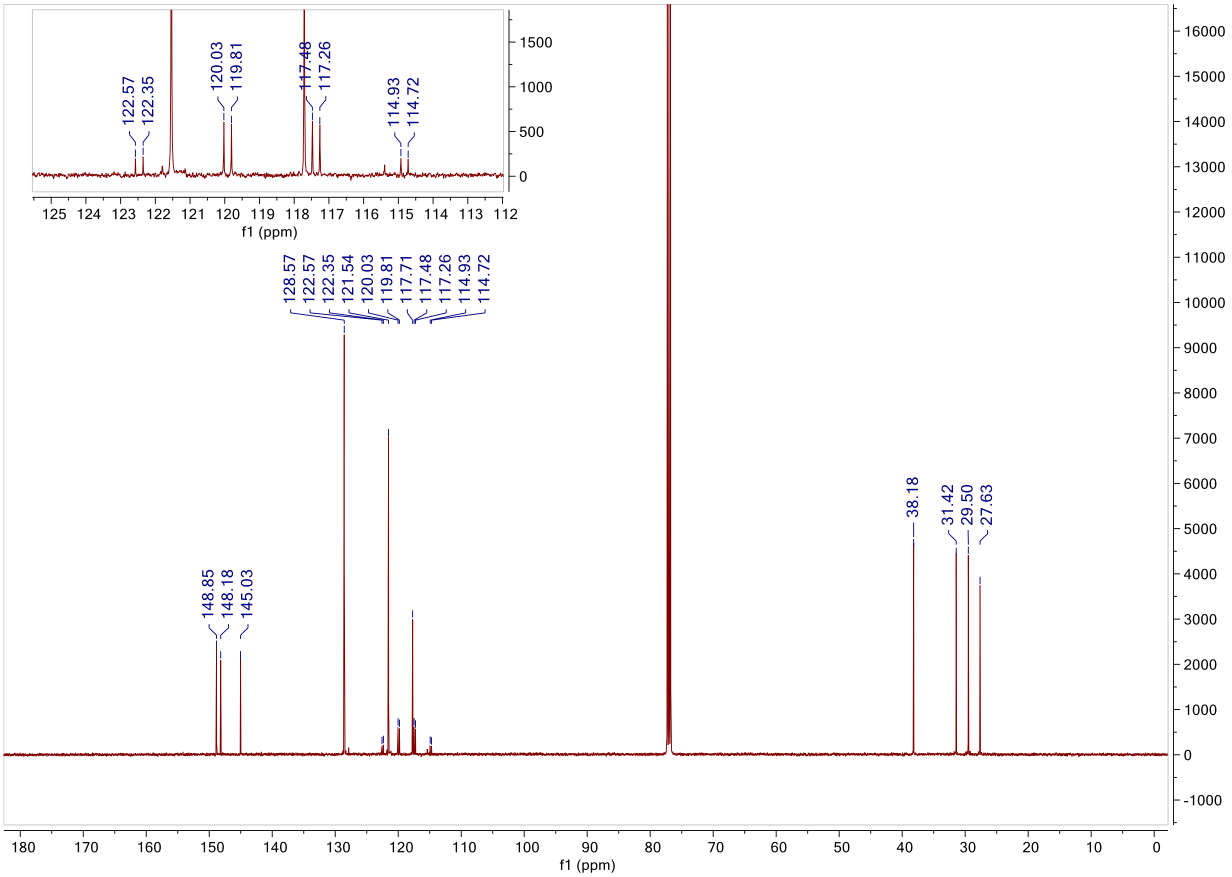

122.57
122.35
120.03
119.81
117.48
117.26
114.93
114.72
125 124 123 122 121 120 119 118 117 116 115 114 113 112
f1 (ppm)
0
500
1000
1500
128.57
122.57
122.35
121.54
120.03
119.81
117.71
117.48
117.26
114.93
114.72
148.85
148.18
145.03
38.18
31.42
29.50
27.63
180 170 160 150 140 130 120 110 100 90 80 70 60 50 40 30 20 10 0
f1 (ppm)
-1000
0
1000
2000
3000
4000
5000
6000
7000
8000
9000
10000
11000
12000
13000
14000
15000
16000
